# Supplementary material for: Cost-effectiveness of insulin pumps compared with multiple daily injections both provided with structured education for adults with type 1 diabetes: a health economic analysis of the Relative Effectiveness of Pumps over Structured Education (REPOSE) randomised controlled trial
Source: BMJ Open. 2018 Apr 7;8(4):e016766. doi: 10.1136/bmjopen-2017-016766 (PMC5893943; doi:10.1136/bmjopen-2017-016766)
Supplement: Supplementary file 1 [file bmjopen-2017-016766supp001.pdf]

Supplementary material A – Economic Evaluation Alongside the clinical trial

Contents

1 Methods 2

1.1 Resource Use by individuals in REPOSE over the 2 year follow up period 2

1.1.1 Treatment switching 7

1.1.2 Estimating the within trial cost effects 7

1.1.3 Estimating within trial QALY effects using EQ-5D and SF-12 7

1.1.4 The statistical model used for the within trial analysis 8

1.1.5 Analysis 8

2 Results 8

2.1 Base Case analysis 8

2.1.1 Summary of the scenario analyses 11

2.1.2 Results of the scenario analyses 12

Table of tables

Table 1: The unit costs used in the within trial analysis of the REPOSE data 3

Table 2: The unit costs of insulin 5

Table 8: Within trial cost-effectiveness analysis results of continuous subcutaneous insulin infusion (pumps) versus multiple daily injections (MDI), both with dose adjustment for normal eating structured education 13

Table of figures

Figure 1: The cost-effectiveness ellipse for the base case within trial analysis 10

Figure 2: The cost-effectiveness acceptability curve for the base case within trial analysis ... 10

List of abbreviations

Abbreviation	Definition
EEACT	economic evaluation alongside the trial
DKA	diabetic ketoacidosis
QALY	Quality adjusted life year
pumps	Insulin pumps
MDI	Multiple daily injections
DAFNE	Dose adjustment for normal eating

1 Methods

1.1 Resource Use by individuals in REPOSE over the 2 year follow up period

Resource use was collected either on an ongoing basis or was self-reported by the individuals in the trial at baseline or at a follow up period (6 months, 1 year and 2 years post-randomisation). All unit costs used to value the reported resource use in the economic evaluation alongside the trial (EEACT), apart from the costs associated with insulin use, are presented in Table **1**. The unit costs associated with insulin use are reported separately in Table 2.

Data was collected on an ongoing basis throughout the trial for all inpatient hospitalisations which were not scheduled to treat a pre-existing condition, incidence of severe hypoglycaemia and whether or not an individual used an insulin pump. At each recorded hospital admission (either self-reported or collected on an ongoing basis) information was collected on the cause. The possible causes for an admission in the REPOSE dataset were: diabetic ketoacidosis (DKA); myocardial infarction; severe hypoglycaemia; ischaemic heart disease; unstable angina; heart failure; foot ulcer; and renal disease. Severe hypoglycaemia was defined in the REPOSE trial as been any hypoglycaemic episode which an individual was unable to treat themselves. If it was reported that an individual did not have an inpatient admission or a paramedic callout for their severe hypoglycaemic event, then it was assumed that a friend or family member provided aid to the individual. These adverse events had no implications for NHS resource use, so these episodes of severe hypoglycaemia were assumed to have no monetary cost to the NHS.

Individual's self-reported resource use since the last REPOSE visit was collected on: the number of face to face and telephone diabetes related contacts with health care professionals; the use of lipid lowering, antiplatelet or depression medication; data at baseline on number of diabetes related admissions in the past year, days spent in hospital and the reason for admission; and the type of insulin used, the average daily dose in the week preceding data collection, the number of injections per day, and the method of insulin delivery. The unit costs associated with insulin use are presented separately from the rest of the unit costs in Table 2. The daily cost of insulin was multiplied by the number of days between each data collection period (6 months, 1 year, and 2 years) to calculate the cost of insulin in the first and second year. If an individual was a receiving insulin pump therapy, the cost of needles, insulin pens and syringes were not applied, as these were already included in the estimates of the cost of insulin pump consumables. From this information a cost of insulin for each individual in the REPOSE trial was calculated.

Table 1: The unit costs used in the within trial analysis of the REPOSE data

Costs used in the within trial analyses	Cost (2013/14)	Notes
**DAFNE courses**		
Cost of a DAFNE course	£363.10	DAFNE fact sheet 6[1]
Cost of a pre-course pump fitting session	£28.82	REPOSE trial data and DAFNE fact sheet 6[1]
**Hypoglycaemia**		
Cost of hypoglycaemia admission	£446.73	NHS reference costs 2013-14.[2] Non-elective inpatient short stay. FCE weighted average of the currency codes: KB01C, KB01D, KB01E, KB01F, KB02G, KB02H, KB02J, KB02K
Paramedic cost per case	£233.58	Elliot *et al* 2014.[3] Table 3.
**Cost of inpatient admissions**		
Diabetic ketoacidosis		
Cost of the first day	£527.78	NHS reference costs 2012-13. Non-elective inpatient short stay. Currency code PA67Z.
Cost of subsequent days	£284.42	NHS reference costs 2012-13. Non-elective inpatients excess bed days. Currency code PA67Z.
Renal hospitalisation		
Cost of the first day	£471.70	NHS reference costs 2013-14.[2]Non-elective inpatients short stay. Weighted average of the currency codes: LA09J, LA09K, LA09L, LA09M, LA09N, LA09P, LA09Q

Cost of subsequent days	£257.87	NHS reference costs 2013-14.[2]Non-elective inpatients excess bed days. Currency codes: LA09J, LA09K, LA09L, LA09M, LA09N, LA09P, LA09Q
Myocardial Infarction		
Cost of the first day	£560.60	NHS reference costs 2013-14.[2]Non-elective inpatients short stay. Weighted average of the currency codes: EB10A, EB10B, EB10C, EB10D, and EB10E.
Cost of subsequent days	£248.89	NHS reference costs 2013-14.[2]Non-elective inpatients excess bed days. Currency codes: EB10A, EB10B, EB10C, EB10D, EB10E.
Foot Ulcer		
Cost of the first day	£509.39	NHS reference costs 2012-13. Non-elective inpatient short stay. Currency codes: KB03C, KB03D, KB03E.
Cost of subsequent days	£156.34	NHS reference costs 2012-13. Non-elective inpatient short stay day. Currency codes: KB03C, KB03D, KB03E.
Other Inpatient stays		
Cost of the first day	£755.44	NHS reference costs 2012-13. Non-elective inpatient short stay. Currency code PA68Z.
Cost of subsequent days	£335.81	NHS reference costs 2012-13. Non elective inpatient excess bed day. Currency code PA68Z.
**Medication costs (per quarter)**		
Cost of lipid medication	£9.27	Prescription Cost Analysis: England 2011 (BNF chapter 2 section 12)
Cost of antiplatelet medication	£1.87	Prescription Cost Analysis: England 2011 (BNF chapter 2 section 9)
Cost of depression medication	£6.08	Prescription Cost Analysis: England 2011 (BNF chapter 4 section 3)
**Cost of diabetes related contacts**		
Cost of a face to face clinic	£105.49	NHS reference costs 2013-14. Non consultant led outpatient attendance. Non admitted face to face follow up. Service description: Diabetic Medicine
Cost of a telephone contact	£75.80	NHS reference costs 2013-14. Non consultant led outpatient attendance. Non admitted non face to face follow up. Service description: Diabetic Medicine

DAFNE – dose adjustment for normal eating MDI, multiple daily injections:; HSCIC, Health & Social Care Information Centre

Table 2: The unit costs of insulin

Item	Average unit cost	Number of units	Cost per unit	Associated yearly cost of an insulin pen	Source
Consumables related to multiple daily injections					
Cost of an insulin needle	£0.11	N/A	N/A	N/A	HSCIC[4]
Cost of an insulin syringe	£0.13	N/A	N/A	N/A	HSCIC[4]
Quick Acting Insulin					
**Human Insulin**					
Vial	£9.87	1000	£0.01	N/A	BNF[5], HSCIC[4]
Cartridges for a reusable pen	£18.97	1500	£0.01	£8.78	
**Animal Insulin**					
Vial	£26.15	1000	£0.03	N/A	BNF[5], HSCIC[4]
Cartridges for a reusable pen	£38.29	1500	£0.03	£5.97	
**Insulin Aspart (NovoRapid)**					
Vial	£14.08	1000	£0.01	N/A	BNF[5], HSCIC[4]
Cartridges for a reusable pen	£28.31	1500	£0.02	£9.59	
Disposable Pen	£30.63	1500	£0.02	N/A	
**Insulin Lispro (Humalog)**					
Vial	£16.61	1000	£0.02	N/A	
Cartridges for a reusable pen	£28.31	1500	£0.02	£8.86	BNF[5], HSCIC[4]
Disposable Pen	£28.31	1500	£0.02	N/A	
**Insulin Glulisine (Apidra)**					
Vial	£16.00	1000	£0.02	N/A	
Cartridges for a reusable pen	£28.30	1500	£0.02	£7.86	BNF[5], HSCIC[4]
Disposable Pen	£28.30	1500	£0.02	N/A	
Background Insulin					
**Human Insulin**					
Vial	£10.41	988	£0.01	N/A	
Cartridges for a reusable pen	£21.52	1500	£0.01	£9.30	BNF[5], HSCIC[4]
Disposable Pen	£21.05	1500	£0.01	N/A	
**Animal Insulin**					
Vial	£26.17	1000	£0.03	N/A	BNF[5], HSCIC[4]
Cartridges for a reusable pen	£38.32	1500	£0.03	£9.57	
**Insulin Detemir (Levemir)**					
Cartridges for a reusable pen	£42.00	1500	£0.03	£9.59	BNF[5], HSCIC[4]
Disposable Pen	£42.10	1500	£0.03	N/A	
**Insulin Glargine (Lantus)**					

Vial	£30.68	1000	£0.03	N/A	
Cartridges for a reusable pen	£41.50	1500	£0.03	£7.86	BNF[5], HSCIC[4]
Disposable Pen	£41.50	1500	£0.03	N/A	
Mixed Insulin					
**Biphasic Isophane Insulin**					
**Animal Insulin**					
Vial	£25.20	1000	£0.03	N/A	BNF[5], HSCIC[4]
Cartridges for a reusable pen	£37.80	1500	£0.03	£5.97	
**Human Insulin**					
Vial	£15.43	987	£0.02	N/A	BNF[5], HSCIC[4]
Cartridges for a reusable pen	£18.94	1500	£0.01	£7.74	
Disposable Pen	£21.43	1500	£0.01	N/A	
**Biphasic Insulin Aspart**					
Cartridges for a reusable pen	£28.79	£28.79	£0.02	£9.59	BNF[5], HSCIC[4]
Disposable Pen	£29.89	£29.89	£0.02		
**Biphasic Insulin Lispro**					
Vial	£16.61	1000	£0.02		
Cartridge for reusable pen	£29.03	1500	£0.02	£8.93	BNF[5], HSCIC[4]
Disposable Pen	£30.13	1500	£0.02		

BNF, British National Formulary; HSCIC, Health & Social Care Information Centre

1.1.1 *Treatment switching*

During the REPOSE trial, it was possible for individuals to switch from insulin delivery mechanism to the other i.e. to switch from insulin pump therapy to multiple daily injections and vice versa. It was possible to switch treatment twice and two individuals did so in the REPOSE trial. The data in the REPOSE trial was analysed to assess the number of people with diabetes who switched treatment. The estimated cost of insulin and insulin pumps were adjusted to reflect the fact that individuals switch treatments.

1.1.2 *Estimating the within trial cost effects*

In the base case analysis, complete cost information was used in the EEACT. Complete total cost information was available for 98%, 90% and 92% of individuals in the intention to treat population at baseline, 1 year and 2 years respectively.

In a scenario analysis missing cost data was imputed for those individuals who attended at least one REPOSE follow up visit. Total discounted cost data was imputed using chained equations (predictive mean matching) using baseline HbA1c, treatment allocation, age at baseline, and baseline cost values as covariates in the imputation equations. 10 different imputed values were calculated for each individual with missing data.

1.1.3 *Estimating within trial QALY effects using EQ-5D and SF-12*

To generate quality adjusted life year (QALY) measures over the two year trial follow-up, information was collected on individual's utility using two different instruments, the EQ-5D and the SF-12. The EQ-5D and SF-12 questionnaires were completed by individuals at baseline and all follow up visits (6 months, 12 months and 24 months).

In the base case within trial analysis, the utility values measured by the EQ-5D-3L were used to calculate QALYs using an area under the curve analysis. EQ-5D utility scores were used in the base case because they are NICE's preferred utility measure.[6] In a scenario analysis, utility values measured using the SF-6D (a measure derived from the SF-12) were used to calculate QALY values.[7] In the base case, only individuals with complete QALY data were included in the analysis. Utilities as measured by EQ-5D-3L were completed by 99%, 93%, 88% and 90% of individuals at baseline, 6, 12 and 24 months respectively. If an individual had a missing 6 month utility value it was assumed that the 6 month utility value

would be the average of the baseline and one year utility values. If an individual had a missing utility score at12 or 24 months, then they were excluded from the base case analysis.

In a scenario analysis, multiple imputation was used to impute missing QALYs values for individuals with an assessment data for least one follow up point. Data was imputed using chained equations (predictive mean matching) using baseline HbA1c, treatment allocation, age at baseline, and baseline cost or QALY values as covariates in the imputation equations. 10 different imputed values were calculated for each individual with missing data in the analyses using imputed data

1.1.4 The statistical model used for the within trial analysis

A seemingly unrelated regression model was used to estimate the costs and QALYs in the EEACT.[8] One seemingly unrelated regression was fitted to four outcome variables: total discounted costs in year 1; total discounted costs in year 2; total discounted QALYs in year 1 and total discounted QALYs in year 2; using the mysureg command in the ml_ado package in STATA version 13.1. For the QALY outcome variables, baseline HbA1c, treatment allocation and baseline utility were included as covariates. Baseline utility was included as a covariate to estimate QALYs so that any baseline differences in health between the two treatment arms was controlled for.[9] For the cost outcome variables, baseline HbA1c, site, treatment allocation and baseline resource use were included as covariates. The standard errors of the seemingly unrelated regression was adjusted for clustering within each DAFNE course.

1.1.5 Analysis

The key measure of cost-effectiveness in the EEACT was the incremental cost-effectiveness ratio base on the mean incremental effect of insulin pumps and dose adjustment for normal eating (pumps+DAFNE) compared to multiple daily injections and dose adjustment for normal eating (MDI+DAFNE) on total costs and total QALYs. The confidence intervals around these effects were estimated from the variance –covariance matrix of the regression model. The results were presented on a cost-effectiveness plane and the uncertainty around the mean effect was presented using a confidence ellipse.

2 Results

2.1 Base Case analysis

The results of the within trial cost-effectiveness analysis are presented using a confidence ellipse in

Figure **1**. In the base case pumps + DAFNE was dominated by MDI + DAFNE as pumps + DAFNE produced fewer mean QALYs at a higher mean cost. The confidence ellipses show pumps + DAFNE was associated with statistically significantly higher costs than MDI + DAFNE at the 5% significance level. The confidence ellipses also show that pumps + DAFNE was not associated with statistically significantly lower QALYs than MDI+DAFNE at the 5% significance level. This is as the confidence ellipse crosses the y axis of the graph at 0. Another point to note is that the confidence ellipses do not cross a threshold ICER of £20,000 per QALY gained; therefore the ICER of pumps + DAFNE compared to MDI + DAFNE is greater than £20,000 per QALY gained at the 95% confidence level.

The cost-effectiveness acceptability curve is presented in Figure 2. It shows that pumps + DAFNE has a 0.0% chance of being cost effective at threshold ICERs of £20,000 per QALY gained and £30,000 per QALY gained.

Table 3 presents the incremental cost and QALY outcomes of pumps + DAFNE compared to MDI + DAFNE in each year of the trial and for both years combined. In the base case the incremental cost in year 2 is lower than the cost in year 1. This is likely due to 1) treatment switching and 2) the rate of DKAs and severe hypoglycaemic events were noticeably lower in the pumps + DAFNE arm in the second year compared to the first year. The incremental QALYs are negative in the first year and positive in the second year. However in neither year is this result statistically significant and in both years, the mean effects are less than 1/100th of a QALY.

Figure 1: The cost-effectiveness ellipse for the base case within trial analysis

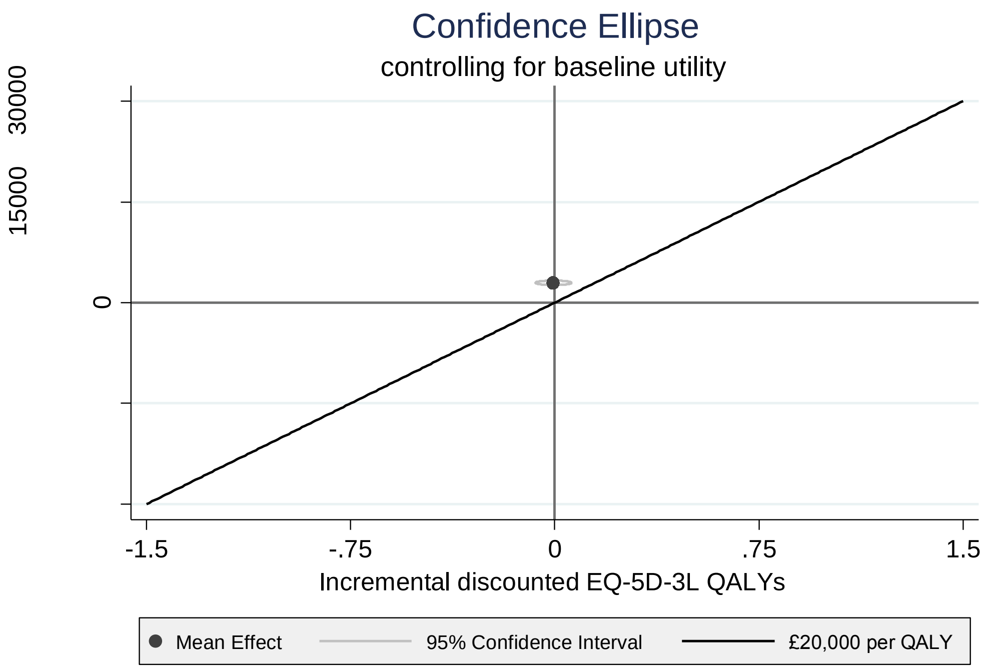

Figure 2: The cost-effectiveness acceptability curve for the base case within trial analysis

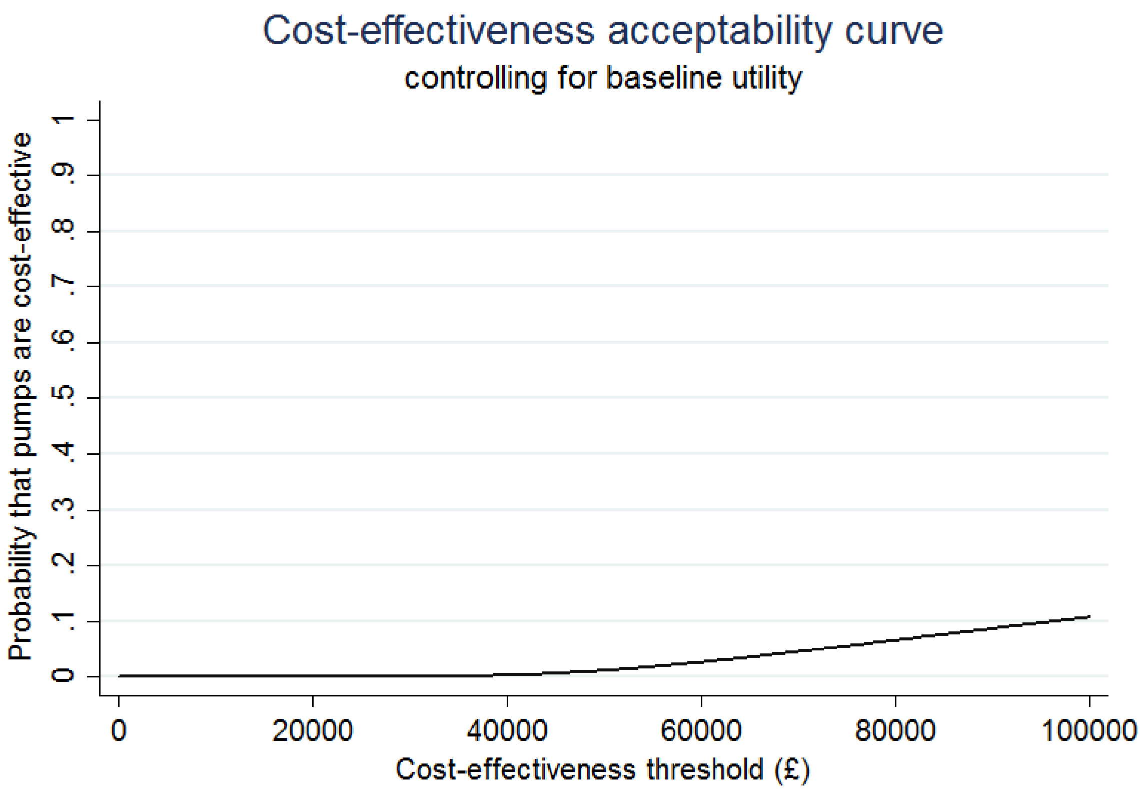

2.1.1 Summary of the scenario analyses

The following scenario analyses were undertaken:

1) Those individuals who adhered to their randomised method of insulin delivery
2) Missing Cost and QALY data were imputed
3) QALYs measured by SF-6D were used instead of QALYs measured using EQ-5D
4) Imputed data and QALYs measured by SF-6D QALYs
5) Pump costs measured by Riemsma et al.[10] were used
6) Riemsma et al. pump costs were used and missing data was imputed
7) The cost of pumps and consumables are 25% lower
8) The cost of pumps and consumables are 25% lower in a per protocol population
9) The cost of pumps and consumables are 50% lower
10) The cost of pumps and consumables are 50% lower in a per protocol population

2.1.2 Results of the scenario analyses

The results of the scenario analyses are also presented in Table 3. It is clear that pumps + DAFNE is dominated by MDI + DAFNE in all analyses, except those conducted in the per protocol population. The lowest ICER is observed in the scenario were the per protocol population is used and there is a cost reduction in insulin pumps and consumables of 50%. The ICER in this scenario is £552,866, which is above the £20,000 to £30,000 per QALY gained threshold considered by NICE.

Table 3: Within trial cost-effectiveness analysis results of continuous subcutaneous insulin infusion (pumps) versus multiple daily injections (MDI), both with dose adjustment for normal eating structured education

		N	Incremental costs in year 1	Incremental costs in year 2	Total incremental costs	Incremental QALYs in year 1	Incremental QALYs in year 2	Total incremental QALYs	ICER (£/QALY gained)
**Base Case** – ITT population with complete costs and QALYs	Mean<br>95% CI	205	£1732<br>(£1511, £1952)	£1228<br>(£1063, £1392)	£2959<br>(£2692, £3227)	-0.007<br>(-0.036,0.022)	0.003<br>(-0.029, 0.035)	-0.004<br>(-0.057, 0.048)	Dominated
**Scenario Analyses**									
Per protocol population	Mean<br>95% CI	188	£1780<br>(£1520, £2041)	£1434<br>(£1328, £1539)	£3214<br>(£2916, £3513)	-0.003<br>(-0.034, 0.027)	0.006<br>(-0.026, 0.037)	0.002<br>(-0.051, 0.056)	£1,369,287
Imputed data	Mean<br>95% CI	260	£1697<br>(£1492, £1901)	£1175<br>(£1006, £1345)	£2872<br>(£2602, £3142)	-0.013<br>(-0.039, 0.014)	0.004<br>(-0.029, 0.037)	-0.009<br>(-0.058, 0.04)	Dominated
SF-6D QALYs	Mean<br>95% CI	196	£1746<br>(£1514, £1978)	£1254<br>(£1096, £1412)	£3000<br>(£2729, £3271)	-0.001<br>(-0.021, 0.019)	-0.002<br>(-0.027, 0.023)	-0.003<br>(-0.045, 0.039)	Dominated
Imputed data and SF-6D QALYs	Mean<br>95% CI	256	£1701<br>(£1494, £1908)	£1186<br>(£1016, £1357)	£2888<br>(£2616, £3159)	-0.003<br>(-0.021, 0.015)	-0.001<br>(-0.024, 0.022)	-0.004<br>(-0.041, 0.034)	Dominated
Riemsma et al. pump costs	Mean<br>95% CI	205	£1679<br>(£1450, £1908)	£1184<br>(£1024, £1343)	£2863<br>(£2586, £3140)	-0.007<br>(-0.036, 0.022)	0.003<br>(-0.029, 0.035)	-0.004<br>(-0.057, 0.048)	Dominated
Imputed data Riemsma et al. pump costs	Mean<br>95% CI	260	£1648<br>(£1434, £1861)	£1125<br>(£964, £1286)	£2772<br>(£2498, £3047)	-0.013<br>(-0.039, 0.014)	0.004<br>(-0.029, 0.037)	-0.009<br>(-0.058, 0.04)	Dominated

Table 3: Within trial cost-effectiveness analysis results of insulin pumps versus multiple daily injections (MDI), both with dose adjustment for normal eating structured education (continued)

		N	Incremental costs in year 1	Incremental costs in year 2	Total incremental costs	Incremental QALYs in year 1	Incremental QALYs in year 2	Total incremental QALYs	ICER (£/QALY gained)
**Base Case** – ITT population with complete costs and QALYs	Mean<br>95% CI	205	£1732<br>(£1511, £1952)	£1228<br>(£1063, £1392)	£2959<br>(£2692, £3227)	-0.007<br>(-0.036,0.022)	0.003<br>(-0.029, 0.035)	-0.004<br>(-0.057, 0.048)	Dominated
**Scenario Analyses**									
The cost of pumps and consumables are 25% lower	Mean<br>95% CI	205	£1285<br>(£1022, £1547)	£955<br>(£850, £1059)	£2239<br>(£1786, £2314)	-0.007<br>(-0.036, 0.022)	0.003<br>(-0.104, 0.11)	-0.004<br>(-0.057, 0.048)	Dominated
The cost of pumps and consumables are 25% lower in a per protocol analysis	Mean<br>95% CI	188	£1223<br>(£1010, £1436)	£768<br>(£634, £902)	£1991<br>(£1939, £2540)	-0.003<br>(-0.034, 0.027)	0.006<br>(-0.026, 0.037)	0.002<br>(-0.051, 0.056)	£966,218
The cost of pumps and consumables are 50% lower	Mean<br>95% CI	205	£767<br>(£532, £1001)	£375<br>(£255, £494)	£1141<br>(£873, £1409)	-0.007<br>(-0.036, 0.022)	0.003<br>(-0.029, 0.035)	-0.004<br>(-0.057, 0.048)	Dominated
The cost of pumps and consumables are 50% lower in a per protocol analysis	Mean<br>95% CI	188	£789<br>(£524, £1053)	£475 (£372, £579)	£1264<br>(£961, £1567)	-0.004<br>(-0.034, 0.027)	0.006<br>(-0.026, 0.037)	0.002<br>(-0.051, 0.056)	£552,866

All costs are reported in 2013/14 prices ;ITT, intention to treat; QALY, quality adjusted life year; CI, confidence interval; ICER, incremental cost-effectiveness ratio

References

1. DAFNE. DAFNE Fact Sheet Six. Online Source. 2012;Available from; http://www.dafne.uk.com/uploads/135/documents/06_factsheetsix_12pt_18_06_12.pdf. Last Accessed 1st July 2015.
2. Department of Health. NHS reference costs 2013 to 2014. Online Source. 2014;Available from; https://www.gov.uk/government/publications/nhs-reference-costs-2013-to-2014. Last accessed 17th July 2015.
3. Elliott J, Jacques RM, Kruger J, Campbell MJ, Amiel SA, Mansell P et al. Substantial reductions in the number of diabetic ketoacidosis and severe hypoglycaemia episodes requiring emergency treatment lead to reduced costs after structured education in adults with Type 1 diabetes. Diabet Med. 2014;31(7):847-53. doi:10.1111/dme.12441.
4. Health & Social Care Information Centre. Prescription Cost Analysis England 2014. Online Source. 2015;Available from; http://www.hscic.gov.uk/catalogue/PUB17274. Last Accessed 2nd Novemeber 2015.
5. British Medical Association and Royal Pharmaceutical Society of Great Britain. British National Formulary August 2015. Online Source. 2015;Available from: https://www.medicinescomplete.com, Last Accessed 9th September 2015.
6. National Institute for Health and Care Excellence. Guide to the methods of technology appraisal 2013. Online Source. 2013;Available from; http://www.nice.org.uk/article/pmg9/resources/non-guidance-guide-to-the-methods-of-technology-appraisal-2013-pdf. Last accessed 13th July 2015.
7. Brazier JE, Roberts J. The estimation of a preference-based measure of health from the SF-12. Med Care. 2004;42(9):851-9.
8. Zellner A. An Efficient Method of Estimating Seemingly Unrelated Regressions and Tests for Aggregation Bias. J Am Stat Assoc. 1962;57(298):348-&.
9. Manca A, Hawkins N, Sculpher MJ. Estimating mean QALYs in trial-based cost-effectiveness analysis: the importance of controlling for baseline utility. Health Econ. 2005;14(5):487-96. doi:10.1002/hec.944.
10. Riemsma R, Corro Ramos I, Birnie R, Büyükkaramikli N, Armstrong N, Ryder S et al. Type 1 diabetes: Integrated sensor-augmented pump therapy systems for managing blood glucose levels (The MiniMed Paradigm Veo System and the Vibe and G4 PLATINUM CGM system), a systematic review and economic evaluation. Kleijnen Systematic Reviews Ltd. 2015.
