# Supplementary material for: Cost-effectiveness of insulin pumps compared with multiple daily injections both provided with structured education for adults with type 1 diabetes: a health economic analysis of the Relative Effectiveness of Pumps over Structured Education (REPOSE) randomised controlled trial
Source: BMJ Open. 2018 Apr 7;8(4):e016766. doi: 10.1136/bmjopen-2017-016766 (PMC5893943; doi:10.1136/bmjopen-2017-016766)
Supplement: Supplementary file 2 [file bmjopen-2017-016766supp002.pdf]

Supplementary material B – Supporting information for the long term modelling

Table of Contents

1 Determining the number of PSA runs 1

2 Determining the number of simulated individuals to use in each run 1

3 Other cause mortality 1

4 Probability of death from end stage renal disease 1

5 The clinical effectiveness parameters 2

5.1 Treatment switching 2

5.2 HbA1c 14

5.3 DKA and severe hypoglycaemia 21

6 The cost of insulin, diabetes related contacts and insulin pumps used in the Sheffield Type 1 Diabetes model 25

7 Detailed results of the scenario and subgroup analyses 32

List of Tables

Table 1: The results of the parametric survival models fitted to patients allocated to the continuous subcutaneous insulin infusion dose adjustment for normal eating arm of the REPOSE trial (n=130) ... 4

Table 2: The results of the parametric survival models fitted to patients allocated to the multiple daily injections with dose adjustment for normal eating arm of the REPOSE trial (n=128) 6

Table 3: A summary of the Akaike information criterion and the Bayesian information criterion for the fitted survival curves used in the long term modelling 8

Table 4: The variance – covariance matrix for the exponential model fitted to the pumps arm 11

Table 5: The variance – covariance matrix for the exponential model fitted to the multiple daily injections arm 11

Table 6: The variance – covariance matrix for the weibull model fitted to the pumps arm 11

Table 7: The variance – covariance matrix for the weibull model fitted to the multiple daily injections arm 11

Table 8: The variance – covariance matrix for the gompertz model fitted to the pumps arm 11

Table 9: The variance-covariance matrix for the gompertz model fitted to the multiple daily injections arm 12

Table 10: The variance – covariance matrix for the log-logistic model fitted to the pumps arm 12

Table 11: The variance – covariance matrix for the log-logistic model fitted to the multiple daily injections arm 12

Table 12: The variance – covariance matrix for the log-normal model fitted to the pumps arm 12

Table 13: The variance – covariance matrix for the log-normal model fitted to the multiple daily injections arm 13

Table 14: The effect of continuous subcutaneous insulin infusion compared to multiple daily injections for all individuals in the intention to treat population. 15

Table 15: The effect of continuous subcutaneous insulin infusion compared to multiple daily injections for all individuals in the per protocol population. 16
Table 18: The variance covariance matrix for the beta regression to predict one year HbA1c in the ITT population 17
Table 19**:** The variance covariance matrix for the beta regression to predict two year HbA1c in the ITT population 18
Table 16: The variance covariance matrix for the beta regression to predict one year HbA1c in the per protocol population 19
Table 17: The variance covariance matrix for the beta regression to predict two year HbA1c in the per protocol population 20
Table 20: A summary of the observed incidence of diabetic ketoacidosis and severe hypoglycaemia in the intention to treat population 22
Table 21: The negative binomial model fitted to the incidence of severe hypoglycaemia at one year and two years 24
Table 22: The negative binomial model fitted to the incidence of diabetic ketoacidosis at one and two years 24
Table 23: The unit costs of insulin 25
Table 24: The variance covariance matrix for the seemingly unrelated regression on insulin costs in year 1 and 2 of REPOSE 29
Table 25: The variance covariance matrix for the seemingly unrelated regression on the cost of diabetes related contacts with health care professionals in year 1 and 2 of REPOSE 30
Table 26: The variance covariance matrix for the seemingly unrelated regression on the cost of insulin pumps and associated consumables in year 1 and 2 of REPOSE 31
Table 27: The One way scenario analyses and subgroup analyses performed using the Sheffield Type 1 Diabetes Policy Model 32

List of Figures

Figure 1: A visual plot of the Kaplan-Meier and survival curves estimated from parametric models for those individuals who were randomised continuous subcutaneous insulin infusion with dose adjustment for normal eating 9
Figure 2: A visual plot of the Kaplan-Meier and survival curves estimated from parametric models for those individuals who were randomised multiple daily injections with dose adjustment for normal eating 10

List of abbreviations in this supplementary material

**Abbreviation**	**Definition**
iNMB	Incremental net monetary benefit
QALY	Quality adjusted life year
PSA	Probabilistic sensitivity analysis
UK	United Kingdom
ESRD	End stage renal disease
DKA	Diabetic ketoacidosis
Pumps	Insulin pumps
MDI	Multiple Daily Injections
DAFNE	Dose adjustment for normal eating
T1DM	Type 1 diabetes mellitus

AIC	Akaike information criterion
BIC	Bayesian information criterion
ITT	Intention to treat
DCCT	Diabetes Control and Complications Trial

1 Determining the number of PSA runs

The stability of the model results was assessed in terms of the stability of incremental net monetrary benefit (iNMB) using a threshold cost per quality adjusted life year (QALY) of £20,000 per QALY gained. This statistic is useful for the purpose of assessing model stability as, so long as the mean iNMB is statistically significantly different from 0 at the 5% level then the results are sufficiently stable that any decision made as a consequence of them will not change.

$$Z_i = the\ iNMB\ for\ each\ PSA\ run$$

$$N = the\ number\ of\ PSA\ simulations$$

$$The\ mean\ iNMB\ is\ given\ by\text{:}\ \bar{Z} = \frac{1}{N}\sum_{i=1}^{N} Z_i$$

$$The\ variance\ in\ mean\ iNMB\ is\ given\ by\text{:}\ Var(\bar{Z}) = \frac{1}{N-1}\sum_{i=1}^{n}(Z_i - \bar{Z})$$

$$The\ standard\ error\ of\ the\ mean\ iNMB\ is\ given\ by\text{:}\ Standard\ error(\bar{Z}) = \sqrt{Var(\bar{Z})/N}$$

The mean incremental net monetary benefit at £20,000 per QALY gained was -£16,201 ($\bar{Z}$). The variance of this was 2.18E+08. The standard error was $\sqrt{2.18E + 8/500} = 661$. As the standard error was under 5% of the mean incremental net monetary benefit, 500 probabilistic sensitivity analysis (PSA) runs was determined to be sufficiently robust for making reliable decisions for these comparators

2 Determining the number of simulated individuals to use in each run

Likewise to determine the stability of the ICER in the deterministic results and the scenario analyses the stability of the iNMB was assessed over 5,00 simulated individuals. The model was run initially using the same 5,000 simulated individuals in each arm of the deterministic base case. The mean incremental net monetary benefit at £20,000 per QALY gained was -£15,759 ($\bar{z}$). The variance of this was 1.27E+10. The standard error was $\sqrt{1.27E + 10/5000} = 1{,}594$. As the standard error was approximately 10% of the mean incremental net monetary benefit, 5000 simulated individuals was determined to be sufficient to produce stable results for the analyses.

3 Other cause mortality

Individuals can also die from other causes. This other-cause mortality was updated in version of the model used for these analyses. Other-cause mortality was calculated using UK life tables from 2012 to 2014 adjusted to exclude the causes either attributed to diabetes mellitus (either type 1, type 2 or unspecified, ICD-10 codes E10-14) or modelled directly in the microvascular and macrovascular disease components (deaths due to: end stage renal disease; myocardial infarction, stroke and heart failure, ICD-10 codes N18, I20-21, I61-64, I50).[1-4]

4 Probability of death from end stage renal disease

This model parameter was altered from the value reported in Heller *et al*.[5] to reflect directly observable data available in Wolfe *et al*.[6] on the probability of end stage renal disease. At baseline, 102,163 patients with diabetes were receiving dialysis and over a maximum follow up of 6 years 44,916 of these patients died. This gave a probability of death from end stage renal disease (ESRD) of 44.00% over 6 years. In the model probabilistic sensitivity analysis, the uncertainty in this probability was parameterised using a beta distribution with an alpha parameter of 44,916 and a beta parameter of 57,247. The probability of death from end stage renal disease over 6 years was first converted into an instantaneous rate of death and then yearly probability of death from end stage renal disease using the method in Briggs et al.[7]

5 The clinical effectiveness parameters

5.1 Treatment switching

The treatment switching curves were used to estimate the incidence of treatment switching in the model in the first and second year. Covariates were used in the parametric models to control for: HbA1c prior to switching, number of diabetic ketoacidosis events (DKAs) and number of severe hypoglycaemic events in the year prior to switching (or at 2 years follow up if no switching occurred). The standard errors of the parametric survival models were adjusted for clustering in each DAFNE course. As separate models were fitted to the insulin pumps + dose adjustment for normal eating (pumps+DAFNE) and the multiple daily injections + dose adjustment for normal eating (MDI+DAFNE) arm, no assumption of proportional hazards or accelerated failure time was made.

The curves were not extrapolated, as the clinical expert opinion of a Professor of Clinical Diabetes & Honorary Consultant Physician and a Professor in Public Health & Health Technology Assessment was that if an individual was still using a pump after two years that they would continue to use pump as in their experience once an adult with type 1 diabetes mellitus (T1DM) was successfully using an insulin pump they were unlikely change their insulin delivery method.

The different parameters of the parametric survival models fitted to the pumps+DAFNE arm is given in Table 1. The equivalent parameters are given for the MDI+DAFNE arm in Table 2.

The goodness of fit of the parametric survival curves were assessed using the Akaike information criterion (AIC), Bayesian information criterion (BIC) and a visual assessment of the survival curves estimated from parametric models plotted against the nonparametric Kaplan-Meier curve . The one and two-year time points are the time points of relevance for assessing the visual fit of the curves in this analysis, as the model uses a yearly time cycle so treatment switching is only predicted in the model at these time points. The AIC and the BIC are given in Table 3. Lower values for these statistics indicate a better model fit. For the pumps+DAFNE arm, the exponential model has the lowest AIC and BIC. For the MDI+DAFNE arm, the Weibull model had the lowest AIC and the exponential model has the lowest BIC. The plot of the parametric survival curves against the underlying Kaplan-Meier curve are provided in Figures 1 and 2. It is clear that the exponential model provides a reasonable fit to the Kaplan-Meier for the pumps+DAFNE arm at the one and two-year time points therefore this curve was used in the base case economic model. It is also clear that for the MDI+DAFNE arm, the exponential curve does not provide a reasonable fit to the Kaplan –Meier curve at one year whereas the Weibull curve provides a reasonable fit at both one and two years. Therefore the Weibull curve was used as the base case curve for the MDI+DAFNE model arm.

The uncertainty in the parametric survival curves was included in the model’s probabilistic sensitivity analysis using a multivariate normal distribution. The variance-covariance matrices and the predicted

coefficients for each of the parametric survival models were used to parameterise the multivariate normal distributions. The coefficients are given in Table 1and Table 2 respectively and the variance – covariance matrices for each of the parametric curves is given in Tables 4 toTable **13**.

Table 1: The results of the parametric survival models fitted to patients allocated to the pumps with dose adjustment for normal eating arm of the REPOSE trial (n=130)

Parameter	Coefficient	Robust standard error	95% confidence interval	
Exponential model<br>S(t) = Exp(-Exp(FV)*time),<br>FV = 0.222*HbA1c+-0.972*number of DKAs + 0.427*number of severe hypos -4.616				
HbA1c	0.222	0.241	-0.251	0.695
Number of DKAs	-0.972	0.474	-1.901	-0.042
Number of severe hypoglycaemic events	0.427	0.087	0.257	0.598
Constant	-4.616	2.125	-8.781	-0.451
Weibull model<br>S(t) = Exp(-Exp(FV)*time^Exp(Ln Scale))<br>FV = 0.221*HbA1c+-0.981*number of DKAs + 4.460*number of severe hypos -4.460				
HbA1c	0.221	0.234	0.016	0.694
Number of DKAs	-0.981	0.471	-7.113	-4.910
Number of severe hypoglycaemic events	0.404	0.085	0.337	0.684
Constant	-4.460	2.100	-10.607	-4.696
Ln Scale	-0.258	0.220	0.111	1.377
Gompertz model<br>S(t) = (-Exp(FV)* (1/Gamma))*(Exp(Gamma*time)-1)<br>FV = = 0.220*HbA1c+-0.983*number of DKAs + 0.407*number of severe hypos -4.307				
HbA1c	0.220	0.236	-0.243	0.684
Number of DKAs	-0.983	0.468	-1.901	-0.065
Number of severe hypoglycaemic events	0.407	0.090	0.230	0.584
Constant	-4.307	2.232	-8.682	0.068
Gamma	-0.316	0.479	-1.256	0.624
Log logistic model<br>S(t) = 1/ (1+(1/Exp(FV)*time)^(1/Exp(Gamma))<br>FV = -0.294*HbA1c+1.406*number of DKAs + -0.554*number of severe hypos +5.637				
HbA1c	-0.294	0.286	-0.855	0.267
Number of DKAs	1.406	0.676	0.081	2.730
Number of severe hypoglycaemic events	-0.554	0.170	-0.887	-0.220
Constant	5.637	2.510	0.718	10.557
Gamma	0.215	0.230	-0.235	0.665
Log normal model<br>S(t) = 1 – Φ ((ln(time) – FV)/ Standard Deviation), were Φ is the standard normal distribution<br>FV = -0.307*HbA1c+ 1.867*number of DKAs + -0.656*number of severe hypos +6.406				
HbA1c	-0.307	0.292	-0.879	0.264
Number of DKAs	1.867	0.755	0.387	3.347
Number of severe hypoglycaemic events	-0.656	0.180	-1.009	-0.304
Constant	6.406	2.520	1.466	11.346
St Dev	1.002	0.206	0.598	1.405

DKA, diabetic ketoacidosis; ln, natural logarithm

DKA, diabetic ketoacidosis; ln, natural logarithm

Table 2: The results of the parametric survival models fitted to patients allocated to the multiple daily injections with dose adjustment for normal eating arm of the REPOSE trial (n=128)

Parameter	Coefficient	Robust standard error	95% confidence interval	
Exponential model<br>S(t) = Exp(-Exp(FV)*time),<br>FV = 0.336*HbA1c+-5.5555*number of DKAs + 0.460*number of severe hypos -6.725				
HbA1c	0.336	0.164	0.014	0.657
Number of DKAs	-5.555	0.561	-6.655	-4.455
Number of severe hypoglycaemic events	0.460	0.074	0.315	0.605
Constant	-6.725	1.450	-9.567	-3.884
Weibull model<br>S(t) = Exp(-Exp(FV)*time^Exp(Ln Scale))<br>FV = 0.335*HbA1c+-6.012*number of DKAs + 0.510*number of severe hypos -7.652				
HbA1c	0.355	0.173	0.016	0.694
Number of DKAs	-6.012	0.562	-7.113	-4.910
Number of severe hypoglycaemic events	0.510	0.089	0.337	0.684
Constant	-7.652	1.508	-10.607	-4.696
Ln Scale	0.744	0.323	0.111	1.377
Gompertz model<br>S(t) = (-Exp(FV)* (1/Gamma))*(Exp(Gamma*time)-1)<br>FV = = 0.350*HbA1c+-6.009*number of DKAs + 0.512*number of severe hypos -8.080				
HbA1c	0.350	0.170	0.016	0.683
Number of DKAs	-6.009	0.562	-7.110	-4.908
Number of severe hypoglycaemic events	0.512	0.094	0.329	0.696
Constant	-8.080	1.471	-10.963	-5.197
Gamma	1.055	0.669	-0.256	2.366
Log logistic model<br>S(t) = 1/ (1+(1/Exp(FV)*time)^(1/Exp(Gamma))<br>FV = -0.181*HbA1c+2.609*number of DKAs + -0.232*number of severe hypos +3.676				
HbA1c	-0.181	0.121	-0.418	0.055
Number of DKAs	2.609	0.799	1.044	4.175
Number of severe hypoglycaemic events	-0.232	0.070	-0.368	-0.095
Constant	3.676	1.317	1.094	6.258
Gamma	-0.780	0.317	-1.401	-0.160
Log normal model<br>S(t) = 1 – Φ ((ln(time) – FV)/ Standard Deviation), were Φ is the standard normal distribution<br>FV = -0.190*HbA1c+ 1.617*number of DKAs + -0.283*number of severe hypos +4.117				
HbA1c	-0.190	0.107	-0.400	0.021
Number of DKAs	1.617	0.517	0.603	2.630
Number of severe hypoglycaemic events	-0.283	0.101	-0.481	-0.086
Constant	4.117	1.291	1.587	6.647
Standard Deviation	0.066	0.338	-0.596	0.728

DKA, diabetic ketoacidosis; ln, natural logarithm

DKA, diabetic ketoacidosis; ln, natural logarithm

Table 3: A summary of the Akaike information criterion and the Bayesian information criterion for the fitted survival curves used in the long term modelling

	Parametric survival model	AIC (smaller is better)	BIC (smaller is better)	-2*log pseudo likelihood
pumps+DAFNE	Exponential	**145.77**	**157.24**	137.77
	Weibull	146.46	160.80	136.46
	Gompertz	147.25	161.59	137.25
	Log logistic	147.49	161.83	137.49
	Log normal	148.48	162.82	138.48
MDI + DAFNE	Exponential	64.36	**75.77**	56.36
	Weibull	**62.55**	76.81	52.55
	Gompertz	63.76	78.02	53.76
	Log logistic	63.78	78.04	53.78
	Log normal	64.97	79.23	54.97

AIC - Akaike information criterion; BIC - Bayesian information criterion; DAFNE – dose adjustment for normal eating; MDI – multiple daily injections;

Figure 1: A visual plot of the Kaplan-Meier and survival curves estimated from parametric models for those individuals who were randomised continuous subcutaneous insulin infusion with dose adjustment for normal eating

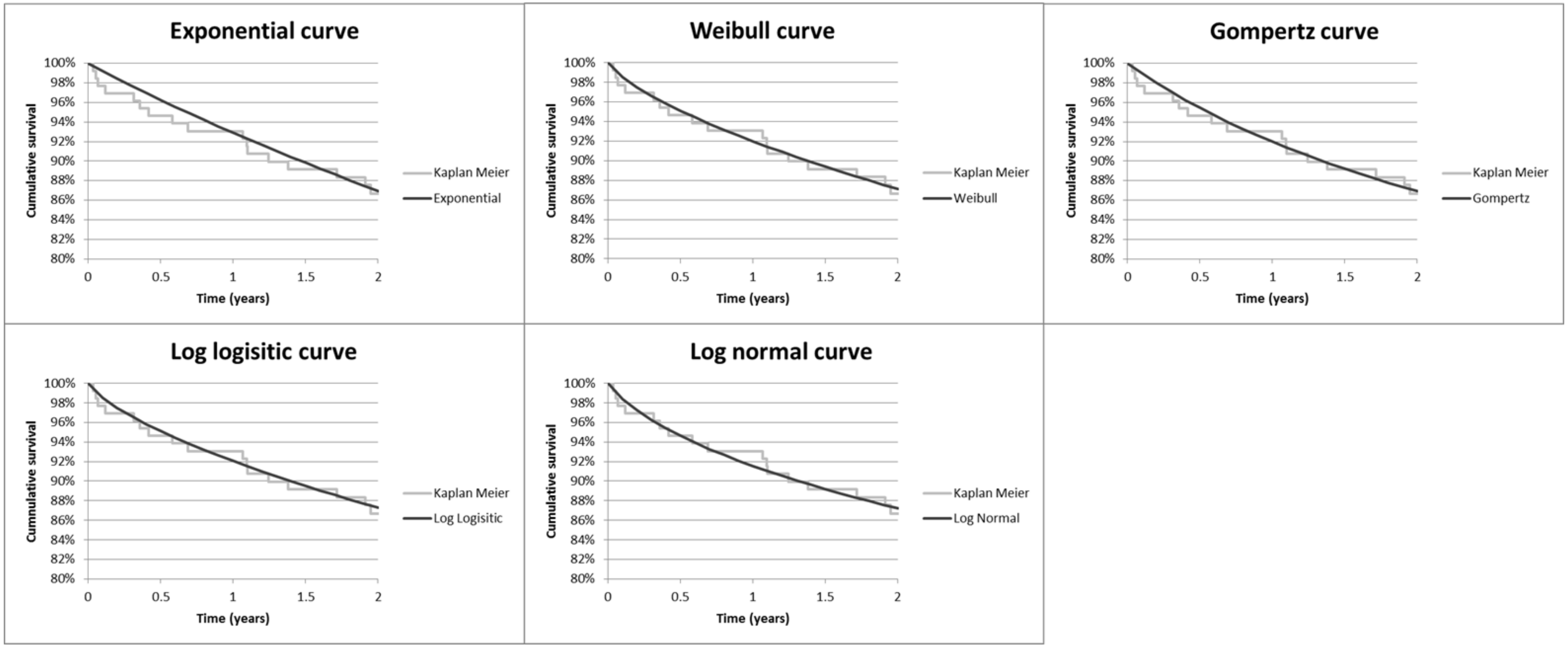

Figure 2: A visual plot of the Kaplan-Meier and survival curves estimated from parametric models for those individuals who were randomised multiple daily injections with dose adjustment for normal eating

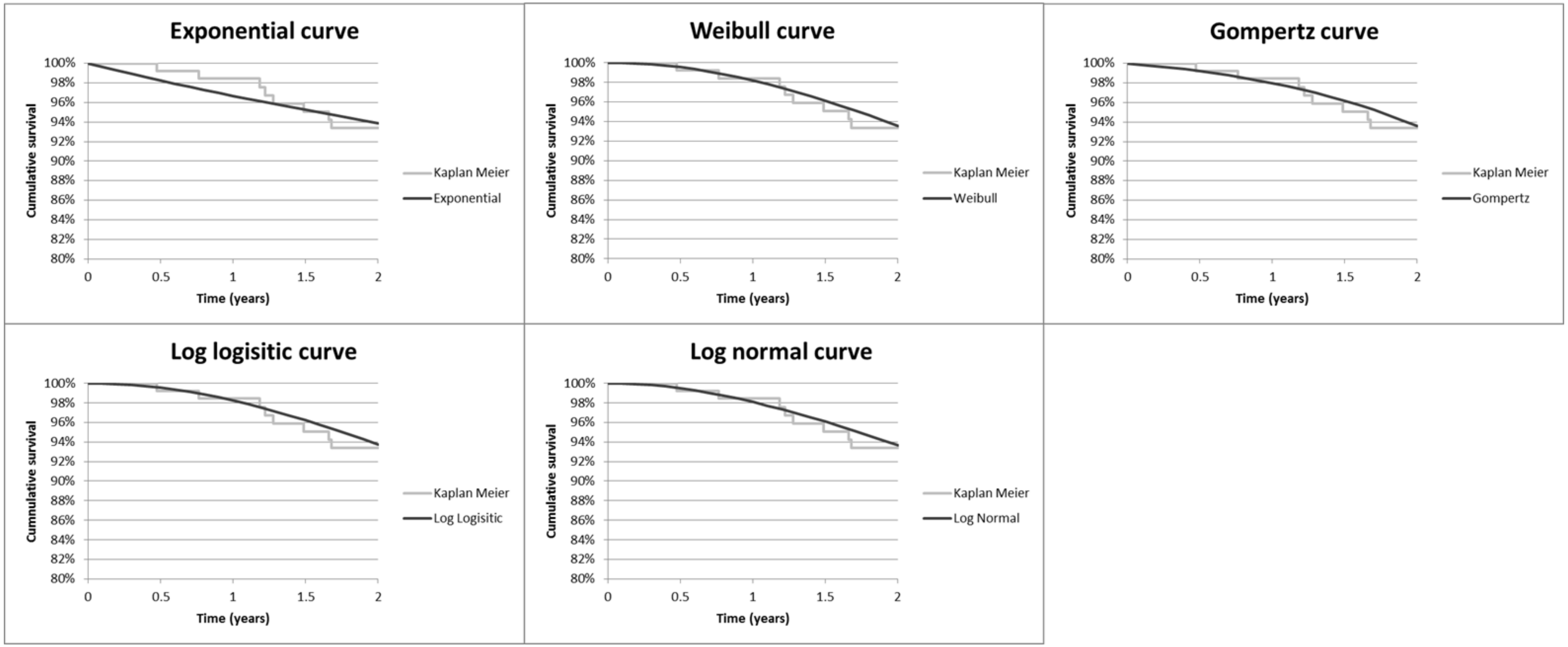

Table 4: The variance – covariance matrix for the exponential model fitted to the pumps arm

	HbA1c	DKAs	Sev Hypos	Constant
HbA1c	0.06			
DKAs	0.06	0.22		
Sev Hypos	0.00	0.00	0.01	
Constant	-0.51	-0.56	-0.02	4.52

Table 5: The variance – covariance matrix for the exponential model fitted to the multiple daily injections arm

	HbA1c	DKAs	Sev Hypos	Constant
HbA1c	0.03			
DKAs	0.00	0.31		
Sev Hypos	0.00	0.01	0.01	
Constant	-0.22	-0.15	0.01	2.10

Table 6: The variance – covariance matrix for the weibull model fitted to the pumps arm

	HbA1c	DKAs	Sev Hypos	Constant	Ln scale parameter
HbA1c	0.05				
DKAs	0.06	0.22			
Sev Hypos	0.00	0.00	0.01		
Constant	-0.49	-0.54	-0.04	4.41	
Ln scale parameter	0.01	0.04	0.01	-0.15	0.05

Table 7: The variance – covariance matrix for the weibull model fitted to the multiple daily injections arm

	HbA1c	DKAs	Sev Hypos	Constant	Ln scale parameter
HbA1c	0.03				
DKAs	0.00	0.32			
Sev Hypos	-0.01	0.02	0.01		
Constant	-0.23	-0.16	-0.01	2.27	
Ln scale parameter	-0.01	0.01	0.01	-0.07	0.10

Table 8: The variance – covariance matrix for the gompertz model fitted to the pumps arm

	HbA1c	DKAs	Sev Hypos	Constant	**Gamma**
HbA1c	0.06				
DKAs	0.06	0.22			

Sev Hypos	0.00	0.00	0.01		
Constant	-0.51	-0.55	-0.05	4.98	
Gamma	0.03	0.03	0.02	-0.46	**0.23**

Table 9: The variance-covariance matrix for the gompertz model fitted to the multiple daily injections arm

	HbA1c	DKAs	Sev Hypos	Constant	Gamma
HbA1c	0.03				
DKAs	0.00	0.32			
Sev Hypos	-0.01	0.02	0.01		
Constant	-0.18	-0.15	-0.03	2.16	
Gamma	-0.04	0.01	0.04	-0.28	0.45

Table 10: The variance – covariance matrix for the log-logistic model fitted to the pumps arm

	HbA1c	DKA	sev hypos	Constant	Gamma
HbA1c	0.08				
DKA	0.03	0.46			
sev hypos	0.01	-0.04	0.03		
Constant	-0.69	0.02	-0.15	6.30	
Gamma	0.01	0.11	-0.01	0.06	0.05

Table 11: The variance – covariance matrix for the log-logistic model fitted to the multiple daily injections arm

	HbA1c	DKA	sev hypos	Constant	Gamma
HbA1c	0.01				
DKA	-0.04	0.64			
sev hypos	0.00	-0.04	0.00		
Constant	-0.15	0.70	-0.05	1.74	
Gamma	-0.02	0.24	-0.02	0.31	0.10

Table 12: The variance – covariance matrix for the log-normal model fitted to the pumps arm

	HbA1c	Number of DKAs	Number of severe hypos	Constant	Standard deviation
HbA1c	0.09				
DKA	-0.01	0.57			
Sev hypos	0.01	-0.06	0.03		
Constant	-0.69	0.42	-0.18	6.35	
Standard deviation	0.01	0.11	-0.02	0.07	0.04

Table 13: The variance – covariance matrix for the log-normal model fitted to the multiple daily injections arm

	HbA1c	DKA	Sev hypos	Constant	Ln sigma parameter
HbA1c	0.01				
DKA	-0.02	0.27			
Sev hypos	0.00	-0.04	0.01		
Constant	-0.12	0.44	-0.08	1.67	
Ln sigma parameter	-0.01	0.17	-0.03	0.33	0.11

5.2 HbA1c

Two beta regressions were used to estimate each individual's HbA1c in the first and second year respectively. Bounds were placed on the beta regression so that HbA1c was between the clinically plausible bounds of 29mmol/mol [4.8%] and 201 mmol/mol [20.5%]. Beta regressions estimate two parameters of interest, the mean effect and the dispersion on the variance. Treatment allocation, baseline HbA1c and centre were included as covariates for the mean effect on HbA1c after one year. Treatment allocation, baseline HbA1c, one year HbA1c and centre were included as covariates for the mean effect on HbA1c after two years. HbA1c in the previous year was used as a covariate for the dispersion parameters. The standard errors of both statistical models were adjusted for clustering within each DAFNE course. Due to presence of treatment switching, an individual's HbA1c was assumed to change as thought they had been allocated to the other trial arm. Therefore, the beta regressions were estimated in the per protocol population (switchers excluded) rather than the intention to treat (ITT) population (switchers included in their randomised arm). A sensitivity analysis was conducted in which the beta regressions were estimated in the ITT population.

The results of the beta regression in the intention to treat population is given in Table 14 and the beta regression estimated in the per protocol population is given in Table 15. The results of these beta regressions are not easily interpretable as changes in HbA1c, as a logit link function is used to estimate the mean effect parameter and the natural logarithm of the dispersion parameter is estimated instead of the dispersion parameter itself.

Missing data was observed for HbA1c values in the per protocol population at 6 months (2.1% missing), 1 year (4.2% missing) and 2 years (4.2% missing). A multiple imputation procedure was employed in individuals with at least one HbA1c value (at 6 or 12 months) after randomisation but no HbA1c value at 24 months. In line with the statistical analysis plan, missing 24 month HbA1c data was imputed by multiple imputation using chained equations (regression) based on 10 imputed data sets with baseline, 6 and 12 months HbA1c measurements, DAFNE course, centre, age, sex, and HFS worry as covariates, if a participant had some HbA1c follow-up data. This imputation procedure was conducted in the ITT population, prior to running the beta regressions. After imputation 236 out of 236 participants in the per protocol population and 259 out of 260 participants in the ITT population had HbA1c follow up data.

The uncertainty in the parametric survival curves was included in the model's probabilistic sensitivity analysis using a multivariate normal distribution. The variance-covariance matrices and the predicted coefficients for each of the beta regressions were used to parameterise the multivariate normal distributions. The coefficients are given in Table 14 and Table 15 respectively and the variance – covariance matrices for each of the beta regressions is given in Table 16 to Table 19 .

Each individual's mean effect parameter and dispersion parameter were used to calculate the expected variance in that individual's actual HbA1c outcome. The individual's predicted mean effect and variance in their mean effect were used to parameterise a beta distribution. Each individual's HbA1c in the model was sampled from the beta distribution which was parameterised by their individualised mean effect and variance. This random draw was then transformed onto the Diabetes Control and Complications Trial (DCCT) (%) scale for use in the risk equations of the model.

Table 14: The effect of continuous subcutaneous insulin infusion compared to multiple daily injections for all individuals in the intention to treat population.

Hba1c at one year (beta scale)	Coefficient	Standard error	t	P>t	95% Confidence interval	
Mean effect (Mu) – using a logit link function						
Treatment allocation (1= pumps + DAFNE, 0=MDI + DAFNE)	-0.056	0.038	-1.49	0.137	-0.131	0.018
Baseline HbA1c (beta scale)	3.978	0.248	16.01	0	3.491	4.465
Constant	-2.223	0.088	-25.28	0	-2.395	-2.050
Centre effects (Cambridge is the reference site)						
Dumfries and Galloway	-0.025	0.074	-0.33	0.738	-0.171	0.121
Edinburgh	-0.019	0.065	-0.3	0.768	-0.147	0.108
Glasgow	-0.154	0.099	-1.55	0.12	-0.348	0.040
Harrogate	0.022	0.041	0.52	0.602	-0.060	0.103
College Hospital - London	0.013	0.065	0.21	0.837	-0.114	0.140
Nottingham	0.214	0.060	3.58	0	0.097	0.331
Sheffield	0.066	0.057	1.17	0.241	-0.045	0.178
Dispersion parameter (phi) - using a natural logarithm link function						
Baseline HbA1c (beta scale)	-2.996862	0.9980645	-3	0.003	-4.954	-1.040
Constant	4.912	0.332	14.79	0	4.261	5.563
Hba1c at two years (beta scale)						
Mean effect (Mu) – using a logit link function						
Treatment allocation(1= pumps + DAFNE, 0=MDI + DAFNE)	-0.018	0.035	-0.52	0.603	-0.086	0.050
One year HbA1c (beta scale)	0.797	0.318	2.51	0.012	0.175	1.419
Baseline HbA1c (beta scale)	3.599	0.342	10.51	0	2.927	4.271
Constant	-2.380	0.091	-26.14	0	-2.558	-2.201
Centre effects (Cambridge is the reference site)						
Dumfries and Galloway	0.047	0.093	0.5	0.617	-0.137	0.230
Edinburgh	0.067	0.085	0.8	0.426	-0.098	0.233
Glasgow	0.137	0.097	1.42	0.155	-0.052	0.327
Harrogate	0.123	0.087	1.41	0.158	-0.048	0.294
College Hospital - London	0.079	0.087	0.9	0.366	-0.092	0.249
Nottingham	0.120	0.110	1.09	0.279	-0.098	0.337
Sheffield	0.156	0.080	1.96	0.05	0.000	0.312
Dispersion parameter (phi)) – using a natural logarithm link function						
One year HbA1c (beta scale)	-4.667	1.129	-4.13	0	-6.881	-2.453
Constant	5.422	0.277	19.56	0	4.879	5.966

MDI, multiple daily injections; DAFNE, dose adjustment for normal eating

beta scale – 0 is a HbA1c of 29 mmol/mol and 1 is a HbA1c of 201 mmol/mol

Table 15: The effect of continuous subcutaneous insulin infusion compared to multiple daily injections for all individuals in the per protocol population.

Hba1c at one year (beta scale)	Coefficient	Standard error	t	P>t	95% Confidence interval	
Mean effect (Mu) – using a logit link function						
Treatment allocation (1= pumps + DAFNE, 0=MDI + DAFNE)	-0.056	0.044	-1.37	0.171	-0.148	0.026
Baseline HbA1c (beta scale)	3.938	0.255	13.62	0	2.978	3.980
Constant	-2.219	0.093	-23.94	0	-2.401	-2.038
Centre effects (Cambridge is the reference site)						
Dumfries and Galloway	-0.019	0.078	-0.25	0.805	-0.172	0.134
Edinburgh	0.020	0.056	0.37	0.714	-0.089	0.130
Glasgow	-0.129	0.095	-1.36	0.175	-0.315	0.057
Harrogate	0.025	0.040	0.62	0.534	-0.054	0.104
College Hospital - London	0.018	0.064	0.28	0.779	-0.107	0.143
Nottingham	0.172	0.039	4.46	0	0.096	0.247
Sheffield	0.084	0.064	1.31	0.191	-0.042	0.209
Dispersion parameter (phi) - using a natural logarithm link function						
Baseline HbA1c (beta scale)	-3.504	1.050	-3.34	0.001	-5.563	-1.446
Constant	5.062	0.351	14.41	0	4.373	5.751
Hba1c at two years (beta scale)						
Mean effect (Mu) – using a logit link function						
Treatment allocation (1= pumps + DAFNE, 0=MDI + DAFNE)	-0.047	0.035	-1.35	0.177	-0.116	0.021
One year HbA1c (beta scale)	3.475	0.340	10.23	0	2.809	4.141
Baseline HbA1c (beta scale)	1.053	0.351	3	0.003	0.365	1.740
Constant	-2.382	0.092	-26.01	0	-2.562	-2.203
Centre effects (Cambridge is the reference site)						
Dumfries and Galloway	0.022	0.088	0.26	0.799	-0.150	0.194
Edinburgh	0.076	0.085	0.89	0.374	-0.091	0.243
Glasgow	0.105	0.096	1.1	0.271	-0.082	0.293
Harrogate	0.092	0.085	1.08	0.28	-0.075	0.258
College Hospital - London	0.053	0.085	0.62	0.538	-0.115	0.220
Nottingham	0.109	0.100	1.1	0.276	-0.089	0.308
Sheffield	0.157	0.078	2.02	0.043	0.005	0.310
Dispersion parameter (phi) - using a natural logarithm link function						
One year HbA1c (beta scale)	-4.809	1.231	-3.9	0	-7.223	-2.394
Constant	5.474	0.302	18.13	0	4.882	6.066

pumps, continuous subcutaneous insulin infusion; MDI, multiple daily injections,
Beta scale – 0 is a HbA1c of 29 mmol/mol and 1 is a HbA1c of 201 mmol/mol

Table 16: The variance covariance matrix for the beta regression to predict one year HbA1c in the ITT population

		Mu										Ln Phi	
		Treatment allocation	Baseline HbA1c (Beta scale)	Dumfries and Galloway	Edinburgh	Glasgow	Harrogate	King's College Hospital – London	Nottingham	Sheffield	Constant	Baseline HbA1c (Beta scale)	Constant
Mu	Treatment allocation	0.001											
	Baseline HbA1c (Beta scale)	0.004	0.062										
	Dumfries and Galloway	-0.001	-0.003	0.006									
	Edinburgh	0.000	0.002	0.001	0.004								
	Glasgow	0.001	0.002	0.001	0.001	0.010							
	Harrogate	0.000	0.001	0.001	0.001	0.002	0.002						
	King's College Hospital – London	0.000	0.004	0.001	0.002	0.002	0.001	0.004					
	Nottingham	0.000	-0.004	0.001	0.001	0.001	0.001	0.001	0.004				
	Sheffield	0.000	0.000	0.001	0.001	0.001	0.001	0.001	0.001	0.003			
	Constant	-0.002	-0.019	0.000	-0.002	-0.002	-0.002	-0.003	0.000	-0.001	0.008		
Phi	Baseline HbA1c (Beta scale)	-0.015	-0.110	0.002	-0.010	-0.027	-0.006	-0.021	0.007	-0.007	0.050	0.996	
	Constant	0.005	0.038	0.000	0.003	0.009	0.000	0.004	0.000	0.002	-0.017	-0.308	0.110

Mu – Mean effect (Mu) – using a logit link function; Ln Phi - Dispersion parameter (phi) - using a natural logarithm link function; * - 1 = pumps, 0 = multiple daily injections; Beta scale – 0 is a HbA1c of 29 mmol/mol and 1 is a HbA1c of 201 mmol/mol

Table 17**:** The variance covariance matrix for the beta regression to predict two year HbA1c in the ITT population

		Mu											ln Phi	
		Treatment allocation *	One year HbA1c (beta scale)	Baseline HbA1c (Beta scale)	Dumfries and Galloway	Edinburgh	Glasgow	Harrogate	King's College Hospital - London	Nottingham	Sheffield	Constant	One year HbA1c (beta scale)	Constant
Mus	Treatment allocation*	0.001												
	One year HbA1c (beta scale)	-0.001	0.101											
	Baseline HbA1c (Beta scale)	0.001	-0.071	0.117										
	Dumfries and Galloway	-0.001	-0.004	0.002	0.009									
	Edinburgh	0.000	-0.006	0.003	0.006	0.007								
	Glasgow	0.000	-0.014	0.005	0.006	0.006	0.009							
	Harrogate	0.000	-0.002	-0.002	0.006	0.006	0.006	0.008						
	King's College Hospital - London	0.000	-0.002	0.002	0.006	0.006	0.006	0.006	0.008					
	Nottingham	0.000	-0.006	-0.004	0.006	0.006	0.007	0.006	0.006	0.012				
	Sheffield	0.000	-0.005	0.001	0.006	0.006	0.006	0.006	0.006	0.006	0.006			
	Constant	0.000	-0.004	-0.010	-0.005	-0.005	-0.004	-0.005	-0.006	-0.004	-0.005	0.008		
ln Phi	One year HbA1c (beta scale)	-0.002	-0.014	-0.085	-0.020	-0.006	-0.002	-0.005	-0.010	-0.012	0.000	0.032	1.276	
	Constant	0.000	0.011	0.020	0.005	0.002	0.000	0.003	0.003	0.003	0.000	-0.010	-0.291	0.077

Mu – Mean effect (Mu) – using a logit link function; Ln Phi - Dispersion parameter (phi) - using a natural logarithm link function; * - 1 = pumps, 0 = multiple daily injections; Beta scale – 0 is a HbA1c of 29 mmol/mol and 1 is a HbA1c of 201 mmol/mol

Table 18: The variance covariance matrix for the beta regression to predict one year HbA1c in the per protocol population

		Mu										Ln Phi	
		Treatment allocation	Baseline HbA1c (Beta scale)	Dumfries and Galloway	Edinburgh	Glasgow	Harrogate	King’s College Hospital – London	Nottingham	Sheffield	Constant	Baseline HbA1c (Beta scale)	Constant
Mu	Treatment allocation	0.001											
	Baseline HbA1c (Beta scale)	0.004	0.084										
	Dumfries and Galloway	-0.001	-0.005	0.006									
	Edinburgh	0.000	0.001	0.001	0.003								
	Glasgow	0.000	0.000	0.002	0.002	0.009							
	Harrogate	0.000	0.000	0.001	0.001	0.001	0.002						
	King’s College Hospital – London	0.000	0.004	0.001	0.001	0.002	0.001	0.004					
	Nottingham	0.000	-0.002	0.001	0.001	0.002	0.001	0.001	0.001				
	Sheffield	0.000	-0.003	0.001	0.001	0.002	0.001	0.001	0.001	0.004			
	Constant	-0.002	-0.024	0.000	-0.002	-0.002	-0.001	-0.002	-0.001	-0.001	0.009		
Phi	Baseline HbA1c (Beta scale)	-0.018	-0.117	-0.006	-0.015	-0.031	-0.010	-0.028	-0.017	-0.016	0.060	1.103	
	Constant	0.007	0.040	0.002	0.004	0.010	0.001	0.006	0.005	0.005	-0.020	-0.343	0.123

Mu – Mean effect (Mu) – using a logit link function; Ln Phi - Dispersion parameter (phi) - using a natural logarithm link function; * - 1 = pumps, 0 = multiple daily injections; Beta scale – 0 is a HbA1c of 29 mmol/mol and 1 is a HbA1c of 201 mmol/mol

Table 19: The variance covariance matrix for the beta regression to predict two year HbA1c in the per protocol population

		Mu											ln Phi	
		Treatment allocation *	One year HbA1c (beta scale)	Baseline HbA1c (Beta scale)	Dumfries and Galloway	Edinburgh	Glasgow	Harrogate	King's College Hospital - London	Nottingham	Sheffield	Constant	One year HbA1c (beta scale)	Constant
Mus	Treatment allocation*	0.001												
	One year HbA1c (beta scale)	-0.001	0.123											
	Baseline HbA1c (Beta scale)	0.001	-0.075	0.115										
	Dumfries and Galloway	-0.001	-0.005	0.003	0.008									
	Edinburgh	0.000	-0.008	0.005	0.006	0.007								
	Glasgow	0.000	-0.016	0.007	0.006	0.006	0.009							
	Harrogate	0.000	-0.004	0.000	0.006	0.006	0.006	0.007						
	King's College Hospital - London	0.000	-0.003	0.004	0.005	0.005	0.005	0.005	0.007					
	Nottingham	0.000	-0.009	-0.001	0.005	0.005	0.006	0.006	0.005	0.010				
	Sheffield	0.000	-0.006	0.001	0.005	0.005	0.006	0.005	0.005	0.005	0.006			
	Constant	0.000	-0.007	-0.010	-0.005	-0.005	-0.003	-0.004	-0.005	-0.003	-0.004	0.008		
ln Phi	One year HbA1c (beta scale)	0.008	-0.096	-0.044	-0.017	-0.008	0.000	0.002	-0.009	0.010	0.004	0.038	1.516	
	Constant	-0.003	0.034	0.009	0.004	0.002	-0.002	0.000	0.002	-0.004	-0.002	-0.011	-0.343	0.091

Mu – Mean effect (Mu) – using a logit link function; Ln Phi - Dispersion parameter (phi) - using a natural logarithm link function; * - 1 = pumps, 0 = multiple daily injections; Beta scale – 0 is a HbA1c of 29 mmol/mol and 1 is a HbA1c of 201 mmol/mol

5.3 DKA and severe hypoglycaemia

To develop the method to incorporate severe hypoglycaemic events and DKA treatment effect evidence into the model, several factors were considered. Data on severe hypoglycaemic events and DKA were collected on an ongoing basis throughout the trial. Self-reported information was also collected on the incidence of DKA. A summary of the numbers of DKAs and severe hypoglycaemic events is given in Table 20. It can be seen that the number of DKAs and severe hypoglycaemic events declines in the second year on every measure, except self-reported DKAs in the MDI+DAFNE arm were the number events was the same in both years. As such, the statistical models used in the economic data estimated the incidence of severe hypoglycaemia and DKA in the first and second years separately.

Table 20: A summary of the observed incidence of diabetic ketoacidosis and severe hypoglycaemia in the intention to treat population

	Year 1			Year 2			Total		
	pumps + DAFNE (n=132)	MDI + DAFNE (n=128)	Total (n=260)	pumps + DAFNE (n=132)	MDI + DAFNE (n=128)	Total (n=260)	pumps + DAFNE (n=132)	MDI + DAFNE (n=128)	Total (n=260)
DKAs - Serious Adverse events									
Number(%) participants with ≥1 DKA	15(11.4%)	1(0.8%)	16 (6.2%)	4(3.0%)	2(1.5%)	6(2.2%)	17(12.9%)	3(2.3%%)	20(7.7%)
Number of hospital admissions	16	5	21	5	4	9	21	9	30
DKAs - Self-reported admissions									
Number(%) participants with ≥1 DKA	17 (12.9%)	6(4.7%)	23 (8.8%)	6(4.5%)	5(3.7%)	11(4.1%)	18 (13.6%)	8(6.3%)	26(10.0%)
Number of self-reported DKAs	24	11	35	7	11	18	26*	13*	39*
Severe hypoglycaemia									
Number(%) participants with ≥1 severe hypo	10(7.6%)	9(7.0%)	19(7.3%)	4(3.0%)	7(5.5%)	11(4.2%)	14(10.6%)	11(8.6%)	25(9.6%)
Number of severe hypoglycaemic events	21	12	33	4	12	16	25	24	49

DAFNE, dose adjustment for normal eating; MDI, multiple daily injections; DKA, diabetic ketoacidosis; * these values are not the sums of the one and two year follow up as some individuals had missing information in either the first or second year.

Negative binomial regressions were used to predict the number of DKAs, and severe hypoglycaemic events in years 1 and 2 for each outcome separately. When the outcome variable was the number of severe hypos in year 1, year 1 HbA1c and treatment group were included as covariates. When the outcome variable was the number of severe hypos in year 2, year 2 HbA1c and treatment group were included as covariates When the outcome variable was the number of DKAs in year 1, year 1 HbA1c and treatment group were included as covariates When the outcome variable was the number of DKAs in year 2, year 2 HbA1c and treatment group were included as covariates. The possibility of using the number of events in the previous year, baseline events for the 1 year outcomes and year 1 events for the 2 year outcomes, as a covariate was explored. However, due to the low number of events, the negative binomial models often did not converge when this was included as a covariate.

The statistical models did not converge for DKAs reported as serious adverse events in the first year. This was not the case for self-reported DKAs and there were more self-reported cases of DKA than were picked up through the reporting of serious adverse events. Therefore, the rates of DKA were estimated using self-reported DKAs as the outcome measure.

The statistical models were fitted using the Zellig package in R version3.2.0 and using specifications described above; it was used to simulate the predicted number of severe hypoglycaemia and DKA events in each trial arm 10,000 times. The simulations were separately in each trial arm and for HbA1c values every 0.1% between 4% and 20.5%. The number of events observed in the simulations was truncated at 20 events per year to reduce the effect of extreme values in the simulation on the cost-effectiveness results. These simulations were then used to determine the probability that an individual would suffer a given number of severe hypoglycaemic events and DKA events in a year, dependent on their HbA1c that year and the trial arm they were allocated to. The probability that an individual would suffer a given number of events was a fixed parameter in the PSA, therefore any differences in the rates of DKA or severe hypoglycaemia for an individual between any two model runs will solely be due to differences in their HbA1c.

The results of the negative binomial regressions are given in Table 21 and Table 22.

Table 21: The negative binomial model fitted to the incidence of severe hypoglycaemia at one year and two years

	Coefficient	Standard error	z value	P>z
Severe hypoglycaemia in year 1				
Treatment allocation (1= pumps + DAFNE, 0=MDI + DAFNE)	0.2861	0.5149	0.556	0.578
One year HbA1c (DCCT % scale)	-0.5010	0.2323	2.157	0.03
Number of severe hypoglycaemic events experienced in the year prior to baseline	2.0708	0.5638	3.673	>0.000
Constant	1.2689	1.8676	0.679	0.49687
Severe hypoglycaemia in year 2				
Treatment allocation (1= pumps + DAFNE, 0=MDI + DAFNE)	-1.1141	0.7202	-1.547	0.122
Two year HbA1c (DCCT % scale)	-0.2019	0.2668	-0.757	0.449
Constant	-0.6367	2.2625	-0.281	0.778

DAFNE, dose adjustment for normal eating, MDI, multiple daily injections

Table 22: The negative binomial model fitted to the incidence of diabetic ketoacidosis at one and two years

	Coefficient	Standard error	z value	P>z
Diabetic ketoacidosis in year 1				
Treatment allocation (1= pumps + DAFNE, 0=MDI + DAFNE)	0.3369	0.4786	0.704	0.481
One year HbA1c (DCCT % scale)	0.4089	0.1246	3.283	0.001
Constant	-5.9443	1.1879	-5.004	>0.00
Diabetic ketoacidosis in year 2				
Treatment allocation (1= pumps + DAFNE, 0=MDI + DAFNE)	-0.07564	0.70426	-0.107	0.914
Two year HbA1c (DCCT % scale)	0.32667	0.19447	1.680	0.093
Number of DKAs in year 1	0.86618	0.51682	1.676	0.094
Constant	-5.98206	1.82156	-3.284	0.01

DAFNE, dose adjustment for normal eating, MDI, multiple daily injections; DKA, diabetic ketoacidosis

6 The cost of insulin, diabetes related contacts and insulin pumps used in the Sheffield Type 1 Diabetes model

Self-reported information was collected on individual’s use of insulin, face to face contacts related to their diabetes with a health care professional and telephone contacts related to their diabetes with a health care professional. Data collected on insulin use included, the type of insulin used, the dose of insulin and the method of insulin delivery. Ongoing information was also collected on whether an individual switched their insulin delivery mechanism (either pumps or MDI). The cost of the diabetes related contacts were sourced from the NHS reference costs and were £105.49 for face to face contacts and £75.80 for telephone contacts.[8] Insulin costs were microcosted using the data in the british national formularly and a prescription costs analysis, the full list of unit costs is given in Table 23. [9] [10] The unit costs of the pumps and their related consumables was obtained from a survey of the prices paid at the REPOSE trial sites.

Table 23: The unit costs of insulin

Item	Average unit cost	Number of units	Cost per unit	Associated yearly cost of an insulin pen	Source
Consumables related to multiple daily injections					
Cost of an insulin needle	£0.11	N/A	N/A	N/A	HSCIC[9]
Cost of an insulin syringe	£0.13	N/A	N/A	N/A	HSCIC[9]
Quick Acting Insulin					
Human Insulin					
Vial	£9.87	1000	£0.01	N/A	BNF[10], HSCIC[9]
Cartridges for a reusable pen	£18.97	1500	£0.01	£8.78	
Animal Insulin					
Vial	£26.15	1000	£0.03	N/A	BNF[10], HSCIC[9]
Cartridges for a reusable pen	£38.29	1500	£0.03	£5.97	
Insulin Aspart (NovoRapid)					
Vial	£14.08	1000	£0.01	N/A	BNF[10], HSCIC[9]
Cartridges for a reusable pen	£28.31	1500	£0.02	£9.59	
Disposable Pen	£30.63	1500	£0.02	N/A	
Insulin Lispro (Humalog)					
Vial	£16.61	1000	£0.02	N/A	
Cartridges for a reusable pen	£28.31	1500	£0.02	£8.86	BNF[10], HSCIC[9]
Disposable Pen	£28.31	1500	£0.02	N/A	
Insulin Glulisine (Apidra)					
Vial	£16.00	1000	£0.02	N/A	
Cartridges for a reusable pen	£28.30	1500	£0.02	£7.86	BNF[10], HSCIC[9]
Disposable Pen	£28.30	1500	£0.02	N/A	

Table 23: the unit costs of insulin (continued)

Item	Average unit cost	Number of units	Cost per unit	Associated yearly cost of an insulin pen	Source
	Background Insulin				
Human Insulin					
Vial	£10.41	988	£0.01	N/A	
Cartridges for a reusable pen	£21.52	1500	£0.01	£9.30	BNF[10], HSCIC[9]
Disposable Pen	£21.05	1500	£0.01	N/A	
Animal Insulin					
Vial	£26.17	1000	£0.03	N/A	BNF[10], HSCIC[9]
Cartridges for a reusable pen	£38.32	1500	£0.03	£9.57	
Insulin Detemir (Levemir)					
Cartridges for a reusable pen	£42.00	1500	£0.03	£9.59	BNF[10], HSCIC[9]
Disposable Pen	£42.10	1500	£0.03	N/A	
Insulin Glargine (Lantus)					
Vial	£30.68	1000	£0.03	N/A	
Cartridges for a reusable pen	£41.50	1500	£0.03	£7.86	BNF[10], HSCIC[9]
Disposable Pen	£41.50	1500	£0.03	N/A	
Mixed Insulin					
Biphasic Isophane Insulin					
Animal Insulin					
Vial	£25.20	1000	£0.03	N/A	BNF[10], HSCIC[9]
Cartridges for a reusable pen	£37.80	1500	£0.03	£5.97	
Human Insulin					
Vial	£15.43	987	£0.02	N/A	BNF[10], HSCIC[9]
Cartridges for a reusable pen	£18.94	1500	£0.01	£7.74	
Disposable Pen	£21.43	1500	£0.01	N/A	
Biphasic Insulin Aspart					
Cartridges for a reusable pen	£28.79	£28.79	£0.02	£9.59	BNF[10], HSCIC[9]
Disposable Pen	£29.89	£29.89	£0.02		
Biphasic Insulin Lispro					
Vial	£16.61	1000	£0.02		
Cartridge for reusable pen	£29.03	1500	£0.02	£8.93	BNF[10], HSCIC[9]
Disposable Pen	£30.13	1500	£0.02		

BNF, British National Formulary; HSCIC, Health & Social Care Information Centre

The cost of insulin, diabetes related contacts and insulin pumps (including consumables) for insulin pump therapy individuals were based on resource use data from the REPOSE trial data. It is expected that the covariates which predict the cost of insulin in year 1 may be correlated with the covariates which predict the cost of insulin in year 2. It is also expected that this may be true for the cost of diabetes related contacts and the cost of insulin pumps (including consumables). Therefore, instead of fitting six independent regression models, three seemingly unrelated regressions were fitted (one seemingly unrelated regression for the cost of insulin, another for the cost of diabetes related contacts and finally one for the cost of insulin pumps (including consumables).

In the cost insulin seemingly unrelated regression model, the cost of insulin in year 1 and the cost of insulin in year 2 were used as the outcome variables for the seemingly unrelated regression model. Baseline cost of insulin, baseline HbA1c, treatment allocation, whether the individual switched from multiple daily injections to insulin pump infusion in year one and whether or not the individual switched from insulin pump infusion to multiple daily injections in year 1 were included as covariates to predict the cost of insulin in year 1. Baseline cost of insulin, baseline HbA1c, the actual method of insulin delivery that an individual was using at the end of the first year, whether the individual switched from multiple daily injections to insulin pump infusion in year two and whether or not the individual switched from insulin pump infusion to multiple daily injections in year two were included as covariates to predict the cost of insulin in year two. The standard errors were adjusted for clustering in each DAFNE course.

In the cost of diabetes related contacts seemingly unrelated regression model, the cost of diabetes related contacts in year 1 and the cost of diabetes related contacts in year 2 were used as the outcome variables for the seemingly unrelated regression model. Baseline cost of diabetes related contacts, baseline HbA1c, and treatment allocation; whether the individual switched from multiple daily injections to insulin pump infusion in year one and whether or not the individual switched from insulin pump infusion to multiple daily injections in year 1 were included as covariates to predict the cost of insulin in year 1. Baseline cost of diabetes related contacts, baseline HbA1c, the actual method of insulin delivery that an individual was using at the end of the first year, whether the individual switched from multiple daily injections to insulin pump infusion in year two and whether or not the individual switched from insulin pump infusion to multiple daily injections in year two were included as covariates to predict the cost of insulin in year two. The standard errors were adjusted for clustering in each DAFNE course.

In the cost of insulin pump seemingly unrelated regression model, the cost of insulin pumps and consumables in year 1 and the cost insulin pumps and consumables in year 2 were the two outcome variables used in the model. No control was made for baseline resource use or baseline HbA1c for either outcome variable, as no individual in the REPOSE trial had previous history of using an insulin pump. Individual’s randomised treatment arm, whether or not they switched from pumps to MDI in the first year and whether or not they switched from MDI to pumps in the first year were included as covariates to predict the cost of insulin pumps and consumables in year 1. Individual’s actual treatment at the end of the first year, whether or not they switched from pumps to MDI in year 2 and whether or not they switched from MDI to pumps in year 2 were included as covariates to predict the cost of insulin pumps and consumables in year 2.

The results of the regressions are given in main text (Table 2). The uncertainty in the costs estimated by the seemingly unrelated regressions were included in the model’s PSA using a multivariate normal

distribution. The variance covariance matrices used to parameterise the uncertainty in the cost parameters is given in TablesTable 24 toTable 26.

Table 24: The variance covariance matrix for the seemingly unrelated regression on insulin costs in year 1 and 2 of REPOSE

		Insulin costs year 1					Insulin costs ongoing					
		Baseline insulin cost	Baseline HbA1c (DCCT(%))	Receiving CSII at the start of the year	Switch from CSII to MDI	Constant	Baseline insulin cost	Baseline HbA1c (DCCT(%))	Receiving CSII at the start of the year	Switch from CSII to MDI	Switch from MDI to CSII	Constant
Insulin costs year 1	Baseline insulin cost	0.02										
	Baseline HbA1c (DCCT(%))	0.05	44.17									
	Receiving CSII at the start of the year	0.20	-27.12	653.72								
	Switch from CSII to MDI	3.86	220.49	-696.50	13054.87							
	Constant	-7.41	-404.77	-233.28	-2850.97	6465.05						
Insulin costs ongoing	Baseline insulin cost	0.01	0.17	-0.05	2.47	-6.18	0.01					
	Baseline HbA1c (DCCT(%))	0.10	49.04	-31.68	13.16	-461.41	0.28	75.98				
	Receiving CSII at the start of the year	-0.09	-20.08	668.10	-909.54	-225.18	-0.22	-41.08	913.07			
	Switch from CSII to MDI	0.30	54.23	279.72	-1171.31	-770.00	0.71	70.30	220.46	3131.19		
	Switch from MDI to CSII	0.96	14.50	200.44	285.09	-666.29	1.01	-18.58	327.56	-6.80	6409.60	
	Constant	-5.61	-484.99	-153.96	-391.66	6527.95	-6.52	-744.68	-201.06	-1115.05	-517.86	9117.89

Table 25: The variance covariance matrix for the seemingly unrelated regression on the cost of diabetes related contacts with health care professionals in year 1 and 2 of REPOSE

		Diabetes related contacts (DRC) in year 1						Diabetes related contacts (ongoing)					
		Baseline DRC cost	Baseline HbA1c	Receiving CSII at the start of the year	Switch from CSII to MDI	Switch from MDI to CSII	Constant	Baseline DRC cost	Baseline HbA1c (%)	Receiving CSII at the start of the year	Switch from CSII to MDI	Switch from MDI to CSII	Constant
Diabetes related contacts (DRC) in year 1	Baseline DRC cost	0.00											
	Baseline HbA1c (%)	-0.37	429.26										
	Receiving CSII at the start of the year	0.90	-414.71	4671.82									
	Switch from CSII to MDI	1.79	-3373.14	-6556.03	135705.03								
	Switch from MDI to CSII	8.01	-3893.52	20945.51	-65526.50	401879.74							
	Constant	2.08	-3491.16	1632.08	32468.75	14206.81	30605.56						
Diabetes related contacts (ongoing)	Baseline DRC cost	0.00	-0.10	0.39	-0.34	3.98	0.33	0.00					
	Baseline HbA1c (%)	-0.09	154.18	-31.36	-1626.90	-181.76	-1365.08	-0.07	633.97				
	Receiving CSII at the start of the year	0.04	-111.02	2245.82	-7211.76	14536.08	480.91	0.01	-339.62	4784.66			
	Switch from CSII to MDI	0.08	251.49	294.56	-3310.51	-9569.30	-2251.13	0.17	-570.77	-1568.74	4477.66		
	Switch from MDI to CSII	0.95	-28.09	174.70	-9237.83	3330.56	-448.54	0.87	-926.86	2204.36	2044.24	23540.78	
	Constant	0.67	-1479.09	-339.05	22162.60	-1783.64	13811.74	0.06	-5516.22	1364.46	4819.50	4946.57	50120.60

Table 26: The variance covariance matrix for the seemingly unrelated regression on the cost of insulin pumps and associated consumables in year 1 and 2 of REPOSE

		Insulin pump and consumables costs (year 1)				Insulin pump and consumables costs (ongoing)			
		Receiving CSII at the start of the year	Switch from CSII to MDI	Switch from MDI to CSII	Constant	Receiving CSII at the start of the year	Switch from CSII to MDI	Switch from MDI to CSII	Constant
Insulin pump and consumables costs (year 1)	Receiving CSII at the start of the year	241.41							
	Switch from CSII to MDI	1990.29	82619.62						
	Switch from MDI to CSII	16.58	-627.60	43661.77					
	Constant	1.41E-04	0.01	-5.10E-04	1.40E-08				
Insulin pump and consumables costs (ongoing)	Receiving CSII at the start of the year	200.78	718.30	180.58	-3.30E-04	190.07			
	Switch from CSII to MDI	361.04	-89.20	-528.03	-0.01	535.66	51325.97		
	Switch from MDI to CSII	-3.84	3.83	-4213.99	4.38E-04	-30.22	24.09	23307.42	
	Constant	-4.20E-04	0.01	-1.24E-03	1.53E-08	-8.70E-04	-0.01	1.52E-03	2.02E-08

7 Detailed results of the scenario and subgroup analyses

Table 27: The One way scenario analyses and subgroup analyses performed using the Sheffield Type 1 Diabetes Policy Model

	MDI + DAFNE		pumps + DAFNE		Incremental		
	Total discounted costs	Total discounted QALYs	Total discounted costs	Total discounted QALYs	Total discounted costs	Total discounted QALYs	ICER (£ per QALY gained)
Base Case - PSA	£81,785	12.98	£100,617	13.11	£18,832	0.13	£149,483
Base Case - Deterministic	£73,963	12.81	£93,236	12.98	£19,273	0.18	£109,684
Scenario - Pump prices were estimated from Riemsma et al.[11]	£73,912	12.81	£92,414	12.98	£18,502	0.18	£105,295
Scenario - 25% price reduction in insulin pumps and consumables	£73,513	12.81	£85,939	12.98	£12,426	0.18	£70,715
Scenario - 50% price reduction in insulin pumps and consumables	£73,063	12.81	£78,641	12.98	£5,578	0.18	£31,747
Scenario – Intention to treat estimate of treatment effect	£74,200	12.90	£94,400	12.96	£20,200	0.06	£316,785
Scenario - Intention to treat estimate of treatment effect and no change in HbA1c if an individual switches treatment	£73,308	12.93	£93,496	12.96	£20,188	0.04	£534,397
Scenario – Base case & no change in HbA1c when switching	£73,799	12.86	£93,329	12.95	£19,530	0.09	£207,874
Scenario – Post-trial HbA1c progression in both arms is estimated from the DCCT	£74,278	12.83	£92,865	13.00	£18,586	0.18	£106,126
Scenario - Individuals return to their baseline HbA1c after 3 years and no progression thereafter	£70,053	13.10	£91,063	12.99	£21,011	-0.10	Dominated
Scenario - HbA1c effects occur one model cycle earlier	£75,015	12.78	£93,276	12.92	£18,262	0.14	£130,208
Scenario – individuals return to their baseline risk of hypos and DKA at three years	£73,716	12.86	£95,012	12.83	£21,296	-0.02	Dominated
Scenario - Switching probabilities were estimated directly from the Kaplan - Meier curves	£73,516	12.83	£93,911	12.94	£20,394	0.12	£172,836
Scenario – The utility decrement for blindness was estimated from the Brown *et al*. study.[12]	£73,963	12.80	£93,236	12.97	£19,273	0.18	£110,115

Table 27: The One way scenario analyses and subgroup analyses performed using the Sheffield Type 1 Diabetes Policy Model (continued)

	MDI + DAFNE		pumps + DAFNE		Incremental		
	Total discounted costs	Total discounted QALYs	Total discounted costs	Total discounted QALYs	Total discounted costs	Total discounted QALYs	ICER (£ per QALY gained)
Subgroup - individuals with a baseline HbA1c < 8.5%	£57,947	13.38	£79,001	13.42	£21,054	0.04	£547,504
Subgroup- individuals with a baseline HbA1c ≥ 8.5%	£85,028	12.32	£103,990	12.40	£18,962	0.07	£253,352
Subgroup - individuals with a baseline HbA1c ≥ 7.5%	£76,735	12.58	£95,481	12.74	£18,746	0.16	£120,239
Subgroup - Individuals with a baseline HbA1c ≥ 7.5% & <8.5%	£61,207	13.07	£82,337	13.19	£21,131	0.12	£176,887
Subgroup - Individuals with a baseline HbA1c ≥ 8.5% & <9.5%	£66,520	13.47	£86,105	13.60	£19,584	0.13	£148,240
Subgroup - Individuals with a baseline HbA1c ≥ 9.5%	£99,249	11.64	£115,473	11.81	£16,224	0.17	£96,231
Subgroup - Individuals in the per protocol population	£72,955	12.72	£92,351	12.88	£19,395	0.17	£115,786
Subgroup - Individuals in the per protocol population and no treatment switching	£70,975	12.78	£95,905	12.86	£24,929	0.09	£286,769

MDI - multiple daily injections; DAFNE - dose adjustment for normal eating; pumps; ICER - incremental cost-effectiveness ratio; PSA - probabilistic sensitivity analysis; DCCT - Diabetes Control and Complications Trial

References

1. Office for National Statistics. National Life Tables, United Kingdom : 2012–2014. *Online Source* 2016;Available from: http://www.ons.gov.uk/peoplepopulationandcommunity/birthsdeathsandmarriages/lifeexpectancies/bulletins/nationallifetablesunitedkingdom/previousReleases . (Last Accessed: 17/03/2016)

2. Office for National Statistics. Deaths Registered in England and Wales (Series DR). Table 5 for years 2014, 2013 and 2012. *Online Source* 2015;Available from: http://www.ons.gov.uk/peoplepopulationandcommunity/birthsdeathsandmarriages/deaths/datasets/deathsregisteredinenglandandwalesseriesdrreferencetables(Last Accessed: 17/03/2016)

3. National Records of Scotland. Vital Events Reference Tables. *Online Source* 2015;Available from: http://www.nrscotland.gov.uk/statistics-and-data/statistics/statistics-by-theme/vital-events/general-publications/vital-events-reference-tables/2014(Last Accessed 17/03/2016)

4. Northern Ireland Statistics and Research Agency. Microdata for Deaths in Northern Ireland, 2001-2014. *Online Source* 2014;Available from: http://www.nisra.gov.uk/archive/demography/publications/births_deaths/Pivot_Table_2001_2014.xlsx(Last Accessed 17/03/2016)

5. Heller S, Lawton J, Amiel S, et al. Improving management of type 1 diabetes in the UK: the Dose Adjustment For Normal Eating (DAFNE) programme as a research test-bed. A mixed-method analysis of the barriers to and facilitators of successful diabetes self-management, a health economic analysis, a cluster randomised controlled trial of different models of delivery of an educational intervention and the potential of insulin pumps and additional educator input to improve outcomes. *Programme grants for applied research* 2014;2(5) doi: 10.3310/pgfar02050

6. Wolfe RA, Ashby VB, Milford EL, et al. Comparison of mortality in all patients on dialysis, patients on dialysis awaiting transplantation, and recipients of a first cadaveric transplant. *N Engl J Med* 1999;341(23):1725-30. doi: 10.1056/NEJM199912023412303

7. Briggs A, Claxton K, Sculpher M. Decision Modelling for Health Economic Evaluation. Oxford: Oxford University Press 2006.

8. Department of Health. NHS reference costs 2013 to 2014. *Online Source* 2014;Available from; https://www.gov.uk/government/publications/nhs-reference-costs-2013-to-2014. Last accessed 17th July 2015

9. Health & Social Care Information Centre. Prescription Cost Analysis England 2014. *Online Source* 2015;Available from; http://www.hscic.gov.uk/catalogue/PUB17274. Last Accessed 2nd Novemeber 2015

10. British Medical Association and Royal Pharmaceutical Society of Great Britain. British National Formulary August 2015. *Online Source* 2015;Available from: https://www.medicinescomplete.com, Last Accessed 9th September 2015

11. Riemsma R, Corro Ramos I, Birnie R, et al. Type 1 diabetes: Integrated sensor-augmented pump therapy systems for managing blood glucose levels (The MiniMed Paradigm Veo System and the Vibe and G4 PLATINUM CGM system), a systematic review and economic evaluation. *Kleijnen Systematic Reviews Ltd* 2015

12. Brown MM, Brown GC, Sharma S, et al. Quality of life with visual acuity loss from diabetic retinopathy and age-related macular degeneration. *Arch Ophthalmol* 2002;120(4):481-4.
