# Supplementary material for: Cost-effectiveness of insulin pumps compared with multiple daily injections both provided with structured education for adults with type 1 diabetes: a health economic analysis of the Relative Effectiveness of Pumps over Structured Education (REPOSE) randomised controlled trial
Source: BMJ Open. 2018 Apr 7;8(4):e016766. doi: 10.1136/bmjopen-2017-016766 (PMC5893943; doi:10.1136/bmjopen-2017-016766)
Supplement: Author's manuscript [file bmjopen-2017-016766.draft_revisions.pdf]

BMJ Open

BMJ Open is committed to open peer review. As part of this commitment we make the peer review history of every article we publish publicly available.

When an article is published we post the peer reviewers' comments and the authors' responses online. We also post the versions of the paper that were used during peer review. These are the versions that the peer review comments apply to.

The versions of the paper that follow are the versions that were submitted during the peer review process. They are not the versions of record or the final published versions. They should not be cited or distributed as the published version of this manuscript.

BMJ Open is an open access journal and the full, final, typeset and author-corrected version of record of the manuscript is available on our site with no access controls, subscription charges or pay-per-view fees (http://bmjopen.bmj.com).

If you have any questions on BMJ Open's open peer review process please email editorial.bmjopen@bmj.com

BMJ Open

The cost-effectiveness of insulin pumps compared to multiple daily injections, both provided with structured education, for adults with type 1 diabetes: a health economic analysis of the relative effectiveness of pumps over structured education (REPOSE) randomised controlled trial.

Journal:	*BMJ Open*
Manuscript ID	bmjopen-2017-016766
Article Type:	Research
Date Submitted by the Author:	08-Mar-2017

Complete List of Authors:	Pollard, Daniel ; University of Sheffield, School of Health and Related Research<br>Brennan, Alan; University of Sheffield, School of Health & Related Research<br>Dixon, Simon; University of Sheffield, School of Health and Related Research<br>Waugh, Norman; University of Warwick, Warwick Evidence<br>Elliott, Jackie; University of Sheffield, Academic Unit of Diabetes, Endocrinology and Metabolism<br>Heller, Simon; Unversity of Sheffield, Academic Unit of Diabetes, Endo and Metab<br>Lee, Ellen; University of Sheffield, Clinical Trials Research Unit<br>Campbell, Michael; University of Sheffield, Health Services Research ScHARR<br>Basarir, Hasan; University of Birmingham,<br>White, David; University of Sheffield, Clinical Trials Research Unit

Keywords:	HEALTH ECONOMICS, DIABETES & ENDOCRINOLOGY, General diabetes < DIABETES & ENDOCRINOLOGY

SCHOLARONE™
Manuscripts

Title: The cost-effectiveness of insulin pumps compared to multiple daily injections, both provided with structured education, for adults with type 1 diabetes: a health economic analysis of the relative effectiveness of pumps over structured education (REPOSE) randomised controlled trial.

Running head: Cost-effectiveness of insulin pumps with structured education for adults with type 1 diabetes

Authors:

Daniel Pollard, Research Associate, School of Health and Related Research (ScHARR), University of Sheffield, Sheffield, UK

Alan Brennan, Professor of Health Economics and Decision Modelling, School of Health and Related Research (ScHARR), University of Sheffield, Sheffield, UK

Simon Dixon, Professor of Health Economics, School of Health and Related Research (ScHARR), University of Sheffield, Sheffield, UK

Norman Waugh, Professor in Public Health, Warwick Medical School – Population Evidence and Technologies, University of Warwick, Coventry, UK

Jackie Elliott, Senior Clinical Lecturer in Diabetes, Academic Unit of Diabetes, Endocrinology and Metabolism, Department of Oncology and Metabolism, School of Medicine and Biomedical Sciences, University of Sheffield, UK

Simon Heller, Professor of Clinical Diabetes, Academic Unit of Diabetes, Endocrinology and Metabolism, Department of Oncology and Metabolism, School of Medicine and Biomedical Sciences, University of Sheffield, UK

Ellen Lee, Research Associate, Clinical Trials Research Unit, School of Health and Related Research (ScHARR), University of Sheffield, Sheffield, UK

Michael Campbell, Professor of Medical Statistics, School of Health and Related Research (ScHARR), University of Sheffield, Sheffield, UK

Hasan Basarir, Research Fellow in Health Economics, Health Economics Unit, Institute of Applied Health Research, College of Medical and Dental Sciences, University of Birmingham, UK

David White, Trial manager, Clinical Trials Research Unit, School of Health and Related Research (ScHARR), University of Sheffield, Sheffield, UK

On behalf of the REPOSE group

Correspondence to Daniel Pollard. Email: d.j.pollard@sheffield.ac.uk. Telephone: +44 (0) 114 222 6126

Abstract: 299 words

Main text: 4,593 words

For peer review only - http://bmjopen.bmj.com/site/about/guidelines.xhtml

Abstract

Objectives

To assess the long term cost-effectiveness of dose adjustment for normal eating and insulin pumps (pumps+DAFNE) compared to dose adjustment for normal eating and multiple daily insulin injections (MDI+DAFNE) for adults with type 1 diabetes mellitus (T1DM) in the UK.

Methods

We undertook a cost-utility analysis using the Sheffield Type 1 Diabetes Policy Model and data from the REPOSE trial to estimate the lifetime incidence of diabetic complications, intervention-based resource use and associated effects on costs and quality adjusted life years (QALYs). All economic analyses took an NHS and personal social services perspective and discounted costs and quality adjusted life years at 3.5% per annum. A probabilistic sensitivity analysis was performed in the base case. Further uncertainties in the cost of pumps and the evidence used to inform the model were explored using scenario analyses.

Setting

Eight diabetes centres in England and Scotland

Participants

Adults with T1DM who were eligible to receive a structured education course, did not have a strong clinical indication for a pump and did not have a preference to receive a pump.

Intervention

pumps +DAFNE

Comparator

MDI+DAFNE

Main outcome measures

Incremental costs, incremental QALYs gained and incremental cost-effectiveness ratios (ICERs).

Results

Compared to MDI+DAFNE, pumps+DAFNE was associated with an incremental discounted lifetime cost of +£18,853 and a gain in discounted lifetime quality adjusted life years of +0.1325. The base case mean ICER was £142,195 per QALY gained. The probability of pump+DAFNE being cost-effective using a cost-effectiveness threshold of £20,000 per QALY gained was 14.0%. All scenario and subgroup analyses examined indicated that the ICER was unlikely to fall below £30,000 per QALY gained.

Conclusions

For peer review only - http://bmjopen.bmj.com/site/about/guidelines.xhtml

Our analysis of the REPOSE data suggests that routine use of pumps in adults without an immediate clinical need for a pump, as identified by NICE, would not be cost-effective.

Strengths and limitations of this paper

This study is the first cost-effectiveness analysis to consider the use of insulin pumps for adults with type 1 diabetes who are eligible to receive to structured education course but not an insulin pump using current UK guidance.

The REPOSE trial was the first trial to address the question of whether insulins pumps were a clinically effective treatment option in this patient population.

An existing validated model of type 1 diabetes and its complications, developed during a previous National Institute for Health Research funded programme grant, was adapted and updated with evidence from the REPOSE trial and more recent evidence on clinical effectiveness, utilities and costs

We considered a UK health care perspective to estimate the key drivers of decision uncertainty.

The main limitations of the study are that the only available evidence in long term trends in HbA1c (the key clinical outcome) was from several observational studies, the analysis of diabetic ketoacidosis was based on self-reported information rather than data from inpatient admissions and that the trial only included one type of insulin pump.

For peer review only

For peer review only - http://bmjopen.bmj.com/site/about/guidelines.xhtml

For peer review only

INTRODUCTION

People with type 1 diabetes mellitus (T1DM) are unable to produce insulin due to autoimmune destruction of their insulin secreting beta cells. Those affected have to inject insulin to prevent diabetic ketoacidosis in the short-term, and in the long term, to prevent microvascular and macrovascular disease. Insulin is generally administered by intermittent subcutaneous injection with the dose adjusted according to eating, physical activity and current blood glucose levels. Therapy is designed to keep blood glucose as close to normal as possible, to prevent both microvascular complications and to reduce the risk of macrovascular disease.[1] A further aim of treatment is to achieve as good a quality of life (QoL) as possible, particularly since self-management of the condition is challenging and arduous, demanding the implementation of complex skills.

Historically, insulin was given twice a day, often as pre-mixed insulin, but such an approach imposes a rigid lifestyle on patients and makes it difficult to maintain blood glucose levels close to normal. Most individuals require intensive insulin therapy to maintain tight glycaemic control. This approach and its integration within a flexible lifestyle is promoted in structured training courses, such as the Dose Adjustment For Normal Eating (DAFNE)[2] course and others.[3] The principles of this multiple daily injection (MDI) via subcutaneous injection approach involves the use of quick-acting insulin injected before eating (with the dose calculated according to the amount of carbohydrate eaten) combined with long-acting background 'basal' insulin, usually given twice daily, to control blood glucose in between meals.

As an alternative to MDI, insulin can be administered by an insulin pump system in which insulin is delivered throughout the day using a small, portable pump, connected by a flexible plastic tube to a subcutaneous cannula. This technology is relatively expensive compared to MDI. Current National Institute for Health and Care Excellence (NICE) guidelines recommend that all adults with T1DM in the UK receive a structured education course of proven benefit at a clinically appropriate time.[4] Insulin pumps are a treatment option for adults with T1DM who either have a HbA1c above 69 mmol/mol (8.5%) or experience disabling hypoglycaemia.[5] Insulin pumps do not replace the need for education and it is currently recommended that specialist teams should provide structured education programmes and advice on diet, lifestyle and exercise for people using an insulin pump.[5]

Use of pumps in T1DM varies substantially between countries. In England, 11.7% of people with T1DM are estimated to use a pump which compares to around 40% in the USA.[6 7] It has been proposed that insulin pumps are underutilised in the UK and that the glycaemic control of adults with T1DM could be improved if pumps were used more widely.[8] Two NICE appraisals found that there was insufficient evidence on insulin pump therapy compared to MDI in which adults with T1DM used both long and short acting insulins.[9 10] Furthermore, a recent observational study of pumps and MDI suggested that much of the previous benefit attributed to pumps may be due to the additional education that pumps users require when initiating therapy.[11] Therefore, uncertainty remains as to the clinical benefit and cost-effectiveness of pumps as a treatment option for adults with T1DM.

The relative effectiveness of pumps over structured education (REPOSE) trial was conducted to assess if pumps offered any additional benefit compared to MDI for the treatment of adults with

For peer review only - http://bmjopen.bmj.com/site/about/guidelines.xhtml

T1DM in the UK who are eligible to attend a structured education course, but do not have an immediate clinical need for a pump. Participants in both trial arms received a DAFNE course, which taught MDI users to use long and short acting insulins appropriately. In this paper we present the cost-effectiveness analysis of pumps+DAFNE compared to MDI+DAFNE for adults in the UK with T1DM who are eligible to receive a structured education course, but did not have a clinical need for the immediate commencement of pump therapy.

METHODS

Economic evaluation methods

The health economic analysis followed the pre-specified health economic analysis plan, which was outlined in the trial protocol paper.[12] Two approaches were undertaken to assess the cost-effectiveness of pumps: an economic evaluation alongside the clinical trial (EEACT); and, a long term economic model to assess the lifetime outcomes. In the EEACT, data on the costs and QALYs were obtained from data collected in the REPOSE trial, whereas the long term model estimated the lifetime costs and QALYs based mainly upon the biomedical outcomes collected in the REPOSE trial. All modelling analyses took a lifetime time horizon and all EEACT analyses took a two-year (trial duration) time horizon. The pre-specified primary analysis relates to the life time modelling, and as such, are reported in this paper The methods and results of the EEACT are reported in supplementary material A. In line with NICE guidance, all economic analyses took an NHS and personal social services perspective and costs and QALY outcomes were discounted at 3.5%.[13]

The economic model

The Sheffield Type 1 Diabetes Policy Model version 1.3.2, henceforth 'the model', is an individual level simulation model used to estimate the lifetime costs and QALYs associated with pump+DAFNE and MDI+DAFNE. The most recently published version of the model[14] was updated using data collected in the REPOSE trial at 12-month and 24-month follow-up. Specifically, the level of HbA1c, risk of severe hypoglycaemia, risk of diabetic ketoacidosis (DKA) and the probability that an individual would switch insulin delivery mechanism and costs-related to the intervention (insulin and MDI consumables, diabetes related contacts with health care professionals, insulin pumps and the associated consumables) were updated. Relevant literature also informed the changes in HbA1c beyond the two-year trial duration and the probability of death from end stage renal disease (see supplementary material B, page 2) in both arms. The model examines disease progression over a lifetime using an annual time cycle. An individual's HbA1c determines their risk of progression for all diabetic complications in the model, which include: nephropathy, neuropathy, retinopathy, macular oedema, myocardial infarction, stroke, heart failure, angina and severe hypoglycaemia and DKA. The model attaches utilities to health states and costs to events, allowing the calculation of costs and QALYs over a lifetime. Full details of how the model calculates the incidence of diabetes complications is provided in Heller *et al* (pages 104-106) and Thokala *et al*.[14 15] The incremental cost-effectiveness ratio (ICER) calculated was compared to the £20,000 to £30,000 per QALY gained threshold used by NICE.[13] In each model run, the life course of 5,000 individuals was simulated. All model analyses were conducted using SIMUL8 professional 2010.

The clinical data

For peer review only - http://bmjopen.bmj.com/site/about/guidelines.xhtml

The details of the methods used in the REPOSE trial have been reported elsewhere.[12] The eligibility criteria of the trial included adults with T1DM if they were eligible to receive a structured education course, did not have a clinical indication to receive a pump immediately, as determined by the investigator, or did not have a strong preference to receive a pump.[12] The REPOSE trial was conducted in eight centres in England and Scotland, involving 267 individuals. Out of these 267 individuals, 260 had HbA1c data for at least one post-baseline follow up visit (ITT population) and 236 adhered to their randomised treatment (per protocol population). Self-reported participant information, which includes the number of DKA events, EQ-5D-3L, SF-12 and resource use were collected at baseline, 6 months, 1 year and 2 years after attending DAFNE training. HbA1c, the primary endpoint, was measured at baseline and at these follow-up appointments. All inpatient admissions, the incidence of severe hypoglycaemia and whether an individual switched insulin delivery method were collected on an ongoing basis. Information was collected on the staff time spent delivering pre-course fitting sessions for individuals allocated to receive a pump. The cost of insulin pumps and associated consumables in routine practice, and the insulin pump consumables used by the REPOSE participants, were collected from seven of the eight trial centres.

The simulation cohort

To obtain the simulation cohort, participants REPOSE data were sampled with replacement 5,000 times.[16] This created a simulation cohort in which individuals with missing characteristics in their REPOSE data were replicates multiple times. Each individual's REPOSE data was included in the simulation cohort a median of 19 times (interquartile range 16 – 22). In the simulation cohort, a single imputation was performed in STATA version 13.1. A chained truncated regression procedure for missing continuous variables, in which variables were limited to positive values, and a Poisson procedure for missing categorical variables. In both sets of imputation models, all characteristics with complete data in the REPOSE trial population were used as predictive covariates. Identical individuals were simulated in both model arms. A summary of the baseline characteristics and completeness of data for the 260 REPOSE participants in the ITT population and the 5,000 individuals in the simulation cohort are given in Table 1.

<Table 1>

The clinical effectiveness data

Statistical analyses of the REPOSE trial data were conducted to estimate treatment switching, HbA1c, severe hypoglycaemia and DKA. Information on long term changes in HbA1c was obtained from the available literature and the REPOSE trial data. Unless otherwise stated, all statistical analyses were conducted using STATA v13.1. Full details on the statistical methods used, the results and how the analyses were incorporated into the model are provided in supplementary material B.

To estimate the incidence of treatment switching in the first and second year of the model, a time to event analysis was conducted using treatment switching as the event of interest. Kaplan-Meier curves were plotted and parametric survival curves were fitted to the Kaplan-Meier curves.[17 18] Separate parametric survival models were fitted to individuals randomised to pump+DAFNE and MDI+DAFNE. The goodness of fit of the parametric survival curves was assessed using the Akaike information criterion (AIC), Bayesian information criterion (BIC) and visual assessment of the fit of the parametric curves to the Kaplan-Meier curves at one and two years. As treatment switching was

For peer review only - http://bmjopen.bmj.com/site/about/guidelines.xhtml

included in the model, it is important to adjust the modelled treatment effectiveness and cost of treatment for those individuals who switch. If individuals switched treatment, then their HbA1c was assumed to change as though they had been allocated to the other arm. In the base case analysis, the HbA1c effect of pump+DAFNE compared to MDI+DAFNE was estimated in the per protocol population, as the people who switched treatment were excluded from this population.

To estimate each individual's HbA1c in the model, a beta regression was fitted to the HbA1c data collected in REPOSE for all individuals in the per protocol population at the one and two-year follow-up.[19]Bounds were placed on the beta regression so that HbA1c was between the clinically plausible bounds of 29mmol/mol [4.8%] and 201 mmol/mol [20.5%], which were provided by two clinical experts in the REPOSE trial management group. Missing data was observed for HbA1c values in the per protocol population at 6 months (2.1% missing), 1 year (4.2% missing) and 2 years (4.2% missing). Full details on the imputation procedure used to account for the missing data and the specification of the beta regression are provided in supplementary material B (pg8). The effects in a beta regression are not easily interpretable by themselves, however information can be obtained on the direction of effect.

The expert opinion of clinical members involved in the literature review of clinical studies for the REPOSE trial was sought to identify studies with long term data on HbA1c for the type of pumps used in the trial and people who used MDI after DAFNE. Three articles on the long term trends in HbA1c for pump users[20-22] and two articles on the long term trends in HbA1c for MDI users post-DAFNE[23 24] were used to estimate yearly changes in HbA1c for each model arm. The average annual increase in HbA1c was calculated for each study by calculating yearly increase in HbA1c between the lowest observed HbA1c value and the last follow up point in the study where the sample size was greater than one quarter of the initial sample size. A standard error could not be directly calculated, as we did not have access to the patient level data from the studies. Instead, data on the standard deviation of the change in HbA1c between the 1 and 2 year follow ups specific to each REPOSE trial arm was used to inform the uncertainty in this parameter. These standard deviations were divided by the square root of the combined sample size of the studies used to inform the mean effect to estimate standard error for this parameter..

Negative binomial regressions were used to estimate the risk of severe hypoglycaemia and DKA in the ITT population of REPOSE using the Zellig package in R version 3.2.0. Negative binomial regressions were fitted separately to severe hypoglycaemia and DKA events and to the first and second year of follow up data. The regressions fitted to the second year data were used to estimate the incidence of severe hypoglycaemia and DKA in all model time cycles, except for the first time cycle. This assumption was based on clinical expert opinion, which was confirmed by the trial management group, that the first 6 months of using a pump was associated with a learning period and after this time the incidence of severe hypoglycaemia and DKA would decrease.

Costs and utilities

The costs and health state utility values used for each health state in the model are provided in Table 2 and Table 3 respectively. All costs were reported in 2013/14 prices. Costs sourced from evidence in previous years were inflated to 2013/14 prices using the hospital and community health services pay and prices index.[25] The cost of insulin and MDI consumables used, cost of diabetes related contacts with healthcare professionals and cost of the pump intervention costs were estimated

For peer review only - http://bmjopen.bmj.com/site/about/guidelines.xhtml

separately for both year 1 and year 2 of the REPOSE trial. The unit cost of insulin and MDI consumables used was obtained from the British National Formulary and Health & Social Care Information Centre, the unit cost of diabetes related contacts were obtained from NHS reference costs and unit cost of an insulin pump (with its associated consumables) was obtained from a survey of the REPOSE trial sites.[26-28] Table 2 part A shows the 6 regression formulae which estimate an individual's annual cost for each of these components as a function of:- their baseline HbA1c, which treatment they start the year on (MDI or pump), whether they switch treatment, and also the participants' self-reported use of insulin and level of contact with healthcare professionals prior to recruitment to the trial. Details on how these parameters were used to estimate the costs in the model are provided in Table 2.

<Table 2 and 3>

Scenario and subgroup analyses

For the base case, a probabilistic sensitivity analysis was conducted where each parameter was sampled from its probability distribution, the results were recorded, the process was repeated 500 times and the averages from these 500 model runs were reported. All statistical models, except for the risks of severe hypoglycaemia and DKA, for which the outcomes were directly simulated in R version 3.2.0, were included in the model using a multivariate normal distribution. The long term changes in HbA1c were assumed to be distributed using independent normal distributions for each model arm. Deterministic scenario analyses were undertaken to assess the robustness of the results. The key scenario analyses for the model include: uncertainty in the estimates of treatment effectiveness, the timing of HbA1c changes in the model, the utility decrement for blindness (-0.26) [29] and the cost of insulin pumps and insulin pump consumables.

A two-way determinsitic threshold analysis was conducted to assess the HbA1c reduction and/or annual cost reduction necessary to potentially make pumps cost-effective in the UK. All threshold analysis runs were conducted deterministically. In this analysis, each individual's HbA1c was estimated as though they received MDI. Then those individuals in the pump arm received a fixed change in HbA1c. This change in HbA1c was varied at 10 different values between-3.3mmol/mol (-0.3%) and-13.1mmol/mol (-1.2%), and annual cost of insulin pumps were also varied between 100% of the observed cost in the REPOSE trial and 50% of the observed cost.

Deterministic subgroup analyses were conducted in the following populations:

1. Baseline HbA1c < 69 mmol/mol (8.5%)
2. Baseline HbA1c ≥ 69 mmol/mol (8.5%)
3. Baseline HbA1c ≥ 58 mmol/mol (7.5%)
4. 69 mmol/mol (8.5%) >baseline HbA1c ≥ 58 mmol/mol (7.5%)
5. 80 mmol/mol (9.5%) > baseline HbA1c ≥ 69 mmol/mol (8.5%)
6. Baseline HbA1c ≥ 80 mmol/mol (9.5%)
7. All individuals in the per protocol population

For peer review only - http://bmjopen.bmj.com/site/about/guidelines.xhtml

For peer review only

The subgroup analyses were conducted by resampling the simulation cohort from the individuals who met each of these criteria in the ITT population.

RESULTS

Clinical effectiveness data

The analysis of treatment switching in the REPOSE trial suggested that an exponential curve provided the best fit to the data for individuals receiving pump+DAFNE and a Weibull curve provided the best fit for individuals in the MDI+DAFNE arm. The results of the analysis of HbA1c data suggested that pump+DAFNE compared to MDI+DAFNE was associated with statistically insignificant HbA1c reductions in both year 1 and in year 2. The analysis of the literature for long-term trends in HbA1c suggested that weighted average annual progression of HbA1c was +0.568mmol/mol (+0.052%) per annum in the pump+DAFNE arm and +0.590 mmol/mol (+0.054%) per annum in the MDI+DAFNE arm. The estimated standard errors for the long-term trends in HbA1c were of 0.627 mmol/mol (0.040%) for MDI+DAFNE and 0.627 mmol/mol (0.057%) for pump+DAFNE. The results of the negative binomial models used to estimate the incidence of DKA indicated that pump+DAFNE compared to MDI+DAFNE was associated with more DKA events in the first year (incidence rate ratio (IRR) 1.40, 95% confidence interval (CI) 0.55 to 3.58), but fewer events in year 2 (IRR 0.93, 95% CI 0.23 to 3.69). The results of the negative binomial models used to estimate the incidence of severe hypoglycaemia indicated that pump+DAFNE compared to MDI+DAFNE was associated with more severe hypoglycaemic events in year 1 (IRR 1.33, 95% CI 0.49 to 3.65) but fewer events in the second year (IRR 0.35, 95% CI 0.08 to 1.44).

Base case results

Table 4 shows the base case results for the long-term cost-effectiveness analysis using the probabilistic sensitivity analysis. For the pump+DAFNE arm, the mean costs of the intervention are £42,124 discounted over the lifetime horizon, which compares with £19,829 for the MDI+DAFNE arm. This leads to an increase in intervention costs of £21,295 over a person's lifetime. The QALYs lived, prior to applying the utility decrements associated with diabetic complications, in the pump+DAFNE arm is 13.9145 QALYs compared to 14.0292 QALYs in the MDI+DAFNE arm, a difference of 0.1147 QALYs. The differential incidence of adverse events in the pump+DAFNE arm compared to the MDI+DAFNE arm leads to a lower costs (-£371) and higher QALYs (+0.0027) per person. The reduced incidence of diabetic complications in the pump+DAFNE arm leads to lower costs (-£2,070) and more QALYs (+0.0152) per person. The net incremental lifetime cost of pump versus MDI is therefore estimated as £18,853 (95%CI £6,175 to £31,645) per person. The net incremental QALY gain per person is 0.1326 (95%CI -0.7087 to 0.9623) QALYs per person. The ICER associated with pump+DAFNE was £142,195 per QALY gained. This is considerable above the £30,000 per QALY threshold, which is the higher limit at which NICE would usually consider interventions to be cost-effective.[13] Figure 1a shows that this result is subject to a small degree of uncertainty, as in 14.0% of the PSA runs, pump+DAFNE would be considered as being cost-effective using the lower end of the range used by NICE.

<Table 4>

For peer review only - http://bmjopen.bmj.com/site/about/guidelines.xhtml

Scenario and subgroup analyses

The results of 11 scenario analyses are presented in Figure 1b. None of the scenario analyses showed pump+DAFNE to be cost-effective versus MDI+DAFNE. The lowest ICER found (£31,747 per QALY gained) was for scenario 3 in which yearly cost of pumps and consumables was halved.

<Figure 1>

The results of the two-way price and effectiveness threshold analysis are given in Table 5. When the annual pump cost is assumed to be 100% of the prices observed in REPOSE, then the analysis shows that the reduction in HbA1c (for pump compared to MDI) would need to be 12.0 mmol/mol (1.1%) or more, for pump to have an ICER below £20,000 per QALY gained. When the annual cost is 25% lower, then a HbA1c reduction of more than 7.7 mmol/mol (0.7%) would be needed to have an ICER below £20,000 per QALY gained. When the annual cost is halved, then a HbA1c reduction of 3.3 mmol/mol (0.3%) would be sufficient to have an ICER below £20,000 per QALY gained.

<Table 5>

The result of the subgroup analyses are presented in Figure 1c. Similarly to the scenario analysis, no subgroup was identified in which pump+DAFNE would be likely to be considered more cost-effective than MDI+DAFNE. The most cost-effective subgroup was those individuals with a baseline HbA1c greater than or equal to 80 mmol/mol (9.5%), with an ICER of £96,394 per QALY gained versus MDI+DAFNE.

DISCUSSION

This is the first study to examine the marginal benefits and cost-effectiveness of insulin delivered using pumps over MDI when both groups have had structured education, in patients without an immediate clinical need for an insulin pump, as recommended by NICE. Our findings show that providing pumps for this wider group of adults with T1DM would be unlikely to be considered cost-effective in the UK because the estimated incremental cost-effectiveness ratio was just over £142,000, substantially higher than the £30,000 per QALY upper limit often used by UK decision makers. This finding is also valid in all scenario and subgroup analyses examined. The threshold analysis indicates that if new insulin pump technology is developed and the costs are similar to current insulin pumps, a trial would have to demonstrate a HbA1c reduction in the region of 11 mmol/mol (1.0%) to 13 mmol/mol (1.2%) compared to MDI+DAFNE for the new technology to be considered cost-effective in the UK for the population analysed in this study. Conversely, if insulin pump prices were halved, then reductions of only 3 mmol/mol compared to MDI are required.

The key strengths of this study are that it is based on a rigorously conducted cluster RCT with economic data directly collected during the study. Data completeness for the primary outcome, was very high at 95%. The study uses an individual level simulation model of type 1 diabetes disease progression over a lifetime horizon. The study does have limitations in terms of the evidence used to inform the long term changes in HbA1c, which were based on 5 observational studies with follow up ranging from 3.7 to 10 years, rather than trials with long follow up periods. Also, as REPOSE is the first study to assess the effectiveness of pumps+DAFNE, the long term evidence was based on observational studies of pumps in which the education component is likely to have been different.

For peer review only - http://bmjopen.bmj.com/site/about/guidelines.xhtml

Our probabilistic sensitivity analysis has incorporated uncertainty around these estimates. Secondly, the analysis of rates of DKA was based on self-reported occurrence of DKA in REPOSE (the same method as used in other recent trials of DAFNE Heller *et al* Chapter 8[14]), rather than corroborated data on inpatient admissions which had a smaller number of events and could not be analysed statistically. Thirdly, only one pump type was assessed in the trial, and some caution may be needed when considering extrapolating results to modern pumps with auto-suspend features. Finally, the results of the threshold analysis used to determine what the effectiveness of pumps would need to be to be considered cost-effective, should be interpreted with some degree of caution for two reasons. Firstly, we used a fixed HbA1c effect for every individual, rather than a method which accounts for heterogeneous treatment responses and secondly we assume switchers from MDI to pump get the fixed HbA1c reduction and switchers from pump to MDI get the same fixed HbA1c increase. In an ITT analysis were switchers are included in their originally randomised arms, smaller HbA1c reductions than the ones in this analysis, would likely lead to the same ICERs.

A 2015 systematic review on the cost-effectiveness of insulin in different countries identified four studies from the UK, three of which presented an ICER.[30] The base case ICERs in these three studies were £11,461, £25,648 and £37,712, which indicate that in two out of the three studies that pumps had an ICER within/below the threshold range usually used by NICE to determine the cost-effectiveness of technologies in the UK. However, these studies most of the previous cost-effectiveness analyses used a reduction in Hba1c of 10-13 mmol/mol (0.95 – 1.2%) associated with pumps compared to MDI, based on the meta-analysis by Weissberg-Benchell *et al.* [31], which included a mixture of RCTs and observational studies. This effect size is much larger than that observed in the REPOSE trial. Some of the effect size in the meta-analysis may be related to the education offered to pump participants which was not offered to MDI participants and that MDI was not administered using both long and short acting analogue insulins in most studies included in the meta analysis. To our knowledge REPOSE is the first large trial in adults with Type 1 diabetes that provides evidence on pumps versus MDI (in which both long and short acting analogue insulins are used) that provides equivalent diabetes education to both trial arms. As such, this economic analysis is the first cost-effectiveness analysis relevant to assessing whether pumps should be offered at the point when adults with T1DM in the UK are eligible to receive structured education and do not have an immediate clinical need to receive a pump.

The key implication of this paper is that unless there is an immediate clinical need for using a pump, clinicians in the NHS should offer adults with T1DM a structured education course of proven benefit, prior to considering pump therapy. This is because, when pumps+DAFNE is compared to MDI+DAFNE, the incremental health benefits are relatively small and the incremental lifetime costs are relatively high. This indicates that it would not be cost-effective to allow all adults with T1DM who are eligible for structured education, and have no clinical need for a pump, to also immediately receive an insulin pump.

A question not addressed by the REPOSE trial is whether pumps would be clinically effective and cost-effective in patients offered the technology at some point after having attended a structured education course. Generating this evidence would require a clinical study, which recruited participants who had previously received a structured education course of proven benefit and randomising them to either continued MDI or pumps. Whilst the current literature does provide some indication of which individuals may benefit from pumps, the clinical evidence informing these

For peer review only - http://bmjopen.bmj.com/site/about/guidelines.xhtml

studies was not limited to those individuals who had first received structured education. This is important because the effectiveness of insulin pumps would be expected to differ in this group, with this being related to observed and unobserved patient characteristics. One particular hypothesis that we believe is worthwhile investigating is whether pumps should be used in the UK by those adults with T1DM who actively self-manage after attending a structured education course, but either have not achieved the NICE target HbA1c levels of less than or equal to 48mmol/mol (6.5%) or who have problematic hypoglycaemia. Another important research question concerns the effectiveness and cost-effectiveness of support programmes post-education, so that more adults with T1DM achieve glycaemic targets. The DAFNEplus NIHR programme grant, which will report in 2022, is developing and testing an adapted DAFNE course (based on previous research, behaviour change theory and technological support) and subsequent structured support to improve self-management and glucose control.

In conclusion, the results indicate that it would not be cost-effective to offer pumps to all adults with T1DM in the UK, who are currently eligible to receive a structured education course and do not have an immediate clinical need for a pump in the UK. Use of MDI+DAFNE is estimated to represent a better use of NHS resources than immediate commencement on a pump. Further research is required to improve the glycaemic control of adults with T1DM in the UK.

For peer review only

Funding

This research was funded by the UK Health Technology Assessment Programme (project number 08/107/01). We also acknowledge the financial support of the Research and Development Programmes of the Department of Health for England and the Scottish Health and Social Care Directorates who supported the costs of consumables and of Medtronic UK Ltd., who provided the insulin pumps for the trial. The funders had no involvement in the design of the protocol, the collection, analysis and interpretation of the data, in the writing of this paper, or in the decision to submit this article for publication.

Conflicts of Interest

SH reports personal fees from Sanofi Aventis and personal fees and other from NovoNordisk and Eli Lilly, outside the submitted work. JE reports personal fees from Astra Zeneca, Merck Sharpe Dohme and Takeda, personal fees and non-financial support from Eli Lilly, Novonordisk and Sanofi, outside the submitted work. NW reports receiving two days of consultancy fees from Novo Nordisk on topics including alternatives to network meta-analysis, unrelated to REPOSE. No other authors have any conflicts of interest to report.

For peer review only

Contributions

Author contributions

Daniel Pollard contributed to the design of all of the health economic analyses and conducted all statistical and modelling analyses used in this paper

Alan Brennan, contributed to the design of the trial, oversaw the design and implementation of the modelling analyses and contributed to the design and development of the model used in the economic analyses

Simon Dixon, contributed to the design of the trial, oversaw the design and implementation of the economic analysis of the trial data and contributed to the estimation of the treatment costs used in the long term economic analyses

Norman Waugh, contributed to the design of the trial, provided expert input on treatment switching and contributed to the health economic analyses

Jackie Elliot, contributed to the design of the trial and provided clinical input into the design of the economic analyses

Simon Heller, designed the trial and provided clinical input into the design of the economic analyses

Michael Campbell contributed to the design of the trial, designed the statistical analysis plan and provided advice on the analysis of HbA1c data used in the economic modelling

Ellen Lee implemented the statistical analysis plan which included data cleaning and imputation procedures for missing HbA1c values and provided advice on the analysis of HbA1c data used in the economic modelling

For peer review only - http://bmjopen.bmj.com/site/about/guidelines.xhtml

Hasan Basarir contributed to the collection of health economic data and contributed to the design and development of the model used in the economic analyses

David White contributed to the collection of all data collecting, including the health economic data.

All authors approved the final draft.

Acknowledgements

Richard Jacques, Mónica Hernández Alava, for their advice on how to conduct the statistical analysis on HbA1c.

Peter Mansell for providing helpful comments on the full report.

The research was sponsored by Sheffield Teaching Hospitals NHS Foundation Trust. The clinical sites taking part were: Cambridge University Hospitals NHS Foundation Trust, Dumfries and Galloway Royal Infirmary, NHS Greater Glasgow and Clyde, Harrogate and District NHS Foundation Trust, King's College Hospital NHS Foundation Trust, NHS Lothian, Nottingham University Hospitals NHS Trust and Sheffield Teaching Hospitals NHS Foundation Trust.

Members of the REPOSE study group

Simon Heller was the chief investigator. Norman Waugh was the deputy chief investigator. Stephanie Amiel, Mark Evans, Fiona Green, Peter Hammond, Alan Jaap, Brian Kennon, Robert Lindsay and Peter Mansell were site principal investigators and contributed to the study design and data interpretation.

Jane Baillie, Anita Beckwith, Helen Brown, Karen Callaby, Katy Davenport, Sarah Donald, Jackie Elliott, Leila Faghahati, Sara Hartnell, Allison Housden, Kalbir Kaur Pabla, Nicola Croxon, Sheena Macdonald, Muna Mohammed, Vicky Steel, Katy Valentine, Pamela Young, Ann Boal, Patsy Clerkin, Lynn Doran, Joanne Flynn, Emma Gibb, Hilary Peddie, Bernie Quinn, Helen Rogers, Janice Shephard, Janet Carling, Ann Collins, Laura Dinning, Christine Hare, Joyce Lodge, Sutapa Ray, Debora Brown, Jenny Farmer, Alison Cox, Chris Cheyette, Pratik Choudhary, Linda East, June Ellul, Katherine Hunt, Kimberley Shaw, Ben Stothard, Lucy Diamond, Lindsay Aniello, Debbie Anderson, Kathy Cockerell, Vida Heaney, Alison Hutchison, Nicola Zammitt, Gayna Babington, Gail Bird, Janet Evans, Tasso Gazis, Nicola Maude, Karen Nunnick, Dawn Spick, Laura Fenn, Carla Gianfrancesco, Valerie Gordon, Linda Greaves, Susan Hudson, Valerie Naylor, Chloe Nisbet, Carolin Taylor, Karen Towse and Candice Ward contributed at sites to participant recruitment, intervention delivery and data collection.

Cindy Cooper, Gemma Hackney, Diana Papaioannou, Emma Whatley and David White provided central trial management, oversight and monitoring. Mike Bradburn, Michael Campbell, Munya Dimairo and Ellen Lee contributed to the statistics. Hasan Basarir, Alan Brennan, Simon Dixon and Daniel Pollard contributed to the health economics. Nina Hallowell, Jackie Kirkham, Julia Lawton and

For peer review only

For peer review only - http://bmjopen.bmj.com/site/about/guidelines.xhtml

David Rankin designed and undertook the qualitative work. Katharine Barnard led the quantitative psychosocial work. Timothy Chater and Kirsty Pemberton provided data management. Fiona Allsop and Lucy Carr provided central administration. Pamela Royle conducted literature searches and exploratory analyses. Gill Thompson and Sharon Walker provided central DAFNE support. Pauline Cowling conducted the fidelity assessment. Henry Smithson provided user representation on the management group.

Data sharing statement

Requests for patient level data and statistical code should be made to the corresponding author and will be considered by the REPOSE trial management group who, although specific consent for data sharing was not obtained, will release data on a case by case basis following the principles for sharing patient level data as described by Smith et al (2015). The presented data do not contain any direct identifiers, we will minimise indirect identifiers and remove free text data, to minimise the risk of identification.

For peer review only - http://bmjopen.bmj.com/site/about/guidelines.xhtml

References

1. The Diabetes Control and Complications Trial Research Group. The effect of intensive treatment of diabetes on the development and progression of long-term complications in insulin-dependent diabetes mellitus. . *N Engl J Med* 1993;329(14):977-86. doi: 10.1056/NEJM199309303291401
2. Dafne Study Group. Training in flexible, intensive insulin management to enable dietary freedom in people with type 1 diabetes: dose adjustment for normal eating (DAFNE) randomised controlled trial. *BMJ* 2002;325(7367):746.
3. Muhlhauser I, Bruckner I, Berger M, et al. Evaluation of an intensified insulin treatment and teaching programme as routine management of type 1 (insulin-dependent) diabetes. The Bucharest-Dusseldorf Study. *Diabetologia* 1987;30(9):681-90.
4. National Institute for Health and Care Excellence. Type 1 diabetes in adults: diagnosis and management. Clinical guideline NG17. *Online Source Available from: http://wwwniceorguk/guidance/ng17/evidence/full-guideline-435400241* 2015;Last Accessed 21st January 2016
5. National Institute for Health and Care Excellence. Continuous subcutaneous insulin infusion for the treatment of diabetes mellitus: NICE technology appraisal guidance [TA 151]. *Online Source* 2008;Available from; https://www.nice.org.uk/guidance/ta151. (Last Accessed 21st January 2016)
6. Hospital and Social Care Information Centre. National Insulin Pump Audit Report, 2013-15: England. 2016
7. Pickup J. Insulin pumps. *Int J Clin Pract Suppl* 2011(170):16-9. doi: 10.1111/j.1742-1241.2010.02574.x
8. Pickup JC. Are insulin pumps underutilized in type 1 diabetes? Yes. *Diabetes Care* 2006;29(6):1449-52. doi: 10.2337/dc06-0011
9. Cummins E, Royle P, Snaith A, et al. Clinical effectiveness and cost-effectiveness of continuous subcutaneous insulin infusion for diabetes: systematic review and economic evaluation. *Health Technol Assess* 2010;14(11):iii-iv, xi-xvi, 1-181. doi: 10.3310/hta14110
10. Colquitt JL, Green C, Sidhu MK, et al. Clinical and cost-effectiveness of continuous subcutaneous insulin infusion for diabetes. *Health Technol Assess* 2004;8(43):iii, 1-171.
11. Leotta SA, Altomare M, Carletti S, et al. Intensive diabetes management in type 1 diabetic patients in poor glycemic control treated with insulin pump therapy or multiple daily injections: Two years follow-up. *4th International Conference on Advanced Technologies and Treatments for Diabetes*;London, 2011
12. White D, Waugh N, Elliott J, et al. The Relative Effectiveness of Pumps Over MDI and Structured Education (REPOSE): study protocol for a cluster randomised controlled trial. *BMJ Open* 2014;4(9):e006204. doi: 10.1136/bmjopen-2014-006204
13. National Institute for Health and Care Excellence. Guide to the methods of technology appraisal 2013. *Online Source* 2013;Available from; http://www.nice.org.uk/article/pmg9/resources/non-guidance-guide-to-the-methods-of-technology-appraisal-2013-pdf. Last accessed 13th July 2015.
14. Heller S, Lawton J, Amiel S, et al. Improving management of type 1 diabetes in the UK: the Dose Adjustment For Normal Eating (DAFNE) programme as a research test-bed. A mixed-method analysis of the barriers to and facilitators of successful diabetes self-management, a health economic analysis, a cluster randomised controlled trial of different models of delivery of an educational intervention and the potential of insulin pumps and additional educator input to improve outcomes. *Programme grants for applied research* 2014;2(5) doi: 10.3310/pgfar02050
15. Thokala P, Kruger J, Brennan A, et al. Assessing the cost-effectiveness of type 1 diabetes interventions: the Sheffield type 1 diabetes policy model. *Diabet Med* 2014;31(4):477-86. doi: 10.1111/dme.12371

For peer review only - http://bmjopen.bmj.com/site/about/guidelines.xhtml

16. Groot Koerkamp B, Weinstein MC, Stijnen T, et al. Uncertainty and patient heterogeneity in medical decision models. *Med Decis Making* 2010;30(2):194-205. doi: 10.1177/0272989X09342277
17. Kaplan EL, Meier P. Nonparametric-Estimation from Incomplete Observations. *J Am Stat Assoc* 1958;53(282):457-81. doi: Doi 10.2307/2281868
18. Collett D. Survival Data in Medical Research. 2nd ed. Boca Ration (FL): Chapman & Hall / CRC 2003.
19. Cribari-Neto F, Zeileis A. Beta Regression in R. *J Stat Softw* 2010;34(2):1-24.
20. Orr CJ, Hopman W, Yen JL, et al. Long-term efficacy of insulin pump therapy on glycemic control in adults with type 1 diabetes mellitus. *Diabetes Technol Ther* 2015;17(1):49-54. doi: 10.1089/dia.2014.0131
21. Carlsson BM, Attvall S, Clements M, et al. Insulin pump-long-term effects on glycemic control: an observational study at 10 diabetes clinics in Sweden. *Diabetes Technol Ther* 2013;15(4):302-7. doi: 10.1089/dia.2012.0286
22. Bruttomesso D, Pianta A, Crazzolara D, et al. Continuous subcutaneous insulin infusion (CSII) in the Veneto region: efficacy, acceptability and quality of life. *Diabet Med* 2002;19(8):628-34.
23. Gunn D, Mansell P. Glycaemic control and weight 7 years after Dose Adjustment For Normal Eating (DAFNE) structured education in Type 1 diabetes. *Diabetic Med* 2012;29(6):807-12.
24. Elliott J, Jacques RM, Kruger J, et al. Substantial reductions in the number of diabetic ketoacidosis and severe hypoglycaemia episodes requiring emergency treatment lead to reduced costs after structured education in adults with Type 1 diabetes. *Diabet Med* 2014;31(7):847-53. doi: 10.1111/dme.12441
25. Curtis L. Unit Costs of Health & Social Care 2014. *Online Source* 2014;Available from; http://www.pssru.ac.uk/project-pages/unit-costs/2014/index.php. Last Accessed 30th June 2015
26. British Medical Association and Royal Pharmaceutical Society of Great Britain. British National Formulary August 2015. *Online Source* 2015;Available from: https://www.medicinescomplete.com, Last Accessed 9th September 2015
27. Department of Health. NHS reference costs 2013 to 2014. *Online Source* 2014;Available from; https://www.gov.uk/government/publications/nhs-reference-costs-2013-to-2014. Last accessed 17th July 2015
28. Health & Social Care Information Centre. Prescription Cost Analysis England 2014. *Online Source* 2015;Available from; http://www.hscic.gov.uk/catalogue/PUB17274. Last Accessed 2nd Novemeber 2015
29. Brown MM, Brown GC, Sharma S, et al. Quality of life with visual acuity loss from diabetic retinopathy and age-related macular degeneration. *Arch Ophthalmol* 2002;120(4):481-4.
30. Roze S, Smith-Palmer J, Valentine W, et al. Cost-effectiveness of continuous subcutaneous insulin infusion versus multiple daily injections of insulin in Type 1 diabetes: a systematic review. *Diabetic Med* 2015;32(11):10. doi: DOI: 10.1111/dme.12792
31. Weissberg-Benchell J, Antisdel-Lomaglio J, Seshadri R. Insulin pump therapy: a meta-analysis. *Diabetes Care* 2003;26(4):1079-87.
32. DAFNE. DAFNE Fact Sheet Six. *Online Source* 2012;Available from; http://www.dafne.uk.com/uploads/135/documents/06_factsheetsix_12pt_18_06_12.pdf. Last Accessed 1st July 2015
33. Department of Health. NHS Reference Costs 2010-11. London: Department of Health 2011.
34. British Medical Association and Royal Pharmaceutical Society of Great Britain. British National Formulary. 63rd edn ed. London: BMA and RPS 2012.
35. McEwan P, Poole CD, Tetlow T, et al. Evaluation of the cost-effectiveness of insulin glargine versus NPH insulin for the treatment of type 1 diabetes in the UK. *Curr Med Res Opin* 2007;23:S7-S19. doi: 10.1185/030079906x167561

For peer review only - http://bmjopen.bmj.com/site/about/guidelines.xhtml

36. Currie CJ, Poole CD, Woehl A, et al. The financial costs of healthcare treatment for people with Type 1 or Type 2 diabetes in the UK with particular reference to differing severity of peripheral neuropathy. *Diabet Med* 2007;24(2):187-94. doi: 10.1111/j.1464-5491.2006.02057.x
37. National Institute for Health and Care Excellence. Ranibizumab for treating diabetic macular oedema (rapid review of technology appraisal guidance 237). NICE technology appraisal guidance 274. *Onlince Source* 2013;Available from: https://www.nice.org.uk/guidance/ta274/resources/ranibizumab-for-treating-diabetic-macular-oedema-rapid-review-of-technology-appraisal-guidance-237-82600612458181
38. British Medical Association and Royal Pharmaceutical Society of Great Britain. British National Formulary August 2016. *Online Source* 2016;Available from: https://www.medicinescomplete.com, Last Accessed 12th December 2016
39. Clarke P, Gray A, Legood R, et al. The impact of diabetes-related complications on healthcare costs: results from the United Kingdom Prospective Diabetes Study (UKPDS Study No. 65). *Diabet Med* 2003;20(6):442-50.
40. Peasgood T, Brennan A, Mansell P, et al. The impact of diabetes-related complications on preference-based measures of health-related quality of life in adults with Type I diabetes. *Medical Decision Making* 2016;In Press doi: DOI: 10.1177/0272989X16658660
41. Coffey JT, Brandle M, Zhou H, et al. Valuing health-related quality of life in diabetes. *Diabetes Care* 2002;25(12):2238-43.
42. Alva M, Gray A, Mihaylova B, et al. The effect of diabetes complications on health-related quality of life: the importance of longitudinal data to address patient heterogeneity. *Health Econ* 2014;23(4):487-500. doi: 10.1002/hec.2930
43. Clarke P, Gray A, Holman R. Estimating utility values for health states of type 2 diabetic patients using the EQ-5D (UKPDS 62). *Med Decis Making* 2002;22(4):340-9.

For peer review only - http://bmjopen.bmj.com/site/about/guidelines.xhtml

Tables

Table 1: The baseline characteristics of REPOSE participants and the simulated cohort

Characteristic	REPOSE trial population (N=260)	Simulated cohort (N=5,000)
**Continuous variables mean (standard deviation) [% of individuals with data prior to imputation]**		
Baseline HbA1c (mmol/mol)	76.0 (18.6) [100%]	75.9 (18.2) [100%]
Baseline HbA1c (%)	9.1 (1.7) [100%]	9.1 (1.7) [100%]
Age (years)	40.4 (13.4) [100%]	40.4 (13.3) [100%]
Diabetes duration (years)	18.0 (12.5) [100%]	18.0 (12.3) [100%]
Triglycerides (mmol/mol)	1.4 (1.0) [100%]	1.4 (0.9) [100%]
Total Cholesterol (mmol/mol)	4.9 (0.9) [99.6%]	4.9 (0.9) [99.7%]
HDL cholesterol (mmol/mol)	1.6 (0.4) [96.5%]	1.6 (0.4) [96.4%]
LDL cholesterol (mmol/mol)	2.8 (0.9) [96.2%]	2.7 (0.9) [96.1%]
Systolic blood pressure	131.4 (16.4) [98.8%]	131.3 (16.0) [98.9%]
Baseline cost of insulin	£357.24 (147.65) [94.8%]	£360.39 (157.92) [98.4%]
Baseline cost of diabetes related contacts	£561.61 (885.92) [100%]	£571.63 (928.92) [100%]
**Categorical variables n/N (percentage) [% of individuals prior to imputation]**		
Gender		
Female	104/260 (40.0%) [40.0%]	1990/5000 (39.8%) [39.9%]
Male	152/260 (58.5%) [58.5%]	2950/5000 (59.0%) [60.1%]
Missing	4/260 (1.5%) [1.5%]	0/5000 (0.0%) [1.4%]
Physical Activity		
Low	67/260 (25.8%) [25.8%]	1266/5000 (25.3%) [25.3%]
Medium	128/260 (49.2%) [49.2%]	2471/5000 (49.4%) [49.4%]
High	65/260 (25.0%) [25.0%]	1263/5000 (25.3%) [25.3%]
Smoking Status		
Current	50/260 (19.2%) [19.2%]	951/5000 (19.2%) [19.2%]
Former	67/260 (25.8%) [25.8%]	1325/5000 (26.3%) [26.3%]
Never	143/260 (55.0%) [55.0%]	2724/5000 (54.5%) [54.5%]
Race		
White	258/260 (99.2%) [99.2%]	4959/5000 (99.2%) [99.2%]
Black	2/260 (0.8%) [0.8%]	41/5000 (0.8%) [0.8%]
Nephropathy		
No complications	239/260 (91.9%) [91.9%]	4600/5000 (92.0%) [92.0%]
Microalbuminuria	13/260 (5.0%) [5.0%]	234/5000 (4.7%) [4.7%]
Macroalbuminuria	7/260 (2.7%) [2.7%]	152/5000 (3.0%) [3.0%]
Dialysis or transplant	1/260 (0.4%) [0.4%]	14/5000 (0.4%) [0.4%]
Neuropathy		
No complications	238/260 (91.5%) [91.5%]	4599/5000 (92.0%) [92.0%]
Neuropathy or Ulcers	22/260 (8.5%) [8.5%]	401/5000 (8.0%) [8.0%]
Retinopathy		
No complications	145/260 (55.8%) [55.8%]	28/5000 (56.0%) [56.0%]
Background diabetic retinopathy	91/260 (35.0%) [35.0%]	1740/5000 (34.8%) [34.8%]
Proliferative diabetic retinopathy	24/260 (9.2%) [9.2%]	465/5000 (9.3%) [9.3%]
Myocardial Infarction		
No complications	255/260 (98.1%) [98.1%]	4896/5000 (97.9%) [97.9%]
Myocardial Infarction	5/260 (1.9%) [1.9%]	104/5000 (2.1%) [2.1%]
Stroke		
No complications	259/260 (99.6%) [99.6%]	4983/5000 (99.7%) [99.7%]
Stroke	1/260 (0.4%) [0.4%]	17/5000 (0.3%) [0.3%]
Heart Failure		
No complications	259/260 (99.6%) [99.6%]	4934/5000 (99.6%) [99.6%]
Heart Failure	1/260 (0.4%) [0.4%]	18/5000 (0.4%) [0.4%]
Angina		
No complications	257/260 (98.9%) [98.9%]	4934/5000 (98.7%) [98.7%]
Angina	3/260 (1.2%) [1.2%]	66/5000 (1.3%) [1.3%]

HDL; high density lipoprotein, LDL Low-density lipoprotein

For peer review only

For peer review only - http://bmjopen.bmj.com/site/about/guidelines.xhtml

Table 2: The health state cost parameters used in the Sheffield Type 1 Diabetes Policy Model

Part A: Regression functions for estimated costs in year 1 and ongoing based on REPOSE trial data (Multivariate normal distributions*)

	Annual Cost of Insulin and MDI consumables (year 1)	Annual Cost of Insulin and MDI consumables (ongoing)	Annual Cost of DRC (year 1)	Annual Cost of DRC (ongoing)	Annual Cost of Insulin Pump and associated consumables (year 1)	Annual Cost of Insulin Pump and associated consumables (ongoing)	**Sources**
Multiplier for the baseline DRC cost (β_1)	-	-	0.11	0.03	-	-	REPOSE trial data in year 1. (year 1 costs)
Multiplier for the baseline insulin cost (β_2)	0.97	1.04	-	-	-	-	REPOSE trial data in year 2. (ongoing costs)
Multiplier for the Baseline HbA1c (DCCT % scale) (β_3)	5.08	12.81	-21.66	12.15	-	-	NHS reference costs[27]
Receiving pump at the start of the year (β_4)	-517.91	-527.64	129.08	88.99	2056.11	2050.99	Pump costing survey
Switch from pump to MDI (β_5)	554.47	153.35	280.16	-47.10	-1143.68	-905.03	
Switch from MDI to pump (β_6)	0†	-353.27	733.95	201.93	804.57	1134.27	
Constant (β_0)	381.77	324.53	415.46	299.80	0.00	0.00	

The cost for each total cost in part A is calculated using the following formula:
Total cost = β_0 + β_1*Individual's baseline diabetes related contact cost + β_2*Individual's baseline insulin cost + β_3*Individual's baseline HbA1c (DCCT % scale) + β_4 * Individual's treatment at the start of the year (1=pump, 0=MDI) + β_5*(1 = switched from pump to MDI, 0 = didn't switch from pump to MDI) + β_6*(1= Switched from MDI to pump, 0 =didn't switch from MDI to pump)

*, the variance covariance matrices used to parameterise the multivariate normal distribution are provided in supplementary material B Tables 4, 7 and 12.

-, this value was not included as a covariate in the regression formula and is taken to be a zero value in the total cost formula

†; this variable was included in the original regressions, however the model would converge when this covariate was included, so this parameters is taken to be a zero value in the total cost formula

Part B: Costs of DAFNE Fixed parameters

**Health State**	**Mean cost (£)**	**SE**	**Source**	**Health State**	**Mean cost (£)**	**SE**	**Source**
Cost of a DAFNE course	363.10	None	DAFNE Fact Sheet Six[32]	Cost of pre-course fitting session for pump users	28.82	None	DAFNE Fact Sheet Six [32] & REPOSE trial data

Part C: Costs of adverse events, comorbidities and complications Gamma Distributions

**Health State**	**Mean Cost (£)**	**SE**	**Source**	**Health State**	**Mean cost (£)**	**SE**	**Source**
**Adverse Events**							
Hypoglycaemia	187	18.69	Heller *et al.*[14]	DKA with Hospitalisation	1399	140	NHS reference costs[33]
**Nephropathy**							
Ongoing yearly cost of Microalbuminuria	36	3.56	BNF[34] and McEwan *et al.*[35]	Ongoing yearly cost of Microalbuminuria ongoing	36	3.56	BNF[34] and McEwan *et al.*[35]

For peer review only - http://bmjopen.bmj.com/site/about/guidelines.xhtml

Ongoing yearly cost of ESRD	24436	2444	NHS Reference costs[33]	Death due to ESRD	0	0	Assumed equal to Zero
**Neuropathy**							
Clinically Confirmed Neuropathy	271	27.14	Currie *et al.*[36]	Clinical Neuropathy	271	27.14	Currie *et al.*[36]
Diabetic foot syndrome	2848	285	NHS Reference costs[33]	PAD with amputation (year 1)	7221	722	NHS Reference costs[33]
Ongoing Yearly cost of PAD with amputation	439	43.93	McEwan *et al.*[35]				
**Retinopathy**							
Background Retinopathy	145	14.47	McEwan *et al.*[35]	Proliferative Retinopathy	661	66.11	McEwan *et al.*[35]
Macular edema (year 1)	5710	571.0	NICE[37], BNF[38]	Macular edema (year 2)	3416	341.6	NICE[37], BNF[38]
Macular edema (year 3)	2562	256.2	NICE[37], BNF[38]	Macular edema (Ongoing)	277	27.7	NICE[37], BNF[38]
Blindness (year 1)	1584	158	Clarke *et al.*[39]	Blindness (ongoing)	519	51.88	Clarke *et al.*[39]
**Cardiovascular**							
First MI (year 1)	6788	679	Clarke *et al.*[39]	Second MI (year 1)	6788	679	Clarke *et al.*[39]
Final MI (year 1)	6788	679	Clarke *et al.*[39]	Ongoing yearly cost of a MI	904	90.43	Clarke *et al.*[39]
Fatal MI	2101	210	Clarke *et al.*[39]	Heart Failure (year 1)	3818	382	Clarke *et al.*[39]
Heart Failure (ongoing)	1173	117	Clarke *et al.*[39]	Fatal HF	3818	382	Clarke *et al.*[39]
First Stroke (year 1)	4361	436	Clarke *et al.*[39]	Second Stroke	4361	436	Clarke *et al.*[39]
Fatal Stroke	5684	568.45	Clarke *et al.*[39]	First Stroke (ongoing)	559	55.90	Clarke *et al.*[39]
Angina (year 1)	3397	340	Clarke *et al.*[39]	Angina (ongoing)	951	95.09	Clarke *et al.*[39]

BNF, British National Formulary; ESRD, End-Stage Renal Disease; PAD, Peripheral Arterial Disease; DAFNE, Dose Adjustment for Normal Eating; pump, insulin pumps; MDI, multiple daily injections

For peer review only

For peer review only - http://bmjopen.bmj.com/site/about/guidelines.xhtml

Table 3: The base case utility parameters

Health state for event	Utility	SE	Beta distribution *Alpha*	*Beta*	Source
Baseline utility value					
Male with type 1 diabetes and no complications	0.866	0.010	947.79	146.90	Peasgood *et al.*[40]
	**Disutility**	**SE**	**Gamma distribution** ***Alpha***	***Beta***	**Source**
Complications or covariates					
Female with type 1 diabetes and no complications	0.0236	0.008	8.70	0.003	Peasgood *et al.*[40]*
**Adverse events**					
Severe Hypoglycaemia	-0.002	-0.002	1	0.002	Peasgood *et al.*[40]
Diabetic Ketoacidosis	-0.0091	-0.01	0.83	0.01	Peasgood *et al.*[40]*
**Nephropathy**					
Microalbuminuria	0				Assumption
Microalbuminuria	-0.017	0.01	2.89	0.01	Coffey *et al.*[41]
ESRD	-0.078	0.026	9	0.01	Coffey *et al.*[41]
**Neuropathy**					
Clinical neuropathy	-0.055	0.01	30.25	0.002	Coffey *et al.*[41]
Clinically confirmed neuropathy	-0.055	0.01	30.25	0.002	Coffey *et al.*[41]
Diabetic foot syndrome	-0.1042	-0.119	0.77	0.14	Peasgood *et al.*[40]
PAD with amputation	-0.1172	-0.055	4.54	0.03	Peasgood *et al.*[40]*
**Retinopathy**					
Background retinopathy	-0.0544	-0.023	5.59	0.01	Peasgood *et al.*[40]
Proliferative retinopathy	-0.0288	-0.026	1.23	0.02	Peasgood *et al.*[40]
Blindness	-0.208	0.013	256	0.001	Coffey *et al.*[41]
**Cardiovascular**					
MI (first year)	-0.065	0.03	4.69	0.01	Alva *et al.*[42]
MI (subsequent years)	-0.057	0.03	3.61	0.02	Alva *et al.*[42]
Heart failure	-0.101	0.032	9.96	0.010	Alva *et al.*[42]
Stroke	-0.165	0.035	22.22	0.007	Alva *et al.*[42]
Angina	-0.09	0.018	25	0.004	Clarke *et al.*[43]†

SE – standard error; ESRD – end stage renal disease; PAD – peripheral arterial disease; MI – myocardial infarction
* - a parameter value was not available in the author's preferred statistical model; † - value is presented in Table 5 as ischaemic heart disease

For peer review only

Table 4: Long-Term Cost-Effectiveness Analysis - base case results using probabilistic sensitivity analysis

	MDI+DAFNE	pump+DAFNE	Incremental
**Mean Lifetime Discounted Costs per Person**			
Intervention costs			
Insulin and MDI consumables	£12,215	£5,476	-£6,740
Diabetes Related contacts	£5,023	£6,289	£1,266
Insulin pumps and pump consumables	£2,228	£28,967	£26,739
DAFNE course	£363	£392	£29
**Subtotal Intervention costs**	£19,829	£41,124	£21,295
Adverse event costs			
Severe Hypoglycaemia	£133	£41	-£92
Diabetic Ketoacidosis	£1,161	£882	-£279
**Subtotal cost of adverse events**	£1,294	£922	-£371
Long term complication costs			
Nephropathy	£40,786	£38,853	-£1,933
Neuropathy	£1,859	£1,805	-£53
Retinopathy + Macular Edema	£6,365	£6,263	-£102
Myocardial Infarction	£1,838	£1,844	£6
Heart Failure	£607	£609	£2
Stroke	£253	£254	£0
Angina	£1,134	£1,143	£8
**Total cost of long term complications**	£52,841	£50,771	-£2,070
**Total costs**	£73,964	£92,817	£18,853<br>(95% CI £6,175, £31,645)
**Mean undiscounted life years per person**			
**Total Life years**	28.3181	28.7999	0.3790<br>(95% CI -2.7392 to 3.3403)
**Mean Discounted QALYs per person**			
QALYs lived<br>(excluding decrements due to complications)	13.9145	14.0292	0.1147
QALYs lost due to adverse events			
Severe Hypoglycaemia	-0.0014	-0.0004	0.0009
Diabetic Ketoacidosis	-0.0075	-0.0057	0.0018
**Subtotal QALYs due to adverse events**	-0.0088	-0.0061	0.0027
QALYs lost due to complications			
Nephropathy	-0.1853	-0.1792	0.0061
Neuropathy	-0.3092	-0.3010	0.0082
Retinopathy + Macular Edema	-0.3316	-0.3293	0.0022
Myocardial Infarction	-0.0528	-0.0532	-0.0004
Heart Failure	-0.0385	-0.0387	-0.0002
Stroke	-0.0343	-0.0345	-0.0002
Angina	-0.0754	-0.0761	-0.0007
**Subtotal QALYs lost due to complications**	-1.0271	-1.0120	0.0152
**Total QALYs**	12.8785	13.0111	0.1326<br>(95%CI-0.7087, 0.9623)
**Summary**			
Total mean discounted costs per person	£80,471	£99,337	£18,853<br>(95% CI £6,175, £31,645)
Total mean undiscounted life years per person	28.3181	28.7999	0.3790<br>(95% CI -2.7392 to 3.3403)
Total mean discounted QALYs per person	12.8785	13.0111	0.1326<br>(95%CI-0.7087, 0.9623)
ICER (£/QALY gained)			£142,195
Probability that pump+DAFNE is cost-effective at a threshold of £20,000 per QALY gained			14.0%
MDI – multiple daily injections; DAFNE – dose adjustment for normal eating; pump – insulin pumps; CI – confidence interval; QALY – quality adjusted life year; ICER – incremental cost-effectiveness ratio			

For peer review only

For peer review only - http://bmjopen.bmj.com/site/about/guidelines.xhtml

Table 5: The incremental cost-effectiveness ratio associated with insulin pumps for different HbA1c reductions (for all adults with T1DM) and annualised prices of insulin pumps and insulin pump consumables

	3.3 mmol/mol (0.3%)	4.4 mmol/mol (0.4%)	5.5 mmol/mol (0.5%)	6.6 mmol/mol (0.6%)	7.7 mmol/mol (0.7%)	8.7 mmol/mol (0.8%)	9.8 mmol/mol (0.9%)	10.9 mmol/mol (1.0%)	12.0 mmol/mol (1.1%)	13.1 mmol/mol (1.2%)
£2,060 (100%)	£75,710	£85,990	£64,493	£47,667	£34,280	£27,951	£25,979	£26,258	£18,953	£17,610
£1,957 (95%)	£69,830	£79,106	£58,901	£43,527	£31,132	£25,128	£23,494	£23,736	£16,730	£15,515
£1,854 (90%)	£63,950	£72,222	£53,309	£39,387	£27,985	£22,306	£21,009	£21,213	£14,507	£13,419
£1,751 (85%)	£58,070	£65,338	£47,717	£35,247	£24,837	£19,483	£18,524	£18,691	£12,284	£11,323
£1,648 (80%)	£52,189	£58,454	£42,125	£31,107	£21,689	£16,661	£16,039	£16,169	£10,060	£9,227
£1,545 (75%)	£46,309	£51,570	£36,533	£26,967	£18,542	£13,838	£13,554	£13,646	£7,837	£7,131
£1,442 (70%)	£40,429	£44,686	£30,941	£22,827	£15,394	£11,016	£11,070	£11,124	£5,614	£5,036
£1,339 (65%)	£34,549	£37,802	£25,349	£18,687	£12,246	£8,193	£8,585	£8,602	£3,391	£2,940
£1,236 (60%)	£28,668	£30,917	£19,757	£14,547	£9,099	£5,370	£6,100	£6,079	£1,167	£844
£1,133 (55%)	£22,788	£24,033	£14,164	£10,407	£5,951	£2,548	£3,615	£3,557	Dominates	Dominates
£1,030 (50%)	£16,908	£17,149	£8,572	£6,267	£2,803	Dominates	£1,130	£1,035	Dominates	Dominates

For peer review only - http://bmjopen.bmj.com/site/about/guidelines.xhtml

Figure Legends

Figure 1: The results of the economic analyses presented on the cost-effectiveness plane for: a) the base case PSA, b) the results of the scenario analyses and c) the results of the pre specified subgroup analyses

For peer review only

For peer review only - http://bmjopen.bmj.com/site/about/guidelines.xhtml

Figure 1

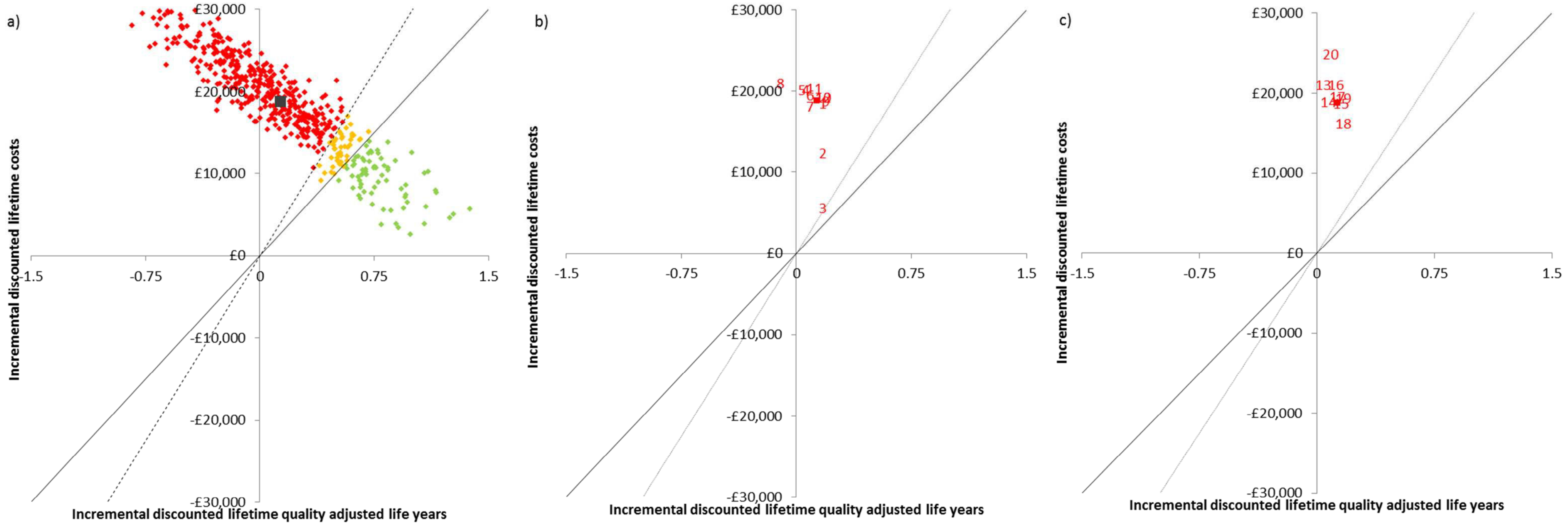

Key for the Scenario analyses

1 = Pump prices were estimated from Riemsma et al.
2 = 25% price reduction in insulin pumps and consumables
3 = 50% price reduction in insulin pumps and consumables
4 = HbA1c differences between CSII and MDI were estimated in the ITT population
5 = 4 & no change in HbA1c when an individual switches treatment#
6 = Base case & no change in Hba1c when switching
7= Post-trial HbA1c progression is 0.045% per annum in both arms (Source DCCT trial)
8 = Individuals return to their baseline HbA1c after 3 years and no progression thereafter
9 = HbA1c effects occur at the start of the year, rather than the end of the year
10 = Individuals return to their baseline risk of hypos and DKA at three years
11 = Switching probabilities were estimated directly from the Kaplan - Meier curves
12 = The Utility value for blindness was -0.26 [Source Brown *et al]*

Key for the subgroup analyses

13 = Baseline HbA1c < 69 mmol/mol (8.5%)
14 = Baseline HbA1c ≥ 69 mmol/mol (8.5%)
15 = Baseline HbA1c ≥ 58 mmol/mol (7.5%)
16 = 69 mmol/mol (8.5%) >baseline HbA1c ≥ 58 mmol/mol (7.5%)
17 = 80 mmol/mol (9.5%) > baseline HbA1c ≥ 69 mmol/mol (8.5%)
18 = Baseline HbA1c ≥ 80 mmol/mol (9.5%)
19 = All individuals in the per protocol population
20 = All individuals in the per protocol population & no treatment switching

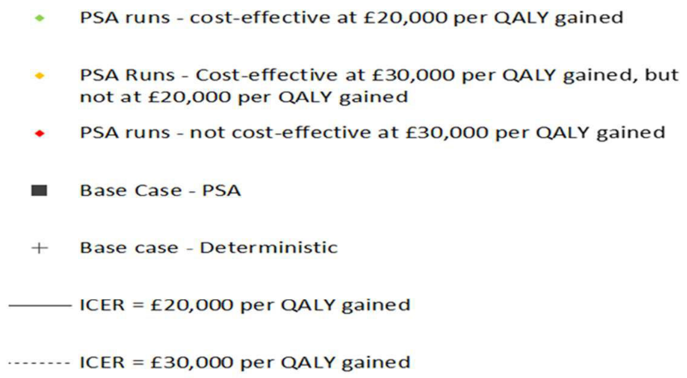

For peer review only - http://bmjopen.bmj.com/site/about/guidelines.xhtml

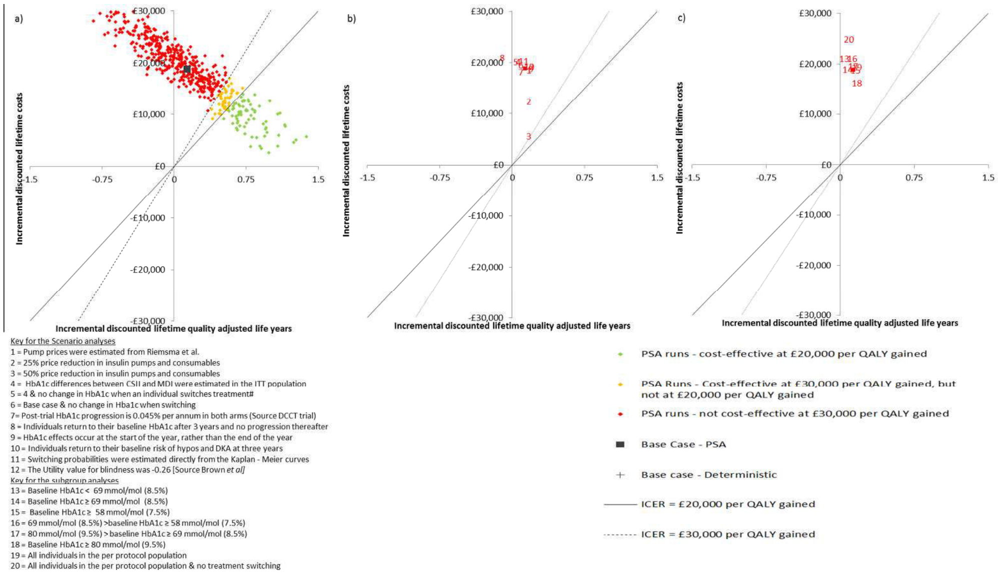

The results of the economic analyses presented on the cost-effectiveness plane for: a) the base case PSA, b) the results of the scenario analyses and c) the results of the pre specified subgroup analyses

361x207mm (150 x 150 DPI)

For peer review only

1 Methods

1.1 Resource Use by individuals in REPOSE over the 2 year follow up period

Resource use was collected either on an ongoing basis or was self-reported by the individuals in the trial at baseline or at a follow up period (6 months, 1 year and 2 years post-randomisation). All unit costs used to value the reported resource use in the economic evaluation alongside the trial (EEACT), apart from the costs associated with insulin use, are presented in Table **1**. The unit costs associated with insulin use are reported separately in Table 2.

Data was collected on an ongoing basis throughout the trial for all inpatient hospitalisations which were not scheduled to treat a pre-existing condition, incidence of severe hypoglycaemia and whether or not an individual used an insulin pump. At each recorded hospital admission (either self-reported or collected on an ongoing basis) information was collected on the cause. The possible causes for an admission in the REPOSE dataset were: diabetic ketoacidosis (DKA); myocardial infarction; severe hypoglycaemia; ischaemic heart disease; unstable angina; heart failure; foot ulcer; and renal disease. Severe hypoglycaemia was defined in the REPOSE trial as been any hypoglycaemic episode which an individual was unable to treat themselves. If it was reported that an individual did not have an inpatient admission or a paramedic callout for their severe hypoglycaemic event, then it was assumed that a friend or family member provided aid to the individual. These adverse events had no implications for NHS resource use, so these episodes of severe hypoglycaemia were assumed to have no monetary cost to the NHS.

Individual's self-reported resource use since the last REPOSE visit was collected on: the number of face to face and telephone diabetes related contacts with health care professionals; the use of lipid lowering, antiplatelet or depression medication; data at baseline on number of diabetes related admissions in the past year, days spent in hospital and the reason for admission; and the type of insulin used, the average daily dose in the week preceding data collection, the number of injections per day, and the method of insulin delivery. The unit costs associated with insulin use are presented separately from the rest of the unit costs in Table 2. The daily cost of insulin was multiplied by the number of days between each data collection period (6 months, 1 year, and 2 years) to calculate the cost of insulin in the first and second year. If an individual was a receiving insulin pump therapy, the cost of needles, insulin pens and syringes were not applied, as these were already included in the estimates of

For peer review only

the cost of insulin pump consumables. From this information a cost of insulin for each individual in the REPOSE trial was calculated.

Table 1: The unit costs used in the within trial analysis of the REPOSE data

Costs used in the within trial analyses	Cost (2013/14)	Notes
**DAFNE courses**		
Cost of a DAFNE course	£363.10	DAFNE fact sheet 6[1]
Cost of a pre-course pump fitting session	£28.82	REPOSE trial data and DAFNE fact sheet 6[1]
**Hypoglycaemia**		
Cost of hypoglycaemia admission	£446.73	NHS reference costs 2013-14.[2] Non-elective inpatient short stay. FCE weighted average of the currency codes: KB01C, KB01D, KB01E, KB01F, KB02G, KB02H, KB02J, KB02K
Paramedic cost per case	£233.58	Elliot *et al* 2014.[3] Table 3.
**Cost of inpatient admissions**		
Diabetic ketoacidosis		
Cost of the first day	£527.78	NHS reference costs 2012-13. Non-elective inpatient short stay. Currency code PA67Z.
Cost of subsequent days	£284.42	NHS reference costs 2012-13. Non-elective inpatients excess bed days. Currency code PA67Z.
Renal hospitalisation		
Cost of the first day	£471.70	NHS reference costs 2013-14.[2]Non-elective inpatients short stay. Weighted average of the currency codes: LA09J, LA09K, LA09L, LA09M, LA09N, LA09P, LA09Q
Cost of subsequent days	£257.87	NHS reference costs 2013-14.[2]Non-elective inpatients excess bed days. Currency codes: LA09J, LA09K, LA09L, LA09M, LA09N, LA09P, LA09Q
Myocardial Infarction		
Cost of the first day	£560.60	NHS reference costs 2013-14.[2]Non-elective inpatients short stay. Weighted average of the currency codes: EB10A, EB10B, EB10C, EB10D, and EB10E.
Cost of subsequent days	£248.89	NHS reference costs 2013-14.[2]Non-elective inpatients excess bed days. Currency codes: EB10A, EB10B, EB10C, EB10D, EB10E.
Foot Ulcer		
Cost of the first day	£509.39	NHS reference costs 2012-13. Non-elective inpatient short stay. Currency codes: KB03C, KB03D, KB03E.
Cost of subsequent days	£156.34	NHS reference costs 2012-13. Non-elective inpatient short stay day. Currency codes: KB03C, KB03D, KB03E.

For peer review only

Other Inpatient stays		
Cost of the first day	£755.44	NHS reference costs 2012-13. Non-elective inpatient short stay. Currency code PA68Z.
Cost of subsequent days	£335.81	NHS reference costs 2012-13. Non elective inpatient excess bed day. Currency code PA68Z.
**Medication costs (per quarter)**		
Cost of lipid medication	£9.27	Prescription Cost Analysis: England 2011 (BNF chapter 2 section 12)
Cost of antiplatelet medication	£1.87	Prescription Cost Analysis: England 2011 (BNF chapter 2 section 9)
Cost of depression medication	£6.08	Prescription Cost Analysis: England 2011 (BNF chapter 4 section 3)
**Cost of diabetes related contacts**		
Cost of a face to face clinic	£105.49	NHS reference costs 2013-14. Non consultant led outpatient attendance. Non admitted face to face follow up. Service description: Diabetic Medicine
Cost of a telephone contact	£75.80	NHS reference costs 2013-14. Non consultant led outpatient attendance. Non admitted non face to face follow up. Service description: Diabetic Medicine

MDI, multiple daily injections: CSII, continuous subcutaneous insulin infusion; HSCIC, Health & Social Care Information Centre

For peer review only

Table 2: The unit costs of insulin

Item	Average unit cost	Number of units	Cost per unit	Associated yearly cost of an insulin pen	Source
Consumables related to multiple daily injections					
Cost of an insulin needle	£0.11	N/A	N/A	N/A	HSCIC[4]
Cost of an insulin syringe	£0.13	N/A	N/A	N/A	HSCIC[4]
Quick Acting Insulin					
**Human Insulin**					
Vial	£9.87	1000	£0.01	N/A	BNF[5], HSCIC[4]
Cartridges for a reusable pen	£18.97	1500	£0.01	£8.78	
**Animal Insulin**					
Vial	£26.15	1000	£0.03	N/A	BNF[5], HSCIC[4]
Cartridges for a reusable pen	£38.29	1500	£0.03	£5.97	
**Insulin Aspart (NovoRapid)**					
Vial	£14.08	1000	£0.01	N/A	BNF[5], HSCIC[4]
Cartridges for a reusable pen	£28.31	1500	£0.02	£9.59	
Disposable Pen	£30.63	1500	£0.02	N/A	
**Insulin Lispro (Humalog)**					
Vial	£16.61	1000	£0.02	N/A	
Cartridges for a reusable pen	£28.31	1500	£0.02	£8.86	BNF[5], HSCIC[4]
Disposable Pen	£28.31	1500	£0.02	N/A	
**Insulin Glulisine (Apidra)**					
Vial	£16.00	1000	£0.02	N/A	
Cartridges for a reusable pen	£28.30	1500	£0.02	£7.86	BNF[5], HSCIC[4]
Disposable Pen	£28.30	1500	£0.02	N/A	
Background Insulin					
**Human Insulin**					
Vial	£10.41	988	£0.01	N/A	
Cartridges for a reusable pen	£21.52	1500	£0.01	£9.30	BNF[5], HSCIC[4]
Disposable Pen	£21.05	1500	£0.01	N/A	
**Animal Insulin**					
Vial	£26.17	1000	£0.03	N/A	BNF[5], HSCIC[4]
Cartridges for a reusable pen	£38.32	1500	£0.03	£9.57	
**Insulin Detemir (Levemir)**					
Cartridges for a reusable pen	£42.00	1500	£0.03	£9.59	BNF[5], HSCIC[4]
Disposable Pen	£42.10	1500	£0.03	N/A	
**Insulin Glargine (Lantus)**					

For peer review only

Vial	£30.68	1000	£0.03	N/A	
Cartridges for a reusable pen	£41.50	1500	£0.03	£7.86	BNF[5], HSCIC[4]
Disposable Pen	£41.50	1500	£0.03	N/A	
Mixed Insulin					
**Biphasic Isophane Insulin**					
**Animal Insulin**					
Vial	£25.20	1000	£0.03	N/A	BNF[5], HSCIC[4]
Cartridges for a reusable pen	£37.80	1500	£0.03	£5.97	
**Human Insulin**					
Vial	£15.43	987	£0.02	N/A	BNF[5], HSCIC[4]
Cartridges for a reusable pen	£18.94	1500	£0.01	£7.74	
Disposable Pen	£21.43	1500	£0.01	N/A	
**Biphasic Insulin Aspart**					
Cartridges for a reusable pen	£28.79	£28.79	£0.02	£9.59	BNF[5], HSCIC[4]
Disposable Pen	£29.89	£29.89	£0.02		
**Biphasic Insulin Lispro**					
Vial	£16.61	1000	£0.02		
Cartridge for reusable pen	£29.03	1500	£0.02	£8.93	BNF[5], HSCIC[4]
Disposable Pen	£30.13	1500	£0.02		

BNF, British National Formulary; HSCIC, Health & Social Care Information Centre

For peer review only

1.1.1 Treatment switching

During the REPOSE trial, it was possible for individuals to switch from insulin delivery mechanism to the other i.e. to switch from insulin pump therapy to multiple daily injections and vice versa. It was possible to switch treatment twice and two individuals did so in the REPOSE trial. The data in the REPOSE trial was analysed to assess the number of people with diabetes who switched treatment. The estimated cost of insulin and insulin pumps were adjusted to reflect the fact that individuals switch treatments.

1.1.2 Estimating the within trial cost effects

In the base case analysis, complete cost information was used in the EEACT. Complete total cost information was available for 98%, 90% and 92% of individuals in the intention to treat population at baseline, 1 year and 2 years respectively.

In a scenario analysis missing cost data was imputed for those individuals who attended at least one REPOSE follow up visit. Total discounted cost data was imputed using chained equations (predictive mean matching) using baseline HbA1c, treatment allocation, age at baseline, and baseline cost values as covariates in the imputation equations. 10 different imputed values were calculated for each individual with missing data.

1.1.3 Estimating within trial QALY effects using EQ-5D and SF-12

To generate quality adjusted life year (QALY) measures over the two year trial follow-up, information was collected on individual's utility using two different instruments, the EQ-5D and the SF-12. The EQ-5D and SF-12 questionnaires were completed by individuals at baseline and all follow up visits (6 months, 12 months and 24 months).

In the base case within trial analysis, the utility values measured by the EQ-5D-3L were used to calculate QALYs using an area under the curve analysis. EQ-5D utility scores were used in the base case because they are NICE's preferred utility measure.[6] In a scenario analysis, utility values measured using the SF-6D (a measure derived from the SF-12) were used to calculate QALY values.[7] In the base case, only individuals with complete QALY data were included in the analysis. Utilities as measured by EQ-5D-3L were completed by 99%, 93%, 88% and 90% of individuals at baseline, 6, 12 and 24 months respectively. If an individual had a missing 6 month utility value it was assumed that the 6 month utility value

For peer review only

would be the average of the baseline and one year utility values. If an individual had a missing utility score at12 or 24 months, then they were excluded from the base case analysis.

In a scenario analysis, multiple imputation was used to impute missing QALYs values for individuals with an assessment data for least one follow up point. Data was imputed using chained equations (predictive mean matching) using baseline HbA1c, treatment allocation, age at baseline, and baseline cost or QALY values as covariates in the imputation equations. 10 different imputed values were calculated for each individual with missing data in the analyses using imputed data

1.1.4 The statistical model used for the within trial analysis

A seemingly unrelated regression model was used to estimate the costs and QALYs in the EEACT.[8] One seemingly unrelated regression was fitted to four outcome variables: total discounted costs in year 1; total discounted costs in year 2; total discounted QALYs in year 1 and total discounted QALYs in year 2; using the mysureg command in the ml_ado package in STATA version 13.1. For the QALY outcome variables, baseline HbA1c, treatment allocation and baseline utility were included as covariates. Baseline utility was included as a covariate to estimate QALYs so that any baseline differences in health between the two treatment arms was controlled for.[9] For the cost outcome variables, baseline HbA1c, site, treatment allocation and baseline resource use were included as covariates. The standard errors of the seemingly unrelated regression was adjusted for clustering within each DAFNE course.

1.1.5 Analysis

The key measure of cost-effectiveness in the EEACT was the incremental cost-effectiveness ratio base on the mean incremental effect of CSII+DAFNE compared to MDI+DAFNE on total costs and total QALYs. The confidence intervals around these effects were estimated from the variance –covariance matrix of the regression model. The results were presented on a cost-effectiveness plane and the uncertainty around the mean effect was presented using a confidence ellipse.

2 Results

2.1 Base Case analysis

For peer review only

The results of the within trial cost-effectiveness analysis are presented using a confidence ellipse in

For peer review only

Figure **1**. In the base case CSII + DAFNE was dominated by MDI + DAFNE as CSII + DAFNE produced fewer mean QALYs at a higher mean cost. The confidence ellipses show CSII + DAFNE was associated with statistically significantly higher costs than MDI + DAFNE at the 5% significance level. The confidence ellipses also show that CSII + DAFNE was not associated with statistically significantly lower QALYs than MDI+DAFNE at the 5% significance level. This is as the confidence ellipse crosses the y axis of the graph at 0. Another point to note is that the confidence ellipses do not cross a threshold ICER of £20,000 per QALY gained; therefore the ICER of CSII + DAFNE compared to MDI + DAFNE is greater than £20,000 per QALY gained at the 95% confidence level.

The cost-effectiveness acceptability curve is presented in Figure 2. It shows that CSII + DAFNE has a 0.0% chance of being cost effective at threshold ICERs of £20,000 per QALY gained and £30,000 per QALY gained.

Table 3 presents the incremental cost and QALY outcomes of CSII + DAFNE compared to MDI + DAFNE in each year of the trial and for both years combined. In the base case the incremental cost in year 2 is lower than the cost in year 1. This is likely due to 1) treatment switching and 2) the rate of DKAs and severe hypoglycaemic events were noticeably lower in the CSII + DAFNE arm in the second year compared to the first year. The incremental QALYs are negative in the first year and positive in the second year. However in neither year is this result statistically significant and in both years, the mean effects are less than 1/100th of a QALY.

For peer review only

Figure 1: The cost-effectiveness ellipse for the base case within trial analysis

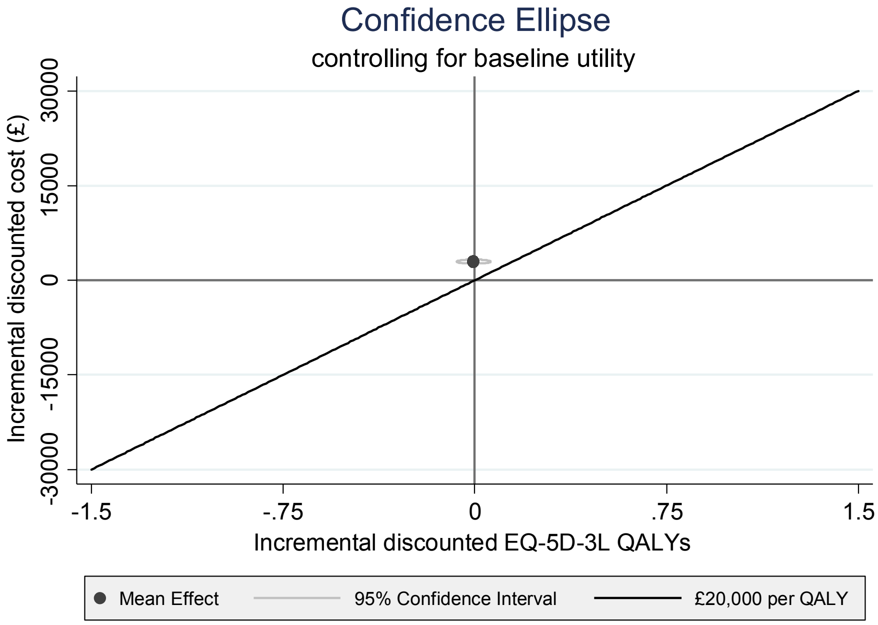

Figure 2: The cost-effectiveness acceptability curve for the base case within trial analysis

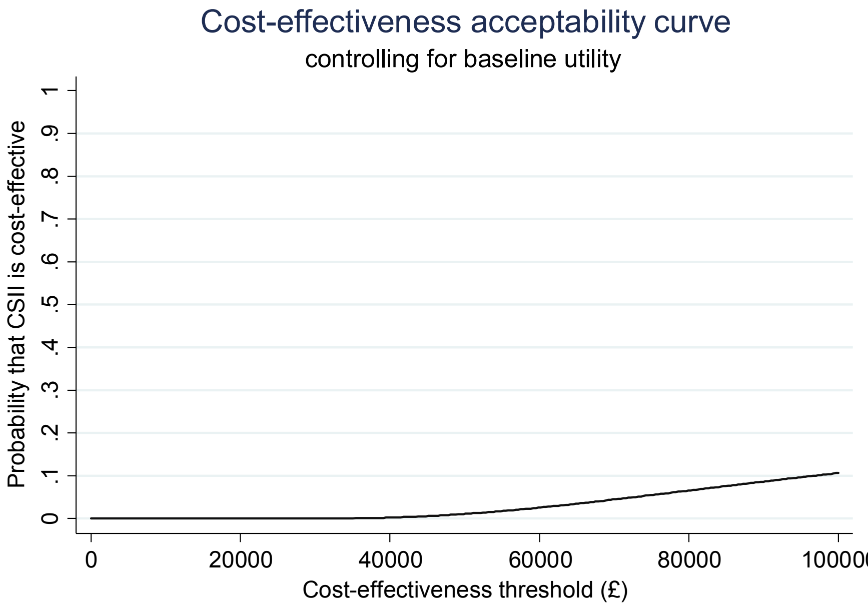

For peer review only - http://bmjopen.bmj.com/site/about/guidelines.xhtml

2.1.1 Summary of the scenario analyses

The following scenario analyses were undertaken:

1) Those individuals who adhered to their randomised method of insulin delivery
2) Missing Cost and QALY data were imputed
3) QALYs measured by SF-6D were used instead of QALYs measured using EQ-5D
4) Imputed data and QALYs measured by SF-6D QALYs
5) Pump costs measured by Riemsma et al.[10] were used
6) Riemsma et al. pump costs were used and missing data was imputed
7) The cost of pumps and consumables are 25% lower
8) The cost of pumps and consumables are 25% lower in a per protocol population
9) The cost of pumps and consumables are 50% lower
10) The cost of pumps and consumables are 50% lower in a per protocol population

For peer review only

2.1.2 Results of the scenario analyses

The results of the scenario analyses are also presented in Table 9. It is clear that CSII + DAFNE is dominated by MDI + DAFNE in all analyses, except those conducted in the per protocol population. The lowest ICER is observed in the scenario were the per protocol population is used and there is a cost reduction in insulin pumps and consumables of 50%. The ICER in this scenario is £552,866, which is above the £20,000 to £30,000 per QALY gained threshold considered by NICE.

For peer review only

Table 3: Within trial cost-effectiveness analysis results of continuous subcutaneous insulin infusion (CSII) versus multiple daily injections (MDI), both with dose adjustment for normal eating structured education

		N	Incremental costs in year 1	Incremental costs in year 2	Total incremental costs	Incremental QALYs in year 1	Incremental QALYs in year 2	Total incremental QALYs	ICER (£/QALY gained)
**Base Case** – ITT population with complete costs and QALYs	Mean<br>95% CI	205	£1732<br>(£1511, £1952)	£1228<br>(£1063, £1392)	£2959<br>(£2692, £3227)	-0.007<br>(-0.036,0.022)	0.003<br>(-0.029, 0.035)	-0.004<br>(-0.057, 0.048)	Dominated
**Scenario Analyses**									
Per protocol population	Mean<br>95% CI	188	£1780<br>(£1520, £2041)	£1434<br>(£1328, £1539)	£3214<br>(£2916, £3513)	-0.003<br>(-0.034, 0.027)	0.006<br>(-0.026, 0.037)	0.002<br>(-0.051, 0.056)	£1,369,287
Imputed data	Mean<br>95% CI	260	£1697<br>(£1492, £1901)	£1175<br>(£1006, £1345)	£2872<br>(£2602, £3142)	-0.013<br>(-0.039, 0.014)	0.004<br>(-0.029, 0.037)	-0.009<br>(-0.058, 0.04)	Dominated
SF-6D QALYs	Mean<br>95% CI	196	£1746<br>(£1514, £1978)	£1254<br>(£1096, £1412)	£3000<br>(£2729, £3271)	-0.001<br>(-0.021, 0.019)	-0.002<br>(-0.027, 0.023)	-0.003<br>(-0.045, 0.039)	Dominated
Imputed data and SF-6D QALYs	Mean<br>95% CI	256	£1701<br>(£1494, £1908)	£1186<br>(£1016, £1357)	£2888<br>(£2616, £3159)	-0.003<br>(-0.021, 0.015)	-0.001<br>(-0.024, 0.022)	-0.004<br>(-0.041, 0.034)	Dominated
Riemsma et al. pump costs	Mean<br>95% CI	205	£1679<br>(£1450, £1908)	£1184<br>(£1024, £1343)	£2863<br>(£2586, £3140)	-0.007<br>(-0.036, 0.022)	0.003<br>(-0.029, 0.035)	-0.004<br>(-0.057, 0.048)	Dominated
Imputed data Riemsma et al. pump costs	Mean<br>95% CI	260	£1648<br>(£1434, £1861)	£1125<br>(£964, £1286)	£2772<br>(£2498, £3047)	-0.013<br>(-0.039, 0.014)	0.004<br>(-0.029, 0.037)	-0.009<br>(-0.058, 0.04)	Dominated

For peer review only

Table 3: Within trial cost-effectiveness analysis results of continuous subcutaneous insulin infusion (CSII) versus multiple daily injections (MDI), both with dose adjustment for normal eating structured education (continued)

		N	Incremental costs in year 1	Incremental costs in year 2	Total incremental costs	Incremental QALYs in year 1	Incremental QALYs in year 2	Total incremental QALYs	ICER (£/QALY gained)
**Base Case** – ITT population with complete costs and QALYs	Mean 95% CI	205	£1732 (£1511, £1952)	£1228 (£1063, £1392)	£2959 (£2692, £3227)	-0.007 (-0.036,0.022)	0.003 (-0.029, 0.035)	-0.004 (-0.057, 0.048)	Dominated
**Scenario Analyses**									
The cost of pumps and consumables are 25% lower	Mean 95% CI	205	£1285 (£1022, £1547)	£955 (£850, £1059)	£2239 (£1786, £2314)	-0.007 (-0.036, 0.022)	0.003 (-0.104, 0.11)	-0.004 (-0.057, 0.048)	Dominated
The cost of pumps and consumables are 25% lower in a per protocol analysis	Mean 95% CI	188	£1223 (£1010, £1436)	£768 (£634, £902)	£1991 (£1939, £2540)	-0.003 (-0.034, 0.027)	0.006 (-0.026, 0.037)	0.002 (-0.051, 0.056)	£966,218
The cost of pumps and consumables are 50% lower	Mean 95% CI	205	£767 (£532, £1001)	£375 (£255, £494)	£1141 (£873, £1409)	-0.007 (-0.036, 0.022)	0.003 (-0.029, 0.035)	-0.004 (-0.057, 0.048)	Dominated
The cost of pumps and consumables are 50% lower in a per protocol analysis	Mean 95% CI	188	£789 (£524, £1053)	£475 (£372, £579)	£1264 (£961, £1567)	-0.004 (-0.034, 0.027)	0.006 (-0.026, 0.037)	0.002 (-0.051, 0.056)	£552,866

All costs are reported in 2013/14 prices ;ITT, intention to treat; QALY, quality adjusted life year; CI, confidence interval; ICER, incremental cost-effectiveness ratio

For peer review only

References

1. DAFNE. DAFNE Fact Sheet Six. Online Source. 2012;Available from; http://www.dafne.uk.com/uploads/135/documents/06_factsheetsix_12pt_18_06_12.pdf. Last Accessed 1st July 2015.
2. Department of Health. NHS reference costs 2013 to 2014. Online Source. 2014;Available from; https://www.gov.uk/government/publications/nhs-reference-costs-2013-to-2014. Last accessed 17th July 2015.
3. Elliott J, Jacques RM, Kruger J, Campbell MJ, Amiel SA, Mansell P et al. Substantial reductions in the number of diabetic ketoacidosis and severe hypoglycaemia episodes requiring emergency treatment lead to reduced costs after structured education in adults with Type 1 diabetes. Diabet Med. 2014;31(7):847-53. doi:10.1111/dme.12441.
4. Health & Social Care Information Centre. Prescription Cost Analysis England 2014. Online Source. 2015;Available from; http://www.hscic.gov.uk/catalogue/PUB17274. Last Accessed 2nd Novemeber 2015.
5. British Medical Association and Royal Pharmaceutical Society of Great Britain. British National Formulary August 2015. Online Source. 2015;Available from: https://www.medicinescomplete.com, Last Accessed 9th September 2015.
6. National Institute for Health and Care Excellence. Guide to the methods of technology appraisal 2013. Online Source. 2013;Available from; http://www.nice.org.uk/article/pmg9/resources/non-guidance-guide-to-the-methods-of-technology-appraisal-2013-pdf. Last accessed 13th July 2015.
7. Brazier JE, Roberts J. The estimation of a preference-based measure of health from the SF-12. Med Care. 2004;42(9):851-9.
8. Zellner A. An Efficient Method of Estimating Seemingly Unrelated Regressions and Tests for Aggregation Bias. J Am Stat Assoc. 1962;57(298):348-&.
9. Manca A, Hawkins N, Sculpher MJ. Estimating mean QALYs in trial-based cost-effectiveness analysis: the importance of controlling for baseline utility. Health Econ. 2005;14(5):487-96. doi:10.1002/hec.944.
10. Riemsma R, Corro Ramos I, Birnie R, Büyükkaramikli N, Armstrong N, Ryder S et al. Type 1 diabetes: Integrated sensor-augmented pump therapy systems for managing blood glucose levels (The MiniMed Paradigm Veo System and the Vibe and G4 PLATINUM CGM system), a systematic review and economic evaluation. Kleijnen Systematic Reviews Ltd. 2015.

For peer review only

Table of Contents

1 Other cause mortality 1

2 The clinical effectiveness parameters 1

2.1 Treatment switching 1

2.2 HbA1c 9

2.3 DKA and severe hypoglycaemia 13

3 The cost of insulin, diabetes related contacts and insulin pumps used in the Sheffield Type 1 Diabetes model 17

4 Detailed results of the scenario and subgroup analyses 21

List of Tables

Table 1: The results of the parametric survival models fitted to patients allocated to the continuous subcutaneous insulin infusion dose adjustment for normal eating arm of the REPOSE trial 2
Table 2: The results of the parametric survival models fitted to patients allocated to the multiple daily injections with dose adjustment for normal eating arm of the REPOSE trial 4
Table 3: A summary of the Akaike information criterion and the Bayesian information criterion for the fitted survival curves used in the long term modelling 5
Table 4: An embedded Excel Worksheet containing the variance-covariance matrices for the different survival curves fitted to the REPOSE trial data (both arms) 8
Table 5: The effect of continuous subcutaneous insulin infusion compared to multiple daily injections for all individuals in the intention to treat population. 10
Table 6: The effect of continuous subcutaneous insulin infusion compared to multiple daily injections for all individuals in the per protocol population. 11
Table 7: An embedded excel worksheet containing the variance-covariance matrix for the beta regressions on HbA1c. 12
Table 8: A summary of the observed incidence of diabetic ketoacidosis and severe hypoglycaemia in the intention to treat population 14
Table 9: The negative binomial model fitted to the incidence of severe hypoglycaemia at baseline, one year and two years 16
Table 10: The negative binomial model fitted to the incidence of diabetic ketoacidosis at baseline, one year and two years 16
Table 11: The unit costs of insulin 17
Table 12: An embedded Excel Worksheet containing the variance covariance matrices for the cost regressions 20
Table 13: The One way scenario analyses and subgroup analyses performed using the Sheffield Type 1 Diabetes Policy Model 21

List of Figures

Figure 1: A visual plot of the Kaplan-Meier and parametric survival curves for those individuals who were randomised continuous subcutaneous insulin infusion with dose adjustment for normal eating ... 6
Figure 2: A visual plot of the Kaplan-Meier and parametric survival curves for those individuals who were randomised multiple daily injections with dose adjustment for normal eating 7

For peer review only - http://bmjopen.bmj.com/site/about/guidelines.xhtml

1 Other cause mortality

Individuals can also die from other causes. This other-cause mortality was updated in version of the model used for these analyses. Other-cause mortality was calculated using UK life tables from 2012 to 2014 adjusted to exclude the causes either attributed to diabetes mellitus (either type 1, type 2 or unspecified, ICD-10 codes E10-14) or modelled directly in the microvascular and macrovascular disease components (deaths due to: end stage renal disease; myocardial infarction, stroke and heart failure, ICD-10 codes N18, I20-21, I61-64, I50).[1-4]

2 Probability of death from end stage renal disease

This model parameter was altered from the value reported in Heller *et al*.[5] to reflect directly observable data available in Wolfe *et al*.[6] on the probability of end stage renal disease. At baseline, 102,163 patients with diabetes were receiving dialysis and over a maximum follow up of 6 years 44,916 of these patients died. This gave a probability of death from ESRD of 44.00% over 6 years. In the model probabilistic sensitivity analysis, the uncertainty in this probability was parameterised using a beta distribution with an alpha parameter of 44,916 and a beta parameter of 57,247. The probability of death from end stage renal disease over 6 years was first converted into an instantaneous rate of death and then yearly probability of death from end stage renal disease using the method in Briggs et al.[7]

3 The clinical effectiveness parameters

3.1 Treatment switching

The treatment switching curves were used to estimate the incidence of treatment switching in the model in the first and second year. Covariates were used in the parametric models to control for: HbA1c prior to switching, number of DKAs and number of severe hypoglycaemic events in the year prior to switching (or 2 years follow up if no switching occurred). The standard errors of the parametric survival models were adjusted for clustering in each DAFNE course. As separate models were fitted to the continuous subcutaneous insulin infusion + dose adjustment for normal eating (CSII+DAFNE) and the multiple daily injections + dose adjustment for normal eating (MDI+DAFNE) arm, no assumption of proportional hazards or accelerated failure time was made.

The curves were not extrapolated, as the clinical expert opinion of a Professor of Clinical Diabetes & Honorary Consultant Physician and a Professor in Public Health & Health Technology Assessment was that if an individual was still using CSII after two years that they would continue to use CSII as in their experience once an adult with type 1 diabetes mellitus (T1DM) was successfully using an insulin pump they were unlikely change their insulin delivery method.

The different parameters of the parametric survival models fitted to the continuous subcutaneous insulin infusion + dose adjustment for normal eating (CSII+DAFNE) arm is given in Table 1. The equivalent parameters are given for the multiple daily injections + dose adjustment for normal eating arm in Table 2.

The goodness of fit of the parametric survival curves were assessed using the Akaike information criterion (AIC), Bayesian information criterion (BIC) and the goodness of fit to the underlying Kaplan-Meier curve at one and two years. The one and two-year time points are the time points of relevance for assessing the visual fit of the curves in this analysis, as the model uses a yearly time cycle so treatment switching is only predicted in the model at these time points. The AIC and the BIC

For peer review only - http://bmjopen.bmj.com/site/about/guidelines.xhtml

are given in Table 3. Lower values for these statistics indicate a better model fit. For the CSII+DAFNE arm, the exponential model has the lowest AIC and BIC. For the MDI+DAFNE arm, the Weibull model had the lowest AIC and the exponential model has the lowest BIC. The plot of the parametric survival curves against the underlying Kaplan-Meier curve are provided in Figures 1 and 2. It is clear that the exponential model provides a reasonable fit to the Kaplan-Meier for the CSII+DAFNE arm at the one and two-year time points therefore this curve was used in the base case economic model. It is also clear that for the MDI+DAFNE arm, the exponential curve does not provide a reasonable fit to the Kaplan –Meier curve at one year whereas the Weibull curve provides a reasonable fit at both one and two years.

The uncertainty in the parametric survival curves was included in the model's probabilistic sensitivity analysis using a multivariate normal distribution. The variance-covariance matrices and the predicted coefficients for each of the parametric survival models were used to parameterise the multivariate normal distributions. The coefficients are given in table 1 and 2 respectively and the variance – covariance matrices for each of the parametric curves is given in table 4 as an embedded Excel workbook.

Table 1: The results of the parametric survival models fitted to patients allocated to the continuous subcutaneous insulin infusion dose adjustment for normal eating arm of the REPOSE trial

Parameter	Coefficient	Robust standard error	95% confidence interval	
Exponential model				
HbA1c	0.222	0.241	-0.251	0.695
Number of DKAs	-0.972	0.474	-1.901	-0.042
Number of severe hypoglycaemic events	0.427	0.087	0.257	0.598
Constant	-4.616	2.125	-8.781	-0.451
Weibull model				
HbA1c	0.221	0.234	0.016	0.694
Number of DKAs	-0.981	0.471	-7.113	-4.910
Number of severe hypoglycaemic events	0.404	0.085	0.337	0.684
Constant	-4.460	2.100	-10.607	-4.696
ln scale parameter	-0.258	0.220	0.111	1.377
Gompertz model				
HbA1c	0.220	0.236	-0.243	0.684
Number of DKAs	-0.983	0.468	-1.901	-0.065
Number of severe hypoglycaemic events	0.407	0.090	0.230	0.584
Constant	-4.307	2.232	-8.682	0.068
Gamma parameter	-0.316	0.479	-1.256	0.624
Log logistic model				
HbA1c	-0.294	0.286	-0.855	0.267
Number of DKAs	1.406	0.676	0.081	2.730
Number of severe hypoglycaemic events	-0.554	0.170	-0.887	-0.220
Constant	5.637	2.510	0.718	10.557
ln gamma parameter	0.215	0.230	-0.235	0.665
Log normal model				

For peer review only - http://bmjopen.bmj.com/site/about/guidelines.xhtml

HbA1c	-0.307	0.292	-0.879	0.264
Number of DKAs	1.867	0.755	0.387	3.347
Number of severe hypoglycaemic events	-0.656	0.180	-1.009	-0.304
Constant	6.406	2.520	1.466	11.346
ln sigma parameter	1.002	0.206	0.598	1.405

DKA, diabetic ketoacidosis; ln, natural logarithm

For peer review only

For peer review only - http://bmjopen.bmj.com/site/about/guidelines.xhtml

Table 2: The results of the parametric survival models fitted to patients allocated to the multiple daily injections with dose adjustment for normal eating arm of the REPOSE trial

Parameter	Coefficient	Robust standard error	95% confidence interval	
Exponential model				
HbA1c	0.336	0.164	0.014	0.657
Number of DKAs	-5.555	0.561	-6.655	-4.455
Number of severe hypoglycaemic events	0.460	0.074	0.315	0.605
Constant	-6.725	1.450	-9.567	-3.884
Weibull model				
HbA1c	0.355	0.173	0.016	0.694
Number of DKAs	-6.012	0.562	-7.113	-4.910
Number of severe hypoglycaemic events	0.510	0.089	0.337	0.684
Constant	-7.652	1.508	-10.607	-4.696
ln scale parameter	0.744	0.323	0.111	1.377
Gompertz model				
HbA1c	0.350	0.170	0.016	0.683
Number of DKAs	-6.009	0.562	-7.110	-4.908
Number of severe hypoglycaemic events	0.512	0.094	0.329	0.696
Constant	-8.080	1.471	-10.963	-5.197
Gamma parameter	1.055	0.669	-0.256	2.366
Log logistic model				
HbA1c	-0.181	0.121	-0.418	0.055
Number of DKAs	2.609	0.799	1.044	4.175
Number of severe hypoglycaemic events	-0.232	0.070	-0.368	-0.095
Constant	3.676	1.317	1.094	6.258
ln gamma parameter	-0.780	0.317	-1.401	-0.160
Log normal model				
HbA1c	-0.190	0.107	-0.400	0.021
Number of DKAs	1.617	0.517	0.603	2.630
Number of severe hypoglycaemic events	-0.283	0.101	-0.481	-0.086
Constant	4.117	1.291	1.587	6.647
ln sigma parameter	0.066	0.338	-0.596	0.728

DKA, diabetic ketoacidosis; ln, natural logarithm

For peer review only

Table 3: A summary of the Akaike information criterion and the Bayesian information criterion for the fitted survival curves used in the long term modelling

Parametric survival model	AIC	BIC
CSII +DAFNE		
Exponential	145.77	157.24
Weibull	146.46	160.80
Gompertz	147.25	161.59
Log logistic	147.49	161.83
Log normal	148.48	162.82
MDI + DAFNE		
Exponential	64.36	75.77
Weibull	62.55	76.81
Gompertz	63.76	78.02
Log logistic	63.78	78.04
Log normal	64.97	79.23

CSII– continuous subcutaneous insulin infusion; DAFNE – dose adjustment for normal eating; MDI – multiple daily injections; AIC - Akaike information criterion; BIC - Bayesian information criterion

For peer review only

For peer review only - http://bmjopen.bmj.com/site/about/guidelines.xhtml

Figure 1: A visual plot of the Kaplan-Meier and parametric survival curves for those individuals who were randomised continuous subcutaneous insulin infusion with dose adjustment for normal eating

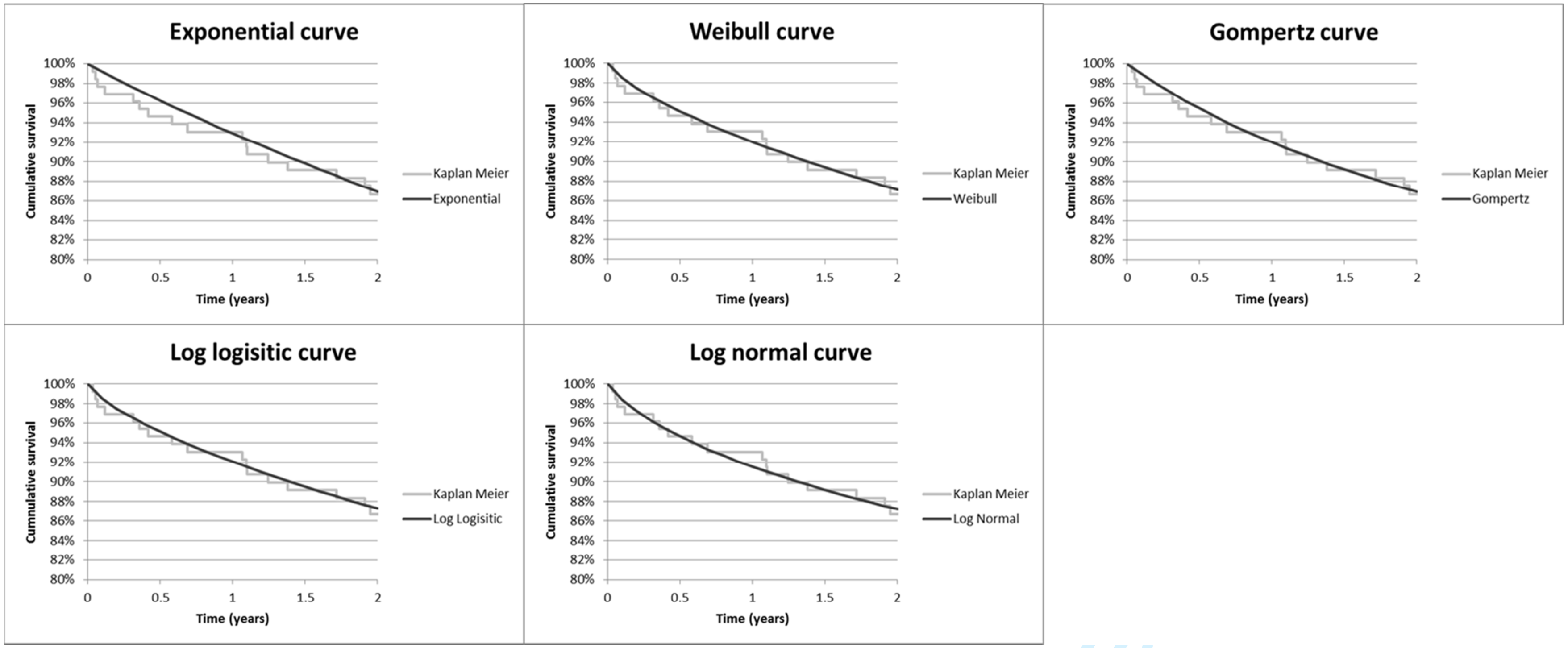

Figure 2: A visual plot of the Kaplan-Meier and parametric survival curves for those individuals who were randomised multiple daily injections with dose adjustment for normal eating

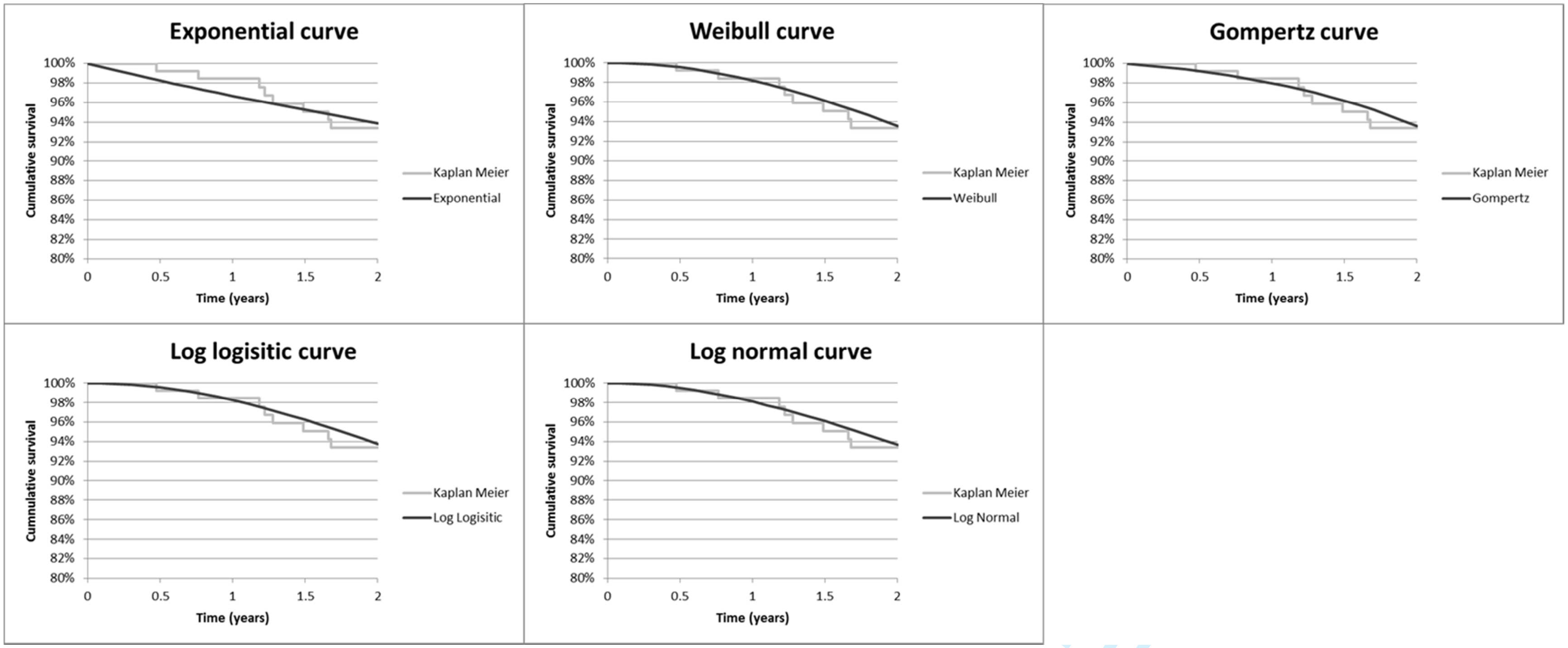

7

Table 4: An embedded Excel Worksheet containing the variance-covariance matrices for the different survival curves fitted to the REPOSE trial data (both arms)

Treatment switching

Exponential model - CSII

	HbA1c	Number of	Number of	Constant
HbA1c	0.05816			
Number of	0.064451	0.224993		
Number of	0.001201	-0.00232	0.007566	
Constant	-0.50842	-0.56159	-0.02293	4.516119

Exponential model - MDI

	HbA1c	Number of	Number of	Constant

For peer review only

3.2 HbA1c

Two beta regressions were used to estimate each individual's HbA1c in the first and second year respectively in the model. Bounds were placed on the beta regression so that HbA1c was between the clinically plausible bounds of 29mmol/mol [4.8%] and 201 mmol/mol [20.5%]. Beta regressions estimate two parameters of interest, the mean effect and the dispersion in the mean. Treatment allocation, baseline HbA1c and centre were included as covariates for the mean effect on HbA1c after one year. Treatment allocation, baseline HbA1c, one year HbA1c and centre were included as covariates for the mean effect on HbA1c after two years. HbA1c in the previous year was used as a covariate for the dispersion parameters. The standard errors of both statistical models were adjusted for clustering within each DAFNE course. Due to presence of treatment switching, an individual's HbA1c was assumed to change as thought they had been allocated to the other trial arm. Therefore, the beta regressions were estimated in the per protocol population (switchers excluded) rather than the ITT population (switchers included in their randomised arm). A sensitivity analysis was conducted in which the beta regressions were estimated in the ITT population.

The results of the beta regression in the per protocol population is given in Table 5 and the beta regression estimated in the ITT population is given in Table 6. The results of these beta regressions are not easily interpretable as changes in HbA1c, as a logit link function is used to estimate the mean effect parameter and the natural logarithm of the dispersion parameter is estimated instead of the dispersion parameter itself.

Missing data was observed for HbA1c values in the per protocol population at 6 months (2.1% missing), 1 year (4.2% missing) and 2 years (4.2% missing). A multiple imputation procedure was employed in individuals with at least one HbA1c value (at 6 or 12 months) after randomisation but no HbA1c value at 24 months. In line with the statistical analysis plan, missing 24 month HbA1c data was imputed by multiple imputation using chained equations (regression) based on 10 imputed data sets with baseline, 6 and 12 months HbA1c measurements, DAFNE course, centre, age, sex, and HFS worry as covariates, if a participant had some HbA1c follow-up data. This imputation procedure was conducted in the ITT population, prior to running the beta regressions. After imputation 236 out of 236 participants in the per protocol population and 259 out of 260 participants in the ITT population had HbA1c follow up data.

The uncertainty in the parametric survival curves was included in the model's probabilistic sensitivity analysis using a multivariate normal distribution. The variance-covariance matrices and the predicted coefficients for each of the beta regressions were used to parameterise the multivariate normal distributions. The coefficients are given in Table 5 and 6 respectively and the variance – covariance matrices for each of the parametric curves is given in table 4 as an embedded Excel workbook.

Each individual's mean effect parameter and dispersion parameter were used to calculate the expected variance in that individual's actual HbA1c outcome. The individual's predicted mean effect and variance in their mean effect were used to parameterise a beta distribution. Each individual's HbA1c in the model was sampled from the beta distribution which was parameterised by their individualised mean effect and variance. This random draw was then transformed onto the DCCT (%) scale for use in the risk equations of the model.

For peer review only - http://bmjopen.bmj.com/site/about/guidelines.xhtml

Table 5: The effect of continuous subcutaneous insulin infusion compared to multiple daily injections for all individuals in the intention to treat population.

Hba1c at one year (beta scale)	Coefficient	Standard error	t	P>t	95% Confidence interval	
Mean effect (Mu)						
Treatment allocation (1= CSII + DAFNE, 0=MDI + DAFNE)	-0.056	0.038	-1.49	0.137	-0.131	0.018
Baseline HbA1c (beta scale)	3.978	0.248	16.01	0	3.491	4.465
Constant	-2.223	0.088	-25.28	0	-2.395	-2.050
Centre effects (Cambridge is the reference site)						
Dumfries and Galloway	-0.025	0.074	-0.33	0.738	-0.171	0.121
Edinburgh	-0.019	0.065	-0.3	0.768	-0.147	0.108
Glasgow	-0.154	0.099	-1.55	0.12	-0.348	0.040
Harrogate	0.022	0.041	0.52	0.602	-0.060	0.103
College Hospital - London	0.013	0.065	0.21	0.837	-0.114	0.140
Nottingham	0.214	0.060	3.58	0	0.097	0.331
Sheffield	0.066	0.057	1.17	0.241	-0.045	0.178
Natural logarithm of the dispersion parameter (ln(phi))						
Baseline HbA1c (beta scale)	-2.996862	0.9980645	-3	0.003	-4.954	-1.040
Constant	4.912	0.332	14.79	0	4.261	5.563
Hba1c at two years (beta scale)						
Mean effect (Mu)						
Treatment allocation(1= CSII + DAFNE, 0=MDI + DAFNE)	-0.018	0.035	-0.52	0.603	-0.086	0.050
One year HbA1c (beta scale)	0.797	0.318	2.51	0.012	0.175	1.419
Baseline HbA1c (beta scale)	3.599	0.342	10.51	0	2.927	4.271
Constant	-2.380	0.091	-26.14	0	-2.558	-2.201
Centre effects (Cambridge is the reference site)						
Dumfries and Galloway	0.047	0.093	0.5	0.617	-0.137	0.230
Edinburgh	0.067	0.085	0.8	0.426	-0.098	0.233
Glasgow	0.137	0.097	1.42	0.155	-0.052	0.327
Harrogate	0.123	0.087	1.41	0.158	-0.048	0.294
College Hospital - London	0.079	0.087	0.9	0.366	-0.092	0.249
Nottingham	0.120	0.110	1.09	0.279	-0.098	0.337
Sheffield	0.156	0.080	1.96	0.05	0.000	0.312
Natural logarithm of the dispersion parameter (ln(phi))						
One year HbA1c (beta scale)	-4.667	1.129	-4.13	0	-6.881	-2.453
Constant	5.422	0.277	19.56	0	4.879	5.966

CSII, continuous subcutaneous insulin infusion; MDI, multiple daily injections; DAFNE, dose adjustment for normal eating

For peer review only - http://bmjopen.bmj.com/site/about/guidelines.xhtml

Table 6: The effect of continuous subcutaneous insulin infusion compared to multiple daily injections for all individuals in the per protocol population.

Hba1c at one year (beta scale)	Coefficient	Standard error	t	P>t	95% Confidence interval	
Mu						
Treatment allocation (1= CSII + DAFNE, 0=MDI + DAFNE)	-0.056	0.044	-1.37	0.171	-0.148	0.026
Baseline HbA1c (beta scale)	3.938	0.255	13.62	0	2.978	3.980
Constant	-2.219	0.093	-23.94	0	-2.401	-2.038
Centre effects (Cambridge is the reference site)						
Dumfries and Galloway	-0.019	0.078	-0.25	0.805	-0.172	0.134
Edinburgh	0.020	0.056	0.37	0.714	-0.089	0.130
Glasgow	-0.129	0.095	-1.36	0.175	-0.315	0.057
Harrogate	0.025	0.040	0.62	0.534	-0.054	0.104
College Hospital - London	0.018	0.064	0.28	0.779	-0.107	0.143
Nottingham	0.172	0.039	4.46	0	0.096	0.247
Sheffield	0.084	0.064	1.31	0.191	-0.042	0.209
Natural logarithm of phi						
Baseline HbA1c (beta scale)	-3.504	1.050	-3.34	0.001	-5.563	-1.446
Constant	5.062	0.351	14.41	0	4.373	5.751
Hba1c at two years (beta scale)						
Mu						
Treatment allocation (1= CSII + DAFNE, 0=MDI + DAFNE)	-0.047	0.035	-1.35	0.177	-0.116	0.021
One year HbA1c (beta scale)	3.475	0.340	10.23	0	2.809	4.141
Baseline HbA1c (beta scale)	1.053	0.351	3	0.003	0.365	1.740
Constant	-2.382	0.092	-26.01	0	-2.562	-2.203
Centre effects (Cambridge is the reference site)						
Dumfries and Galloway	0.022	0.088	0.26	0.799	-0.150	0.194
Edinburgh	0.076	0.085	0.89	0.374	-0.091	0.243
Glasgow	0.105	0.096	1.1	0.271	-0.082	0.293
Harrogate	0.092	0.085	1.08	0.28	-0.075	0.258
College Hospital - London	0.053	0.085	0.62	0.538	-0.115	0.220
Nottingham	0.109	0.100	1.1	0.276	-0.089	0.308
Sheffield	0.157	0.078	2.02	0.043	0.005	0.310
Natural logarithm of phi						
One year HbA1c (beta scale)	-4.809	1.231	-3.9	0	-7.223	-2.394
Constant	5.474	0.302	18.13	0	4.882	6.066

CSII, continuous subcutaneous insulin infusion; MDI, multiple daily injections

For peer review only - http://bmjopen.bmj.com/site/about/guidelines.xhtml

Table 7: An embedded excel worksheet containing the variance-covariance matrix for the beta regressions on HbA1c.

	King's Colle	-0.00011	-0.00188	0.002045	0.005957	0.005836
	Nottinghan	-0.00021	-0.00561	-0.00384	0.006185	0.005762
	Sheffield	-0.00046	-0.00459	0.000829	0.005942	0.005672
	Constant	-0.00027	-0.00427	-0.01022	-0.00538	-0.00497
Ln Phi	Baseline Hb	-0.00235	-0.01365	-0.08491	-0.02003	-0.00635
	Constant	7.28E-05	0.010746	0.019731	0.005442	0.002026

For peer review only

For peer review only - http://bmjopen.bmj.com/site/about/guidelines.xhtml

3.3 DKA and severe hypoglycaemia

To develop the method to incorporate severe hypoglycaemic events and Diabetic Ketoacidosis (DKA) treatment effect evidence into the model, several factors were considered. Data on severe hypoglycaemic events and Diabetic Ketoacidosis (DKA) were collected on an ongoing basis throughout the trial. Self-reported information was also collected on the incidence of DKA. A summary of the numbers of DKAs and severe hypoglycaemic events is given in Table 9. It can be seen that the number of DKAs and severe hypoglycaemic events declines in the second year on every measure, except self-reported DKAs in the MDI+DAFNE arm were the number events was the same in both years. As such, the statistical models used in the economic data estimated the incidence of severe hypoglycaemia and DKA in the first and second years separately.

For peer review only

Table 8: A summary of the observed incidence of diabetic ketoacidosis and severe hypoglycaemia in the intention to treat population

	Year 1			Year 2			Total		
	CSII + DAFNE (n=132)	MDI + DAFNE (n=128)	Total (n=260)	CSII + DAFNE (n=132)	MDI + DAFNE (n=128)	Total (n=260)	CSII + DAFNE (n=132)	MDI + DAFNE (n=128)	Total (n=260)
DKAs - Serious Adverse events									
Number(%) participants with ≥1 DKA	15(11.4%)	1(0.8%)	16 (6.2%)	4(3.0%)	2(1.5%)	6(2.2%)	17(12.9%)	3(2.3%%)	20(7.7%)
Number of hospital admissions	16	5	21	5	4	9	21	9	30
DKAs - Self-reported admissions									
Number(%) participants with ≥1 DKA	17 (12.9%)	6(4.7%)	23 (8.8%)	6(4.5%)	5(3.7%)	11(4.1%)	18 (13.6%)	8(6.3%)	26(10.0%)
Number of self-reported DKAs	24	11	35	7	11	18	26*	13*	39*
Severe hypoglycaemia									
Number(%) participants with ≥1 severe hypo	10(7.6%)	9(7.0%)	19(7.3%)	4(3.0%)	7(5.5%)	11(4.2%)	14(10.6%)	11(8.6%)	25(9.6%)
Number of severe hypoglycaemic events	21	12	33	4	12	16	25	24	49

CSII, continuous subcutaneous insulin infusion; DAFNE, dose adjustment for normal eating; MDI, multiple daily injections; DKA, diabetic ketoacidosis; * these values are not the sums of the one and two year follow up as some individuals had missing information in either the first or second year.

For peer review only - http://bmjopen.bmj.com/site/about/guidelines.xhtml

Negative binomial regressions were used to predict the number of DKAs, and severe hypoglycaemic events in years 1 and 2 for each outcome separately. When the outcome variable was the number of severe hypos in year 1, year 1 HbA1c and treatment group were included as covariates. When the outcome variable was the number of severe hypos in year 2, year 2 HbA1c and treatment group were included as covariates When the outcome variable was the number of DKAs in year 1, year 1 HbA1c and treatment group were included as covariates When the outcome variable was the number of DKAs in year 2, year 2 HbA1c and treatment group were included as covariates. The possibility of using the number of events in the previous year, baseline events for the 1 year outcomes and year 1 events for the 2 year outcomes, as a covariate was explored. However, due to the low number of events, the negative binomial models often did not converge when this was included as a covariate.

The statistical models did not converge for DKAs reported as serious adverse events in the first year. This was not the case for self-reported DKAs and there were more self-reported cases of DKA than were picked up through the reporting of serious adverse events. Therefore, the rates of DKA were estimated using self-reported DKAs as the outcome measure.

The statistical models were fitted using the Zellig package in R version3.2.0 and using specifications described above; it was used to simulate the predicted number of severe hypoglycaemia and DKA events in each trial arm 10,000 times. The simulations were separately in each trial arm and for HbA1c values every 0.1% between 4% and 20.5%. The number of events observed in the simulations was truncated at 20 events per year to reduce the effect of extreme values in the simulation on the cost-effectiveness results. These simulations were then used to determine the probability that an individual would suffer a given number of severe hypoglycaemic events and DKA events in a year, dependent on their HbA1c that year and the trial arm they were allocated to. The probability that an individual would suffer a given number of events was a fixed parameter in the PSA, therefore any differences in the rates of DKA or severe hypoglycaemia for an individual between any two model runs will solely be due to differences in their HbA1c.

The results of the negative binomial regressions are given in Table 10 and 11.

For peer review only - http://bmjopen.bmj.com/site/about/guidelines.xhtml

Table 9: The negative binomial model fitted to the incidence of severe hypoglycaemia at baseline, one year and two years

	Coefficient	Standard error	z value	P>z
Severe hypoglycaemia in year 1				
Treatment allocation (1= CSII + DAFNE, 0=MDI + DAFNE)	0.2861	0.5149	0.556	0.578
One year HbA1c (DCCT % scale)	-0.5010	0.2323	2.157	0.03
Number of severe hypoglycaemic events experienced in the year prior to baseline	2.0708	0.5638	3.673	>0.000
Constant	1.2689	1.8676	0.679	0.49687
Severe hypoglycaemia in year 2				
Treatment allocation (1= CSII + DAFNE, 0=MDI + DAFNE)	-1.1141	0.7202	-1.547	0.122
Two year HbA1c (DCCT % scale)	-0.2019	0.2668	-0.757	0.449
Constant	-0.6367	2.2625	-0.281	0.778

CSII, continuous subcutaneous insulin infusion, DAFNE, dose adjustment for normal eating, MDI, multiple daily injections

Table 10: The negative binomial model fitted to the incidence of diabetic ketoacidosis at baseline, one year and two years

	Coefficient	Standard error	z value	P>z
Diabetic ketoacidosis in year 1				
Treatment allocation (1= CSII + DAFNE, 0=MDI + DAFNE)	0.3369	0.4786	0.704	0.481
One year HbA1c (DCCT % scale)	0.4089	0.1246	3.283	0.001
Constant	-5.9443	1.1879	-5.004	>0.00
Diabetic ketoacidosis in year 2				
Treatment allocation (1= CSII + DAFNE, 0=MDI + DAFNE)	-0.07564	0.70426	-0.107	0.914
Two year HbA1c (DCCT % scale)	0.32667	0.19447	1.680	0.093
Number of DKAs in year 1	0.86618	0.51682	1.676	0.094
Constant	-5.98206	1.82156	-3.284	0.01

CSII, continuous subcutaneous insulin infusion, DAFNE, dose adjustment for normal eating, MDI, multiple daily injections; DKA, diabetic ketoacidosis

For peer review only

For peer review only - http://bmjopen.bmj.com/site/about/guidelines.xhtml

4 The cost of insulin, diabetes related contacts and insulin pumps used in the Sheffield Type 1 Diabetes model

Self-reported information was collected on individual's use of insulin, face to face contacts related to their diabetes with a health care professional and telephone contacts related to their diabetes with a health care professional. Data collected on insulin use included, the type of insulin used, the dose of insulin and the method of insulin delivery. Ongoing information was also collected on whether an individual switched their insulin delivery mechanism (either CSII or MDI). The cost of the diabetes related contacts were sourced from the NHS reference costs and were £105.49 for face to face contacts and £75.80 for telephone contacts.[8] Insulin costs were microcosted using the data in the british national formularly and a prescription costs analysis, the full list of unit costs is given in Table X. [9 10] The unit costs of the pumps and their related consumables was obtained from a survey of the prices paid at the REPOSE trial sites.

Table 11: The unit costs of insulin

Item	Average unit cost	Number of units	Cost per unit	Associated yearly cost of an insulin pen	Source
Consumables related to multiple daily injections					
Cost of an insulin needle	£0.11	N/A	N/A	N/A	HSCIC[9]
Cost of an insulin syringe	£0.13	N/A	N/A	N/A	HSCIC[9]
Quick Acting Insulin					
**Human Insulin**					
Vial	£9.87	1000	£0.01	N/A	BNF[10], HSCIC[9]
Cartridges for a reusable pen	£18.97	1500	£0.01	£8.78	
**Animal Insulin**					
Vial	£26.15	1000	£0.03	N/A	BNF[10], HSCIC[9]
Cartridges for a reusable pen	£38.29	1500	£0.03	£5.97	
**Insulin Aspart (NovoRapid)**					
Vial	£14.08	1000	£0.01	N/A	BNF[10], HSCIC[9]
Cartridges for a reusable pen	£28.31	1500	£0.02	£9.59	
Disposable Pen	£30.63	1500	£0.02	N/A	
**Insulin Lispro (Humalog)**					
Vial	£16.61	1000	£0.02	N/A	
Cartridges for a reusable pen	£28.31	1500	£0.02	£8.86	BNF[10], HSCIC[9]
Disposable Pen	£28.31	1500	£0.02	N/A	
**Insulin Glulisine (Apidra)**					
Vial	£16.00	1000	£0.02	N/A	
Cartridges for a reusable pen	£28.30	1500	£0.02	£7.86	BNF[10], HSCIC[9]
Disposable Pen	£28.30	1500	£0.02	N/A	

For peer review only - http://bmjopen.bmj.com/site/about/guidelines.xhtml

Table 11: the unit costs of insulin (continued)

Item	Average unit cost	Number of units	Cost per unit	Associated yearly cost of an insulin pen	Source
			Background Insulin		
**Human Insulin**					
Vial	£10.41	988	£0.01	N/A	
Cartridges for a reusable pen	£21.52	1500	£0.01	£9.30	BNF[10], HSCIC[9]
Disposable Pen	£21.05	1500	£0.01	N/A	
**Animal Insulin**					
Vial	£26.17	1000	£0.03	N/A	BNF[10], HSCIC[9]
Cartridges for a reusable pen	£38.32	1500	£0.03	£9.57	
**Insulin Detemir (Levemir)**					
Cartridges for a reusable pen	£42.00	1500	£0.03	£9.59	BNF[10], HSCIC[9]
Disposable Pen	£42.10	1500	£0.03	N/A	
**Insulin Glargine (Lantus)**					
Vial	£30.68	1000	£0.03	N/A	
Cartridges for a reusable pen	£41.50	1500	£0.03	£7.86	BNF[10], HSCIC[9]
Disposable Pen	£41.50	1500	£0.03	N/A	
Mixed Insulin					
**Biphasic Isophane Insulin**					
**Animal Insulin**					
Vial	£25.20	1000	£0.03	N/A	BNF[10], HSCIC[9]
Cartridges for a reusable pen	£37.80	1500	£0.03	£5.97	
**Human Insulin**					
Vial	£15.43	987	£0.02	N/A	BNF[10], HSCIC[9]
Cartridges for a reusable pen	£18.94	1500	£0.01	£7.74	
Disposable Pen	£21.43	1500	£0.01	N/A	
**Biphasic Insulin Aspart**					
Cartridges for a reusable pen	£28.79	£28.79	£0.02	£9.59	BNF[10], HSCIC[9]
Disposable Pen	£29.89	£29.89	£0.02		
**Biphasic Insulin Lispro**					
Vial	£16.61	1000	£0.02		
Cartridge for reusable pen	£29.03	1500	£0.02	£8.93	BNF[10], HSCIC[9]
Disposable Pen	£30.13	1500	£0.02		

BNF, British National Formulary; HSCIC, Health & Social Care Information Centre

For peer review only

The cost of insulin, diabetes related contacts and insulin pumps (including consumables) for insulin pump therapy individuals were based on resource use data from the REPOSE trial data. It is expected that the covariates which predict the cost of insulin in year 1 may be correlated with the covariates which predict the cost of insulin in year 2. It is also expected that this may be true for the cost of diabetes related contacts and the cost of insulin pumps (including consumables). Therefore, instead of fitting six independent regression models, three seemingly unrelated regressions were fitted (one seemingly unrelated regression for the cost of insulin, another for the cost of diabetes related contacts and finally one for the cost of insulin pumps (including consumables).

In the cost insulin seemingly unrelated regression model, the cost of insulin in year 1 and the cost of insulin in year 2 were used as the outcome variables for the seemingly unrelated regression model. Baseline cost of insulin, baseline HbA1c, treatment allocation, whether the individual switched from multiple daily injections to insulin pump infusion in year one and whether or not the individual switched from insulin pump infusion to multiple daily injections in year 1 were included as covariates to predict the cost of insulin in year 1. Baseline cost of insulin, baseline HbA1c, the actual method of insulin delivery that an individual was using at the end of the first year, whether the individual switched from multiple daily injections to insulin pump infusion in year two and whether or not the individual switched from insulin pump infusion to multiple daily injections in year two were included as covariates to predict the cost of insulin in year two. The standard errors were adjusted for clustering in each DAFNE course.

In the cost of diabetes related contacts seemingly unrelated regression model, the cost of diabetes related contacts in year 1 and the cost of diabetes related contacts in year 2 were used as the outcome variables for the seemingly unrelated regression model. Baseline cost of diabetes related contacts, baseline HbA1c, and treatment allocation; whether the individual switched from multiple daily injections to insulin pump infusion in year one and whether or not the individual switched from insulin pump infusion to multiple daily injections in year 1 were included as covariates to predict the cost of insulin in year 1. Baseline cost of diabetes related contacts, baseline HbA1c, the actual method of insulin delivery that an individual was using at the end of the first year, whether the individual switched from multiple daily injections to insulin pump infusion in year two and whether or not the individual switched from insulin pump infusion to multiple daily injections in year two were included as covariates to predict the cost of insulin in year two. The standard errors were adjusted for clustering in each DAFNE course.

In the cost of insulin pump seemingly unrelated regression model, the cost of insulin pumps and consumables in year 1 and the cost insulin pumps and consumables in year 2 were the two outcome variables used in the model. No control was made for baseline resource use or baseline HbA1c for either outcome variable, as no individual in the REPOSE trial had previous history of using an insulin pump. Individual's randomised treatment arm, whether or not they switched from CSII to MDI in the first year and whether or not they switched from MDI to CSII in the first year were included as covariates to predict the cost of insulin pumps and consumables in year 1. Individual's actual treatment at the end of the first year, whether or not they switched from CSII to MDI in year 2 and whether or not they switched from MDI to CSII in year 2 were included as covariates to predict the cost of insulin pumps and consumables in year 2.

The results of the regressions are given in main text (Table 2)/ The uncertainty in the costs estimated by the seeming unrelated regressions were included in the model's PSA using a probabilistic

For peer review only - http://bmjopen.bmj.com/site/about/guidelines.xhtml

sensitivity analysis. The variance covariance matrices used in to parameterise the uncertainty in the cost parameters is given in Table 12.

Table 12: An embedded Excel Worksheet containing the variance covariance matrices for the cost regressions.

Variance - covariance matrix for insulin costs

			Insulin (
		Multiplier for the baseline insulin cost	Multiplier for the Baseline HbA1c
Insulin costs year 1	Multiplier for the baseline insulin cost	0.0204	
	Multiplier for the Baseline HbA1c	0.0495	44.1713
	Receiving CSII at the start of the year	0.2008	-27.1177
	Switch from CSII to MDI	3.8634	220.4913
	Constant	-7.4078	-404.7725
Insulin costs ongoing	Multiplier for the baseline insulin cost	0.0140	0.1719

For peer review only

For peer review only - http://bmjopen.bmj.com/site/about/guidelines.xhtml

5 Detailed results of the scenario and subgroup analyses

Table 13: The One way scenario analyses and subgroup analyses performed using the Sheffield Type 1 Diabetes Policy Model

	MDI + DAFNE		CSII + DAFNE		Incremental		
	Total discounted costs	Total discounted QALYs	Total discounted costs	Total discounted QALYs	Total discounted costs	Total discounted QALYs	ICER (£ per QALY gained)
Base Case - PSA	£81,785	12.9845	£100,617	13.1105	£18,832	0.1260	£149,483
Base Case - Deterministic	£73,963	12.8077	£93,236	12.9834	£19,273	0.1757	£109,684
Scenario - Pump prices were estimated from Riemsma et al.[11]	£73,912	12.8077	£92,414	12.9834	£18,502	0.1757	£105,295
Scenario - 25% price reduction in insulin pumps and consumables	£73,513	12.8077	£85,939	12.9834	£12,426	0.1757	£70,715
Scenario - 50% price reduction in insulin pumps and consumables	£73,063	12.8077	£78,641	12.9834	£5,578	0.1757	£31,747
Scenario – Intention to treat estimate of treatment effect	£74,200	12.8997	£94,400	12.9635	£20,200	0.0638	£316,785
Scenario - Intention to treat estimate of treatment effect and no change in HbA1c if an individual switches treatment	£73,308	12.9272	£93,496	12.9650	£20,188	0.0378	£534,397
Scenario – Base case & no change in HbA1c when switching	£73,799	12.8560	£93,329	12.9499	£19,530	0.0939	£207,874
Scenario – Post-trial HbA1c progression in both arms is estimated from the DCCT	£74,278	12.8269	£92,472	12.9704	£18,194	0.1435	£126,776
Scenario - Individuals return to their baseline HbA1c after 3 years and no progression thereafter	£70,053	13.0962	£91,063	12.9921	£21,011	-0.1041	Dominated
Scenario - HbA1c effects occur one model cycle earlier	£75,014	12.7840	£93,868	12.9796	£18,854	0.1956	£96,394
Scenario – individuals return to their baseline risk of hypos and DKA at three years	£73,963	12.8077	£93,236	12.9834	£19,273	0.1757	£109,684
Scenario - Switching probabilities were estimated directly from the Kaplan - Meier curves	£73,516	12.8267	£93,911	12.9447	£20,394	0.1180	£172,836
Scenario – The utility decrement for blindness was estimated from the Brown *et al*. study.[12]	£81,785	12.9845	£100,617	13.1105	£18,832	0.1260	£149,483

For peer review only - http://bmjopen.bmj.com/site/about/guidelines.xhtml

Table 13: The One way scenario analyses and subgroup analyses performed using the Sheffield Type 1 Diabetes Policy Model (continued)

	MDI + DAFNE		CSII + DAFNE		Incremental		
	Total discounted costs	Total discounted QALYs	Total discounted costs	Total discounted QALYs	Total discounted costs	Total discounted QALYs	ICER (£ per QALY gained)
Subgroup - individuals with a baseline HbA1c < 8.5%	£73,963	12.7992	£93,236	12.9743	£19,273	0.1750	£110,115
Subgroup- individuals with a baseline HbA1c ≥ 8.5%	£57,947	13.3800	£79,001	13.4185	£21,054	0.0385	£547,504
Subgroup - individuals with a baseline HbA1c ≥ 7.5%	£85,028	12.3238	£103,990	12.3986	£18,962	0.0748	£253,352
Subgroup - Individuals with a baseline HbA1c ≥ 7.5% & <8.5%	£76,735	12.5809	£95,481	12.7369	£18,746	0.1559	£120,239
Subgroup - Individuals with a baseline HbA1c ≥ 8.5% & <9.5%	£61,207	13.0718	£82,337	13.1913	£21,131	0.1195	£176,887
Subgroup - Individuals with a baseline HbA1c ≥ 9.5%	£66,520	13.4669	£86,105	13.5991	£19,584	0.1321	£148,240
Subgroup - Individuals in the per protocol population	£99,249	11.6388	£115,473	11.8074	£16,224	0.1686	£96,231
Subgroup - Individuals in the per protocol population and no treatment switching	£72,955	12.7159	£92,351	12.8834	£19,395	0.1675	£115,786

MDI - multiple daily injections; DAFNE - dose adjustment for normal eating; CSII - continuous subcutaneous insulin infusion; ICER - incremental cost-effectiveness ratio; PSA - probabilistic sensitivity analysis; DCCT - Diabetes Control and Complications Trial

For peer review only - http://bmjopen.bmj.com/site/about/guidelines.xhtml

References

1. Office for National Statistics. National Life Tables, United Kingdom : 2012–2014. *Online Source* 2016;Available from: http://www.ons.gov.uk/peoplepopulationandcommunity/birthsdeathsandmarriages/lifeexpectancies/bulletins/nationallifetablesunitedkingdom/previousReleases . (Last Accessed: 17/03/2016)
2. Office for National Statistics. Deaths Registered in England and Wales (Series DR). Table 5 for years 2014, 2013 and 2012. *Online Source* 2015;Available from: http://www.ons.gov.uk/peoplepopulationandcommunity/birthsdeathsandmarriages/deaths/datasets/deathsregisteredinenglandandwalesseriesdrreferencetables(Last Accessed: 17/03/2016)
3. National Records of Scotland. Vital Events Reference Tables. *Online Source* 2015;Available from: http://www.nrscotland.gov.uk/statistics-and-data/statistics/statistics-by-theme/vital-events/general-publications/vital-events-reference-tables/2014(Last Accessed 17/03/2016)
4. Northern Ireland Statistics and Research Agency. Microdata for Deaths in Northern Ireland, 2001-2014. *Online Source* 2014;Available from: http://www.nisra.gov.uk/archive/demography/publications/births_deaths/Pivot_Table_2001_2014.xlsx(Last Accessed 17/03/2016)
5. Heller S, Lawton J, Amiel S, et al. Improving management of type 1 diabetes in the UK: the Dose Adjustment For Normal Eating (DAFNE) programme as a research test-bed. A mixed-method analysis of the barriers to and facilitators of successful diabetes self-management, a health economic analysis, a cluster randomised controlled trial of different models of delivery of an educational intervention and the potential of insulin pumps and additional educator input to improve outcomes. *Programme grants for applied research* 2014;2(5) doi: 10.3310/pgfar02050
6. Wolfe RA, Ashby VB, Milford EL, et al. Comparison of mortality in all patients on dialysis, patients on dialysis awaiting transplantation, and recipients of a first cadaveric transplant. *N Engl J Med* 1999;341(23):1725-30. doi: 10.1056/NEJM199912023412303
7. Briggs A, Claxton K, Sculpher M. Decision Modelling for Health Economic Evaluation. Oxford: Oxford University Press 2006.
8. Department of Health. NHS reference costs 2013 to 2014. *Online Source* 2014;Available from; https://www.gov.uk/government/publications/nhs-reference-costs-2013-to-2014. Last accessed 17th July 2015
9. Health & Social Care Information Centre. Prescription Cost Analysis England 2014. *Online Source* 2015;Available from; http://www.hscic.gov.uk/catalogue/PUB17274. Last Accessed 2nd Novemeber 2015
10. British Medical Association and Royal Pharmaceutical Society of Great Britain. British National Formulary August 2015. *Online Source* 2015;Available from: https://www.medicinescomplete.com, Last Accessed 9th September 2015
11. Riemsma R, Corro Ramos I, Birnie R, et al. Type 1 diabetes: Integrated sensor-augmented pump therapy systems for managing blood glucose levels (The MiniMed Paradigm Veo System and the Vibe and G4 PLATINUM CGM system), a systematic review and economic evaluation. *Kleijnen Systematic Reviews Ltd* 2015
12. Brown MM, Brown GC, Sharma S, et al. Quality of life with visual acuity loss from diabetic retinopathy and age-related macular degeneration. *Arch Ophthalmol* 2002;120(4):481-4.

For peer review only - http://bmjopen.bmj.com/site/about/guidelines.xhtml

CHEERS Checklist
Items to include when reporting economic evaluations of health interventions

The **ISPOR CHEERS Task Force Report**, *Consolidated Health Economic Evaluation Reporting Standards (CHEERS)—Explanation and Elaboration: A Report of the ISPOR Health Economic Evaluations Publication Guidelines Good Reporting Practices Task Force,* provides examples and further discussion of the 24-item CHEERS Checklist and the CHEERS Statement. It may be accessed via the *Value in Health* or via the ISPOR Health Economic Evaluation Publication Guidelines – CHEERS: Good Reporting Practices webpage: http://www.ispor.org/TaskForces/EconomicPubGuidelines.asp

Section/item	Item No	Recommendation	Reported on page No/ line No
**Title and abstract**			
Title	1	Identify the study as an economic evaluation or use more specific terms such as "cost-effectiveness analysis", and describe the interventions compared.	pi, line 1 to 3
Abstract	2	Provide a structured summary of objectives, perspective, setting, methods (including study design and inputs), results (including base case and uncertainty analyses), and conclusions.	pii to piii, line 2
**Introduction**			
Background and objectives	3	Provide an explicit statement of the broader context for the study.<br>Present the study question and its relevance for health policy or practice decisions.	p1, lines 1 to 41<br>p1, line 66 to p2, line 6
**Methods**			
Target population and subgroups	4	Describe characteristics of the base case population and subgroups analysed, including why they were chosen.	p3, line 16 to 27 & p5, line 31 to p6 line 2
Setting and location	5	State relevant aspects of the system(s) in which the decision(s) need(s) to be made.	p2, lines 10 to 17
Study perspective	6	Describe the perspective of the study and relate this to the costs being evaluated.	p2, line 16 to 26
Comparators	7	Describe the interventions or strategies being compared and state why they were chosen.	p1, line 12 to 41
Time horizon	8	State the time horizon(s) over which costs and consequences are being evaluated and say why appropriate.	p2, line 16 to 17
Discount rate	9	Report the choice of discount rate(s) used for costs and outcomes and say why appropriate.	p2, line 19 to 20
Choice of health outcomes	10	Describe what outcomes were used as the measure(s) of benefit in the evaluation and their relevance for the type of analysis performed.	p2, line 13 to 15
Measurement of effectiveness	11a	*Single study-based estimates:* Describe fully the design features of the single effectiveness study and why the single study was a sufficient source of clinical effectiveness data.	p3, line 1 to 13 & p1, line 39 to p2, line 2

	11b	*Synthesis-based estimates:* Describe fully the methods used for identification of included studies and synthesis of clinical effectiveness data.	N/A
Measurement and valuation of preference based outcomes	12	If applicable, describe the population and methods used to elicit preferences for outcomes.	N/A
Estimating resources and costs	13a	*Single study-based economic evaluation:* Describe approaches used to estimate resource use associated with the alternative interventions. Describe primary or secondary research methods for valuing each resource item in terms of its unit cost. Describe any adjustments made to approximate to opportunity costs.	N/A
	13b	*Model-based economic evaluation:* Describe approaches and data sources used to estimate resource use associated with model health states. Describe primary or secondary research methods for valuing each resource item in terms of its unit cost. Describe any adjustments made to approximate to opportunity costs.	p4, line 38 to p5, line 10
Currency, price date, and conversion	14	Report the dates of the estimated resource quantities and unit costs. Describe methods for adjusting estimated unit costs to the year of reported costs if necessary. Describe methods for converting costs into a common currency base and the exchange rate.	p4 lines 21 to 40
Choice of model	15	Describe and give reasons for the specific type of decision-analytical model used. Providing a figure to show model structure is strongly recommended.	p2, line 21 to 40
Assumptions	16	Describe all structural or other assumptions underpinning the decision-analytical model.	
Analytical methods	17	Describe all analytical methods supporting the evaluation. This could include methods for dealing with skewed, missing, or censored data; extrapolation methods; methods for pooling data; approaches to validate or make adjustments (such as half cycle corrections) to a model; and methods for handling population heterogeneity and uncertainty.	p3, line 29 to p4, line 36
**Results**			
Study parameters	18	Report the values, ranges, references, and, if used, probability distributions for all parameters. Report reasons or sources for distributions used to represent uncertainty where appropriate. Providing a table to show the input values is strongly recommended.	Tables 2&3 & Supplementary Material B: Tables 1, 2, 5, 6, 9 and 10
Incremental costs and outcomes	19	For each intervention, report mean values for the main categories of estimated costs and outcomes of interest, as well as mean differences between the comparator groups. If applicable, report incremental cost-effectiveness ratios.	p6 line 28 to 39
Characterising uncertainty	20a	*Single study-based economic evaluation:* Describe the effects of sampling uncertainty for the estimated incremental cost and incremental effectiveness parameters, together with the impact	N/A

		of methodological assumptions (such as discount rate, study perspective).	
	20b	*Model-based economic evaluation:* Describe the effects on the results of uncertainty for all input parameters, and uncertainty related to the structure of the model and assumptions.	p6, line33 to p7, line 12
Characterising heterogeneity	21	If applicable, report differences in costs, outcomes, or cost-effectiveness that can be explained by variations between subgroups of patients with different baseline characteristics or other observed variability in effects that are not reducible by more information.	p7 line 16 to 18
**Discussion**			
Study findings, limitations, generalisability, and current knowledge	22	Summarise key study findings and describe how they support the conclusions reached. Discuss limitations and the generalisability of the findings and how the findings fit with current knowledge.	p7 line 20 to p8 line 13
**Other**			
Source of funding	23	Describe how the study was funded and the role of the funder in the identification, design, conduct, and reporting of the analysis. Describe other non-monetary sources of support.	p10 lines 2 to 8
Conflicts of interest	24	Describe any potential for conflict of interest of study contributors in accordance with journal policy. In the absence of a journal policy, we recommend authors comply with International Committee of Medical Journal Editors recommendations.	p10 lines 10 to 15

For consistency, the CHEERS Statement checklist format is based on the format of the CONSORT statement checklist

The **ISPOR CHEERS Task Force Report** provides examples and further discussion of the 24-item CHEERS Checklist and the CHEERS Statement. It may be accessed via the *Value in Health* link or via the ISPOR Health Economic Evaluation Publication Guidelines – CHEERS: Good Reporting Practices webpage: http://www.ispor.org/TaskForces/EconomicPubGuidelines.asp

The citation for the CHEERS Task Force Report is:

Husereau D, Drummond M, Petrou S, et al. Consolidated health economic evaluation reporting standards (CHEERS)—Explanation and elaboration: A report of the ISPOR health economic evaluations publication guidelines good reporting practices task force. Value Health 2013;16:231-50.

BMJ Open

The cost-effectiveness of insulin pumps compared to multiple daily injections, both provided with structured education, for adults with type 1 diabetes: a health economic analysis of the relative effectiveness of pumps over structured education (REPOSE) randomised controlled trial.

Journal:	*BMJ Open*
Manuscript ID	bmjopen-2017-016766.R1
Article Type:	Research
Date Submitted by the Author:	05-Dec-2017

Complete List of Authors:	Pollard, Daniel ; University of Sheffield, School of Health and Related Research<br>Brennan, Alan; University of Sheffield, School of Health & Related Research<br>Dixon, Simon; University of Sheffield, School of Health and Related Research<br>Waugh, Norman; University of Warwick, Warwick Evidence<br>Elliott, Jackie; University of Sheffield, Academic Unit of Diabetes, Endocrinology and Metabolism<br>Heller, Simon; Unversity of Sheffield, Academic Unit of Diabetes, Endo and Metab<br>Lee, Ellen; University of Sheffield, Clinical Trials Research Unit<br>Campbell, Michael; University of Sheffield, Health Services Research ScHARR<br>Basarir, Hasan; RTI Health Solutions<br>White, David; University of Sheffield, Clinical Trials Research Unit

**Primary Subject Heading**:	Health economics
Secondary Subject Heading:	Diabetes and endocrinology
Keywords:	HEALTH ECONOMICS, DIABETES & ENDOCRINOLOGY, General diabetes < DIABETES & ENDOCRINOLOGY

SCHOLARONE™ Manuscripts

Title: The cost-effectiveness of insulin pumps compared to multiple daily injections, both provided with structured education, for adults with type 1 diabetes: a health economic analysis of the relative effectiveness of pumps over structured education (REPOSE) randomised controlled trial.

Running head: Cost-effectiveness of insulin pumps with structured education for adults with type 1 diabetes

Authors:

Daniel Pollard, Research Associate, School of Health and Related Research (ScHARR), University of Sheffield, Sheffield, UK

Alan Brennan, Professor of Health Economics and Decision Modelling, School of Health and Related Research (ScHARR), University of Sheffield, Sheffield, UK

Simon Dixon, Professor of Health Economics, School of Health and Related Research (ScHARR), University of Sheffield, Sheffield, UK

Norman Waugh, Professor in Public Health, Warwick Medical School – Population Evidence and Technologies, University of Warwick, Coventry, UK

Jackie Elliott, Senior Clinical Lecturer in Diabetes, Academic Unit of Diabetes, Endocrinology and Metabolism, Department of Oncology and Metabolism, School of Medicine and Biomedical Sciences, University of Sheffield, UK

Simon Heller, Professor of Clinical Diabetes, Academic Unit of Diabetes, Endocrinology and Metabolism, Department of Oncology and Metabolism, School of Medicine and Biomedical Sciences, University of Sheffield, UK

Ellen Lee, Research Associate, Clinical Trials Research Unit, School of Health and Related Research (ScHARR), University of Sheffield, Sheffield, UK

Michael Campbell, Professor of Medical Statistics, School of Health and Related Research (ScHARR), University of Sheffield, Sheffield, UK

Hasan Basarir, Director, RTI Health Solutions, Greater Manchester, UK

David White, Trial manager, Clinical Trials Research Unit, School of Health and Related Research (ScHARR), University of Sheffield, Sheffield, UK

On behalf of the REPOSE group

Correspondence to Daniel Pollard. Email: d.j.pollard@sheffield.ac.uk. Telephone: +44 (0) 114 222 6126

Abstract: 298 words

Main text: 5,160 words

For peer review only

For peer review only - http://bmjopen.bmj.com/site/about/guidelines.xhtml

Abstract

Objectives

To assess the long term cost-effectiveness of insulin pumps and dose adjustment for normal eating (pumps+DAFNE) compared to multiple daily insulin injections and dose adjustment for normal eating (MDI+DAFNE) for adults with type 1 diabetes mellitus (T1DM) in the UK.

Methods

We undertook a cost-utility analysis using the Sheffield Type 1 Diabetes Policy Model and data from the REPOSE trial to estimate the lifetime incidence of diabetic complications, intervention-based resource use and associated effects on costs and quality adjusted life years (QALYs). All economic analyses took an NHS and personal social services perspective and discounted costs and quality adjusted life years at 3.5% per annum. A probabilistic sensitivity analysis was performed on the base case. Further uncertainties in the cost of pumps and the evidence used to inform the model were explored using scenario analyses.

Setting

Eight diabetes centres in England and Scotland

Participants

Adults with T1DM who were eligible to receive a structured education course, did not have a strong clinical indication or a preference for a pump.

Intervention

pumps +DAFNE

Comparator

MDI+DAFNE

Main outcome measures

Incremental costs, incremental QALYs gained and incremental cost-effectiveness ratios (ICERs).

Results

Compared to MDI+DAFNE, pumps+DAFNE was associated with an incremental discounted lifetime cost of +£18,853 (95%CI £6,175, £31,645) and a gain in discounted lifetime quality adjusted life years of +0.13 (95%CI -0.70, +0.96). The base case mean ICER was £142,195 per QALY gained. The probability of pump+DAFNE being cost-effective using a cost-effectiveness threshold of £20,000 per QALY gained was 14.0%. All scenario and subgroup analyses examined indicated that the ICER was unlikely to fall below £30,000 per QALY gained.

Conclusions

For peer review only - http://bmjopen.bmj.com/site/about/guidelines.xhtml

Our analysis of the REPOSE data suggests that routine use of pumps in adults without an immediate clinical need for a pump, as identified by NICE, would not be cost-effective.

Strengths and limitations of this paper

This study is the first cost-effectiveness analysis to consider the use of insulin pumps for adults with type 1 diabetes who are eligible to receive structured education course but not an insulin pump using current UK guidance.

The REPOSE trial was the first trial to address the question of whether insulin pumps were a clinically effective treatment option in this patient population.

An existing validated model of type 1 diabetes and its complications, developed during a previous National Institute for Health Research funded programme grant, was adapted and updated with evidence from the REPOSE trial and more recent evidence on clinical effectiveness, utilities and costs

We considered a UK health care perspective to estimate the key drivers of decision uncertainty.

The main limitations of the study are that the only available evidence in long term trends in HbA1c (the key clinical outcome) was from several observational studies, the analysis of diabetic ketoacidosis was based on self-reported information rather than data from inpatient admissions and that the trial only included one type of insulin pump.

For peer review only - http://bmjopen.bmj.com/site/about/guidelines.xhtml

For peer review only

For peer review only - http://bmjopen.bmj.com/site/about/guidelines.xhtml

INTRODUCTION

People with type 1 diabetes mellitus (T1DM) are unable to produce insulin due to autoimmune destruction of their insulin secreting beta cells. Those affected have to inject insulin to prevent diabetic ketoacidosis in the short-term, and in the long term, to prevent microvascular and macrovascular disease. Insulin is generally administered by intermittent subcutaneous injection with the dose adjusted according to eating, physical activity and current blood glucose levels. Therapy is designed to keep blood glucose as close to normal as possible, to prevent both microvascular complications and to reduce the risk of macrovascular disease.[1] A further aim of treatment is to achieve as good a quality of life (QoL) as possible, particularly since self-management of the condition is challenging and arduous, demanding the implementation of complex skills.

Historically, insulin was given twice a day, often as pre-mixed insulin, but such an approach imposes a rigid lifestyle on patients and makes it difficult to maintain blood glucose levels close to normal. Most individuals require intensive insulin therapy to maintain tight glycaemic control. This approach and its integration within a flexible lifestyle is promoted in structured training courses, such as the Dose Adjustment For Normal Eating (DAFNE)[2] course and others.[3] The principles of this multiple daily injection (MDI) via subcutaneous injection approach involves the use of quick-acting insulin injected before eating (with the dose calculated according to the amount of carbohydrate eaten) combined with long-acting background 'basal' insulin, usually given twice daily, to control blood glucose in between meals.

As an alternative to MDI, insulin can be administered by an insulin pump system in which insulin is delivered throughout the day using a small, portable pump, connected by a flexible plastic tube to a subcutaneous cannula. This technology is relatively expensive compared to MDI. Current National Institute for Health and Care Excellence (NICE) guidelines recommend that all adults with T1DM in the UK receive a structured education course of proven benefit at a clinically appropriate time.[4] Insulin pumps are a treatment option for adults with T1DM who either have a HbA1c above 69 mmol/mol (8.5%) or experience disabling hypoglycaemia.[5] Insulin pumps do not replace the need for education and it is currently recommended that specialist teams should provide structured education programmes and advice on diet, lifestyle and exercise for people using an insulin pump.[5]

Use of pumps in T1DM varies substantially between countries. In England, 11.7% of people with T1DM are estimated to use a pump which compares to around 40% in the USA.[6 7] It has been proposed that insulin pumps are underutilised in the UK and that the glycaemic control of adults with T1DM could be improved if pumps were used more widely.[8] Two NICE appraisals found that there was insufficient evidence on insulin pump therapy compared to MDI in which adults with T1DM used both long and short acting insulins.[9 10] Furthermore, a recent observational study of pumps and MDI suggested that much of the previous benefit attributed to pumps may be due to the additional education that pumps users require when initiating therapy.[11] Therefore, uncertainty remains as to the clinical benefit and cost-effectiveness of pumps as a treatment option for adults with T1DM.

The relative effectiveness of pumps over structured education (REPOSE) trial was conducted to assess if pumps offered any additional benefit compared to MDI for the treatment of adults with

For peer review only - http://bmjopen.bmj.com/site/about/guidelines.xhtml

T1DM in the UK who are eligible to attend a structured education course, but do not have an immediate clinical need for a pump. Participants in both trial arms received a DAFNE course, which taught MDI users to use long and short acting insulins appropriately. In this paper we present the cost-effectiveness analysis of pumps+DAFNE compared to MDI+DAFNE for adults in the UK with T1DM who are eligible to receive a structured education course, but did not have a clinical need for the immediate commencement of pump therapy.

METHODS

Economic evaluation methods

The health economic analysis followed the pre-specified health economic analysis plan, which was outlined in the trial protocol paper.[12] Two approaches were undertaken to assess the cost-effectiveness of pumps: an economic evaluation alongside the clinical trial (EEACT); and, a long term economic model to assess the lifetime outcomes. In the EEACT, data on the costs and QALYs were obtained from data collected in the REPOSE trial, whereas the long term model estimated the lifetime costs and QALYs based mainly upon the biomedical outcomes collected in the REPOSE trial. All modelling analyses took a lifetime time horizon and all EEACT analyses took a two-year (trial duration) time horizon. The pre-specified primary analysis relates to the life time modelling, and as such, are reported in this paper. The methods and results of the EEACT are reported in supplementary material A. In line with NICE guidance, all economic analyses took an NHS and personal social services perspective and costs and QALY outcomes were discounted at 3.5%.[13]

The economic model

The Sheffield Type 1 Diabetes Policy Model version 1.3.2, henceforth 'the model', is an individual level simulation model used to estimate the lifetime costs and QALYs associated with pump+DAFNE and MDI+DAFNE. The most recently published version of the model[14] was updated using data collected in the REPOSE trial at 12-month and 24-month follow-up. Specifically, the level of HbA1c, risk of severe hypoglycaemia, risk of diabetic ketoacidosis (DKA) and the probability that an individual would switch insulin delivery mechanism and costs-related to the intervention (insulin and MDI consumables, diabetes related contacts with health care professionals, insulin pumps and the associated consumables) were updated. Relevant literature also informed the changes in HbA1c beyond the two-year trial duration and the probability of death from end stage renal disease (see supplementary material B, page 2) in both arms. The model examines disease progression over a lifetime using an annual time cycle. An individual's HbA1c determines their risk of progression for all diabetic complications in the model, which include: nephropathy, neuropathy, retinopathy, macular oedema, myocardial infarction, stroke, heart failure, angina and severe hypoglycaemia and DKA. A higher HbA1c increases the risk of progression for all complications in the model. Individuals in the model are at risk of death from the incidence of: nephropathy, myocardial infarction, stroke, heart failure, angina and all-cause mortality. HbA1c indirectly effects mortality in the model, as the probability death does not differ by HbA1c, however the risk of experiencing these events is higher for someone with a higher HbA1c.The model attaches utilities to health states and costs to events, allowing the calculation of costs and QALYs over a lifetime. Full details of how the model calculates the incidence of diabetes complications is provided in Heller *et al* (pages 104-106) and Thokala *et al*.[14 15] The incremental cost-effectiveness ratio (ICER) calculated was compared to the £20,000 to

For peer review only - http://bmjopen.bmj.com/site/about/guidelines.xhtml

£30,000 per QALY gained threshold used by NICE.[13] In each model run, the life course of 5,000 individuals was simulated. This number of simulated individuals was considered to be sufficiently robust for decision making (see Supplementary Material B, page 1). All model analyses were conducted using SIMUL8 professional 2010.[16]

The clinical data

The details of the methods used in the REPOSE trial have been reported elsewhere.[12] The eligibility criteria of the trial included adults with T1DM if they were eligible to receive a structured education course, did not have a clinical indication to receive a pump immediately, as determined by the investigator, or did not have a strong preference to receive a pump.[12] The REPOSE trial was conducted in eight centres in England and Scotland, involving 267 individuals. Out of these 267 individuals, 260 had HbA1c data for at least one post-baseline follow up visit (ITT population) and 236 adhered to their randomised treatment (per protocol population). Self-reported participant information, which includes the number of DKA events, EQ-5D-3L, SF-12 and resource use were collected at baseline, 6 months, 1 year and 2 years after attending DAFNE training. HbA1c, the primary endpoint, was measured at baseline and at these follow-up appointments. All inpatient admissions, the incidence of severe hypoglycaemia and whether an individual switched insulin delivery method were collected on an ongoing basis. Information was collected on the staff time spent delivering pre-course fitting sessions for individuals allocated to receive a pump. The cost of insulin pumps and associated consumables in routine practice, and the insulin pump consumables used by the REPOSE participants, were collected from seven of the eight trial centres.

The simulation cohort

To obtain the simulation cohort, participants REPOSE data (n=260) were sampled with replacement 5,000 times.[17] This created a simulation cohort of 5,000 individuals for whom some would have missing baseline characteristics. Each individual from REPOSE was included in the simulation cohort a median of 19 times (interquartile range 16 – 22). To obtain data values for these missing variables in the simulation cohort, two imputation procedures were used. A chained truncated regression procedure for missing continuous variables (baseline: total cholesterol, HDL cholesterol, systolic blood pressure and cost of insulin used in the year prior to baseline), in which variables were limited to positive values, and a Poisson procedure for missing categorical variables (gender). In the truncated regression imputation procedure, all characteristics with complete data in the REPOSE trial population were used as predictive covariates. In the Poisson imputation procedure, all characteristics with complete data in the REPOSE trial population and the imputed data for the continuous variables were used as predictive covariates. In both imputation procedures, a single imputation was used. As this was preformed in the simulation cohort rather than the REPOSE data, the replications of the individuals in the simulation cohort had different data values if they had missing baseline data from the trial. Identical individuals were simulated in both model arms. A summary of the baseline characteristics and completeness of data for the 260 REPOSE participants in the ITT population and the 5,000 individuals in the simulation cohort are given in Table 1.

<Table 1>

The clinical effectiveness data

For peer review only - http://bmjopen.bmj.com/site/about/guidelines.xhtml

Statistical analyses of the REPOSE trial data were conducted to estimate treatment switching, HbA1c, severe hypoglycaemia and DKA. The main clinical results paper is available, however it differs in this paper as it focuses on statistical in those people with a HbA1c greater than or equal to 58 mmol/mol (7.5%).[18] Information on long term changes in HbA1c was obtained from the available literature and the REPOSE trial data. Unless otherwise stated, all statistical analyses were conducted using STATA v13.1.[19] Full details on the statistical methods used, the results and how the analyses were incorporated into the model are provided in supplementary material B.

To estimate the incidence of treatment switching in the first and second year of the model, a time to event analysis was conducted using treatment switching as the event of interest. Kaplan-Meier curves were plotted and parametric survival curves were fitted to the Kaplan-Meier curves.[20 21] Separate parametric survival models were fitted to individuals randomised to pump+DAFNE and MDI+DAFNE. The goodness of fit of the parametric survival curves was assessed using the Akaike information criterion (AIC), Bayesian information criterion (BIC) and visual assessment of the fit of the parametric curves to the Kaplan-Meier curves at one and two years. As treatment switching was included in the model, it is important to adjust the modelled treatment effectiveness and cost of treatment for those individuals who switch. If individuals switched treatment, then their HbA1c was assumed to change as though they had been allocated to the other arm. No explicit effect of switching on the incidence rate ratio associated with the model arms for either severe hypoglycaemia or DKA was included. However, due to the change in HbA1c associated with switching, individuals who switched from CSII to MDI were at a lower risk of severe hypoglycaemia and at a higher risk of DKA. The opposite was true for those individuals who switched from MDI to CSII. In the base case analysis, the HbA1c effect of pump+DAFNE compared to MDI+DAFNE was estimated in the per protocol population, as the people who switched treatment were excluded from this population.

To estimate each individual's HbA1c in the model, a beta regression was fitted to the HbA1c data collected in REPOSE for all individuals in the per protocol population at the one and two-year follow-up.[22] Bounds were placed on the beta regression so that HbA1c was between the clinically plausible bounds of 29mmol/mol [4.8%] and 201 mmol/mol [20.5%], which were provided by two clinical experts in the REPOSE trial management group. Missing data was observed for HbA1c values in the per protocol population at 6 months (2.1% missing), 1 year (4.2% missing) and 2 years (4.2% missing). Full details on the imputation procedure used to account for the missing data and the specification of the beta regression are provided in supplementary material B (see pg8). The effects in a beta regression are not easily interpretable by themselves, however information can be obtained on the direction of effect.

The expert opinion of clinical members involved in the literature review of clinical studies for the REPOSE trial was sought to identify studies with long term data on HbA1c for the type of pumps used in the trial and people who used MDI after DAFNE. Three articles on the long term trends in HbA1c for pump users[23-25] and two articles on the long term trends in HbA1c for MDI users post-DAFNE[26 27] were used to estimate yearly changes in HbA1c for each model arm. The average annual increase in HbA1c was calculated for each study by calculating yearly increase in HbA1c between the lowest observed HbA1c value and the last follow up point in the study where the sample size was greater than one quarter of the initial sample size. A standard error could not be directly calculated, as we did not have access to the patient level data from the studies. Instead, data on the standard

For peer review only - http://bmjopen.bmj.com/site/about/guidelines.xhtml

deviation of the change in HbA1c between the 1 and 2 year follow ups specific to each REPOSE trial arm was used to inform the uncertainty in this parameter. These standard deviations were divided by the square root of the combined sample size of the studies used to inform the mean effect to estimate standard error for this parameter.

Negative binomial regressions were used to estimate the risk of severe hypoglycaemia and DKA in the ITT population of REPOSE using the Zellig package in R version 3.2.0. Negative binomial regressions were fitted separately to severe hypoglycaemia and DKA events and to the first and second year of follow up data. The regressions fitted to the second year data were used to estimate the incidence of severe hypoglycaemia and DKA in all model time cycles, except for the first time cycle. This assumption was based on clinical expert opinion, which was confirmed by the trial management group, that the first 6 months of using a pump was associated with a learning period and after this time the incidence of severe hypoglycaemia and DKA would decrease.

Costs and utilities

The costs and health state utility values used for each health state in the model are provided in Table 2 and Table 3 respectively. All costs were reported in 2013/14 prices. Costs sourced from evidence in previous years were inflated to 2013/14 prices using the hospital and community health services pay and prices index.[28] The cost of insulin and MDI consumables used, cost of diabetes related contacts with healthcare professionals and cost of the pump intervention costs were estimated separately for both year 1 and year 2 of the REPOSE trial. The unit cost of insulin and MDI consumables used was obtained from the British National Formulary and Health & Social Care Information Centre, the unit cost of diabetes related contacts were obtained from NHS reference costs and unit cost of an insulin pump (with its associated consumables) was obtained from a survey of the REPOSE trial sites.[29-31] Table 2 part A shows the 6 regression formulae which estimate an individual's annual cost for each of these components as a function of:- their baseline HbA1c, which treatment they start the year on (MDI or pump), whether they switch treatment, and also the participants' self-reported use of insulin and level of contact with healthcare professionals prior to recruitment to the trial. Details on how these parameters were used to estimate the costs in the model are provided in Table 2.

<Table 2 and 3>

Scenario and subgroup analyses

For the base case, a probabilistic sensitivity analysis (PSA) was conducted where each parameter was sampled from its probability distribution, the results were recorded, the process was repeated 500 times and the averages from these 500 model runs were reported. This number of PSA runs was considered to be sufficient to allow for robust decision making based upon the model results (see Supplementary Material B, page 1). All statistical models, except for the risks of severe hypoglycaemia and DKA, for which the outcomes were directly simulated in R version 3.2.0, were included in the model using a multivariate normal distribution.[32] The long term changes in HbA1c were assumed to be distributed using independent normal distributions for each model arm. Deterministic scenario analyses were undertaken to assess the robustness of the results. The key scenario analyses for the model include: uncertainty in the estimates of treatment effectiveness, the

For peer review only - http://bmjopen.bmj.com/site/about/guidelines.xhtml

timing of HbA1c changes in the model, the utility decrement for blindness (-0.26) [33] and the cost of insulin pumps and insulin pump consumables.

A two-way deterministic threshold analysis was conducted to assess the HbA1c reduction and/or annual cost reduction necessary to potentially make pumps cost-effective in the UK. All threshold analysis runs were conducted deterministically. In this analysis, each individual's HbA1c was estimated as though they received MDI. Then those individuals in the pump arm received a fixed change in HbA1c. This change in HbA1c was varied at 10 different values between-3.3mmol/mol (-0.3%) and-13.1mmol/mol (-1.2%), and annual cost of insulin pumps were also varied between 100% of the observed cost in the REPOSE trial and 50% of the observed cost.

Deterministic subgroup analyses were conducted in the following populations:

1. Baseline HbA1c < 69 mmol/mol (8.5%)
2. Baseline HbA1c ≥ 69 mmol/mol (8.5%)
3. Baseline HbA1c ≥ 58 mmol/mol (7.5%)
4. 69 mmol/mol (8.5%) >baseline HbA1c ≥ 58 mmol/mol (7.5%)
5. 80 mmol/mol (9.5%) > baseline HbA1c ≥ 69 mmol/mol (8.5%)
6. Baseline HbA1c ≥ 80 mmol/mol (9.5%)
7. All individuals in the per protocol population

The subgroup analyses were conducted by resampling the simulation cohort from the individuals who met each of these criteria in the ITT population.

RESULTS

Clinical effectiveness data

Treatment Switching

The analysis of treatment switching in the REPOSE trial suggested that an exponential curve provided the best fit to the data for individuals receiving pump+DAFNE and a Weibull curve provided the best fit for individuals in the MDI+DAFNE arm.

HbA1c

The results of the analysis of HbA1c data suggested that pump+DAFNE compared to MDI+DAFNE was associated with statistically insignificant HbA1c reductions in both year 1 and in year 2. The analysis of the literature for long-term trends in HbA1c suggested that weighted average annual progression of HbA1c was +0.568mmol/mol (+0.052%) per annum in the pump+DAFNE arm and +0.590 mmol/mol (+0.054%) per annum in the MDI+DAFNE arm. The estimated standard errors for the long-term trends in HbA1c were of 0.627 mmol/mol (0.040%) for MDI+DAFNE and 0.627 mmol/mol (0.057%) for pump+DAFNE.

For peer review only - http://bmjopen.bmj.com/site/about/guidelines.xhtml

These data produce a trace of HbA1c as in Figure 1. The solid lines show the average HbA1c in the model for each year. The dotted lines show what the HbA1c would have been in each year, if a higher HbA1c did not have an indirect effect on the number of deaths in the model or treatment switching effects. The dashed line, shows the number of people remaining alive in each year and is plotted on the secondary axis. As expected from the base case clinical results, the MDI+DAFNE arm has a higher HbA1c than the CSII+DAFNE arm and this effect slightly increases over time

<Figure 1>

Severe Hypoglycemia and Diabetic Ketoacidosis

The results of the negative binomial models used to estimate the incidence of DKA indicated that pump+DAFNE compared to MDI+DAFNE was associated with more DKA events in the first year (incidence rate ratio (IRR) 1.40, 95% confidence interval (CI) 0.55 to 3.58), but fewer events in year 2 (IRR 0.93, 95% CI 0.23 to 3.69). The results of the negative binomial models used to estimate the incidence of severe hypoglycaemia indicated that pump+DAFNE compared to MDI+DAFNE was associated with more severe hypoglycaemic events in year 1 (IRR 1.33, 95% CI 0.49 to 3.65) but fewer events in the second year (IRR 0.35, 95% CI 0.08 to 1.44).

Base case results

Table 4 shows the base case results for the long-term cost-effectiveness analysis using the probabilistic sensitivity analysis. For the pump+DAFNE arm, the mean costs of the intervention are £42,124 discounted over the lifetime horizon, which compares with £19,829 for the MDI+DAFNE arm. This leads to an increase in intervention costs of £21,295 over a person's lifetime. The QALYs lived, prior to applying the utility decrements associated with diabetic complications, in the pump+DAFNE arm is 13.91 QALYs compared to 14.03 QALYs in the MDI+DAFNE arm, a difference of 0.11 QALYs. The differential incidence of adverse events in the pump+DAFNE arm compared to the MDI+DAFNE arm leads to a lower costs (-£371) and higher QALYs (+0.00) per person. The reduced incidence of diabetic complications in the pump+DAFNE arm leads to lower costs (-£2,070) and more QALYs (+0.01) per person. The net incremental lifetime cost of pump versus MDI is therefore estimated as £18,853 (95%CI £6,175 to £31,645) per person. The net incremental QALY gain per person is 0.13(95%CI -0.70 to +0.96) QALYs per person. The ICER associated with pump+DAFNE was £142,195 per QALY gained. This is considerable above the £30,000 per QALY threshold, which is the higher limit at which NICE would usually consider interventions to be cost-effective.[13] Figure 2a shows that this result is subject to a small degree of uncertainty, as in 14.0% of the PSA runs, pump+DAFNE would be considered as being cost-effective using the lower end of the range used by NICE.

<Table 4>

Scenario and subgroup analyses

The results of 11 scenario analyses are presented in Figure 2b. None of the scenario analyses showed pump+DAFNE to be cost-effective versus MDI+DAFNE. The lowest ICER found (£31,747 per QALY gained) was for scenario 3 in which yearly cost of pumps and consumables was halved.

<Figure 2>

For peer review only - http://bmjopen.bmj.com/site/about/guidelines.xhtml

The results of the two-way price and effectiveness threshold analysis are given in Table 5. When the annual pump cost is assumed to be 100% of the prices observed in REPOSE, then the analysis shows that the reduction in HbA1c (for pump compared to MDI) would need to be 12.0 mmol/mol (1.1%) or more, for pump to have an ICER below £20,000 per QALY gained. When the annual cost is 25% lower, then a HbA1c reduction of more than 7.7 mmol/mol (0.7%) would be needed to have an ICER below £20,000 per QALY gained. When the annual cost is halved, then a HbA1c reduction of 3.3 mmol/mol (0.3%) would be sufficient to have an ICER below £20,000 per QALY gained.

<Table 5>

The result of the subgroup analyses are presented in Figure 2c. Similarly to the scenario analysis, no subgroup was identified in which pump+DAFNE would be likely to be considered more cost-effective than MDI+DAFNE. The most cost-effective subgroup was those individuals with a baseline HbA1c greater than or equal to 80 mmol/mol (9.5%), with an ICER of £96,394 per QALY gained versus MDI+DAFNE.

DISCUSSION

This is the first study to examine the marginal benefits and cost-effectiveness of insulin delivered using pumps over MDI when both groups have had structured education, in patients without an immediate clinical need for an insulin pump, as recommended by NICE. Our findings show that providing pumps for this wider group of adults with T1DM would be unlikely to be considered cost-effective in the UK because the estimated incremental cost-effectiveness ratio was just over £142,000, substantially higher than the £30,000 per QALY upper limit often used by UK decision makers. This finding is also valid in all scenario and subgroup analyses examined. The threshold analysis indicates that if new insulin pump technology is developed and the costs are similar to current insulin pumps, a trial would have to demonstrate a HbA1c reduction in the region of 11 mmol/mol (1.0%) to 13 mmol/mol (1.2%) compared to MDI+DAFNE for the new technology to be considered cost-effective in the UK for the population analysed in this study. Conversely, if insulin pump prices were halved, then reductions of only 3 mmol/mol compared to MDI are required.

The key strengths of this study are that it is based on a rigorously conducted cluster RCT with economic data directly collected during the study. Data completeness for the primary outcome, was very high at 95%. The study uses an individual level simulation model of type 1 diabetes disease progression over a lifetime horizon. The study does have limitations in terms of the evidence used to inform the long term changes in HbA1c, which were based on 5 observational studies with follow up ranging from 3.7 to 10 years, rather than trials with long follow up periods. Also, as REPOSE is the first study to assess the effectiveness of pumps+DAFNE, the long term evidence was based on observational studies of pumps in which the education component is likely to have been different from the DAFNE structured education provided in the REPOSE trial. Our probabilistic sensitivity analysis has incorporated uncertainty around these estimates. Secondly, the analysis of rates of DKA was based on self-reported occurrence of DKA in REPOSE (the same method as used in other recent trials of DAFNE Heller *et al* Chapter 8[14]), rather than corroborated data on inpatient admissions which had a smaller number of events and could not be analysed statistically. Thirdly, only one pump type was assessed in the trial, and some caution may be needed when considering extrapolating results to modern pumps with auto-suspend features. Fourthly, the results of the threshold analysis

For peer review only - http://bmjopen.bmj.com/site/about/guidelines.xhtml

used to determine what the effectiveness of pumps would need to be considered cost-effective, should be interpreted with some degree of caution for two reasons. Firstly, we used a fixed HbA1c effect for every individual, rather than a method which accounts for heterogeneous treatment responses and secondly we assume switchers from MDI to pump get the fixed HbA1c reduction and switchers from pump to MDI get the same fixed HbA1c increase. In an ITT analysis where switchers are included in their originally randomised arms, smaller HbA1c reductions than the ones in this analysis, would likely lead to the same ICERs. Finally, as no one study could provide sufficient information on the disutility decrements associated with diabetic events for people with type 1 diabetes the data comes from disparate sources. This means that the magnitude of some events appears to be small compared to others. For example end stage renal disease has a utility decrement of -0.078 whereas heart failure has a higher decrement of -0.101.

A 2015 systematic review on the cost-effectiveness of insulin in various countries identified four studies from the UK, three of which presented an ICER.[34] The base case ICERs in these three studies were £11,461, £25,648 and £37,712, which indicate that in two out of the three studies that pumps had an ICER within/below the threshold range usually used by NICE to determine the cost-effectiveness of technologies in the UK. However, these studies most of the previous cost-effectiveness analyses used a reduction in Hba1c of 10-13 mmol/mol (0.95 – 1.2%) associated with pumps compared to MDI, based on the meta-analysis by Weissberg-Benchell *et al.* [35], which included a mixture of RCTs and observational studies. This effect size is much larger than that observed in the REPOSE trial. Some of the effect size in the meta-analysis may be related to the education offered to people in the pump arms, which was not offered to people in the MDI arms and that MDI was not administered using both long and short acting analogue insulins in most studies included in the meta analysis. To our knowledge REPOSE is the first large trial in adults with Type 1 diabetes that provides evidence on pumps versus MDI (in which both long and short acting analogue insulins are used) that provides equivalent diabetes education to both trial arms. As such, this economic analysis is the first cost-effectiveness analysis relevant to assessing whether pumps should be offered at the point when adults with T1DM in the UK are eligible to receive structured education and do not have an immediate clinical need to receive a pump.

The key implication of this paper is that unless there is an immediate clinical need for using a pump, clinicians in the NHS should offer adults with T1DM a structured education course of proven benefit, prior to considering pump therapy. This is because, when pumps+DAFNE is compared to MDI+DAFNE, the incremental health benefits are relatively small and the incremental lifetime costs are relatively high. This indicates that it would not be cost-effective to allow all adults with T1DM who are eligible for structured education, and have no clinical need for a pump, to also immediately receive an insulin pump.

A question not addressed by the REPOSE trial is whether pumps would be clinically effective and cost-effective in patients offered the technology at some point after having attended a structured education course. Generating this evidence would require a clinical study, which recruited participants who had previously received a structured education course of proven benefit and randomising them to either continued MDI or pumps. Whilst the current literature does provide some indication of which individuals may benefit from pumps, the clinical evidence informing these studies was not limited to those individuals who had first received structured education. This is important because the effectiveness of insulin pumps would be expected to differ in this group, with

For peer review only - http://bmjopen.bmj.com/site/about/guidelines.xhtml

this being related to observed and unobserved patient characteristics. One particular hypothesis that we believe is worthwhile investigating is whether pumps should be used in the UK by those adults with T1DM who actively self-manage after attending a structured education course, but either have not achieved the NICE target HbA1c levels of less than or equal to 48mmol/mol (6.5%) or who have problematic hypoglycaemia. Another important research question concerns the effectiveness and cost-effectiveness of support programmes post-education, so that more adults with T1DM achieve glycaemic targets. The DAFNEplus NIHR programme grant, which will report in 2022, is developing and testing an adapted DAFNE course (based on previous research, behaviour change theory and technological support) and subsequent structured support to improve self-management and glucose control.[36]

In conclusion, the results indicate that it would not be cost-effective to offer pumps to all adults with T1DM in the UK, who are currently eligible to receive a structured education course and do not have an immediate clinical need for a pump in the UK. Use of MDI+DAFNE is estimated to represent a better use of NHS resources than immediate commencement on a pump. Further research is required to improve the glycaemic control of adults with T1DM in the UK.

For peer review only - http://bmjopen.bmj.com/site/about/guidelines.xhtml

Funding

This research was funded by the UK Health Technology Assessment Programme (project number 08/107/01). We also acknowledge the financial support of the Research and Development Programmes of the Department of Health for England and the Scottish Health and Social Care Directorates who supported the costs of consumables and of Medtronic UK Ltd., who provided the insulin pumps for the trial. The funders had no involvement in the design of the protocol, the collection, analysis and interpretation of the data, in the writing of this paper, or in the decision to submit this article for publication. The views and opinions expressed herein are those of the authors and do not necessarily reflect those of the HTA, NIHR, NHS, the Department of Health or Medtronic UK Ltd.

Conflicts of Interest

SH reports personal fees from Sanofi Aventis and personal fees and other from NovoNordisk and Eli Lilly, outside the submitted work. JE reports personal fees from Astra Zeneca, Merck Sharpe Dohme and Takeda, personal fees and non-financial support from Eli Lilly, Novonordisk and Sanofi, outside the submitted work. NW reports receiving two days of consultancy fees from Novo Nordisk on topics including alternatives to network meta-analysis, unrelated to REPOSE. No other authors have any conflicts of interest to report.

Contributions

Author contributions

DP contributed to the design of all of the health economic analyses and conducted all statistical and modelling analyses used in this paper. AB, oversaw the design and implementation of the modelling analyses. SD, oversaw the design and implementation of the economic analysis of the trial data and contributed to the estimation of the treatment costs used in the long term economic analyses. AB, SD, NW, JE, SH, MC contributed to the design of the trial. NW, JE, SH provided clinical input into the design of the economic analyses. MC, designed the statistical analysis plan. EL, implemented the statistical analysis plan, parts of which were used in the economic evaluation. HB, DW contributed to data collection for the economic analyses. AB, HB designed and developed previous versions of the economic model used in these analyses. DP wrote the first draft. All authors approved the final draft. DP is the guarantor.

Acknowledgements

Richard Jacques, Mónica Hernández Alava, for their advice on how to conduct the statistical analysis on HbA1c.

Peter Mansell for providing helpful comments on the full report.

The research was sponsored by Sheffield Teaching Hospitals NHS Foundation Trust. The clinical sites taking part were: Cambridge University Hospitals NHS Foundation Trust, Dumfries and Galloway Royal Infirmary, NHS Greater Glasgow and Clyde, Harrogate and District NHS Foundation Trust, King's College Hospital NHS Foundation Trust, NHS Lothian, Nottingham University Hospitals NHS Trust and Sheffield Teaching Hospitals NHS Foundation Trust.

For peer review only

For peer review only - http://bmjopen.bmj.com/site/about/guidelines.xhtml

Members of the REPOSE study group

Simon Heller was the chief investigator. Norman Waugh was the deputy chief investigator. Stephanie Amiel, Mark Evans, Fiona Green, Peter Hammond, Alan Jaap, Brian Kennon, Robert Lindsay and Peter Mansell were site principal investigators and contributed to the study design and data interpretation.

Jane Baillie, Anita Beckwith, Helen Brown, Karen Callaby, Katy Davenport, Sarah Donald, Jackie Elliott, Leila Faghahati, Sara Hartnell, Allison Housden, Kalbir Kaur Pabla, Nicola Croxon, Sheena Macdonald, Muna Mohammed, Vicky Steel, Katy Valentine, Pamela Young, Ann Boal, Patsy Clerkin, Lynn Doran, Joanne Flynn, Emma Gibb, Hilary Peddie, Bernie Quinn, Helen Rogers, Janice Shephard, Janet Carling, Ann Collins, Laura Dinning, Christine Hare, Joyce Lodge, Sutapa Ray, Debora Brown, Jenny Farmer, Alison Cox, Chris Cheyette, Pratik Choudhary, Linda East, June Ellul, Katherine Hunt, Kimberley Shaw, Ben Stothard, Lucy Diamond, Lindsay Aniello, Debbie Anderson, Kathy Cockerell, Vida Heaney, Alison Hutchison, Nicola Zammitt, Gayna Babington, Gail Bird, Janet Evans, Tasso Gazis, Nicola Maude, Karen Nunnick, Dawn Spick, Laura Fenn, Carla Gianfrancesco, Valerie Gordon, Linda Greaves, Susan Hudson, Valerie Naylor, Chloe Nisbet, Carolin Taylor, Karen Towse and Candice Ward contributed at sites to participant recruitment, intervention delivery and data collection.

Cindy Cooper, Gemma Hackney, Diana Papaioannou, Emma Whatley and David White provided central trial management, oversight and monitoring. Mike Bradburn, Michael Campbell, Munya Dimairo and Ellen Lee contributed to the statistics. Hasan Basarir, Alan Brennan, Simon Dixon and Daniel Pollard contributed to the health economics. Nina Hallowell, Jackie Kirkham, Julia Lawton and David Rankin designed and undertook the qualitative work. Katharine Barnard led the quantitative psychosocial work. Timothy Chater and Kirsty Pemberton provided data management. Fiona Allsop and Lucy Carr provided central administration. Pamela Royle conducted literature searches and exploratory analyses. Gill Thompson and Sharon Walker provided central DAFNE support. Pauline Cowling conducted the fidelity assessment. Henry Smithson provided user representation on the management group.

Data Sharing

Requests for patient level data and statistical code should be made to the corresponding author and will be considered by the REPOSE trial management group who, although specific consent for data sharing was not obtained, will release data on a case by case basis following the principles for sharing patient level data as described by Smith et al (2015). The presented data do not contain any direct identifiers, we will minimise indirect identifiers and remove free text data, to minimise the risk of identification.

For peer review only

For peer review only - http://bmjopen.bmj.com/site/about/guidelines.xhtml

For peer review only

For peer review only - http://bmjopen.bmj.com/site/about/guidelines.xhtml

References

1. The Diabetes Control and Complications Trial Research Group. The effect of intensive treatment of diabetes on the development and progression of long-term complications in insulin-dependent diabetes mellitus. . *N Engl J Med* 1993;329(14):977-86. doi: 10.1056/NEJM199309303291401
2. Dafne Study Group. Training in flexible, intensive insulin management to enable dietary freedom in people with type 1 diabetes: dose adjustment for normal eating (DAFNE) randomised controlled trial. *BMJ* 2002;325(7367):746.
3. Muhlhauser I, Bruckner I, Berger M, et al. Evaluation of an intensified insulin treatment and teaching programme as routine management of type 1 (insulin-dependent) diabetes. The Bucharest-Dusseldorf Study. *Diabetologia* 1987;30(9):681-90.
4. National Institute for Health and Care Excellence. Type 1 diabetes in adults: diagnosis and management. Clinical guideline NG17. *Online Source Available from: http://wwwniceorguk/guidance/ng17/evidence/full-guideline-435400241* 2015;Last Accessed 21st January 2016
5. National Institute for Health and Care Excellence. Continuous subcutaneous insulin infusion for the treatment of diabetes mellitus: NICE technology appraisal guidance [TA 151]. *Online Source* 2008;Available from; https://www.nice.org.uk/guidance/ta151. (Last Accessed 21st January 2016)
6. Hospital and Social Care Information Centre. National Insulin Pump Audit Report, 2013-15: England. 2016
7. Pickup J. Insulin pumps. *Int J Clin Pract Suppl* 2011(170):16-9. doi: 10.1111/j.1742-1241.2010.02574.x
8. Pickup JC. Are insulin pumps underutilized in type 1 diabetes? Yes. *Diabetes Care* 2006;29(6):1449-52. doi: 10.2337/dc06-0011
9. Cummins E, Royle P, Snaith A, et al. Clinical effectiveness and cost-effectiveness of continuous subcutaneous insulin infusion for diabetes: systematic review and economic evaluation. *Health Technol Assess* 2010;14(11):iii-iv, xi-xvi, 1-181. doi: 10.3310/hta14110
10. Colquitt JL, Green C, Sidhu MK, et al. Clinical and cost-effectiveness of continuous subcutaneous insulin infusion for diabetes. *Health Technol Assess* 2004;8(43):iii, 1-171.
11. Leotta SA, Altomare M, Carletti S, et al. Intensive diabetes management in type 1 diabetic patients in poor glycemic control treated with insulin pump therapy or multiple daily injections: Two years follow-up. *4th International Conference on Advanced Technologies and Treatments for Diabetes*;London, 2011
12. White D, Waugh N, Elliott J, et al. The Relative Effectiveness of Pumps Over MDI and Structured Education (REPOSE): study protocol for a cluster randomised controlled trial. *BMJ Open* 2014;4(9):e006204. doi: 10.1136/bmjopen-2014-006204
13. National Institute for Health and Care Excellence. Guide to the methods of technology appraisal 2013. *Online Source* 2013;Available from; http://www.nice.org.uk/article/pmg9/resources/non-guidance-guide-to-the-methods-of-technology-appraisal-2013-pdf. Last accessed 13th July 2015.
14. Heller S, Lawton J, Amiel S, et al. Improving management of type 1 diabetes in the UK: the Dose Adjustment For Normal Eating (DAFNE) programme as a research test-bed. A mixed-method analysis of the barriers to and facilitators of successful diabetes self-management, a health economic analysis, a cluster randomised controlled trial of different models of delivery of an educational intervention and the potential of insulin pumps and additional educator input to improve outcomes. *Programme grants for applied research* 2014;2(5) doi: 10.3310/pgfar02050
15. Thokala P, Kruger J, Brennan A, et al. Assessing the cost-effectiveness of type 1 diabetes interventions: the Sheffield type 1 diabetes policy model. *Diabet Med* 2014;31(4):477-86. doi: 10.1111/dme.12371

For peer review only - http://bmjopen.bmj.com/site/about/guidelines.xhtml

16. SIMUL8 corporation. SIMUL8 professional 2010. Boston2010.
17. Groot Koerkamp B, Stijnen T, Weinstein MC, et al. The combined analysis of uncertainty and patient heterogeneity in medical decision models. *Med Decis Making* 2011;31(4):650-61. doi: 10.1177/0272989X10381282 [published Online First: 2010/10/27]
18. The Repose Study Group. Relative effectiveness of insulin pump treatment over multiple daily injections and structured education during flexible intensive insulin treatment for type 1 diabetes: cluster randomised trial (REPOSE). *BMJ* 2017;356:j1285. doi: 10.1136/bmj.j1285 [published Online First: 2017/04/01]
19. Statacorp. Stata Statistical Software. Release 13.1. College Station, Texas: Stata Press 2013.
20. Kaplan EL, Meier P. Nonparametric-Estimation from Incomplete Observations. *J Am Stat Assoc* 1958;53(282):457-81. doi: Doi 10.2307/2281868
21. Collett D. Survival Data in Medical Research. 2nd ed. Boca Ration (FL): Chapman & Hall / CRC 2003.
22. Cribari-Neto F, Zeileis A. Beta Regression in R. *J Stat Softw* 2010;34(2):1-24.
23. Orr CJ, Hopman W, Yen JL, et al. Long-term efficacy of insulin pump therapy on glycemic control in adults with type 1 diabetes mellitus. *Diabetes Technol Ther* 2015;17(1):49-54. doi: 10.1089/dia.2014.0131
24. Carlsson BM, Attvall S, Clements M, et al. Insulin pump-long-term effects on glycemic control: an observational study at 10 diabetes clinics in Sweden. *Diabetes Technol Ther* 2013;15(4):302-7. doi: 10.1089/dia.2012.0286
25. Bruttomesso D, Pianta A, Crazzolara D, et al. Continuous subcutaneous insulin infusion (CSII) in the Veneto region: efficacy, acceptability and quality of life. *Diabet Med* 2002;19(8):628-34.
26. Gunn D, Mansell P. Glycaemic control and weight 7 years after Dose Adjustment For Normal Eating (DAFNE) structured education in Type 1 diabetes. *Diabetic Med* 2012;29(6):807-12.
27. Speight J, Amiel SA, Bradley C, et al. Long-term biomedical and psychosocial outcomes following DAFNE (Dose Adjustment For Normal Eating) structured education to promote intensive insulin therapy in adults with sub-optimally controlled Type 1 diabetes. *Diabetes Res Clin Pract* 2010;89(1):22-9. doi: 10.1016/j.diabres.2010.03.017
28. Curtis L. Unit Costs of Health & Social Care 2014. *Online Source* 2014;Available from; http://www.pssru.ac.uk/project-pages/unit-costs/2014/index.php. Last Accessed 30th June 2015
29. British Medical Association and Royal Pharmaceutical Society of Great Britain. British National Formulary August 2015. *Online Source* 2015;Available from: https://www.medicinescomplete.com, Last Accessed 9th September 2015
30. Department of Health. NHS reference costs 2013 to 2014. *Online Source* 2014;Available from; https://www.gov.uk/government/publications/nhs-reference-costs-2013-to-2014. Last accessed 17th July 2015
31. Health & Social Care Information Centre. Prescription Cost Analysis England 2014. *Online Source* 2015;Available from; http://www.hscic.gov.uk/catalogue/PUB17274. Last Accessed 2nd Novemeber 2015
32. R Core Team. R: A language and environment for statistical computing. R Foundation for Statistical Computing. *Vienna, Austria* 2015;URL https://www.R-project.org/.
33. Brown MM, Brown GC, Sharma S, et al. Quality of life with visual acuity loss from diabetic retinopathy and age-related macular degeneration. *Arch Ophthalmol* 2002;120(4):481-4.
34. Roze S, Smith-Palmer J, Valentine W, et al. Cost-effectiveness of continuous subcutaneous insulin infusion versus multiple daily injections of insulin in Type 1 diabetes: a systematic review. *Diabetic Med* 2015;32(11):10. doi: DOI: 10.1111/dme.12792
35. Weissberg-Benchell J, Antisdel-Lomaglio J, Seshadri R. Insulin pump therapy: a meta-analysis. *Diabetes Care* 2003;26(4):1079-87.
36. National Institute for Health Research. Programme Grants for Applied Research. Active, completed and closed programme grants (last updated June 2017). *Online Source* 2017;Last

For peer review only

For peer review only - http://bmjopen.bmj.com/site/about/guidelines.xhtml

Accessed 15th November 2017(Availble from: https://www.nihr.ac.uk/research-and-impact/documents/Funded%20studies/PGfAR%20funded%20projects%20June%202017.xlsx)
37. DAFNE. DAFNE Fact Sheet Six. *Online Source* 2012;Available from; http://www.dafne.uk.com/uploads/135/documents/06_factsheetsix_12pt_18_06_12.pdf. Last Accessed 1st July 2015
38. Department of Health. NHS Reference Costs 2010-11. London: Department of Health 2011.
39. British Medical Association and Royal Pharmaceutical Society of Great Britain. British National Formulary. 63rd edn ed. London: BMA and RPS 2012.
40. McEwan P, Poole CD, Tetlow T, et al. Evaluation of the cost-effectiveness of insulin glargine versus NPH insulin for the treatment of type 1 diabetes in the UK. *Curr Med Res Opin* 2007;23:S7-S19. doi: 10.1185/030079906x167561
41. Currie CJ, Poole CD, Woehl A, et al. The financial costs of healthcare treatment for people with Type 1 or Type 2 diabetes in the UK with particular reference to differing severity of peripheral neuropathy. *Diabet Med* 2007;24(2):187-94. doi: 10.1111/j.1464-5491.2006.02057.x
42. National Institute for Health and Care Excellence. Ranibizumab for treating diabetic macular oedema (rapid review of technology appraisal guidance 237). NICE technology appraisal guidance 274. *Onlince Source* 2013;Available from: https://www.nice.org.uk/guidance/ta274/resources/ranibizumab-for-treating-diabetic-macular-oedema-rapid-review-of-technology-appraisal-guidance-237-82600612458181
43. British Medical Association and Royal Pharmaceutical Society of Great Britain. British National Formulary August 2016. *Online Source* 2016;Available from: https://www.medicinescomplete.com, Last Accessed 12th December 2016
44. Clarke P, Gray A, Legood R, et al. The impact of diabetes-related complications on healthcare costs: results from the United Kingdom Prospective Diabetes Study (UKPDS Study No. 65). *Diabet Med* 2003;20(6):442-50.
45. Peasgood T, Brennan A, Mansell P, et al. The impact of diabetes-related complications on preference-based measures of health-related quality of life in adults with Type I diabetes. *Medical Decision Making* 2016;In Press doi: DOI: 10.1177/0272989X16658660
46. Coffey JT, Brandle M, Zhou H, et al. Valuing health-related quality of life in diabetes. *Diabetes Care* 2002;25(12):2238-43.
47. Alva M, Gray A, Mihaylova B, et al. The effect of diabetes complications on health-related quality of life: the importance of longitudinal data to address patient heterogeneity. *Health Econ* 2014;23(4):487-500. doi: 10.1002/hec.2930
48. Clarke P, Gray A, Holman R. Estimating utility values for health states of type 2 diabetic patients using the EQ-5D (UKPDS 62). *Med Decis Making* 2002;22(4):340-9.

For peer review only - http://bmjopen.bmj.com/site/about/guidelines.xhtml

Tables

Table 1: The baseline characteristics of REPOSE participants and the simulated cohort

Characteristic	REPOSE trial population (N=260)	Simulated cohort (N=5,000)
**Continuous variables mean (standard deviation) [% of individuals with data prior to imputation]**		
Baseline HbA1c (mmol/mol)	76.0 (18.6) [100%]	75.9 (18.2) [100%]
Baseline HbA1c (%)	9.1 (1.7) [100%]	9.1 (1.7) [100%]
Age (years)	40.4 (13.4) [100%]	40.4 (13.3) [100%]
Diabetes duration (years)	18.0 (12.5) [100%]	18.0 (12.3) [100%]
Triglycerides (mmol/mol)	1.4 (1.0) [100%]	1.4 (0.9) [100%]
Total Cholesterol (mmol/mol)	4.9 (0.9) [99.6%]	4.9 (0.9) [99.7%]
HDL cholesterol (mmol/mol)	1.6 (0.4) [96.5%]	1.6 (0.4) [96.4%]
LDL cholesterol (mmol/mol)	2.8 (0.9) [96.2%]	2.7 (0.9) [96.1%]
Systolic blood pressure	131.4 (16.4) [98.8%]	131.3 (16.0) [98.9%]
Baseline cost of insulin	£357.24 (147.65) [94.8%]	£360.39 (157.92) [98.4%]
Baseline cost of diabetes related contacts	£561.61 (885.92) [100%]	£571.63 (928.92) [100%]
**Categorical variables n/N (percentage) [% of individuals prior to imputation]**		
Gender		
Female	104/260 (40.0%) [40.0%]	1990/5000 (39.8%) [39.3%]
Male	152/260 (58.5%) [58.5%]	2950/5000 (59.0%) [59.3%]
Missing	4/260 (1.5%) [1.5%]	0/5000 (0.0%) [1.4%]
Physical Activity		
Low	67/260 (25.8%) [25.8%]	1266/5000 (25.3%) [25.3%]
Medium	128/260 (49.2%) [49.2%]	2471/5000 (49.4%) [49.4%]
High	65/260 (25.0%) [25.0%]	1263/5000 (25.3%) [25.3%]
Smoking Status		
Current	50/260 (19.2%) [19.2%]	951/5000 (19.2%) [19.2%]
Former	67/260 (25.8%) [25.8%]	1325/5000 (26.3%) [26.3%]
Never	143/260 (55.0%) [55.0%]	2724/5000 (54.5%) [54.5%]
Race		
White	258/260 (99.2%) [99.2%]	4959/5000 (99.2%) [99.2%]
Black	2/260 (0.8%) [0.8%]	41/5000 (0.8%) [0.8%]
Nephropathy		
No complications	239/260 (91.9%) [91.9%]	4600/5000 (92.0%) [92.0%]
Microalbuminuria	13/260 (5.0%) [5.0%]	234/5000 (4.7%) [4.7%]
Macroalbuminuria	7/260 (2.7%) [2.7%]	152/5000 (3.0%) [3.0%]
Dialysis or transplant	1/260 (0.4%) [0.4%]	14/5000 (0.4%) [0.4%]
Neuropathy		
No complications	238/260 (91.5%) [91.5%]	4599/5000 (92.0%) [92.0%]
Neuropathy or Ulcers	22/260 (8.5%) [8.5%]	401/5000 (8.0%) [8.0%]
Retinopathy		
No complications	145/260 (55.8%) [55.8%]	28/5000 (56.0%) [56.0%]
Background diabetic retinopathy	91/260 (35.0%) [35.0%]	1740/5000 (34.8%) [34.8%]
Proliferative diabetic retinopathy	24/260 (9.2%) [9.2%]	465/5000 (9.3%) [9.3%]
Myocardial Infarction		
No complications	255/260 (98.1%) [98.1%]	4896/5000 (97.9%) [97.9%]
Myocardial Infarction	5/260 (1.9%) [1.9%]	104/5000 (2.1%) [2.1%]
Stroke		
No complications	259/260 (99.6%) [99.6%]	4983/5000 (99.7%) [99.7%]
Stroke	1/260 (0.4%) [0.4%]	17/5000 (0.3%) [0.3%]
Heart Failure		
No complications	259/260 (99.6%) [99.6%]	4934/5000 (99.6%) [99.6%]
Heart Failure	1/260 (0.4%) [0.4%]	18/5000 (0.4%) [0.4%]
Angina		
No complications	257/260 (98.9%) [98.9%]	4934/5000 (98.7%) [98.7%]
Angina	3/260 (1.2%) [1.2%]	66/5000 (1.3%) [1.3%]

HDL; high density lipoprotein, LDL Low-density lipoprotein

For peer review only

Table 2: The health state cost parameters used in the Sheffield Type 1 Diabetes Policy Model

Part A: Seemingly unrelated regression functions for estimated costs in year 1 and ongoing based on REPOSE trial data (Multivariate normal distributions*)

	Annual Cost of Insulin and MDI consumables (year 1)	Annual Cost of Insulin and MDI consumables (ongoing)	Annual Cost of DRC (year 1)	Annual Cost of DRC (ongoing)	Annual Cost of Insulin Pump and associated consumables (year 1)	Annual Cost of Insulin Pump and associated consumables (ongoing)	**Sources**
Multiplier for the baseline DRC cost (β_1)	-	-	0.11	0.03	-	-	REPOSE trial data in year 1. (year 1 costs)<br>REPOSE trial data in year 2. (ongoing costs)<br>NHS reference costs[30]<br>Pump costing survey
Multiplier for the baseline insulin cost (β_2)	0.97	1.04	-	-	-	-	
Multiplier for the Baseline HbA1c (DCCT % scale) (β_3)	5.08	12.81	-21.66	12.15	-	-	
Receiving pump at the start of the year (β_4)	-517.91	-527.64	129.08	88.99	2056.11	2050.99	
Switch from pump to MDI (β_5)	554.47	153.35	280.16	-47.10	-1143.68	-905.03	
Switch from MDI to pump (β_6)	0[†]	-353.27	733.95	201.93	804.57	1134.27	
Constant (β_0)	381.77	324.53	415.46	299.80	0.00	0.00	

The cost for each total cost in part A is calculated using the following formula:
Total cost = β_0 + β_1*Individual's baseline diabetes related contact cost + β_2*Individual's baseline insulin cost + β_3*Individual's baseline HbA1c (DCCT % scale) + β_4 * Individual's treatment at the start of the year (1=pump, 0=MDI) + β_5*(1 = switched from pump to MDI, 0 = didn't switch from pump to MDI) + β_6*(1= Switched from MDI to pump, 0 =didn't switch from MDI to pump)

*, the variance covariance matrices used to parameterise the multivariate normal distribution are provided in supplementary material B Tables 4, 7 and 12.

-, this value was not included as a covariate in the regression formula and is taken to be a zero value in the total cost formula

†; this variable was included in the original regressions, however the model would converge when this covariate was included, so this parameters is taken to be a zero value in the total cost formula

Part B: Costs of DAFNE Fixed parameters

**Health State**	**Mean cost (£)**	**SE**	**Source**	**Health State**	**Mean cost (£)**	**SE**	**Source**
Cost of a DAFNE course	363.10	None	DAFNE Fact Sheet Six[37]	Cost of pre-course fitting session for pump users	28.82	None	DAFNE Fact Sheet Six [37] & REPOSE trial data

Part C: Costs of adverse events, comorbidities and complications Gamma Distributions

**Health State**	**Mean Cost (£)**	**SE**	**Source**	**Health State**	**Mean cost (£)**	**SE**	**Source**
**Adverse Events**							
Hypoglycaemia	187	18.69	Heller *et al.*[14]	DKA with Hospitalisation	1399	140	NHS reference costs[38]
**Nephropathy**							
Ongoing yearly cost of Microalbuminuria	36	3.56	BNF[39] and McEwan *et al.*[40]	Ongoing yearly cost of Microalbuminuria ongoing	36	3.56	BNF[39] and McEwan *et al.*[40]

For peer review only

For peer review only - http://bmjopen.bmj.com/site/about/guidelines.xhtml

Ongoing yearly cost of ESRD	24436	2444	NHS Reference costs[38]	Death due to ESRD	0	0	Assumed equal to Zero
**Neuropathy**							
Clinically Confirmed Neuropathy	271	27.14	Currie *et al.*[41]	Clinical Neuropathy	271	27.14	Currie *et al.*[41]
Diabetic foot syndrome	2848	285	NHS Reference costs[38]	PAD with amputation (year 1)	7221	722	NHS Reference costs[38]
Ongoing Yearly cost of PAD with amputation	439	43.93	McEwan *et al.*[40]				
**Retinopathy**							
Background Retinopathy	145	14.47	McEwan *et al.*[40]	Proliferative Retinopathy	661	66.11	McEwan *et al.*[40]
Macular edema (year 1)	5710	571.0	NICE[42], BNF[43]	Macular edema (year 2)	3416	341.6	NICE[42], BNF[43]
Macular edema (year 3)	2562	256.2	NICE[42], BNF[43]	Macular edema (Ongoing)	277	27.7	NICE[42], BNF[43]
Blindness (year 1)	1584	158	Clarke *et al.*[44]	Blindness (ongoing)	519	51.88	Clarke *et al.*[44]
**Cardiovascular**							
First MI (year 1)	6788	679	Clarke *et al.*[44]	Second MI (year 1)	6788	679	Clarke *et al.*[44]
Final MI (year 1)	6788	679	Clarke *et al.*[44]	Ongoing yearly cost of a MI	904	90.43	Clarke *et al.*[44]
Fatal MI	2101	210	Clarke *et al.*[44]	Heart Failure (year 1)	3818	382	Clarke *et al.*[44]
Heart Failure (ongoing)	1173	117	Clarke *et al.*[44]	Fatal HF	3818	382	Clarke *et al.*[44]
First Stroke (year 1)	4361	436	Clarke *et al.*[44]	Second Stroke	4361	436	Clarke *et al.*[44]
Fatal Stroke	5684	568.45	Clarke *et al.*[44]	First Stroke (ongoing)	559	55.90	Clarke *et al.*[44]
Angina (year 1)	3397	340	Clarke *et al.*[44]	Angina (ongoing)	951	95.09	Clarke *et al.*[44]

BNF, British National Formulary; ESRD, End-Stage Renal Disease; PAD, Peripheral Arterial Disease; DAFNE, Dose Adjustment for Normal Eating; pump, insulin pumps; MDI, multiple daily injections

For peer review only

Table 3: The base case utility parameters

Health state for event	Utility	SE	Beta distribution *Alpha*	*Beta*	Source
Baseline utility value					
Male with type 1 diabetes and no complications	0.866	0.010	947.79	146.90	Peasgood *et al.*[45]
	**Disutility**	**SE**	**Gamma distribution *Alpha***	***Beta***	**Source**
Complications or covariates					
Female with type 1 diabetes and no complications	0.0236	0.008	8.70	0.003	Peasgood *et al.*[45]*
**Adverse events‡**					
Severe Hypoglycaemia	-0.002	-0.002	1	0.002	Peasgood *et al.*[45]
Diabetic Ketoacidosis	-0.0091	-0.01	0.83	0.01	Peasgood *et al.*[45]*
**Nephropathy‖**					
Microalbuminuria	0				Assumption
Microalbuminuria	-0.017	0.01	2.89	0.01	Coffey *et al.*[46]
ESRD	-0.078	0.026	9	0.01	Coffey *et al.*[46]
**Neuropathy‖ (applied to the history of events)**					
Clinical neuropathy	-0.055	0.01	30.25	0.002	Coffey *et al.*[46]
Clinically confirmed neuropathy	-0.055	0.01	30.25	0.002	Coffey *et al.*[46]
Diabetic foot syndrome	-0.1042	-0.119	0.77	0.14	Peasgood *et al.*[45]
PAD with amputation	-0.1172	-0.055	4.54	0.03	Peasgood *et al.*[45]*
**Retinopathy‖ (applied to the history of events)**					
Background retinopathy	-0.0544	-0.023	5.59	0.01	Peasgood *et al.*[45]
Proliferative retinopathy	-0.0288	-0.026	1.23	0.02	Peasgood *et al.*[45]
Blindness	-0.208	0.013	256	0.001	Coffey *et al.*[46]
**Cardiovascular‖ (applied to the history of events)**					
MI (first year)	-0.065	0.03	4.69	0.01	Alva *et al.*[47]
MI (subsequent years)	-0.057	0.03	3.61	0.02	Alva *et al.*[47]
Heart failure	-0.101	0.032	9.96	0.010	Alva *et al.*[47]
Stroke	-0.165	0.035	22.22	0.007	Alva *et al.*[47]
Angina	-0.09	0.018	25	0.004	Clarke *et al.*[48]†

SE – standard error; ESRD – end stage renal disease; PAD – peripheral arterial disease; MI – myocardial infarction
* - a parameter value was not available in the author's preferred statistical model; † - value is presented in Table 5 as ischaemic heart disease. ‡ - these disutilites are applied transiently to the number of these events in each year. ‖ - these disutilites are applied to the history of ever having had one of these events

For peer review only - http://bmjopen.bmj.com/site/about/guidelines.xhtml

Table 4: Long-Term Cost-Effectiveness Analysis - base case results using probabilistic sensitivity analysis

	MDI+DAFNE	pump+DAFNE	Incremental
**Mean Lifetime Discounted Costs per Person**			
Intervention costs			
Insulin and MDI consumables	£12,215	£5,476	-£6,740
Diabetes Related contacts	£5,023	£6,289	£1,266
Insulin pumps and pump consumables	£2,228	£28,967	£26,739
DAFNE course	£363	£392	£29
**Subtotal Intervention costs**	£19,829	£41,124	£21,295
Adverse event costs			
Severe Hypoglycaemia	£133	£41	-£92
Diabetic Ketoacidosis	£1,161	£882	-£279
**Subtotal cost of adverse events**	£1,294	£922	-£371
Long term complication costs			
Nephropathy	£40,786	£38,853	-£1,933
Neuropathy	£1,859	£1,805	-£53
Retinopathy + Macular Edema	£6,365	£6,263	-£102
Myocardial Infarction	£1,838	£1,844	£6
Heart Failure	£607	£609	£2
Stroke	£253	£254	£0
Angina	£1,134	£1,143	£8
**Total cost of long term complications**	£52,841	£50,771	-£2,070
**Total costs**	£73,964	£92,817	£18,853<br>(95% CI £6,175, £31,645)
**Mean undiscounted life years per person**			
**Total Life years**	28.3181	28.7999	0.3790<br>(95% CI -2.7392 to 3.3403)
**Mean Discounted QALYs per person**			
QALYs lived<br>(excluding decrements due to complications)	13.9145	14.0292	0.1147
QALYs lost due to adverse events			
Severe Hypoglycaemia	-0.0014	-0.0004	0.0009
Diabetic Ketoacidosis	-0.0075	-0.0057	0.0018
**Subtotal QALYs due to adverse events**	-0.0088	-0.0061	0.0027
QALYs lost due to complications			
Nephropathy	-0.1853	-0.1792	0.0061
Neuropathy	-0.3092	-0.3010	0.0082
Retinopathy + Macular Edema	-0.3316	-0.3293	0.0022
Myocardial Infarction	-0.0528	-0.0532	-0.0004
Heart Failure	-0.0385	-0.0387	-0.0002
Stroke	-0.0343	-0.0345	-0.0002
Angina	-0.0754	-0.0761	-0.0007
**Subtotal QALYs lost due to complications**	-1.0271	-1.0120	0.0152
**Total QALYs**	12.8785	13.0111	0.1326<br>(95%CI-0.7087, 0.9623)
**Summary**			
Total mean discounted costs per person	£80,471	£99,337	£18,853<br>(95% CI £6,175, £31,645)
Total mean undiscounted life years per person	28.3181	28.7999	0.3790<br>(95% CI -2.7392 to 3.3403)
Total mean discounted QALYs per person	12.8785	13.0111	0.1326<br>(95%CI-0.7087, 0.9623)
ICER (£/QALY gained)			£142,195
Probability that pump+DAFNE is cost-effective at a threshold of £20,000 per QALY gained			14.0%
MDI – multiple daily injections; DAFNE – dose adjustment for normal eating; pump – insulin pumps; CI – confidence interval; QALY – quality adjusted life year; ICER – incremental cost-effectiveness ratio			

For peer review only - http://bmjopen.bmj.com/site/about/guidelines.xhtml

Table 5: The incremental cost-effectiveness ratio associated with insulin pumps for different HbA1c reductions (for all adults with T1DM) and annualised prices of insulin pumps and insulin pump consumables

	3.3 mmol/mol (0.3%)	4.4 mmol/mol (0.4%)	5.5 mmol/mol (0.5%)	6.6 mmol/mol (0.6%)	7.7 mmol/mol (0.7%)	8.7 mmol/mol (0.8%)	9.8 mmol/mol (0.9%)	10.9 mmol/mol (1.0%)	12.0 mmol/mol (1.1%)	13.1 mmol/mol (1.2%)
£2,060 (100%)	£75,710	£85,990	£64,493	£47,667	£34,280	£27,951	£25,979	£26,258	£18,953	£17,610
£1,957 (95%)	£69,830	£79,106	£58,901	£43,527	£31,132	£25,128	£23,494	£23,736	£16,730	£15,515
£1,854 (90%)	£63,950	£72,222	£53,309	£39,387	£27,985	£22,306	£21,009	£21,213	£14,507	£13,419
£1,751 (85%)	£58,070	£65,338	£47,717	£35,247	£24,837	£19,483	£18,524	£18,691	£12,284	£11,323
£1,648 (80%)	£52,189	£58,454	£42,125	£31,107	£21,689	£16,661	£16,039	£16,169	£10,060	£9,227
£1,545 (75%)	£46,309	£51,570	£36,533	£26,967	£18,542	£13,838	£13,554	£13,646	£7,837	£7,131
£1,442 (70%)	£40,429	£44,686	£30,941	£22,827	£15,394	£11,016	£11,070	£11,124	£5,614	£5,036
£1,339 (65%)	£34,549	£37,802	£25,349	£18,687	£12,246	£8,193	£8,585	£8,602	£3,391	£2,940
£1,236 (60%)	£28,668	£30,917	£19,757	£14,547	£9,099	£5,370	£6,100	£6,079	£1,167	£844
£1,133 (55%)	£22,788	£24,033	£14,164	£10,407	£5,951	£2,548	£3,615	£3,557	Dominates	Dominates
£1,030 (50%)	£16,908	£17,149	£8,572	£6,267	£2,803	Dominates	£1,130	£1,035	Dominates	Dominates

For peer review only - http://bmjopen.bmj.com/site/about/guidelines.xhtml

Figure Legends

Figure 1: The trace of: a) the mean HbA1c in each year of the model, b) the mean HbA1c ignoring the effects of death and treatment switching, c) the number of people alive in each year of the model, in both arms over 30 years

Figure 2: The results of the economic analyses presented on the cost-effectiveness plane for: a) the base case probabilistic sensitivity analyses, b) the results of the scenario analyses and c) the results of the pre specified subgroup analyses

For peer review only

For peer review only - http://bmjopen.bmj.com/site/about/guidelines.xhtml

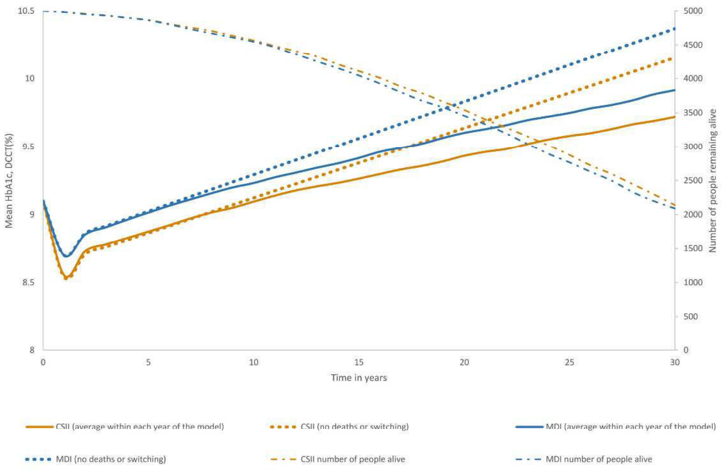

The trace of: a) the mean HbA1c in each year of the model, b) the mean HbA1c ignoring the effects of death and treatment switching, c) the number of people alive in each year of the model, in both arms over 30 years

338x190mm (300 x 300 DPI)

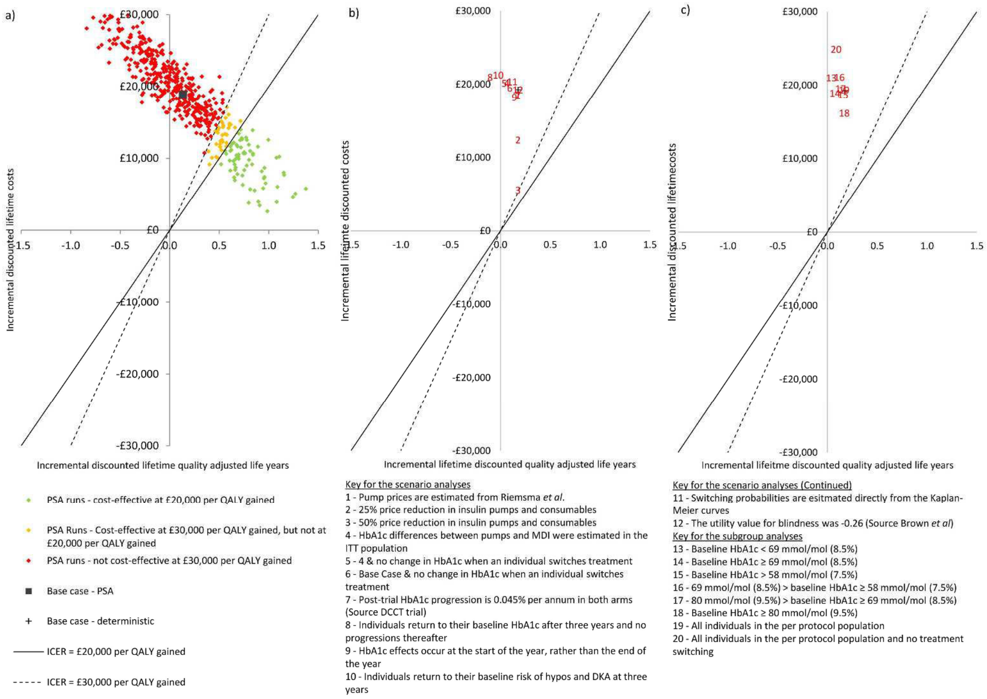

The results of the economic analyses presented on the cost-effectiveness plane for: a) the base case probabilistic sensitivity analyses, b) the results of the scenario analyses and c) the results of the pre specified subgroup analyses

297x209mm (300 x 300 DPI)

Supplementary material A – Economic Evaluation Alongside the clinical trial

Contents

1 Methods....2

1.1 Resource Use by individuals in REPOSE over the 2 year follow up period 2

1.1.1 Treatment switching....7

1.1.2 Estimating the within trial cost effects....7

1.1.3 Estimating within trial QALY effects using EQ-5D and SF-12....7

1.1.4 The statistical model used for the within trial analysis....8

1.1.5 Analysis....8

2 Results....8

2.1 Base Case analysis 8

2.1.1 Summary of the scenario analyses....11

2.1.2 Results of the scenario analyses....12

Table of tables

Table 1: The unit costs used in the within trial analysis of the REPOSE data 3

Table 2: The unit costs of insulin....5

Table 8: Within trial cost-effectiveness analysis results of continuous subcutaneous insulin infusion (pumps) versus multiple daily injections (MDI), both with dose adjustment for normal eating structured education....13

Table of figures

Figure 1: The cost-effectiveness ellipse for the base case within trial analysis....10

Figure 2: The cost-effectiveness acceptability curve for the base case within trial analysis...10

For peer review only

List of abbreviations

Abbreviation	Definition
EEACT	economic evaluation alongside the trial
DKA	diabetic ketoacidosis
QALY	Quality adjusted life year
pumps	Insulin pumps
MDI	Multiple daily injections
DAFNE	Dose adjustment for normal eating

1 Methods

1.1 Resource Use by individuals in REPOSE over the 2 year follow up period

Resource use was collected either on an ongoing basis or was self-reported by the individuals in the trial at baseline or at a follow up period (6 months, 1 year and 2 years post-randomisation). All unit costs used to value the reported resource use in the economic evaluation alongside the trial (EEACT), apart from the costs associated with insulin use, are presented in Table **1**. The unit costs associated with insulin use are reported separately in Table 2.

Data was collected on an ongoing basis throughout the trial for all inpatient hospitalisations which were not scheduled to treat a pre-existing condition, incidence of severe hypoglycaemia and whether or not an individual used an insulin pump. At each recorded hospital admission (either self-reported or collected on an ongoing basis) information was collected on the cause. The possible causes for an admission in the REPOSE dataset were: diabetic ketoacidosis (DKA); myocardial infarction; severe hypoglycaemia; ischaemic heart disease; unstable angina; heart failure; foot ulcer; and renal disease. Severe hypoglycaemia was defined in the REPOSE trial as been any hypoglycaemic episode which an individual was unable to treat themselves. If it was reported that an individual did not have an inpatient admission or a paramedic callout for their severe hypoglycaemic event, then it was assumed that a friend or family member provided aid to the individual. These adverse events had no implications for NHS resource use, so these episodes of severe hypoglycaemia were assumed to have no monetary cost to the NHS.

For peer review only

Individual's self-reported resource use since the last REPOSE visit was collected on: the number of face to face and telephone diabetes related contacts with health care professionals; the use of lipid lowering, antiplatelet or depression medication; data at baseline on number of diabetes related admissions in the past year, days spent in hospital and the reason for admission; and the type of insulin used, the average daily dose in the week preceding data collection, the number of injections per day, and the method of insulin delivery. The unit costs associated with insulin use are presented separately from the rest of the unit costs in Table 2. The daily cost of insulin was multiplied by the number of days between each data collection period (6 months, 1 year, and 2 years) to calculate the cost of insulin in the first and second year. If an individual was a receiving insulin pump therapy, the cost of needles, insulin pens and syringes were not applied, as these were already included in the estimates of the cost of insulin pump consumables. From this information a cost of insulin for each individual in the REPOSE trial was calculated.

Table 1: The unit costs used in the within trial analysis of the REPOSE data

Costs used in the within trial analyses	Cost (2013/14)	Notes
**DAFNE courses**		
Cost of a DAFNE course	£363.10	DAFNE fact sheet 6[1]
Cost of a pre-course pump fitting session	£28.82	REPOSE trial data and DAFNE fact sheet 6[1]
**Hypoglycaemia**		
Cost of hypoglycaemia admission	£446.73	NHS reference costs 2013-14.[2] Non-elective inpatient short stay. FCE weighted average of the currency codes: KB01C, KB01D, KB01E, KB01F, KB02G, KB02H, KB02J, KB02K
Paramedic cost per case	£233.58	Elliot *et al* 2014.[3] Table 3.
**Cost of inpatient admissions**		
Diabetic ketoacidosis		
Cost of the first day	£527.78	NHS reference costs 2012-13. Non-elective inpatient short stay. Currency code PA67Z.
Cost of subsequent days	£284.42	NHS reference costs 2012-13. Non-elective inpatients excess bed days. Currency code PA67Z.
Renal hospitalisation		
Cost of the first day	£471.70	NHS reference costs 2013-14.[2]Non-elective inpatients short stay. Weighted average of the currency codes: LA09J, LA09K, LA09L, LA09M, LA09N, LA09P, LA09Q

For peer review only

Cost of subsequent days	£257.87	NHS reference costs 2013-14.[2]Non-elective inpatients excess bed days. Currency codes: LA09J, LA09K, LA09L, LA09M, LA09N, LA09P, LA09Q
Myocardial Infarction		
Cost of the first day	£560.60	NHS reference costs 2013-14.[2]Non-elective inpatients short stay. Weighted average of the currency codes: EB10A, EB10B, EB10C, EB10D, and EB10E.
Cost of subsequent days	£248.89	NHS reference costs 2013-14.[2]Non-elective inpatients excess bed days. Currency codes: EB10A, EB10B, EB10C, EB10D, EB10E.
Foot Ulcer		
Cost of the first day	£509.39	NHS reference costs 2012-13. Non-elective inpatient short stay. Currency codes: KB03C, KB03D, KB03E.
Cost of subsequent days	£156.34	NHS reference costs 2012-13. Non-elective inpatient short stay day. Currency codes: KB03C, KB03D, KB03E.
Other Inpatient stays		
Cost of the first day	£755.44	NHS reference costs 2012-13. Non-elective inpatient short stay. Currency code PA68Z.
Cost of subsequent days	£335.81	NHS reference costs 2012-13. Non elective inpatient excess bed day. Currency code PA68Z.
**Medication costs (per quarter)**		
Cost of lipid medication	£9.27	Prescription Cost Analysis: England 2011 (BNF chapter 2 section 12)
Cost of antiplatelet medication	£1.87	Prescription Cost Analysis: England 2011 (BNF chapter 2 section 9)
Cost of depression medication	£6.08	Prescription Cost Analysis: England 2011 (BNF chapter 4 section 3)
**Cost of diabetes related contacts**		
Cost of a face to face clinic	£105.49	NHS reference costs 2013-14. Non consultant led outpatient attendance. Non admitted face to face follow up. Service description: Diabetic Medicine
Cost of a telephone contact	£75.80	NHS reference costs 2013-14. Non consultant led outpatient attendance. Non admitted non face to face follow up. Service description: Diabetic Medicine

DAFNE – dose adjustment for normal eating MDI, multiple daily injections:; HSCIC, Health & Social Care Information Centre

For peer review only

Table 2: The unit costs of insulin

Item	Average unit cost	Number of units	Cost per unit	Associated yearly cost of an insulin pen	Source
Consumables related to multiple daily injections					
Cost of an insulin needle	£0.11	N/A	N/A	N/A	HSCIC[4]
Cost of an insulin syringe	£0.13	N/A	N/A	N/A	HSCIC[4]
Quick Acting Insulin					
**Human Insulin**					
Vial	£9.87	1000	£0.01	N/A	BNF[5], HSCIC[4]
Cartridges for a reusable pen	£18.97	1500	£0.01	£8.78	
**Animal Insulin**					
Vial	£26.15	1000	£0.03	N/A	BNF[5], HSCIC[4]
Cartridges for a reusable pen	£38.29	1500	£0.03	£5.97	
**Insulin Aspart (NovoRapid)**					
Vial	£14.08	1000	£0.01	N/A	BNF[5], HSCIC[4]
Cartridges for a reusable pen	£28.31	1500	£0.02	£9.59	
Disposable Pen	£30.63	1500	£0.02	N/A	
**Insulin Lispro (Humalog)**					
Vial	£16.61	1000	£0.02	N/A	
Cartridges for a reusable pen	£28.31	1500	£0.02	£8.86	BNF[5], HSCIC[4]
Disposable Pen	£28.31	1500	£0.02	N/A	
**Insulin Glulisine (Apidra)**					
Vial	£16.00	1000	£0.02	N/A	
Cartridges for a reusable pen	£28.30	1500	£0.02	£7.86	BNF[5], HSCIC[4]
Disposable Pen	£28.30	1500	£0.02	N/A	
Background Insulin					
**Human Insulin**					
Vial	£10.41	988	£0.01	N/A	
Cartridges for a reusable pen	£21.52	1500	£0.01	£9.30	BNF[5], HSCIC[4]
Disposable Pen	£21.05	1500	£0.01	N/A	
**Animal Insulin**					
Vial	£26.17	1000	£0.03	N/A	BNF[5], HSCIC[4]
Cartridges for a reusable pen	£38.32	1500	£0.03	£9.57	
**Insulin Detemir (Levemir)**					
Cartridges for a reusable pen	£42.00	1500	£0.03	£9.59	BNF[5], HSCIC[4]
Disposable Pen	£42.10	1500	£0.03	N/A	
**Insulin Glargine (Lantus)**					

For peer review only

Vial	£30.68	1000	£0.03	N/A	
Cartridges for a reusable pen	£41.50	1500	£0.03	£7.86	BNF[5], HSCIC[4]
Disposable Pen	£41.50	1500	£0.03	N/A	
Mixed Insulin					
**Biphasic Isophane Insulin**					
**Animal Insulin**					
Vial	£25.20	1000	£0.03	N/A	BNF[5], HSCIC[4]
Cartridges for a reusable pen	£37.80	1500	£0.03	£5.97	
**Human Insulin**					
Vial	£15.43	987	£0.02	N/A	BNF[5], HSCIC[4]
Cartridges for a reusable pen	£18.94	1500	£0.01	£7.74	
Disposable Pen	£21.43	1500	£0.01	N/A	
**Biphasic Insulin Aspart**					
Cartridges for a reusable pen	£28.79	£28.79	£0.02	£9.59	BNF[5], HSCIC[4]
Disposable Pen	£29.89	£29.89	£0.02		
**Biphasic Insulin Lispro**					
Vial	£16.61	1000	£0.02		
Cartridge for reusable pen	£29.03	1500	£0.02	£8.93	BNF[5], HSCIC[4]
Disposable Pen	£30.13	1500	£0.02		

BNF, British National Formulary; HSCIC, Health & Social Care Information Centre

For peer review only

1.1.1 Treatment switching

During the REPOSE trial, it was possible for individuals to switch from insulin delivery mechanism to the other i.e. to switch from insulin pump therapy to multiple daily injections and vice versa. It was possible to switch treatment twice and two individuals did so in the REPOSE trial. The data in the REPOSE trial was analysed to assess the number of people with diabetes who switched treatment. The estimated cost of insulin and insulin pumps were adjusted to reflect the fact that individuals switch treatments.

1.1.2 Estimating the within trial cost effects

In the base case analysis, complete cost information was used in the EEACT. Complete total cost information was available for 98%, 90% and 92% of individuals in the intention to treat population at baseline, 1 year and 2 years respectively.

In a scenario analysis missing cost data was imputed for those individuals who attended at least one REPOSE follow up visit. Total discounted cost data was imputed using chained equations (predictive mean matching) using baseline HbA1c, treatment allocation, age at baseline, and baseline cost values as covariates in the imputation equations. 10 different imputed values were calculated for each individual with missing data.

1.1.3 Estimating within trial QALY effects using EQ-5D and SF-12

To generate quality adjusted life year (QALY) measures over the two year trial follow-up, information was collected on individual's utility using two different instruments, the EQ-5D and the SF-12. The EQ-5D and SF-12 questionnaires were completed by individuals at baseline and all follow up visits (6 months, 12 months and 24 months).

In the base case within trial analysis, the utility values measured by the EQ-5D-3L were used to calculate QALYs using an area under the curve analysis. EQ-5D utility scores were used in the base case because they are NICE's preferred utility measure.[6] In a scenario analysis, utility values measured using the SF-6D (a measure derived from the SF-12) were used to calculate QALY values.[7] In the base case, only individuals with complete QALY data were included in the analysis. Utilities as measured by EQ-5D-3L were completed by 99%, 93%, 88% and 90% of individuals at baseline, 6, 12 and 24 months respectively. If an individual had a missing 6 month utility value it was assumed that the 6 month utility value

For peer review only

would be the average of the baseline and one year utility values. If an individual had a missing utility score at12 or 24 months, then they were excluded from the base case analysis.

In a scenario analysis, multiple imputation was used to impute missing QALYs values for individuals with an assessment data for least one follow up point. Data was imputed using chained equations (predictive mean matching) using baseline HbA1c, treatment allocation, age at baseline, and baseline cost or QALY values as covariates in the imputation equations. 10 different imputed values were calculated for each individual with missing data in the analyses using imputed data

1.1.4 The statistical model used for the within trial analysis

A seemingly unrelated regression model was used to estimate the costs and QALYs in the EEACT.[8] One seemingly unrelated regression was fitted to four outcome variables: total discounted costs in year 1; total discounted costs in year 2; total discounted QALYs in year 1 and total discounted QALYs in year 2; using the mysureg command in the ml_ado package in STATA version 13.1. For the QALY outcome variables, baseline HbA1c, treatment allocation and baseline utility were included as covariates. Baseline utility was included as a covariate to estimate QALYs so that any baseline differences in health between the two treatment arms was controlled for.[9] For the cost outcome variables, baseline HbA1c, site, treatment allocation and baseline resource use were included as covariates. The standard errors of the seemingly unrelated regression was adjusted for clustering within each DAFNE course.

1.1.5 Analysis

The key measure of cost-effectiveness in the EEACT was the incremental cost-effectiveness ratio base on the mean incremental effect of insulin pumps and dose adjustment for normal eating (pumps+DAFNE) compared to multiple daily injections and dose adjustment for normal eating (MDI+DAFNE) on total costs and total QALYs. The confidence intervals around these effects were estimated from the variance –covariance matrix of the regression model. The results were presented on a cost-effectiveness plane and the uncertainty around the mean effect was presented using a confidence ellipse.

2 Results

2.1 Base Case analysis

For peer review only

The results of the within trial cost-effectiveness analysis are presented using a confidence ellipse in

For peer review only

Figure **1**. In the base case pumps + DAFNE was dominated by MDI + DAFNE as pumps + DAFNE produced fewer mean QALYs at a higher mean cost. The confidence ellipses show pumps + DAFNE was associated with statistically significantly higher costs than MDI + DAFNE at the 5% significance level. The confidence ellipses also show that pumps + DAFNE was not associated with statistically significantly lower QALYs than MDI+DAFNE at the 5% significance level. This is as the confidence ellipse crosses the y axis of the graph at 0. Another point to note is that the confidence ellipses do not cross a threshold ICER of £20,000 per QALY gained; therefore the ICER of pumps + DAFNE compared to MDI + DAFNE is greater than £20,000 per QALY gained at the 95% confidence level.

The cost-effectiveness acceptability curve is presented in Figure 2. It shows that pumps + DAFNE has a 0.0% chance of being cost effective at threshold ICERs of £20,000 per QALY gained and £30,000 per QALY gained.

Table 3 presents the incremental cost and QALY outcomes of pumps + DAFNE compared to MDI + DAFNE in each year of the trial and for both years combined. In the base case the incremental cost in year 2 is lower than the cost in year 1. This is likely due to 1) treatment switching and 2) the rate of DKAs and severe hypoglycaemic events were noticeably lower in the pumps + DAFNE arm in the second year compared to the first year. The incremental QALYs are negative in the first year and positive in the second year. However in neither year is this result statistically significant and in both years, the mean effects are less than 1/100th of a QALY.

For peer review only

Figure 1: The cost-effectiveness ellipse for the base case within trial analysis

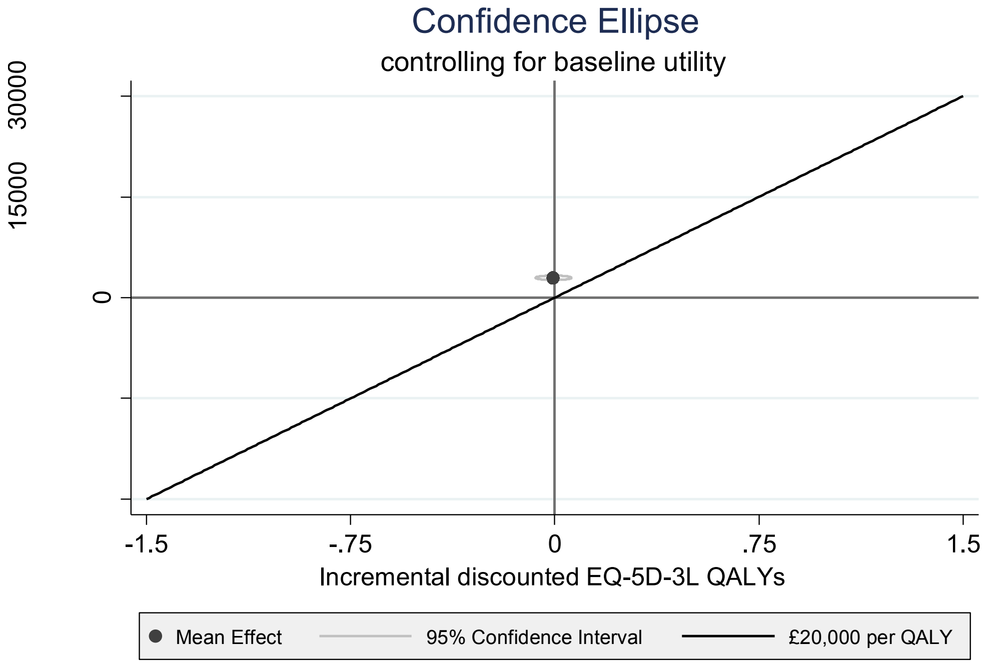

Figure 2: The cost-effectiveness acceptability curve for the base case within trial analysis

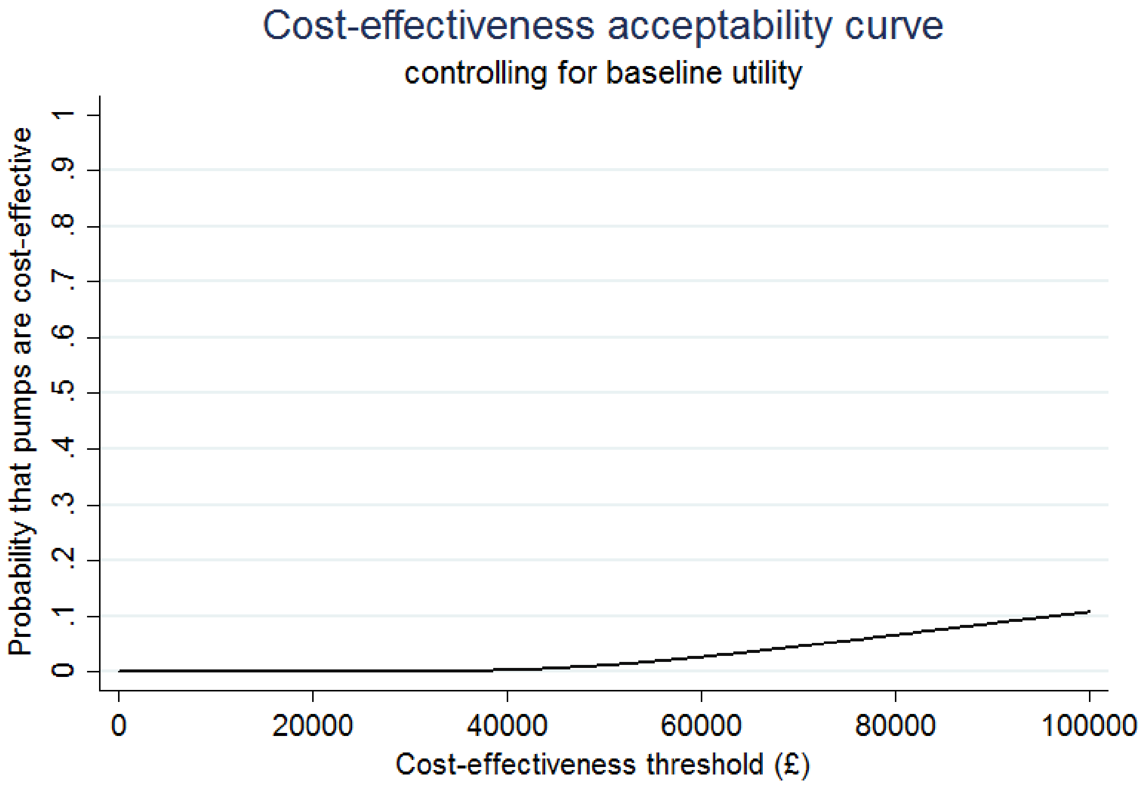

2.1.1 Summary of the scenario analyses

The following scenario analyses were undertaken:

1) Those individuals who adhered to their randomised method of insulin delivery
2) Missing Cost and QALY data were imputed
3) QALYs measured by SF-6D were used instead of QALYs measured using EQ-5D
4) Imputed data and QALYs measured by SF-6D QALYs
5) Pump costs measured by Riemsma et al.[10] were used
6) Riemsma et al. pump costs were used and missing data was imputed
7) The cost of pumps and consumables are 25% lower
8) The cost of pumps and consumables are 25% lower in a per protocol population
9) The cost of pumps and consumables are 50% lower
10) The cost of pumps and consumables are 50% lower in a per protocol population

For peer review only

2.1.2 Results of the scenario analyses

The results of the scenario analyses are also presented in Table 3. It is clear that pumps + DAFNE is dominated by MDI + DAFNE in all analyses, except those conducted in the per protocol population. The lowest ICER is observed in the scenario were the per protocol population is used and there is a cost reduction in insulin pumps and consumables of 50%. The ICER in this scenario is £552,866, which is above the £20,000 to £30,000 per QALY gained threshold considered by NICE.

For peer review only

Table 3: Within trial cost-effectiveness analysis results of continuous subcutaneous insulin infusion (pumps) versus multiple daily injections (MDI), both with dose adjustment for normal eating structured education

		N	Incremental costs in year 1	Incremental costs in year 2	Total incremental costs	Incremental QALYs in year 1	Incremental QALYs in year 2	Total incremental QALYs	ICER (£/QALY gained)
**Base Case** – ITT population with complete costs and QALYs	Mean<br>95% CI	205	£1732<br>(£1511, £1952)	£1228<br>(£1063, £1392)	£2959<br>(£2692, £3227)	-0.007<br>(-0.036,0.022)	0.003<br>(-0.029, 0.035)	-0.004<br>(-0.057, 0.048)	Dominated
**Scenario Analyses**									
Per protocol population	Mean<br>95% CI	188	£1780<br>(£1520, £2041)	£1434<br>(£1328, £1539)	£3214<br>(£2916, £3513)	-0.003<br>(-0.034, 0.027)	0.006<br>(-0.026, 0.037)	0.002<br>(-0.051, 0.056)	£1,369,287
Imputed data	Mean<br>95% CI	260	£1697<br>(£1492, £1901)	£1175<br>(£1006, £1345)	£2872<br>(£2602, £3142)	-0.013<br>(-0.039, 0.014)	0.004<br>(-0.029, 0.037)	-0.009<br>(-0.058, 0.04)	Dominated
SF-6D QALYs	Mean<br>95% CI	196	£1746<br>(£1514, £1978)	£1254<br>(£1096, £1412)	£3000<br>(£2729, £3271)	-0.001<br>(-0.021, 0.019)	-0.002<br>(-0.027, 0.023)	-0.003<br>(-0.045, 0.039)	Dominated
Imputed data and SF-6D QALYs	Mean<br>95% CI	256	£1701<br>(£1494, £1908)	£1186<br>(£1016, £1357)	£2888<br>(£2616, £3159)	-0.003<br>(-0.021, 0.015)	-0.001<br>(-0.024, 0.022)	-0.004<br>(-0.041, 0.034)	Dominated
Riemsma et al. pump costs	Mean<br>95% CI	205	£1679<br>(£1450, £1908)	£1184<br>(£1024, £1343)	£2863<br>(£2586, £3140)	-0.007<br>(-0.036, 0.022)	0.003<br>(-0.029, 0.035)	-0.004<br>(-0.057, 0.048)	Dominated
Imputed data Riemsma et al. pump costs	Mean<br>95% CI	260	£1648<br>(£1434, £1861)	£1125<br>(£964, £1286)	£2772<br>(£2498, £3047)	-0.013<br>(-0.039, 0.014)	0.004<br>(-0.029, 0.037)	-0.009<br>(-0.058, 0.04)	Dominated

For peer review only

Table 3: Within trial cost-effectiveness analysis results of insulin pumps versus multiple daily injections (MDI), both with dose adjustment for normal eating structured education (continued)

		N	Incremental costs in year 1	Incremental costs in year 2	Total incremental costs	Incremental QALYs in year 1	Incremental QALYs in year 2	Total incremental QALYs	ICER (£/QALY gained)
**Base Case** – ITT population with complete costs and QALYs	Mean<br>95% CI	205	£1732<br>(£1511, £1952)	£1228<br>(£1063, £1392)	£2959<br>(£2692, £3227)	-0.007<br>(-0.036,0.022)	0.003<br>(-0.029, 0.035)	-0.004<br>(-0.057, 0.048)	Dominated
**Scenario Analyses**									
The cost of pumps and consumables are 25% lower	Mean<br>95% CI	205	£1285<br>(£1022, £1547)	£955<br>(£850, £1059)	£2239<br>(£1786, £2314)	-0.007<br>(-0.036, 0.022)	0.003<br>(-0.104, 0.11)	-0.004<br>(-0.057, 0.048)	Dominated
The cost of pumps and consumables are 25% lower in a per protocol analysis	Mean<br>95% CI	188	£1223<br>(£1010, £1436)	£768<br>(£634, £902)	£1991<br>(£1939, £2540)	-0.003<br>(-0.034, 0.027)	0.006<br>(-0.026, 0.037)	0.002<br>(-0.051, 0.056)	£966,218
The cost of pumps and consumables are 50% lower	Mean<br>95% CI	205	£767<br>(£532, £1001)	£375<br>(£255, £494)	£1141<br>(£873, £1409)	-0.007<br>(-0.036, 0.022)	0.003<br>(-0.029, 0.035)	-0.004<br>(-0.057, 0.048)	Dominated
The cost of pumps and consumables are 50% lower in a per protocol analysis	Mean<br>95% CI	188	£789<br>(£524, £1053)	£475 (£372, £579)	£1264<br>(£961, £1567)	-0.004<br>(-0.034, 0.027)	0.006<br>(-0.026, 0.037)	0.002<br>(-0.051, 0.056)	£552,866

All costs are reported in 2013/14 prices ;ITT, intention to treat; QALY, quality adjusted life year; CI, confidence interval; ICER, incremental cost-effectiveness ratio

For peer review only

References

1. DAFNE. DAFNE Fact Sheet Six. Online Source. 2012;Available from; http://www.dafne.uk.com/uploads/135/documents/06_factsheetsix_12pt_18_06_12.pdf. Last Accessed 1st July 2015.
2. Department of Health. NHS reference costs 2013 to 2014. Online Source. 2014;Available from; https://www.gov.uk/government/publications/nhs-reference-costs-2013-to-2014. Last accessed 17th July 2015.
3. Elliott J, Jacques RM, Kruger J, Campbell MJ, Amiel SA, Mansell P et al. Substantial reductions in the number of diabetic ketoacidosis and severe hypoglycaemia episodes requiring emergency treatment lead to reduced costs after structured education in adults with Type 1 diabetes. Diabet Med. 2014;31(7):847-53. doi:10.1111/dme.12441.
4. Health & Social Care Information Centre. Prescription Cost Analysis England 2014. Online Source. 2015;Available from; http://www.hscic.gov.uk/catalogue/PUB17274. Last Accessed 2nd Novemeber 2015.
5. British Medical Association and Royal Pharmaceutical Society of Great Britain. British National Formulary August 2015. Online Source. 2015;Available from: https://www.medicinescomplete.com, Last Accessed 9th September 2015.
6. National Institute for Health and Care Excellence. Guide to the methods of technology appraisal 2013. Online Source. 2013;Available from; http://www.nice.org.uk/article/pmg9/resources/non-guidance-guide-to-the-methods-of-technology-appraisal-2013-pdf. Last accessed 13th July 2015.
7. Brazier JE, Roberts J. The estimation of a preference-based measure of health from the SF-12. Med Care. 2004;42(9):851-9.
8. Zellner A. An Efficient Method of Estimating Seemingly Unrelated Regressions and Tests for Aggregation Bias. J Am Stat Assoc. 1962;57(298):348-&.
9. Manca A, Hawkins N, Sculpher MJ. Estimating mean QALYs in trial-based cost-effectiveness analysis: the importance of controlling for baseline utility. Health Econ. 2005;14(5):487-96. doi:10.1002/hec.944.
10. Riemsma R, Corro Ramos I, Birnie R, Büyükkaramikli N, Armstrong N, Ryder S et al. Type 1 diabetes: Integrated sensor-augmented pump therapy systems for managing blood glucose levels (The MiniMed Paradigm Veo System and the Vibe and G4 PLATINUM CGM system), a systematic review and economic evaluation. Kleijnen Systematic Reviews Ltd. 2015.

For peer review only

Supplementary material B – Supporting information for the long term modelling

Table of Contents

1 Determining the number of PSA runs 1
2 Determining the number of simulated individuals to use in each run 1
3 Other cause mortality 1
4 Probability of death from end stage renal disease 1
5 The clinical effectiveness parameters 2
5.1 Treatment switching 2
5.2 HbA1c 14
5.3 DKA and severe hypoglycaemia 21
6 The cost of insulin, diabetes related contacts and insulin pumps used in the Sheffield Type 1 Diabetes model 25
7 Detailed results of the scenario and subgroup analyses 32

List of Tables

Table 1: The results of the parametric survival models fitted to patients allocated to the continuous subcutaneous insulin infusion dose adjustment for normal eating arm of the REPOSE trial (n=130) ... 4
Table 2: The results of the parametric survival models fitted to patients allocated to the multiple daily injections with dose adjustment for normal eating arm of the REPOSE trial (n=128) 6
Table 3: A summary of the Akaike information criterion and the Bayesian information criterion for the fitted survival curves used in the long term modelling 8
Table 4: The variance – covariance matrix for the exponential model fitted to the pumps arm 11
Table 5: The variance – covariance matrix for the exponential model fitted to the multiple daily injections arm 11
Table 6: The variance – covariance matrix for the weibull model fitted to the pumps arm 11
Table 7: The variance – covariance matrix for the weibull model fitted to the multiple daily injections arm 11
Table 8: The variance – covariance matrix for the gompertz model fitted to the pumps arm 11
Table 9: The variance-covariance matrix for the gompertz model fitted to the multiple daily injections arm 12
Table 10: The variance – covariance matrix for the log-logistic model fitted to the pumps arm 12
Table 11: The variance – covariance matrix for the log-logistic model fitted to the multiple daily injections arm 12
Table 12: The variance – covariance matrix for the log-normal model fitted to the pumps arm 12
Table 13: The variance – covariance matrix for the log-normal model fitted to the multiple daily injections arm 13
Table 14: The effect of continuous subcutaneous insulin infusion compared to multiple daily injections for all individuals in the intention to treat population. 15

For peer review only - http://bmjopen.bmj.com/site/about/guidelines.xhtml

Table 15: The effect of continuous subcutaneous insulin infusion compared to multiple daily injections for all individuals in the per protocol population. 16
Table 18: The variance covariance matrix for the beta regression to predict one year HbA1c in the ITT population 17
Table 19: The variance covariance matrix for the beta regression to predict two year HbA1c in the ITT population 18
Table 16: The variance covariance matrix for the beta regression to predict one year HbA1c in the per protocol population 19
Table 17: The variance covariance matrix for the beta regression to predict two year HbA1c in the per protocol population 20
Table 20: A summary of the observed incidence of diabetic ketoacidosis and severe hypoglycaemia in the intention to treat population 22
Table 21: The negative binomial model fitted to the incidence of severe hypoglycaemia at one year and two years 24
Table 22: The negative binomial model fitted to the incidence of diabetic ketoacidosis at one and two years 24
Table 23: The unit costs of insulin 25
Table 24: The variance covariance matrix for the seemingly unrelated regression on insulin costs in year 1 and 2 of REPOSE 29
Table 25: The variance covariance matrix for the seemingly unrelated regression on the cost of diabetes related contacts with health care professionals in year 1 and 2 of REPOSE 30
Table 26: The variance covariance matrix for the seemingly unrelated regression on the cost of insulin pumps and associated consumables in year 1 and 2 of REPOSE 31
Table 27: The One way scenario analyses and subgroup analyses performed using the Sheffield Type 1 Diabetes Policy Model 32

List of Figures

Figure 1: A visual plot of the Kaplan-Meier and survival curves estimated from parametric models for those individuals who were randomised continuous subcutaneous insulin infusion with dose adjustment for normal eating 9
Figure 2: A visual plot of the Kaplan-Meier and survival curves estimated from parametric models for those individuals who were randomised multiple daily injections with dose adjustment for normal eating 10

List of abbreviations in this supplementary material

**Abbreviation**	**Definition**
iNMB	Incremental net monetary benefit
QALY	Quality adjusted life year
PSA	Probabilistic sensitivity analysis
UK	United Kingdom
ESRD	End stage renal disease
DKA	Diabetic ketoacidosis
Pumps	Insulin pumps
MDI	Multiple Daily Injections
DAFNE	Dose adjustment for normal eating
T1DM	Type 1 diabetes mellitus

For peer review only - http://bmjopen.bmj.com/site/about/guidelines.xhtml

AIC	Akaike information criterion
BIC	Bayesian information criterion
ITT	Intention to treat
DCCT	Diabetes Control and Complications Trial

For peer review only

For peer review only - http://bmjopen.bmj.com/site/about/guidelines.xhtml

1 Determining the number of PSA runs

The stability of the model results was assessed in terms of the stability of incremental net monetary benefit (iNMB) using a threshold cost per quality adjusted life year (QALY) of £20,000 per QALY gained. This statistic is useful for the purpose of assessing model stability as, so long as the mean iNMB is statistically significantly different from 0 at the 5% level then the results are sufficiently stable that any decision made as a consequence of them will not change.

$$Z_i = the\ iNMB\ for\ each\ PSA\ run$$

$$N = the\ number\ of\ PSA\ simulations$$

$$The\ mean\ iNMB\ is\ given\ by:\ \bar{Z} = \frac{1}{N}\sum_{i=1}^{N} Z_i$$

$$The\ variance\ in\ mean\ iNMB\ is\ given\ by: Var(\bar{Z}) = \frac{1}{N-1}\sum_{i=1}^{n}(Z_i - \bar{Z})$$

$$The\ standard\ error\ of\ the\ mean\ iNMB\ is\ given\ by: Standard\ error(\bar{Z}) = \sqrt{Var(\bar{Z})/N}$$

The mean incremental net monetary benefit at £20,000 per QALY gained was -£16,201 ($\bar{Z}$). The variance of this was 2.18E+08. The standard error was $\sqrt{2.18E + 8/500} = 661$. As the standard error was under 5% of the mean incremental net monetary benefit, 500 probabilistic sensitivity analysis (PSA) runs was determined to be sufficiently robust for making reliable decisions for these comparators

2 Determining the number of simulated individuals to use in each run

Likewise to determine the stability of the ICER in the deterministic results and the scenario analyses the stability of the iNMB was assessed over 5,00 simulated individuals. The model was run initially using the same 5,000 simulated individuals in each arm of the deterministic base case. The mean incremental net monetary benefit at £20,000 per QALY gained was -£15,759 ($\bar{z}$). The variance of this was 1.27E+10. The standard error was $\sqrt{1.27E + 10/5000} = 1{,}594$. As the standard error was approximately 10% of the mean incremental net monetary benefit, 5000 simulated individuals was determined to be sufficient to produce stable results for the analyses.

3 Other cause mortality

Individuals can also die from other causes. This other-cause mortality was updated in version of the model used for these analyses. Other-cause mortality was calculated using UK life tables from 2012 to 2014 adjusted to exclude the causes either attributed to diabetes mellitus (either type 1, type 2 or unspecified, ICD-10 codes E10-14) or modelled directly in the microvascular and macrovascular disease components (deaths due to: end stage renal disease; myocardial infarction, stroke and heart failure, ICD-10 codes N18, I20-21, I61-64, I50).[1-4]

4 Probability of death from end stage renal disease

This model parameter was altered from the value reported in Heller *et al.*[5] to reflect directly observable data available in Wolfe *et al.*[6] on the probability of end stage renal disease. At baseline, 102,163 patients with diabetes were receiving dialysis and over a maximum follow up of 6 years 44,916 of these patients died. This gave a probability of death from end stage renal disease (ESRD) of 44.00% over 6 years. In the model probabilistic sensitivity analysis, the uncertainty in this probability was parameterised using a beta distribution with an alpha parameter of 44,916 and a beta parameter of 57,247. The probability of death from end stage renal disease over 6 years was first converted into an instantaneous rate of death and then yearly probability of death from end stage renal disease using the method in Briggs et al.[7]

5 The clinical effectiveness parameters

5.1 Treatment switching

The treatment switching curves were used to estimate the incidence of treatment switching in the model in the first and second year. Covariates were used in the parametric models to control for: HbA1c prior to switching, number of diabetic ketoacidosis events (DKAs) and number of severe hypoglycaemic events in the year prior to switching (or at 2 years follow up if no switching occurred). The standard errors of the parametric survival models were adjusted for clustering in each DAFNE course. As separate models were fitted to the insulin pumps + dose adjustment for normal eating (pumps+DAFNE) and the multiple daily injections + dose adjustment for normal eating (MDI+DAFNE) arm, no assumption of proportional hazards or accelerated failure time was made.

The curves were not extrapolated, as the clinical expert opinion of a Professor of Clinical Diabetes & Honorary Consultant Physician and a Professor in Public Health & Health Technology Assessment was that if an individual was still using a pump after two years that they would continue to use pump as in their experience once an adult with type 1 diabetes mellitus (T1DM) was successfully using an insulin pump they were unlikely change their insulin delivery method.

The different parameters of the parametric survival models fitted to the pumps+DAFNE arm is given in Table 1. The equivalent parameters are given for the MDI+DAFNE arm in Table 2.

The goodness of fit of the parametric survival curves were assessed using the Akaike information criterion (AIC), Bayesian information criterion (BIC) and a visual assessment of the survival curves estimated from parametric models plotted against the nonparametric Kaplan-Meier curve . The one and two-year time points are the time points of relevance for assessing the visual fit of the curves in this analysis, as the model uses a yearly time cycle so treatment switching is only predicted in the model at these time points. The AIC and the BIC are given in Table 3. Lower values for these statistics indicate a better model fit. For the pumps+DAFNE arm, the exponential model has the lowest AIC and BIC. For the MDI+DAFNE arm, the Weibull model had the lowest AIC and the exponential model has the lowest BIC. The plot of the parametric survival curves against the underlying Kaplan-Meier curve are provided in Figures 1 and 2. It is clear that the exponential model provides a reasonable fit to the Kaplan-Meier for the pumps+DAFNE arm at the one and two-year time points therefore this curve was used in the base case economic model. It is also clear that for the MDI+DAFNE arm, the exponential curve does not provide a reasonable fit to the Kaplan –Meier curve at one year whereas the Weibull curve provides a reasonable fit at both one and two years. Therefore the Weibull curve was used as the base case curve for the MDI+DAFNE model arm.

The uncertainty in the parametric survival curves was included in the model's probabilistic sensitivity analysis using a multivariate normal distribution. The variance-covariance matrices and the predicted

For peer review only - http://bmjopen.bmj.com/site/about/guidelines.xhtml

coefficients for each of the parametric survival models were used to parameterise the multivariate normal distributions. The coefficients are given in Table 1and Table 2 respectively and the variance – covariance matrices for each of the parametric curves is given in Tables 4 toTable **13**.

For peer review only

For peer review only - http://bmjopen.bmj.com/site/about/guidelines.xhtml

Table 1: The results of the parametric survival models fitted to patients allocated to the pumps with dose adjustment for normal eating arm of the REPOSE trial (n=130)

Parameter	Coefficient	Robust standard error	95% confidence interval	
Exponential model<br>S(t) = Exp(-Exp(FV)*time),<br>FV = 0.222*HbA1c+-0.972*number of DKAs + 0.427*number of severe hypos -4.616				
HbA1c	0.222	0.241	-0.251	0.695
Number of DKAs	-0.972	0.474	-1.901	-0.042
Number of severe hypoglycaemic events	0.427	0.087	0.257	0.598
Constant	-4.616	2.125	-8.781	-0.451
Weibull model<br>S(t) = Exp(-Exp(FV)*time^Exp(Ln Scale))<br>FV = 0.221*HbA1c+-0.981*number of DKAs + 4.460*number of severe hypos -4.460				
HbA1c	0.221	0.234	0.016	0.694
Number of DKAs	-0.981	0.471	-7.113	-4.910
Number of severe hypoglycaemic events	0.404	0.085	0.337	0.684
Constant	-4.460	2.100	-10.607	-4.696
Ln Scale	-0.258	0.220	0.111	1.377
Gompertz model<br>S(t) = (-Exp(FV)* (1/Gamma))*(Exp(Gamma*time)-1)<br>FV = = 0.220*HbA1c+-0.983*number of DKAs + 0.407*number of severe hypos -4.307				
HbA1c	0.220	0.236	-0.243	0.684
Number of DKAs	-0.983	0.468	-1.901	-0.065
Number of severe hypoglycaemic events	0.407	0.090	0.230	0.584
Constant	-4.307	2.232	-8.682	0.068
Gamma	-0.316	0.479	-1.256	0.624
Log logistic model<br>S(t) = 1/ (1+(1/Exp(FV)*time)^(1/Exp(Gamma))<br>FV = -0.294*HbA1c+1.406*number of DKAs + -0.554*number of severe hypos +5.637				
HbA1c	-0.294	0.286	-0.855	0.267
Number of DKAs	1.406	0.676	0.081	2.730
Number of severe hypoglycaemic events	-0.554	0.170	-0.887	-0.220
Constant	5.637	2.510	0.718	10.557
Gamma	0.215	0.230	-0.235	0.665
Log normal model<br>S(t) = 1 – Φ ((ln(time) – FV)/ Standard Deviation), were Φ is the standard normal distribution<br>FV = -0.307*HbA1c+ 1.867*number of DKAs + -0.656*number of severe hypos +6.406				
HbA1c	-0.307	0.292	-0.879	0.264
Number of DKAs	1.867	0.755	0.387	3.347
Number of severe hypoglycaemic events	-0.656	0.180	-1.009	-0.304
Constant	6.406	2.520	1.466	11.346
St Dev	1.002	0.206	0.598	1.405

For peer review only - http://bmjopen.bmj.com/site/about/guidelines.xhtml

DKA, diabetic ketoacidosis; ln, natural logarithm

For peer review only

For peer review only - http://bmjopen.bmj.com/site/about/guidelines.xhtml

Table 2: The results of the parametric survival models fitted to patients allocated to the multiple daily injections with dose adjustment for normal eating arm of the REPOSE trial (n=128)

Parameter	Coefficient	Robust standard error	95% confidence interval	
Exponential model<br>S(t) = Exp(-Exp(FV)*time),<br>FV = 0.336*HbA1c+-5.5555*number of DKAs + 0.460*number of severe hypos -6.725				
HbA1c	0.336	0.164	0.014	0.657
Number of DKAs	-5.555	0.561	-6.655	-4.455
Number of severe hypoglycaemic events	0.460	0.074	0.315	0.605
Constant	-6.725	1.450	-9.567	-3.884
Weibull model<br>S(t) = Exp(-Exp(FV)*time^Exp(Ln Scale))<br>FV = 0.335*HbA1c+-6.012*number of DKAs + 0.510*number of severe hypos -7.652				
HbA1c	0.355	0.173	0.016	0.694
Number of DKAs	-6.012	0.562	-7.113	-4.910
Number of severe hypoglycaemic events	0.510	0.089	0.337	0.684
Constant	-7.652	1.508	-10.607	-4.696
Ln Scale	0.744	0.323	0.111	1.377
Gompertz model<br>S(t) = (-Exp(FV)* (1/Gamma))*(Exp(Gamma*time)-1)<br>FV = = 0.350*HbA1c+-6.009*number of DKAs + 0.512*number of severe hypos -8.080				
HbA1c	0.350	0.170	0.016	0.683
Number of DKAs	-6.009	0.562	-7.110	-4.908
Number of severe hypoglycaemic events	0.512	0.094	0.329	0.696
Constant	-8.080	1.471	-10.963	-5.197
Gamma	1.055	0.669	-0.256	2.366
Log logistic model<br>S(t) = 1/ (1+(1/Exp(FV)*time)^(1/Exp(Gamma))<br>FV = -0.181*HbA1c+2.609*number of DKAs + -0.232*number of severe hypos +3.676				
HbA1c	-0.181	0.121	-0.418	0.055
Number of DKAs	2.609	0.799	1.044	4.175
Number of severe hypoglycaemic events	-0.232	0.070	-0.368	-0.095
Constant	3.676	1.317	1.094	6.258
Gamma	-0.780	0.317	-1.401	-0.160
Log normal model<br>S(t) = 1 – Φ ((ln(time) – FV)/ Standard Deviation), were Φ is the standard normal distribution<br>FV = -0.190*HbA1c+ 1.617*number of DKAs + -0.283*number of severe hypos +4.117				
HbA1c	-0.190	0.107	-0.400	0.021
Number of DKAs	1.617	0.517	0.603	2.630
Number of severe hypoglycaemic events	-0.283	0.101	-0.481	-0.086
Constant	4.117	1.291	1.587	6.647
Standard Deviation	0.066	0.338	-0.596	0.728

For peer review only

For peer review only - http://bmjopen.bmj.com/site/about/guidelines.xhtml

DKA, diabetic ketoacidosis; ln, natural logarithm

For peer review only

For peer review only - http://bmjopen.bmj.com/site/about/guidelines.xhtml

Table 3: A summary of the Akaike information criterion and the Bayesian information criterion for the fitted survival curves used in the long term modelling

	Parametric survival model	AIC (smaller is better)	BIC (smaller is better)	-2*log pseudo likelihood
pumps+DAFNE	Exponential	**145.77**	**157.24**	137.77
	Weibull	146.46	160.80	136.46
	Gompertz	147.25	161.59	137.25
	Log logistic	147.49	161.83	137.49
	Log normal	148.48	162.82	138.48
MDI + DAFNE	Exponential	64.36	**75.77**	56.36
	Weibull	**62.55**	76.81	52.55
	Gompertz	63.76	78.02	53.76
	Log logistic	63.78	78.04	53.78
	Log normal	64.97	79.23	54.97

AIC - Akaike information criterion; BIC - Bayesian information criterion; DAFNE – dose adjustment for normal eating; MDI – multiple daily injections;

For peer review only

For peer review only - http://bmjopen.bmj.com/site/about/guidelines.xhtml

Figure 1: A visual plot of the Kaplan-Meier and survival curves estimated from parametric models for those individuals who were randomised continuous subcutaneous insulin infusion with dose adjustment for normal eating

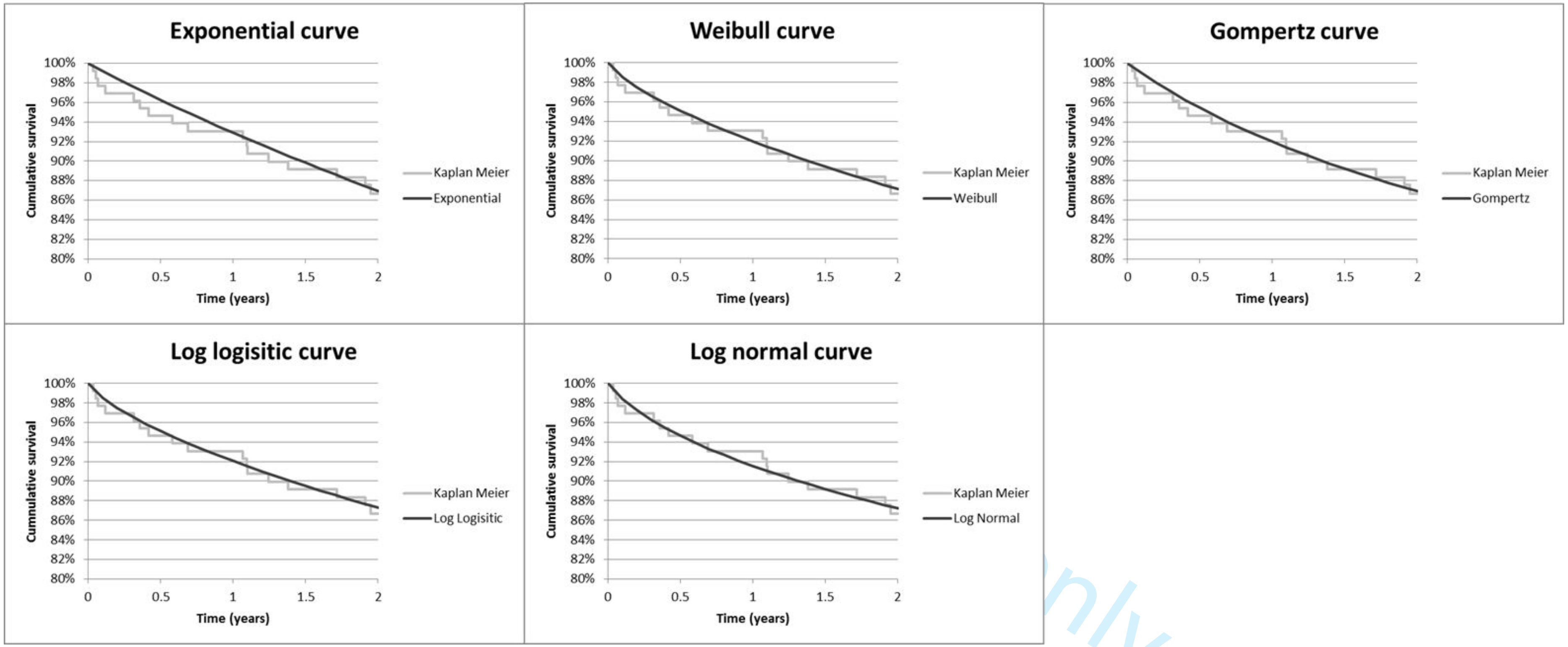

Figure 2: A visual plot of the Kaplan-Meier and survival curves estimated from parametric models for those individuals who were randomised multiple daily injections with dose adjustment for normal eating

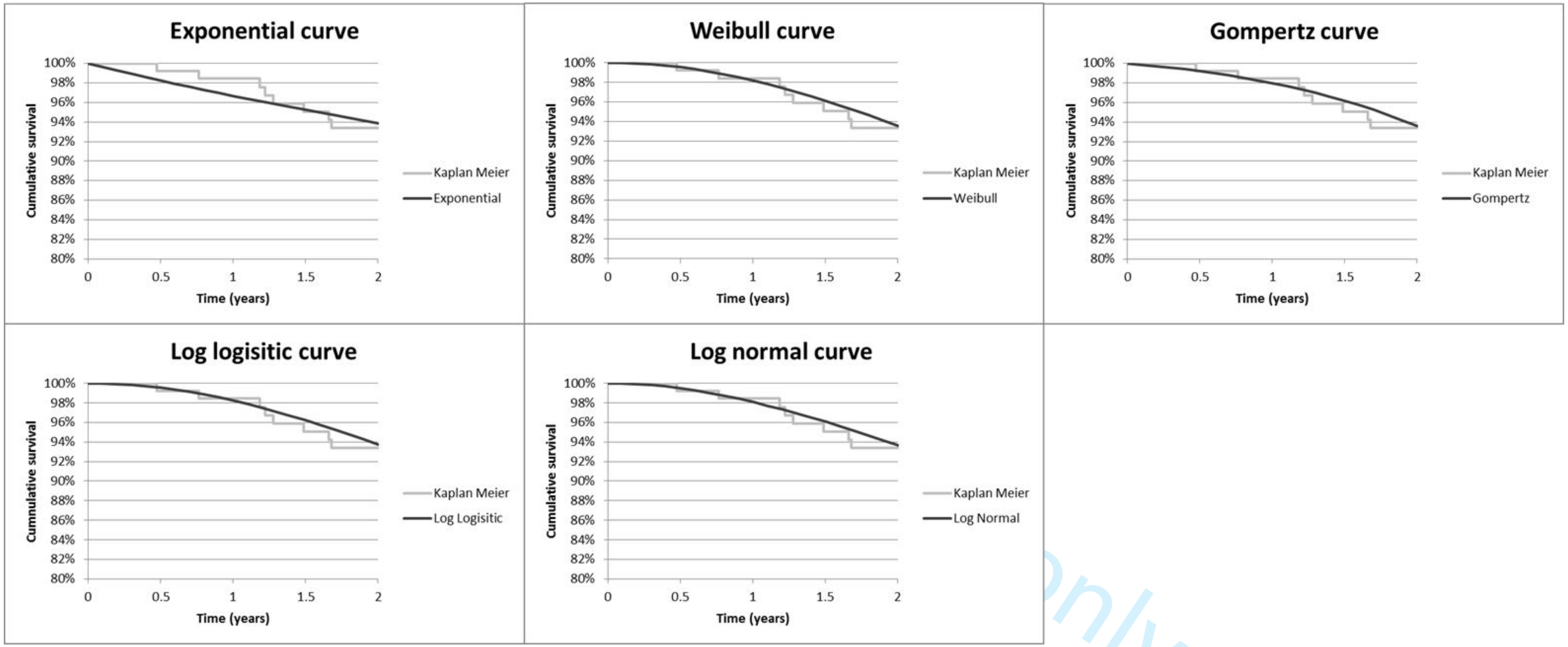

For peer review only - http://bmjopen.bmj.com/site/about/guidelines.xhtml

1 Table 4: The variance – covariance matrix for the exponential model fitted to the pumps arm

	HbA1c	DKAs	Sev Hypos	Constant
HbA1c	0.06			
DKAs	0.06	0.22		
Sev Hypos	0.00	0.00	0.01	
Constant	-0.51	-0.56	-0.02	4.52

2

3 Table 5: The variance – covariance matrix for the exponential model fitted to the multiple daily
4 injections arm

	HbA1c	DKAs	Sev Hypos	Constant
HbA1c	0.03			
DKAs	0.00	0.31		
Sev Hypos	0.00	0.01	0.01	
Constant	-0.22	-0.15	0.01	2.10

5

6 Table 6: The variance – covariance matrix for the weibull model fitted to the pumps arm

	HbA1c	DKAs	Sev Hypos	Constant	Ln scale parameter
HbA1c	0.05				
DKAs	0.06	0.22			
Sev Hypos	0.00	0.00	0.01		
Constant	-0.49	-0.54	-0.04	4.41	
Ln scale parameter	0.01	0.04	0.01	-0.15	0.05

7

8 Table 7: The variance – covariance matrix for the weibull model fitted to the multiple daily injections
9 arm

	HbA1c	DKAs	Sev Hypos	Constant	Ln scale parameter
HbA1c	0.03				
DKAs	0.00	0.32			
Sev Hypos	-0.01	0.02	0.01		
Constant	-0.23	-0.16	-0.01	2.27	
Ln scale parameter	-0.01	0.01	0.01	-0.07	0.10

10

11 Table 8: The variance – covariance matrix for the gompertz model fitted to the pumps arm

	HbA1c	DKAs	Sev Hypos	Constant	**Gamma**
HbA1c	0.06				
DKAs	0.06	0.22			

For peer review only

Sev Hypos	0.00	0.00	0.01		
Constant	-0.51	-0.55	-0.05	4.98	
Gamma	0.03	0.03	0.02	-0.46	**0.23**

Table 9: The variance-covariance matrix for the gompertz model fitted to the multiple daily injections arm

	HbA1c	DKAs	Sev Hypos	Constant	Gamma
HbA1c	0.03				
DKAs	0.00	0.32			
Sev Hypos	-0.01	0.02	0.01		
Constant	-0.18	-0.15	-0.03	2.16	
Gamma	-0.04	0.01	0.04	-0.28	0.45

Table 10: The variance – covariance matrix for the log-logistic model fitted to the pumps arm

	HbA1c	DKA	sev hypos	Constant	Gamma
HbA1c	0.08				
DKA	0.03	0.46			
sev hypos	0.01	-0.04	0.03		
Constant	-0.69	0.02	-0.15	6.30	
Gamma	0.01	0.11	-0.01	0.06	0.05

Table 11: The variance – covariance matrix for the log-logistic model fitted to the multiple daily injections arm

	HbA1c	DKA	sev hypos	Constant	Gamma
HbA1c	0.01				
DKA	-0.04	0.64			
sev hypos	0.00	-0.04	0.00		
Constant	-0.15	0.70	-0.05	1.74	
Gamma	-0.02	0.24	-0.02	0.31	0.10

Table 12: The variance – covariance matrix for the log-normal model fitted to the pumps arm

	HbA1c	Number of DKAs	Number of severe hypos	Constant	Standard deviation
HbA1c	0.09				
DKA	-0.01	0.57			
Sev hypos	0.01	-0.06	0.03		
Constant	-0.69	0.42	-0.18	6.35	
Standard deviation	0.01	0.11	-0.02	0.07	0.04

For peer review only - http://bmjopen.bmj.com/site/about/guidelines.xhtml

Table 13: The variance – covariance matrix for the log-normal model fitted to the multiple daily injections arm

	HbA1c	DKA	Sev hypos	Constant	Ln sigma parameter
HbA1c	0.01				
DKA	-0.02	0.27			
Sev hypos	0.00	-0.04	0.01		
Constant	-0.12	0.44	-0.08	1.67	
Ln sigma parameter	-0.01	0.17	-0.03	0.33	0.11

For peer review only

For peer review only - http://bmjopen.bmj.com/site/about/guidelines.xhtml

5.2 HbA1c

Two beta regressions were used to estimate each individual's HbA1c in the first and second year respectively. Bounds were placed on the beta regression so that HbA1c was between the clinically plausible bounds of 29mmol/mol [4.8%] and 201 mmol/mol [20.5%]. Beta regressions estimate two parameters of interest, the mean effect and the dispersion on the variance. Treatment allocation, baseline HbA1c and centre were included as covariates for the mean effect on HbA1c after one year. Treatment allocation, baseline HbA1c, one year HbA1c and centre were included as covariates for the mean effect on HbA1c after two years. HbA1c in the previous year was used as a covariate for the dispersion parameters. The standard errors of both statistical models were adjusted for clustering within each DAFNE course. Due to presence of treatment switching, an individual's HbA1c was assumed to change as thought they had been allocated to the other trial arm. Therefore, the beta regressions were estimated in the per protocol population (switchers excluded) rather than the intention to treat (ITT) population (switchers included in their randomised arm). A sensitivity analysis was conducted in which the beta regressions were estimated in the ITT population.

The results of the beta regression in the intention to treat population is given in Table 14 and the beta regression estimated in the per protocol population is given in Table 15. The results of these beta regressions are not easily interpretable as changes in HbA1c, as a logit link function is used to estimate the mean effect parameter and the natural logarithm of the dispersion parameter is estimated instead of the dispersion parameter itself.

Missing data was observed for HbA1c values in the per protocol population at 6 months (2.1% missing), 1 year (4.2% missing) and 2 years (4.2% missing). A multiple imputation procedure was employed in individuals with at least one HbA1c value (at 6 or 12 months) after randomisation but no HbA1c value at 24 months. In line with the statistical analysis plan, missing 24 month HbA1c data was imputed by multiple imputation using chained equations (regression) based on 10 imputed data sets with baseline, 6 and 12 months HbA1c measurements, DAFNE course, centre, age, sex, and HFS worry as covariates, if a participant had some HbA1c follow-up data. This imputation procedure was conducted in the ITT population, prior to running the beta regressions. After imputation 236 out of 236 participants in the per protocol population and 259 out of 260 participants in the ITT population had HbA1c follow up data.

The uncertainty in the parametric survival curves was included in the model's probabilistic sensitivity analysis using a multivariate normal distribution. The variance-covariance matrices and the predicted coefficients for each of the beta regressions were used to parameterise the multivariate normal distributions. The coefficients are given in Table 14 and Table 15 respectively and the variance – covariance matrices for each of the beta regressions is given in Table 16 to Table 19 .

Each individual's mean effect parameter and dispersion parameter were used to calculate the expected variance in that individual's actual HbA1c outcome. The individual's predicted mean effect and variance in their mean effect were used to parameterise a beta distribution. Each individual's HbA1c in the model was sampled from the beta distribution which was parameterised by their individualised mean effect and variance. This random draw was then transformed onto the Diabetes Control and Complications Trial (DCCT) (%) scale for use in the risk equations of the model.

For peer review only

Table 14: The effect of continuous subcutaneous insulin infusion compared to multiple daily injections for all individuals in the intention to treat population.

Hba1c at one year (beta scale)	Coefficient	Standard error	t	P>t	95% Confidence interval	
Mean effect (Mu) – using a logit link function						
Treatment allocation (1= pumps + DAFNE, 0=MDI + DAFNE)	-0.056	0.038	-1.49	0.137	-0.131	0.018
Baseline HbA1c (beta scale)	3.978	0.248	16.01	0	3.491	4.465
Constant	-2.223	0.088	-25.28	0	-2.395	-2.050
Centre effects (Cambridge is the reference site)						
Dumfries and Galloway	-0.025	0.074	-0.33	0.738	-0.171	0.121
Edinburgh	-0.019	0.065	-0.3	0.768	-0.147	0.108
Glasgow	-0.154	0.099	-1.55	0.12	-0.348	0.040
Harrogate	0.022	0.041	0.52	0.602	-0.060	0.103
College Hospital - London	0.013	0.065	0.21	0.837	-0.114	0.140
Nottingham	0.214	0.060	3.58	0	0.097	0.331
Sheffield	0.066	0.057	1.17	0.241	-0.045	0.178
Dispersion parameter (phi) - using a natural logarithm link function						
Baseline HbA1c (beta scale)	-2.996862	0.9980645	-3	0.003	-4.954	-1.040
Constant	4.912	0.332	14.79	0	4.261	5.563
Hba1c at two years (beta scale)						
Mean effect (Mu) – using a logit link function						
Treatment allocation(1= pumps + DAFNE, 0=MDI + DAFNE)	-0.018	0.035	-0.52	0.603	-0.086	0.050
One year HbA1c (beta scale)	0.797	0.318	2.51	0.012	0.175	1.419
Baseline HbA1c (beta scale)	3.599	0.342	10.51	0	2.927	4.271
Constant	-2.380	0.091	-26.14	0	-2.558	-2.201
Centre effects (Cambridge is the reference site)						
Dumfries and Galloway	0.047	0.093	0.5	0.617	-0.137	0.230
Edinburgh	0.067	0.085	0.8	0.426	-0.098	0.233
Glasgow	0.137	0.097	1.42	0.155	-0.052	0.327
Harrogate	0.123	0.087	1.41	0.158	-0.048	0.294
College Hospital - London	0.079	0.087	0.9	0.366	-0.092	0.249
Nottingham	0.120	0.110	1.09	0.279	-0.098	0.337
Sheffield	0.156	0.080	1.96	0.05	0.000	0.312
Dispersion parameter (phi)) – using a natural logarithm link function						
One year HbA1c (beta scale)	-4.667	1.129	-4.13	0	-6.881	-2.453
Constant	5.422	0.277	19.56	0	4.879	5.966

MDI, multiple daily injections; DAFNE, dose adjustment for normal eating

beta scale – 0 is a HbA1c of 29 mmol/mol and 1 is a HbA1c of 201 mmol/mol

For peer review only - http://bmjopen.bmj.com/site/about/guidelines.xhtml

Table 15: The effect of continuous subcutaneous insulin infusion compared to multiple daily injections for all individuals in the per protocol population.

Hba1c at one year (beta scale)	Coefficient	Standard error	t	P>t	95% Confidence interval	
Mean effect (Mu) – using a logit link function						
Treatment allocation (1= pumps + DAFNE, 0=MDI + DAFNE)	-0.056	0.044	-1.37	0.171	-0.148	0.026
Baseline HbA1c (beta scale)	3.938	0.255	13.62	0	2.978	3.980
Constant	-2.219	0.093	-23.94	0	-2.401	-2.038
Centre effects (Cambridge is the reference site)						
Dumfries and Galloway	-0.019	0.078	-0.25	0.805	-0.172	0.134
Edinburgh	0.020	0.056	0.37	0.714	-0.089	0.130
Glasgow	-0.129	0.095	-1.36	0.175	-0.315	0.057
Harrogate	0.025	0.040	0.62	0.534	-0.054	0.104
College Hospital - London	0.018	0.064	0.28	0.779	-0.107	0.143
Nottingham	0.172	0.039	4.46	0	0.096	0.247
Sheffield	0.084	0.064	1.31	0.191	-0.042	0.209
Dispersion parameter (phi) - using a natural logarithm link function						
Baseline HbA1c (beta scale)	-3.504	1.050	-3.34	0.001	-5.563	-1.446
Constant	5.062	0.351	14.41	0	4.373	5.751
Hba1c at two years (beta scale)						
Mean effect (Mu) – using a logit link function						
Treatment allocation (1= pumps + DAFNE, 0=MDI + DAFNE)	-0.047	0.035	-1.35	0.177	-0.116	0.021
One year HbA1c (beta scale)	3.475	0.340	10.23	0	2.809	4.141
Baseline HbA1c (beta scale)	1.053	0.351	3	0.003	0.365	1.740
Constant	-2.382	0.092	-26.01	0	-2.562	-2.203
Centre effects (Cambridge is the reference site)						
Dumfries and Galloway	0.022	0.088	0.26	0.799	-0.150	0.194
Edinburgh	0.076	0.085	0.89	0.374	-0.091	0.243
Glasgow	0.105	0.096	1.1	0.271	-0.082	0.293
Harrogate	0.092	0.085	1.08	0.28	-0.075	0.258
College Hospital - London	0.053	0.085	0.62	0.538	-0.115	0.220
Nottingham	0.109	0.100	1.1	0.276	-0.089	0.308
Sheffield	0.157	0.078	2.02	0.043	0.005	0.310
Dispersion parameter (phi) - using a natural logarithm link function						
One year HbA1c (beta scale)	-4.809	1.231	-3.9	0	-7.223	-2.394
Constant	5.474	0.302	18.13	0	4.882	6.066

pumps, continuous subcutaneous insulin infusion; MDI, multiple daily injections,
Beta scale – 0 is a HbA1c of 29 mmol/mol and 1 is a HbA1c of 201 mmol/mol

For peer review only

Table 16: The variance covariance matrix for the beta regression to predict one year HbA1c in the ITT population

		Mu										Ln Phi	
		Treatment allocation	Baseline HbA1c (Beta scale)	Dumfries and Galloway	Edinburgh	Glasgow	Harrogate	King's College Hospital – London	Nottingham	Sheffield	Constant	Baseline HbA1c (Beta scale)	Constant
Mu	Treatment allocation	0.001											
	Baseline HbA1c (Beta scale)	0.004	0.062										
	Dumfries and Galloway	-0.001	-0.003	0.006									
	Edinburgh	0.000	0.002	0.001	0.004								
	Glasgow	0.001	0.002	0.001	0.001	0.010							
	Harrogate	0.000	0.001	0.001	0.001	0.002	0.002						
	King's College Hospital – London	0.000	0.004	0.001	0.002	0.002	0.001	0.004					
	Nottingham	0.000	-0.004	0.001	0.001	0.001	0.001	0.001	0.004				
	Sheffield	0.000	0.000	0.001	0.001	0.001	0.001	0.001	0.001	0.003			
	Constant	-0.002	-0.019	0.000	-0.002	-0.002	-0.002	-0.003	0.000	-0.001	0.008		
Phi	Baseline HbA1c (Beta scale)	-0.015	-0.110	0.002	-0.010	-0.027	-0.006	-0.021	0.007	-0.007	0.050	0.996	
	Constant	0.005	0.038	0.000	0.003	0.009	0.000	0.004	0.000	0.002	-0.017	-0.308	0.110

Mu – Mean effect (Mu) – using a logit link function; Ln Phi - Dispersion parameter (phi) - using a natural logarithm link function; * - 1 = pumps, 0 = multiple daily injections; Beta scale – 0 is a HbA1c of 29 mmol/mol and 1 is a HbA1c of 201 mmol/mol

For peer review only - http://bmjopen.bmj.com/site/about/guidelines.xhtml

Table 17**:** The variance covariance matrix for the beta regression to predict two year HbA1c in the ITT population

		Mu											ln Phi	
		Treatment allocation *	One year HbA1c (beta scale)	Baseline HbA1c (Beta scale)	Dumfries and Galloway	Edinburgh	Glasgow	Harrogate	King's College Hospital - London	Nottingham	Sheffield	Constant	One year HbA1c (beta scale)	Constant
Mus	Treatment allocation*	0.001												
	One year HbA1c (beta scale)	-0.001	0.101											
	Baseline HbA1c (Beta scale)	0.001	-0.071	0.117										
	Dumfries and Galloway	-0.001	-0.004	0.002	0.009									
	Edinburgh	0.000	-0.006	0.003	0.006	0.007								
	Glasgow	0.000	-0.014	0.005	0.006	0.006	0.009							
	Harrogate	0.000	-0.002	-0.002	0.006	0.006	0.006	0.008						
	King's College Hospital - London	0.000	-0.002	0.002	0.006	0.006	0.006	0.006	0.008					
	Nottingham	0.000	-0.006	-0.004	0.006	0.006	0.007	0.006	0.006	0.012				
	Sheffield	0.000	-0.005	0.001	0.006	0.006	0.006	0.006	0.006	0.006	0.006			
	Constant	0.000	-0.004	-0.010	-0.005	-0.005	-0.004	-0.005	-0.006	-0.004	-0.005	0.008		
ln Phi	One year HbA1c (beta scale)	-0.002	-0.014	-0.085	-0.020	-0.006	-0.002	-0.005	-0.010	-0.012	0.000	0.032	1.276	
	Constant	0.000	0.011	0.020	0.005	0.002	0.000	0.003	0.003	0.003	0.000	-0.010	-0.291	0.077

Mu – Mean effect (Mu) – using a logit link function; Ln Phi - Dispersion parameter (phi) - using a natural logarithm link function; * - 1 = pumps, 0 = multiple daily injections; Beta scale – 0 is a HbA1c of 29 mmol/mol and 1 is a HbA1c of 201 mmol/mol

For peer review only - http://bmjopen.bmj.com/site/about/guidelines.xhtml

Table 18: The variance covariance matrix for the beta regression to predict one year HbA1c in the per protocol population

		Mu										Ln Phi	
		Treatment allocation	Baseline HbA1c (Beta scale)	Dumfries and Galloway	Edinburgh	Glasgow	Harrogate	King's College Hospital – London	Nottingham	Sheffield	Constant	Baseline HbA1c (Beta scale)	Constant
Mu	Treatment allocation	0.001											
	Baseline HbA1c (Beta scale)	0.004	0.084										
	Dumfries and Galloway	-0.001	-0.005	0.006									
	Edinburgh	0.000	0.001	0.001	0.003								
	Glasgow	0.000	0.000	0.002	0.002	0.009							
	Harrogate	0.000	0.000	0.001	0.001	0.001	0.002						
	King's College Hospital – London	0.000	0.004	0.001	0.001	0.002	0.001	0.004					
	Nottingham	0.000	-0.002	0.001	0.001	0.002	0.001	0.001	0.001				
	Sheffield	0.000	-0.003	0.001	0.001	0.002	0.001	0.001	0.001	0.004			
	Constant	-0.002	-0.024	0.000	-0.002	-0.002	-0.001	-0.002	-0.001	-0.001	0.009		
Phi	Baseline HbA1c (Beta scale)	-0.018	-0.117	-0.006	-0.015	-0.031	-0.010	-0.028	-0.017	-0.016	0.060	1.103	
	Constant	0.007	0.040	0.002	0.004	0.010	0.001	0.006	0.005	0.005	-0.020	-0.343	0.123

Mu – Mean effect (Mu) – using a logit link function; Ln Phi - Dispersion parameter (phi) - using a natural logarithm link function; * - 1 = pumps, 0 = multiple daily injections; Beta scale – 0 is a HbA1c of 29 mmol/mol and 1 is a HbA1c of 201 mmol/mol

For peer review only - http://bmjopen.bmj.com/site/about/guidelines.xhtml

For peer review only

Table 19: The variance covariance matrix for the beta regression to predict two year HbA1c in the per protocol population

		Mu											ln Phi	
		Treatment allocation *	One year HbA1c (beta scale)	Baseline HbA1c (Beta scale)	Dumfries and Galloway	Edinburgh	Glasgow	Harrogate	King's College Hospital - London	Nottingham	Sheffield	Constant	One year HbA1c (beta scale)	Constant
Mus	Treatment allocation*	0.001												
	One year HbA1c (beta scale)	-0.001	0.123											
	Baseline HbA1c (Beta scale)	0.001	-0.075	0.115										
	Dumfries and Galloway	-0.001	-0.005	0.003	0.008									
	Edinburgh	0.000	-0.008	0.005	0.006	0.007								
	Glasgow	0.000	-0.016	0.007	0.006	0.006	0.009							
	Harrogate	0.000	-0.004	0.000	0.006	0.006	0.006	0.007						
	King's College Hospital - London	0.000	-0.003	0.004	0.005	0.005	0.005	0.005	0.007					
	Nottingham	0.000	-0.009	-0.001	0.005	0.005	0.006	0.006	0.005	0.010				
	Sheffield	0.000	-0.006	0.001	0.005	0.005	0.006	0.005	0.005	0.005	0.006			
	Constant	0.000	-0.007	-0.010	-0.005	-0.005	-0.003	-0.004	-0.005	-0.003	-0.004	0.008		
ln Phi	One year HbA1c (beta scale)	0.008	-0.096	-0.044	-0.017	-0.008	0.000	0.002	-0.009	0.010	0.004	0.038	1.516	
	Constant	-0.003	0.034	0.009	0.004	0.002	-0.002	0.000	0.002	-0.004	-0.002	-0.011	-0.343	0.091

Mu – Mean effect (Mu) – using a logit link function; Ln Phi - Dispersion parameter (phi) - using a natural logarithm link function; * - 1 = pumps, 0 = multiple daily injections; Beta scale – 0 is a HbA1c of 29 mmol/mol and 1 is a HbA1c of 201 mmol/mol

For peer review only

For peer review only - http://bmjopen.bmj.com/site/about/guidelines.xhtml

5.3 DKA and severe hypoglycaemia

To develop the method to incorporate severe hypoglycaemic events and DKA treatment effect evidence into the model, several factors were considered. Data on severe hypoglycaemic events and DKA were collected on an ongoing basis throughout the trial. Self-reported information was also collected on the incidence of DKA. A summary of the numbers of DKAs and severe hypoglycaemic events is given in Table 20. It can be seen that the number of DKAs and severe hypoglycaemic events declines in the second year on every measure, except self-reported DKAs in the MDI+DAFNE arm were the number events was the same in both years. As such, the statistical models used in the economic data estimated the incidence of severe hypoglycaemia and DKA in the first and second years separately.

For peer review only

For peer review only - http://bmjopen.bmj.com/site/about/guidelines.xhtml

Table 20: A summary of the observed incidence of diabetic ketoacidosis and severe hypoglycaemia in the intention to treat population

	Year 1			Year 2			Total		
	pumps + DAFNE (n=132)	MDI + DAFNE (n=128)	Total (n=260)	pumps + DAFNE (n=132)	MDI + DAFNE (n=128)	Total (n=260)	pumps + DAFNE (n=132)	MDI + DAFNE (n=128)	Total (n=260)
DKAs - Serious Adverse events									
Number(%) participants with ≥1 DKA	15(11.4%)	1(0.8%)	16 (6.2%)	4(3.0%)	2(1.5%)	6(2.2%)	17(12.9%)	3(2.3%%)	20(7.7%)
Number of hospital admissions	16	5	21	5	4	9	21	9	30
DKAs - Self-reported admissions									
Number(%) participants with ≥1 DKA	17 (12.9%)	6(4.7%)	23 (8.8%)	6(4.5%)	5(3.7%)	11(4.1%)	18 (13.6%)	8(6.3%)	26(10.0%)
Number of self-reported DKAs	24	11	35	7	11	18	26*	13*	39*
Severe hypoglycaemia									
Number(%) participants with ≥1 severe hypo	10(7.6%)	9(7.0%)	19(7.3%)	4(3.0%)	7(5.5%)	11(4.2%)	14(10.6%)	11(8.6%)	25(9.6%)
Number of severe hypoglycaemic events	21	12	33	4	12	16	25	24	49

DAFNE, dose adjustment for normal eating; MDI, multiple daily injections; DKA, diabetic ketoacidosis; * these values are not the sums of the one and two year follow up as some individuals had missing information in either the first or second year.

For peer review only - http://bmjopen.bmj.com/site/about/guidelines.xhtml

Negative binomial regressions were used to predict the number of DKAs, and severe hypoglycaemic events in years 1 and 2 for each outcome separately. When the outcome variable was the number of severe hypos in year 1, year 1 HbA1c and treatment group were included as covariates. When the outcome variable was the number of severe hypos in year 2, year 2 HbA1c and treatment group were included as covariates When the outcome variable was the number of DKAs in year 1, year 1 HbA1c and treatment group were included as covariates When the outcome variable was the number of DKAs in year 2, year 2 HbA1c and treatment group were included as covariates. The possibility of using the number of events in the previous year, baseline events for the 1 year outcomes and year 1 events for the 2 year outcomes, as a covariate was explored. However, due to the low number of events, the negative binomial models often did not converge when this was included as a covariate.

The statistical models did not converge for DKAs reported as serious adverse events in the first year. This was not the case for self-reported DKAs and there were more self-reported cases of DKA than were picked up through the reporting of serious adverse events. Therefore, the rates of DKA were estimated using self-reported DKAs as the outcome measure.

The statistical models were fitted using the Zellig package in R version3.2.0 and using specifications described above; it was used to simulate the predicted number of severe hypoglycaemia and DKA events in each trial arm 10,000 times. The simulations were separately in each trial arm and for HbA1c values every 0.1% between 4% and 20.5%. The number of events observed in the simulations was truncated at 20 events per year to reduce the effect of extreme values in the simulation on the cost-effectiveness results. These simulations were then used to determine the probability that an individual would suffer a given number of severe hypoglycaemic events and DKA events in a year, dependent on their HbA1c that year and the trial arm they were allocated to. The probability that an individual would suffer a given number of events was a fixed parameter in the PSA, therefore any differences in the rates of DKA or severe hypoglycaemia for an individual between any two model runs will solely be due to differences in their HbA1c.

The results of the negative binomial regressions are given in Table 21 and Table 22.

For peer review only - http://bmjopen.bmj.com/site/about/guidelines.xhtml

Table 21: The negative binomial model fitted to the incidence of severe hypoglycaemia at one year and two years

	Coefficient	Standard error	z value	P>z
Severe hypoglycaemia in year 1				
Treatment allocation (1= pumps + DAFNE, 0=MDI + DAFNE)	0.2861	0.5149	0.556	0.578
One year HbA1c (DCCT % scale)	-0.5010	0.2323	2.157	0.03
Number of severe hypoglycaemic events experienced in the year prior to baseline	2.0708	0.5638	3.673	>0.000
Constant	1.2689	1.8676	0.679	0.49687
Severe hypoglycaemia in year 2				
Treatment allocation (1= pumps + DAFNE, 0=MDI + DAFNE)	-1.1141	0.7202	-1.547	0.122
Two year HbA1c (DCCT % scale)	-0.2019	0.2668	-0.757	0.449
Constant	-0.6367	2.2625	-0.281	0.778

DAFNE, dose adjustment for normal eating, MDI, multiple daily injections

Table 22: The negative binomial model fitted to the incidence of diabetic ketoacidosis at one and two years

	Coefficient	Standard error	z value	P>z
Diabetic ketoacidosis in year 1				
Treatment allocation (1= pumps + DAFNE, 0=MDI + DAFNE)	0.3369	0.4786	0.704	0.481
One year HbA1c (DCCT % scale)	0.4089	0.1246	3.283	0.001
Constant	-5.9443	1.1879	-5.004	>0.00
Diabetic ketoacidosis in year 2				
Treatment allocation (1= pumps + DAFNE, 0=MDI + DAFNE)	-0.07564	0.70426	-0.107	0.914
Two year HbA1c (DCCT % scale)	0.32667	0.19447	1.680	0.093
Number of DKAs in year 1	0.86618	0.51682	1.676	0.094
Constant	-5.98206	1.82156	-3.284	0.01

DAFNE, dose adjustment for normal eating, MDI, multiple daily injections; DKA, diabetic ketoacidosis

For peer review only

For peer review only - http://bmjopen.bmj.com/site/about/guidelines.xhtml

6 The cost of insulin, diabetes related contacts and insulin pumps used in the Sheffield Type 1 Diabetes model

Self-reported information was collected on individual's use of insulin, face to face contacts related to their diabetes with a health care professional and telephone contacts related to their diabetes with a health care professional. Data collected on insulin use included, the type of insulin used, the dose of insulin and the method of insulin delivery. Ongoing information was also collected on whether an individual switched their insulin delivery mechanism (either pumps or MDI). The cost of the diabetes related contacts were sourced from the NHS reference costs and were £105.49 for face to face contacts and £75.80 for telephone contacts.[8] Insulin costs were microcosted using the data in the british national formularly and a prescription costs analysis, the full list of unit costs is given in Table 23. [9 10] The unit costs of the pumps and their related consumables was obtained from a survey of the prices paid at the REPOSE trial sites.

For peer review only

Table 23: The unit costs of insulin

Item	Average unit cost	Number of units	Cost per unit	Associated yearly cost of an insulin pen	Source
Consumables related to multiple daily injections					
Cost of an insulin needle	£0.11	N/A	N/A	N/A	HSCIC[9]
Cost of an insulin syringe	£0.13	N/A	N/A	N/A	HSCIC[9]
Quick Acting Insulin					
Human Insulin					
Vial	£9.87	1000	£0.01	N/A	BNF[10], HSCIC[9]
Cartridges for a reusable pen	£18.97	1500	£0.01	£8.78	
Animal Insulin					
Vial	£26.15	1000	£0.03	N/A	BNF[10], HSCIC[9]
Cartridges for a reusable pen	£38.29	1500	£0.03	£5.97	
Insulin Aspart (NovoRapid)					
Vial	£14.08	1000	£0.01	N/A	BNF[10], HSCIC[9]
Cartridges for a reusable pen	£28.31	1500	£0.02	£9.59	
Disposable Pen	£30.63	1500	£0.02	N/A	
Insulin Lispro (Humalog)					
Vial	£16.61	1000	£0.02	N/A	
Cartridges for a reusable pen	£28.31	1500	£0.02	£8.86	BNF[10], HSCIC[9]
Disposable Pen	£28.31	1500	£0.02	N/A	
Insulin Glulisine (Apidra)					
Vial	£16.00	1000	£0.02	N/A	
Cartridges for a reusable pen	£28.30	1500	£0.02	£7.86	BNF[10], HSCIC[9]
Disposable Pen	£28.30	1500	£0.02	N/A	

Table 23: the unit costs of insulin (continued)

Item	Average unit cost	Number of units	Cost per unit	Associated yearly cost of an insulin pen	Source
	Background Insulin				
Human Insulin					
Vial	£10.41	988	£0.01	N/A	
Cartridges for a reusable pen	£21.52	1500	£0.01	£9.30	BNF[10], HSCIC[9]
Disposable Pen	£21.05	1500	£0.01	N/A	
Animal Insulin					
Vial	£26.17	1000	£0.03	N/A	BNF[10], HSCIC[9]
Cartridges for a reusable pen	£38.32	1500	£0.03	£9.57	
Insulin Detemir (Levemir)					
Cartridges for a reusable pen	£42.00	1500	£0.03	£9.59	BNF[10], HSCIC[9]
Disposable Pen	£42.10	1500	£0.03	N/A	
Insulin Glargine (Lantus)					
Vial	£30.68	1000	£0.03	N/A	
Cartridges for a reusable pen	£41.50	1500	£0.03	£7.86	BNF[10], HSCIC[9]
Disposable Pen	£41.50	1500	£0.03	N/A	
Mixed Insulin					
Biphasic Isophane Insulin					
Animal Insulin					
Vial	£25.20	1000	£0.03	N/A	BNF[10], HSCIC[9]
Cartridges for a reusable pen	£37.80	1500	£0.03	£5.97	
Human Insulin					
Vial	£15.43	987	£0.02	N/A	BNF[10], HSCIC[9]
Cartridges for a reusable pen	£18.94	1500	£0.01	£7.74	
Disposable Pen	£21.43	1500	£0.01	N/A	
Biphasic Insulin Aspart					
Cartridges for a reusable pen	£28.79	£28.79	£0.02	£9.59	BNF[10], HSCIC[9]
Disposable Pen	£29.89	£29.89	£0.02		
Biphasic Insulin Lispro					
Vial	£16.61	1000	£0.02		
Cartridge for reusable pen	£29.03	1500	£0.02	£8.93	BNF[10], HSCIC[9]
Disposable Pen	£30.13	1500	£0.02		

BNF, British National Formulary; HSCIC, Health & Social Care Information Centre

For peer review only

The cost of insulin, diabetes related contacts and insulin pumps (including consumables) for insulin pump therapy individuals were based on resource use data from the REPOSE trial data. It is expected that the covariates which predict the cost of insulin in year 1 may be correlated with the covariates which predict the cost of insulin in year 2. It is also expected that this may be true for the cost of diabetes related contacts and the cost of insulin pumps (including consumables). Therefore, instead of fitting six independent regression models, three seemingly unrelated regressions were fitted (one seemingly unrelated regression for the cost of insulin, another for the cost of diabetes related contacts and finally one for the cost of insulin pumps (including consumables).

In the cost insulin seemingly unrelated regression model, the cost of insulin in year 1 and the cost of insulin in year 2 were used as the outcome variables for the seemingly unrelated regression model. Baseline cost of insulin, baseline HbA1c, treatment allocation, whether the individual switched from multiple daily injections to insulin pump infusion in year one and whether or not the individual switched from insulin pump infusion to multiple daily injections in year 1 were included as covariates to predict the cost of insulin in year 1. Baseline cost of insulin, baseline HbA1c, the actual method of insulin delivery that an individual was using at the end of the first year, whether the individual switched from multiple daily injections to insulin pump infusion in year two and whether or not the individual switched from insulin pump infusion to multiple daily injections in year two were included as covariates to predict the cost of insulin in year two. The standard errors were adjusted for clustering in each DAFNE course.

In the cost of diabetes related contacts seemingly unrelated regression model, the cost of diabetes related contacts in year 1 and the cost of diabetes related contacts in year 2 were used as the outcome variables for the seemingly unrelated regression model. Baseline cost of diabetes related contacts, baseline HbA1c, and treatment allocation; whether the individual switched from multiple daily injections to insulin pump infusion in year one and whether or not the individual switched from insulin pump infusion to multiple daily injections in year 1 were included as covariates to predict the cost of insulin in year 1. Baseline cost of diabetes related contacts, baseline HbA1c, the actual method of insulin delivery that an individual was using at the end of the first year, whether the individual switched from multiple daily injections to insulin pump infusion in year two and whether or not the individual switched from insulin pump infusion to multiple daily injections in year two were included as covariates to predict the cost of insulin in year two. The standard errors were adjusted for clustering in each DAFNE course.

In the cost of insulin pump seemingly unrelated regression model, the cost of insulin pumps and consumables in year 1 and the cost insulin pumps and consumables in year 2 were the two outcome variables used in the model. No control was made for baseline resource use or baseline HbA1c for either outcome variable, as no individual in the REPOSE trial had previous history of using an insulin pump. Individual's randomised treatment arm, whether or not they switched from pumps to MDI in the first year and whether or not they switched from MDI to pumps in the first year were included as covariates to predict the cost of insulin pumps and consumables in year 1. Individual's actual treatment at the end of the first year, whether or not they switched from pumps to MDI in year 2 and whether or not they switched from MDI to pumps in year 2 were included as covariates to predict the cost of insulin pumps and consumables in year 2.

The results of the regressions are given in main text (Table 2). The uncertainty in the costs estimated by the seemingly unrelated regressions were included in the model's PSA using a multivariate normal

For peer review only - http://bmjopen.bmj.com/site/about/guidelines.xhtml

distribution. The variance covariance matrices used to parameterise the uncertainty in the cost parameters is given in TablesTable 24 toTable 26.

For peer review only - http://bmjopen.bmj.com/site/about/guidelines.xhtml

Table 24: The variance covariance matrix for the seemingly unrelated regression on insulin costs in year 1 and 2 of REPOSE

		Insulin costs year 1					Insulin costs ongoing					
		Baseline insulin cost	Baseline HbA1c (DCCT(%))	Receiving CSII at the start of the year	Switch from CSII to MDI	Constant	Baseline insulin cost	Baseline HbA1c (DCCT(%))	Receiving CSII at the start of the year	Switch from CSII to MDI	Switch from MDI to CSII	Constant
Insulin costs year 1	Baseline insulin cost	0.02										
	Baseline HbA1c (DCCT(%))	0.05	44.17									
	Receiving CSII at the start of the year	0.20	-27.12	653.72								
	Switch from CSII to MDI	3.86	220.49	-696.50	13054.87							
	Constant	-7.41	-404.77	-233.28	-2850.97	6465.05						
Insulin costs ongoing	Baseline insulin cost	0.01	0.17	-0.05	2.47	-6.18	0.01					
	Baseline HbA1c (DCCT(%))	0.10	49.04	-31.68	13.16	-461.41	0.28	75.98				
	Receiving CSII at the start of the year	-0.09	-20.08	668.10	-909.54	-225.18	-0.22	-41.08	913.07			
	Switch from CSII to MDI	0.30	54.23	279.72	-1171.31	-770.00	0.71	70.30	220.46	3131.19		
	Switch from MDI to CSII	0.96	14.50	200.44	285.09	-666.29	1.01	-18.58	327.56	-6.80	6409.60	
	Constant	-5.61	-484.99	-153.96	-391.66	6527.95	-6.52	-744.68	-201.06	-1115.05	-517.86	9117.89

For peer review only

Table 25: The variance covariance matrix for the seemingly unrelated regression on the cost of diabetes related contacts with health care professionals in year 1 and 2 of REPOSE

		Diabetes related contacts (DRC) in year 1						Diabetes related contacts (ongoing)					
		Baseline DRC cost	Baseline HbA1c	Receiving CSII at the start of the year	Switch from CSII to MDI	Switch from MDI to CSII	Constant	Baseline DRC cost	Baseline HbA1c (%)	Receiving CSII at the start of the year	Switch from CSII to MDI	Switch from MDI to CSII	Constant
Diabetes related contacts (DRC) in year 1	Baseline DRC cost	0.00											
	Baseline HbA1c (%)	-0.37	429.26										
	Receiving CSII at the start of the year	0.90	-414.71	4671.82									
	Switch from CSII to MDI	1.79	-3373.14	-6556.03	135705.03								
	Switch from MDI to CSII	8.01	-3893.52	20945.51	-65526.50	401879.74							
	Constant	2.08	-3491.16	1632.08	32468.75	14206.81	30605.56						
Diabetes related contacts (ongoing)	Baseline DRC cost	0.00	-0.10	0.39	-0.34	3.98	0.33	0.00					
	Baseline HbA1c (%)	-0.09	154.18	-31.36	-1626.90	-181.76	-1365.08	-0.07	633.97				
	Receiving CSII at the start of the year	0.04	-111.02	2245.82	-7211.76	14536.08	480.91	0.01	-339.62	4784.66			
	Switch from CSII to MDI	0.08	251.49	294.56	-3310.51	-9569.30	-2251.13	0.17	-570.77	-1568.74	4477.66		
	Switch from MDI to CSII	0.95	-28.09	174.70	-9237.83	3330.56	-448.54	0.87	-926.86	2204.36	2044.24	23540.78	
	Constant	0.67	-1479.09	-339.05	22162.60	-1783.64	13811.74	0.06	-5516.22	1364.46	4819.50	4946.57	50120.60

For peer review only

Table 26: The variance covariance matrix for the seemingly unrelated regression on the cost of insulin pumps and associated consumables in year 1 and 2 of REPOSE

		Insulin pump and consumables costs (year 1)				Insulin pump and consumables costs (ongoing)			
		Receiving CSII at the start of the year	Switch from CSII to MDI	Switch from MDI to CSII	Constant	Receiving CSII at the start of the year	Switch from CSII to MDI	Switch from MDI to CSII	Constant
Insulin pump and consumables costs (year 1)	Receiving CSII at the start of the year	241.41							
	Switch from CSII to MDI	1990.29	82619.62						
	Switch from MDI to CSII	16.58	-627.60	43661.77					
	Constant	1.41E-04	0.01	-5.10E-04	1.40E-08				
Insulin pump and consumables costs (ongoing)	Receiving CSII at the start of the year	200.78	718.30	180.58	-3.30E-04	190.07			
	Switch from CSII to MDI	361.04	-89.20	-528.03	-0.01	535.66	51325.97		
	Switch from MDI to CSII	-3.84	3.83	-4213.99	4.38E-04	-30.22	24.09	23307.42	
	Constant	-4.20E-04	0.01	-1.24E-03	1.53E-08	-8.70E-04	-0.01	1.52E-03	2.02E-08

For peer review only

7 Detailed results of the scenario and subgroup analyses

Table 27: The One way scenario analyses and subgroup analyses performed using the Sheffield Type 1 Diabetes Policy Model

	MDI + DAFNE		pumps + DAFNE		Incremental		
	Total discounted costs	Total discounted QALYs	Total discounted costs	Total discounted QALYs	Total discounted costs	Total discounted QALYs	ICER (£ per QALY gained)
Base Case - PSA	£81,785	12.98	£100,617	13.11	£18,832	0.13	£149,483
Base Case - Deterministic	£73,963	12.81	£93,236	12.98	£19,273	0.18	£109,684
Scenario - Pump prices were estimated from Riemsma et al.[11]	£73,912	12.81	£92,414	12.98	£18,502	0.18	£105,295
Scenario - 25% price reduction in insulin pumps and consumables	£73,513	12.81	£85,939	12.98	£12,426	0.18	£70,715
Scenario - 50% price reduction in insulin pumps and consumables	£73,063	12.81	£78,641	12.98	£5,578	0.18	£31,747
Scenario – Intention to treat estimate of treatment effect	£74,200	12.90	£94,400	12.96	£20,200	0.06	£316,785
Scenario - Intention to treat estimate of treatment effect and no change in HbA1c if an individual switches treatment	£73,308	12.93	£93,496	12.96	£20,188	0.04	£534,397
Scenario – Base case & no change in HbA1c when switching	£73,799	12.86	£93,329	12.95	£19,530	0.09	£207,874
Scenario – Post-trial HbA1c progression in both arms is estimated from the DCCT	£74,278	12.83	£92,865	13.00	£18,586	0.18	£106,126
Scenario - Individuals return to their baseline HbA1c after 3 years and no progression thereafter	£70,053	13.10	£91,063	12.99	£21,011	-0.10	Dominated
Scenario - HbA1c effects occur one model cycle earlier	£75,015	12.78	£93,276	12.92	£18,262	0.14	£130,208
Scenario – individuals return to their baseline risk of hypos and DKA at three years	£73,716	12.86	£95,012	12.83	£21,296	-0.02	Dominated
Scenario - Switching probabilities were estimated directly from the Kaplan - Meier curves	£73,516	12.83	£93,911	12.94	£20,394	0.12	£172,836
Scenario – The utility decrement for blindness was estimated from the Brown *et al*. study.[12]	£73,963	12.80	£93,236	12.97	£19,273	0.18	£110,115

For peer review only - http://bmjopen.bmj.com/site/about/guidelines.xhtml

Table 27: The One way scenario analyses and subgroup analyses performed using the Sheffield Type 1 Diabetes Policy Model (continued)

	MDI + DAFNE		pumps + DAFNE		Incremental		
	Total discounted costs	Total discounted QALYs	Total discounted costs	Total discounted QALYs	Total discounted costs	Total discounted QALYs	ICER (£ per QALY gained)
Subgroup - individuals with a baseline HbA1c < 8.5%	£57,947	13.38	£79,001	13.42	£21,054	0.04	£547,504
Subgroup- individuals with a baseline HbA1c ≥ 8.5%	£85,028	12.32	£103,990	12.40	£18,962	0.07	£253,352
Subgroup - individuals with a baseline HbA1c ≥ 7.5%	£76,735	12.58	£95,481	12.74	£18,746	0.16	£120,239
Subgroup - Individuals with a baseline HbA1c ≥ 7.5% & <8.5%	£61,207	13.07	£82,337	13.19	£21,131	0.12	£176,887
Subgroup - Individuals with a baseline HbA1c ≥ 8.5% & <9.5%	£66,520	13.47	£86,105	13.60	£19,584	0.13	£148,240
Subgroup - Individuals with a baseline HbA1c ≥ 9.5%	£99,249	11.64	£115,473	11.81	£16,224	0.17	£96,231
Subgroup - Individuals in the per protocol population	£72,955	12.72	£92,351	12.88	£19,395	0.17	£115,786
Subgroup - Individuals in the per protocol population and no treatment switching	£70,975	12.78	£95,905	12.86	£24,929	0.09	£286,769

MDI - multiple daily injections; DAFNE - dose adjustment for normal eating; pumps; ICER - incremental cost-effectiveness ratio; PSA - probabilistic sensitivity analysis; DCCT - Diabetes Control and Complications Trial

For peer review only - http://bmjopen.bmj.com/site/about/guidelines.xhtml

References

1. Office for National Statistics. National Life Tables, United Kingdom : 2012–2014. *Online Source* 2016;Available from: http://www.ons.gov.uk/peoplepopulationandcommunity/birthsdeathsandmarriages/lifeexpectancies/bulletins/nationallifetablesunitedkingdom/previousReleases . (Last Accessed: 17/03/2016)

2. Office for National Statistics. Deaths Registered in England and Wales (Series DR). Table 5 for years 2014, 2013 and 2012. *Online Source* 2015;Available from: http://www.ons.gov.uk/peoplepopulationandcommunity/birthsdeathsandmarriages/deaths/datasets/deathsregisteredinenglandandwalesseriesdrreferencetables(Last Accessed: 17/03/2016)

3. National Records of Scotland. Vital Events Reference Tables. *Online Source* 2015;Available from: http://www.nrscotland.gov.uk/statistics-and-data/statistics/statistics-by-theme/vital-events/general-publications/vital-events-reference-tables/2014(Last Accessed 17/03/2016)

4. Northern Ireland Statistics and Research Agency. Microdata for Deaths in Northern Ireland, 2001-2014. *Online Source* 2014;Available from: http://www.nisra.gov.uk/archive/demography/publications/births_deaths/Pivot_Table_2001_2014.xlsx(Last Accessed 17/03/2016)

5. Heller S, Lawton J, Amiel S, et al. Improving management of type 1 diabetes in the UK: the Dose Adjustment For Normal Eating (DAFNE) programme as a research test-bed. A mixed-method analysis of the barriers to and facilitators of successful diabetes self-management, a health economic analysis, a cluster randomised controlled trial of different models of delivery of an educational intervention and the potential of insulin pumps and additional educator input to improve outcomes. *Programme grants for applied research* 2014;2(5) doi: 10.3310/pgfar02050

6. Wolfe RA, Ashby VB, Milford EL, et al. Comparison of mortality in all patients on dialysis, patients on dialysis awaiting transplantation, and recipients of a first cadaveric transplant. *N Engl J Med* 1999;341(23):1725-30. doi: 10.1056/NEJM199912023412303

7. Briggs A, Claxton K, Sculpher M. Decision Modelling for Health Economic Evaluation. Oxford: Oxford University Press 2006.

8. Department of Health. NHS reference costs 2013 to 2014. *Online Source* 2014;Available from; https://www.gov.uk/government/publications/nhs-reference-costs-2013-to-2014. Last accessed 17th July 2015

9. Health & Social Care Information Centre. Prescription Cost Analysis England 2014. *Online Source* 2015;Available from; http://www.hscic.gov.uk/catalogue/PUB17274. Last Accessed 2nd Novemeber 2015

10. British Medical Association and Royal Pharmaceutical Society of Great Britain. British National Formulary August 2015. *Online Source* 2015;Available from: https://www.medicinescomplete.com, Last Accessed 9th September 2015

11. Riemsma R, Corro Ramos I, Birnie R, et al. Type 1 diabetes: Integrated sensor-augmented pump therapy systems for managing blood glucose levels (The MiniMed Paradigm Veo System and the Vibe and G4 PLATINUM CGM system), a systematic review and economic evaluation. *Kleijnen Systematic Reviews Ltd* 2015

12. Brown MM, Brown GC, Sharma S, et al. Quality of life with visual acuity loss from diabetic retinopathy and age-related macular degeneration. *Arch Ophthalmol* 2002;120(4):481-4.

For peer review only - http://bmjopen.bmj.com/site/about/guidelines.xhtml

For peer review only

For peer review only - http://bmjopen.bmj.com/site/about/guidelines.xhtml

CHEERS Checklist
Items to include when reporting economic evaluations of health interventions

The **ISPOR CHEERS Task Force Report**, *Consolidated Health Economic Evaluation Reporting Standards (CHEERS)—Explanation and Elaboration: A Report of the ISPOR Health Economic Evaluations Publication Guidelines Good Reporting Practices Task Force,* provides examples and further discussion of the 24-item CHEERS Checklist and the CHEERS Statement. It may be accessed via the *Value in Health* or via the ISPOR Health Economic Evaluation Publication Guidelines – CHEERS: Good Reporting Practices webpage: http://www.ispor.org/TaskForces/EconomicPubGuidelines.asp

Section/item	Item No	Recommendation	Reported on page No/ line No
**Title and abstract**			
Title	1	Identify the study as an economic evaluation or use more specific terms such as "cost-effectiveness analysis", and describe the interventions compared.	pi, line 1 to 3
Abstract	2	Provide a structured summary of objectives, perspective, setting, methods (including study design and inputs), results (including base case and uncertainty analyses), and conclusions.	pii to piii, line 2
**Introduction**			
Background and objectives	3	Provide an explicit statement of the broader context for the study.<br>Present the study question and its relevance for health policy or practice decisions.	p1, lines 1 to 41<br>p1, line 64 to p2, line 6
**Methods**			
Target population and subgroups	4	Describe characteristics of the base case population and subgroups analysed, including why they were chosen.	p3, line 16 to 27 & p5, line 31 to p6 line 2
Setting and location	5	State relevant aspects of the system(s) in which the decision(s) need(s) to be made.	p2, lines 10 to 17
Study perspective	6	Describe the perspective of the study and relate this to the costs being evaluated.	p2, line 16 to 20
Comparators	7	Describe the interventions or strategies being compared and state why they were chosen.	p1, line 12 to 41
Time horizon	8	State the time horizon(s) over which costs and consequences are being evaluated and say why appropriate.	p2, line 16 to 17
Discount rate	9	Report the choice of discount rate(s) used for costs and outcomes and say why appropriate.	p2, line 19 to 20
Choice of health outcomes	10	Describe what outcomes were used as the measure(s) of benefit in the evaluation and their relevance for the type of analysis performed.	p2, line 13 to 15
Measurement of effectiveness	11a	*Single study-based estimates:* Describe fully the design features of the single effectiveness study and why the single study was a sufficient source of clinical effectiveness data.	p3, line 1 to 13 & p1, line 39 to p2, line 2

	11b	*Synthesis-based estimates:* Describe fully the methods used for identification of included studies and synthesis of clinical effectiveness data.	N/A
Measurement and valuation of preference based outcomes	12	If applicable, describe the population and methods used to elicit preferences for outcomes.	N/A
Estimating resources and costs	13a	*Single study-based economic evaluation:* Describe approaches used to estimate resource use associated with the alternative interventions. Describe primary or secondary research methods for valuing each resource item in terms of its unit cost. Describe any adjustments made to approximate to opportunity costs.	N/A
	13b	*Model-based economic evaluation:* Describe approaches and data sources used to estimate resource use associated with model health states. Describe primary or secondary research methods for valuing each resource item in terms of its unit cost. Describe any adjustments made to approximate to opportunity costs.	p4, line38 to p5, line 10
Currency, price date, and conversion	14	Report the dates of the estimated resource quantities and unit costs. Describe methods for adjusting estimated unit costs to the year of reported costs if necessary. Describe methods for converting costs into a common currency base and the exchange rate.	p4 lines 21 to 40
Choice of model	15	Describe and give reasons for the specific type of decision-analytical model used. Providing a figure to show model structure is strongly recommended.	p2, line 21 to 40
Assumptions	16	Describe all structural or other assumptions underpinning the decision-analytical model.	
Analytical methods	17	Describe all analytical methods supporting the evaluation. This could include methods for dealing with skewed, missing, or censored data; extrapolation methods; methods for pooling data; approaches to validate or make adjustments (such as half cycle corrections) to a model; and methods for handling population heterogeneity and uncertainty.	p3, line 29 to p4, line 36
**Results**			
Study parameters	18	Report the values, ranges, references, and, if used, probability distributions for all parameters. Report reasons or sources for distributions used to represent uncertainty where appropriate. Providing a table to show the input values is strongly recommended.	Tables 2&3 & Supplementary Material B: Tables 1, 2, 5, 6, 9 and 10
Incremental costs and outcomes	19	For each intervention, report mean values for the main categories of estimated costs and outcomes of interest, as well as mean differences between the comparator groups. If applicable, report incremental cost-effectiveness ratios.	p6 line 28 to 39
Characterising uncertainty	20a	*Single study-based economic evaluation:* Describe the effects of sampling uncertainty for the estimated incremental cost and incremental effectiveness parameters, together with the impact	N/A

		of methodological assumptions (such as discount rate, study perspective).	
	20b	*Model-based economic evaluation:* Describe the effects on the results of uncertainty for all input parameters, and uncertainty related to the structure of the model and assumptions.	p6, line33 to p7, line 12
Characterising heterogeneity	21	If applicable, report differences in costs, outcomes, or cost-effectiveness that can be explained by variations between subgroups of patients with different baseline characteristics or other observed variability in effects that are not reducible by more information.	p7 line16 to 18
**Discussion**			
Study findings, limitations, generalisability, and current knowledge	22	Summarise key study findings and describe how they support the conclusions reached. Discuss limitations and the generalisability of the findings and how the findings fit with current knowledge.	p7 line20 to p8 line 13
**Other**			
Source of funding	23	Describe how the study was funded and the role of the funder in the identification, design, conduct, and reporting of the analysis. Describe other non-monetary sources of support.	p10 lines2 to 8
Conflicts of interest	24	Describe any potential for conflict of interest of study contributors in accordance with journal policy. In the absence of a journal policy, we recommend authors comply with International Committee of Medical Journal Editors recommendations.	p10 lines 10 to 15

For consistency, the CHEERS Statement checklist format is based on the format of the CONSORT statement checklist

The **ISPOR CHEERS Task Force Report** provides examples and further discussion of the 24-item CHEERS Checklist and the CHEERS Statement. It may be accessed via the *Value in Health* link or via the ISPOR Health Economic Evaluation Publication Guidelines – CHEERS: Good Reporting Practices webpage: http://www.ispor.org/TaskForces/EconomicPubGuidelines.asp

The citation for the CHEERS Task Force Report is:
Husereau D, Drummond M, Petrou S, et al. Consolidated health economic evaluation reporting standards (CHEERS)—Explanation and elaboration: A report of the ISPOR health economic evaluations publication guidelines good reporting practices task force. Value Health 2013;16:231-50.
